# Engineering Transport Orbitals in Single-Molecule Junctions

**DOI:** 10.1021/acs.jpclett.2c01851

**Published:** 2022-09-27

**Authors:** Abdalghani Daaoub, Luca Ornago, David Vogel, Pablo Bastante, Sara Sangtarash, Matteo Parmeggiani, Jerry Kamer, Nicolás Agraït, Marcel Mayor, Herre van der Zant, Hatef Sadeghi

**Affiliations:** † School of Engineering, University of Warwick, CV4 7AL Coventry, United Kingdom; ‡ Kavli Institute of Nanoscience, Delft University of Technology, Lorentzweg 1, 2628 CJ Delft, The Netherlands; § Department of Chemistry, University of Basel, St. Johanns-Ring 19, 4056 Basel, Switzerland; ∥ Departamento de Física de la Materia Condensada, Universidad Autónoma de Madrid, E-28049 Madrid, Spain; ⊥ Department of Applied Science and Technology (DISAT), Politecnico di Torino, Corso Duca degli Abruzzi 24, 10129 Torino, Italy; ∇ Institute for Nanotechnology, Karlsruhe Institute of Technology (KIT), P.O. Box 3640, 76021 Karlsruhe, Germany; ○ Lehn Institute of Functional Materials (LIFM), School of Chemistry, Sun Yat-Sen University (SYSU), 510275 Guangzhou, China

## Abstract

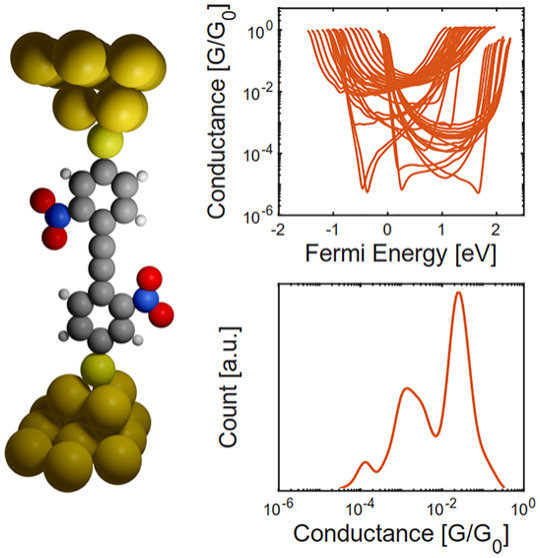

Controlling charge transport through molecules is challenging because it requires engineering of the energy of molecular orbitals involved in the transport process. While side groups are central to maintaining solubility in many molecular materials, their role in modulating charge transport through single-molecule junctions has received less attention. Here, using two break-junction techniques and computational modeling, we investigate systematically the effect of electron-donating and -withdrawing side groups on the charge transport through single molecules. By characterizing the conductance and thermopower, we demonstrate that side groups can be used to manipulate energy levels of the transport orbitals. Furthermore, we develop a novel statistical approach to model quantum transport through molecular junctions. The proposed method does not treat the electrodes’ chemical potential as a free parameter and leads to more robust prediction of electrical conductance as confirmed by our experiment. The new method is generic and can be used to predict the conductance of molecules.

Single-molecule junctions are of fundamental interest because molecules are the smallest objects that offer the structural diversity necessary to enable the integration of electronic functions through molecular design.^1−3^ Controlling and manipulating charge transport through molecules is challenging due to the sub-nanometer size of the molecular junctions. In traditional semiconductors, the chemical potential of the charge carrying band can be manipulated by doping with other elements, allowing for the engineering of their charge transport properties. The valence band and the conduction band of a semiconductor correspond to the HOMO and LUMO levels of a molecule. Therefore, employing the concept of band structure modulation by doping to molecular junctions requires energy level engineering of the molecular orbitals involved in the transport process. This can be realized by introducing substituents tuning the electron density of the molecule’s frontier orbitals. Variation of these substituents requires bottom-up synthesis of the desired structures.

While substituents are key in numerous molecular materials, they were less considered in single-molecule junctions. In most cases, flat delocalized π-systems were considered, and the major role of the substituents was to maintain solubility and processability.^4−6^ As a consequence, experimental studies of the effect of side groups on the electrical conductance of single molecules are largely focused on electrically inert side groups such as alkane or methoxy groups which are used for solubility.^4−6^ These side groups do not normally have an effect on the electrical conductance. For example, it was shown that the addition of two methoxy, hexyloxy, alkyl, alkoxy, fluoride, or *tert*-butyl side groups to a *para*-connected oligo-phenylene-ethynylenes (OPE3) backbone has no significant effect on the electrical conductance compared to that of the unsubstituted OPE3.^4,5,7−9^ Although methoxy substituents do not have an effect on the electrical conductance, they increase the Seebeck coefficient by ∼3 μV/K.^9^ Furthermore, small conductance variations were reported for a series of monosubstituted benzene-1,4-diamines with electron-donating and electron-withdrawing substituents.^10^ Side groups can also influence the conductance indirectly by changing the conformation of the molecule^11,12^ or the quantum interference pattern through them.^13−16^

Tolanes, the smallest structural variant of OPE derivatives (Figure 1a), are highly conjugated, chemically versatile, and known to be well behaved in molecular junction measurements.^17^ Therefore, in this paper, single tolane junctions are systematically studied both theoretically and experimentally to examine the effect of side groups on their electrical conductance and Seebeck coefficient (Figure 1a). For the experimental investigation, the tolanes are terminally decorated with either thiol- (series **a** in Figure 1b) or methylsulfide- (series **b** in Figure 1b) anchor groups and integrated between two gold electrodes. We chose two different anchor groups because the electronic coupling to the electrodes depends on the anchor groups. In particular, the electronic coupling through the sulfur atom is significantly affected by the gate-way orbitals^18^ that are present in molecules with an SAc anchor (due to the 1Au–S configuration) but are absent in molecules with an SMe anchor. To tune the energy level of the parent tolane structure **1**, either electron-withdrawing nitro groups in **2**, or electron-donating dimethylamino groups in **3** are introduced (Figures 1b and 2a). Similar to tuning the Fermi level by doping in semiconductors, in what follows, we demonstrate that the molecular transport levels can be tuned by these substituents. This is confirmed by electrical conductance measurements using scanning tunnelling microscope break junction (STMBJ) and mechanically controlled break junction (MCBJ) techniques. Moreover, Seebeck coefficient measurements using STMBJ and quantum transport calculations using first-principle methods support this finding.

**Figure 1:**
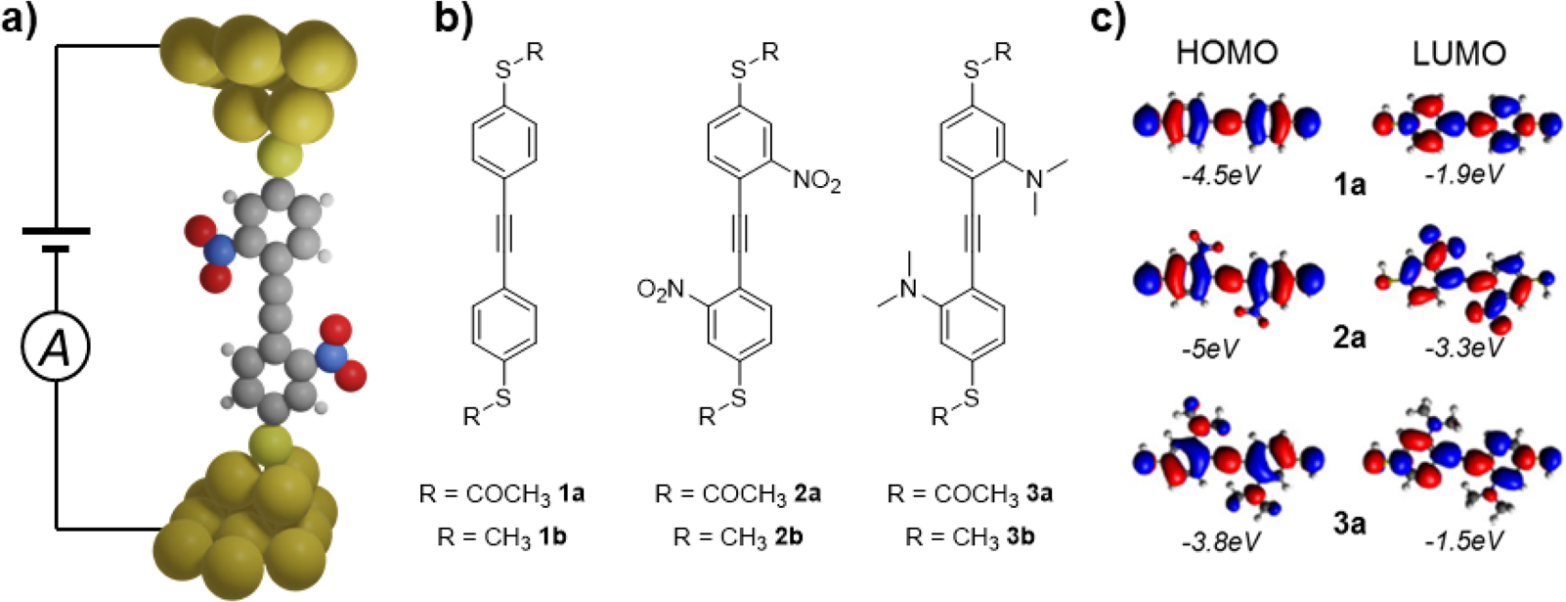
Schematic of molecular junctions formed. (a) An example of a tolane molecule with a nitro substituent and thiol anchor group connected to two gold electrodes; (b) the series **1**–**3** of tolane model compounds with acetyl masked thiol anchor groups (a) and methylsulfide anchor groups (b); (c) frontier molecular orbitals of **1a**, **2a**, **3a**.

**Figure 2:**
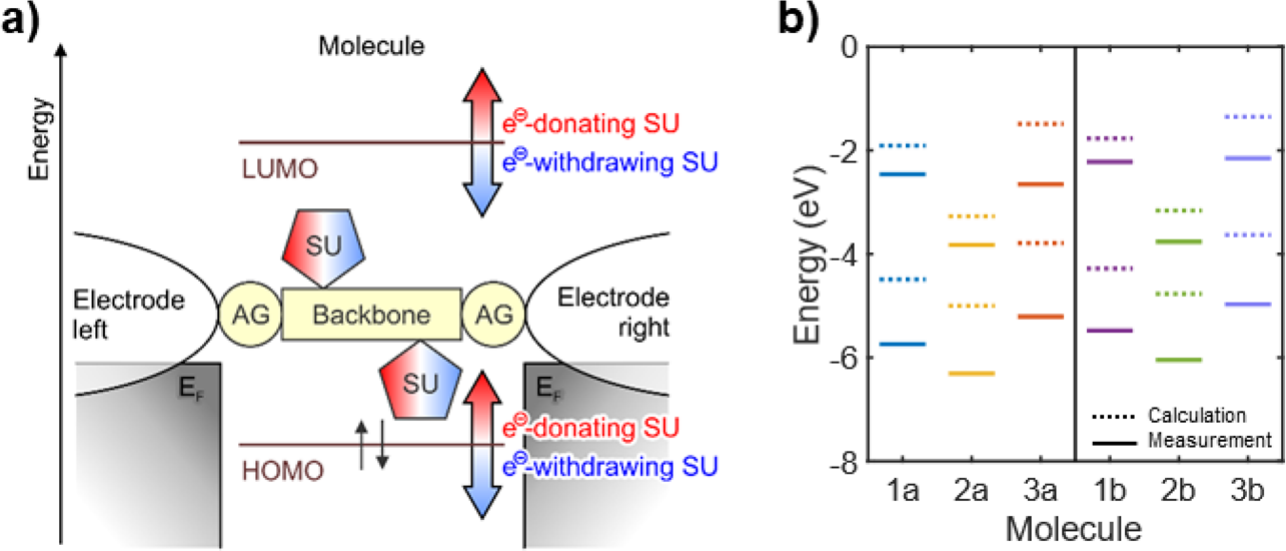
Energy level alignment (a) A simplified sketch of a molecular junction where the molecule’s backbone is linked over anchor groups (AG) to the adjacent electrodes. The parent molecule may be decorated by additional substituents (SU), e.g., electron-donating and -withdrawing groups and (b) energy level diagram with HOMO/LUMO levels of the tolanes **1a**, **1b**, **2a**, **2b**, **3a**, and **3b**. The solid (dashed) line shows the HOMO/LUMO levels determined by CV (calculations using first-principle methods).

The experimentally obtained data are further processed by a machine learning clustering approach (see Methods). This revealed conductance features that cannot be explained using traditional quantum transport calculation methods using only a given junction configuration. To elucidate these hidden trends in our experiment, we developed a novel statistical approach to model quantum transport through junctions. The proposed method is significant because it does not treat electrodes’ Fermi energy as a free parameter and can be used across the molecular electronics community and beyond to avoid the ambiguity in the predictions of conductance values.

In what follows, our aim is to systematically investigate the effect of substituents on the conductance by the use of a small series of tolane model compounds **1**–**3** (Figure 1b). We refer to this practice as “molecular doping” or better “frontier orbital engineering (FOE)”. The tolane structure combines compactness and good π-conjugation enabling their detection as a single molecule in MCBJ and STMBJ experiments. They also offer enough space to introduce substituents for the envisaged FOE. To tune the energy level of the frontier orbitals, either two nitro or two dimethylamino groups were introduced in the *ortho*-position to the acetylene to complement the unsubstituted parent structure **1** with **2** and **3**, respectively (Figure 1b).

The introduction of electron-withdrawing substituents like nitro^19,20^ groups moves the position of both frontier orbitals down in energy, while electron-donating substituents such as dimethylamino groups move them up (Figure 1c). Our cyclic voltammetry (CV) measurements and molecular energy calculations using first-principle methods (see Computational Methods) verify this as shown in Figure 2b. The energy levels of the highest occupied molecular orbital (HOMO) and the lowest unoccupied molecular orbital (LUMO) predicted by calculations are in good agreement with the ones determined by the CV measurement (Figure 2b). However, there is a systematic offset between the measured and the calculated data, and the calculated gap size seems to be smaller. It is well-known that density functional theory underestimates the gap size.^21^ The electrochemical analyses however have to be handled with care as irreversible voltammograms (Figure S1.81) have been recorded, and thus, the determined onset/offset potential used to calculate the HOMO and LUMO level introduces a higher uncertainty. Nevertheless, the gap size determined by CV and by UV–vis are comparable, verifying the experimentally determined HOMO/LUMO levels. All calculated and measured data are listed in Table S1.1 of the Supporting Information (SI). Furthermore, to study the effect of anchor groups on FOE, the tolane series **1**–**3** are synthesized with acetyl masked thiol anchor groups (series **a**) as well as with methylsulfide anchor groups (series **b**). The detailed synthetic procedures are described in the Supporting Information. Acetyl masked thiol anchor groups enable covalent thiol-gold bonds upon in situ deprotection during the immobilization of the molecule, whereas methylsulfide anchor groups form transient molecular junctions based on less stable coordinative bonds to the electrode.

We characterize the charge transport properties of the tolane series using both MCBJ and STMBJ techniques in ambient conditions (see Experimental Methods for more details). The measurements show the presence of high-conductance (HC) and low-conductance (LC) plateaus. Concentrating first on the HC plateau, the covalently bonded molecules show a most probable conductance above 1 × 10^–3^*G*_0_ (Table 1), where *G*_0_ = 2e^2^/*h* ≈ 77.5 μS is the conductance quantum, while the −SMe terminated compounds show around half an order of magnitude lower conductance than their −SAc counterpart (Table 1). This decrease is expected due to the weaker electronic coupling to the gold electrodes of −SMe compared to −SAc.^22^ For the −SAc anchored set of molecules, the conductance follows the trend **2a** ≈ **1a** < **3a** as shown in Figure 3a,b, top left panel. The HC plateaus determined for −SMe terminated compounds are within the margin of error from each other: **1b** ≈ **2b** ≈ **3b** (Table 1, and Figure 3a,b bottom left panel). Notably, a first analysis of the raw histograms did not show a high-conductance plateau for **2a** in both MCBJ and STMBJ measurements. This might be due to the low percentage of HC traces and the effect of the slanted LC traces. However, clustering allows isolating a conductance class with a HC plateau in the measurements of **2a** (Supporting Information Figures S2.2, S2.3, S3.3).

**Figure 3:**
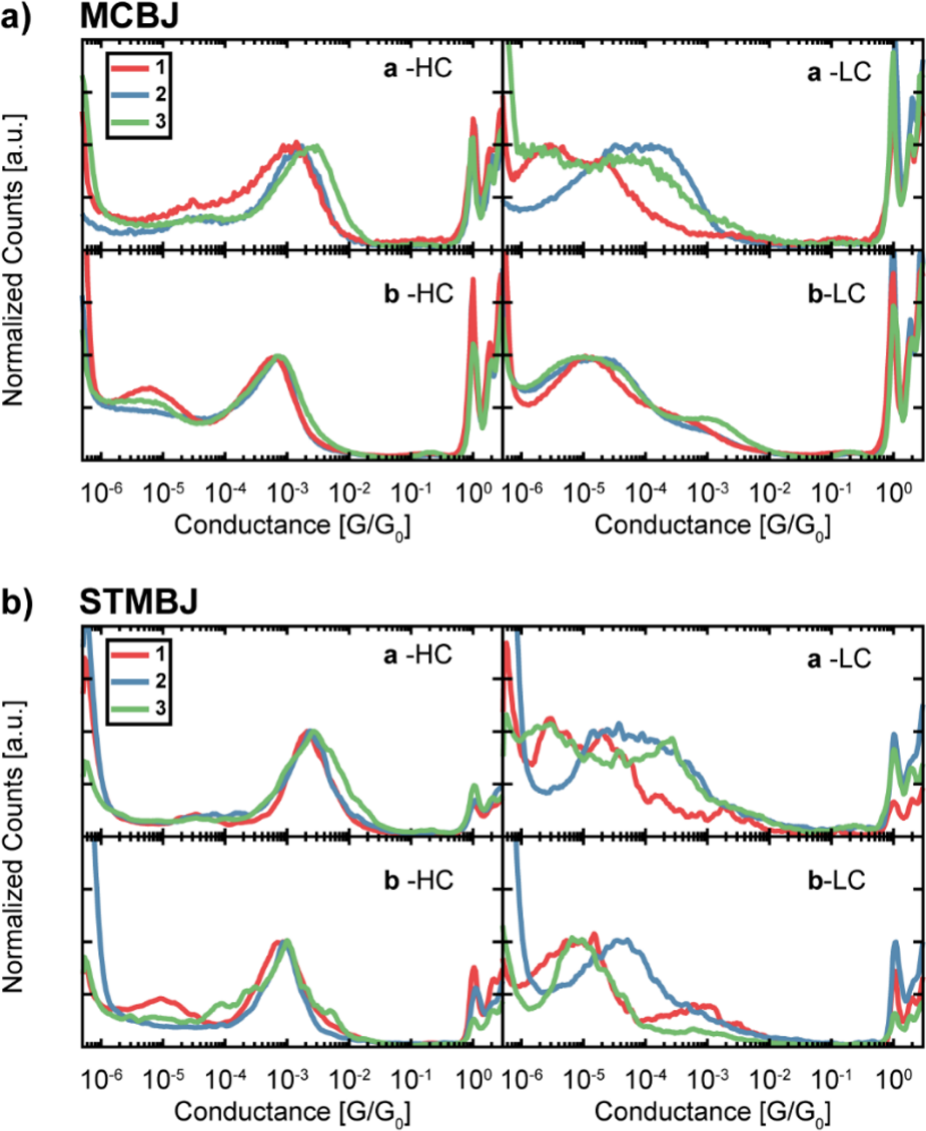
Measured conductance. 1D-conductance histograms for (a) MCBJ and (b) STMBJ measurements of the HC (left) and LC (right) peaks as obtained from clustering, separated by anchoring group (top: −SAc, bottom: −SMe; see Supporting Information for more information). The measurements have been normalized by the peak height to make the peak position and shape directly comparable across the different molecules.

**Table 1:**
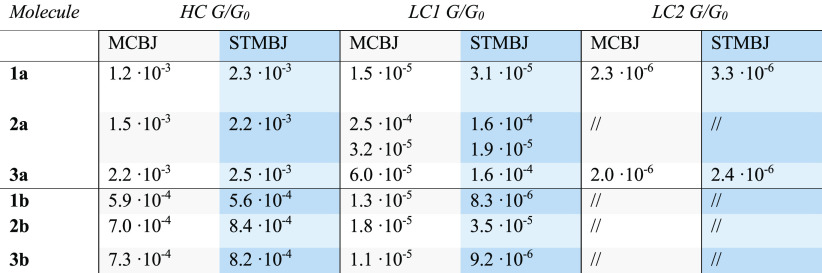
Average of the Most Probable Conductance Values Across All the Measurements for Each Molecule, in Units of *G*_0_ ≈ 77.5 μS, for Both MCBJ and STMBJ Experiments^a^

a The columns correspond to the values extracted by fitting with a log-normal distribution of the high-conductance (HC) class, and the two low conductance classes (LC1 and LC2). The conductance values of each measurement can be found in Supporting Information sections 2.1 and 3.1.

Focusing on the −SAc compounds, the LC slanted features follow the trend **2a** > **3a** > **1a**. Further analysis reveals the presence of two types of low-conductance features for −SAc compounds: slanted plateaus falling in the 10^–5^*G*_0_ range (LC1), which were measured for all compounds, and long, flat plateaus in the 10^–6^*G*_0_ range (LC2), which were measured only for **1a** and **3a** (Figures S2.1, S2.4, S2.5, S.3.2, S3.4). For **2a**, the slanted features can be separated into two groups: one in the 10^–5^*G*_0_ range (LC1^L^) very similar to what is observed as LC1 for the other compounds, and the other in the 10^–4^*G*_0_ range (LC1^H^) that shows shorter traces (Figures S2.2, S2.3, S3.3). Tolanes terminated with −SMe show a LC1 low-conductance feature, with very similar conductance among each other during MCBJ measurement, while, for STMBJ measurements, **2b** has half an order of magnitude higher conductance than **1b** and **3b** (Table 1, Figure 3a,b bottom left).

To further elucidate the relationship between the data obtained by the MCBJ and STMBJ measurements and the structural modifications introduced by the bottom up synthesis, we employed density functional theory (DFT) combined with the quantum transport calculations.^23^ To include contacting modalities in the theoretical analysis, several molecule electrode conformations were investigated. This includes junctions in which the anchor is connected to the electrodes via one, two or three gold atoms as shown in Figure 4a. For this, the material-specific mean-field Hamiltonians were obtained from the optimized geometry of the junctions using DFT.^24^ The resulting Hamiltonians were then combined with the quantum transport code GOLLUM^23,25^ to calculate the transmission coefficient *T*(*E*) for electrons passing from one electrode to the other through molecules **1**–**3**. *T*(*E*) was combined with the Landauer formula to obtain the electrical conductance (see Computational Methods for details).

**Figure 4:**
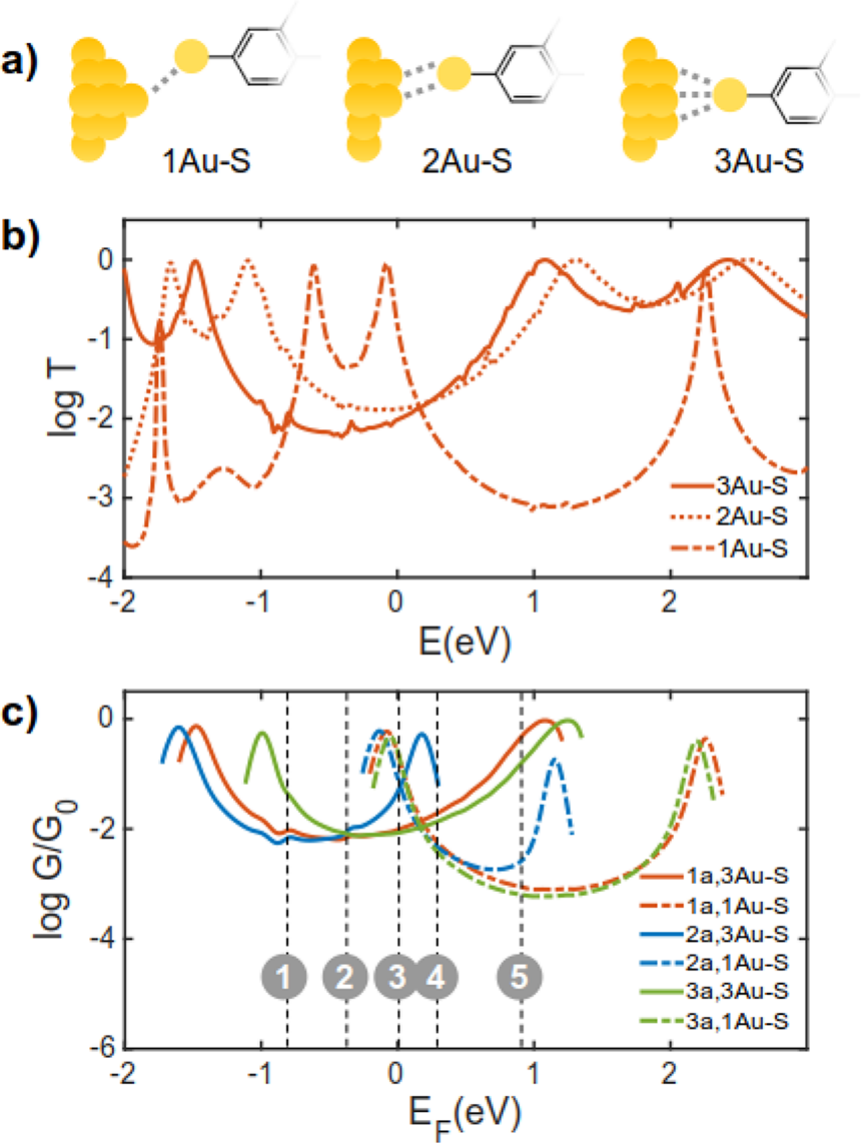
The effect of contacting modalities to the electrode. (a) Molecule/gold contact through one, two, and three gold atoms, (b) example of the transmission coefficient for molecule **1a** with different contacting conformations to the electrodes, (c) example of the conductance for molecule **1a**, **2a**, and **3a** with different contacting conformations to the electrodes. For clarity, *G* is shown for the *E*_F_ between HOMO and LUMO resonances only. *E* = 0 eV shows DFT Fermi energy. The gray dashed lines (marked by 1–5) show examples of the choice of Fermi energies. The conductance trend strongly depends on the choice of Fermi energy. To avoid this uncertainty in the prediction of the conductance trend, we propose a new modeling method based on computed conductance histograms.

Figure 4b shows an example of *T*(*E*) that was obtained for molecule **1a**, which is connected to the electrodes using three different contacting modalities (Figure 4a). It becomes clear that *T*(*E*) (and therefore conductance) is significantly influenced by the contacting modalities. Figure 4c shows *G* of **1a**, **2a**, and **3a**, which are connected to the electrodes via one or three gold atoms. *G* is significantly dependent on the conformation and choice of *E*_F_. For example, for junctions formed by connecting the anchor to three gold atoms (3Au–S), *G* of **3a** is highest when *E*_F_ is near the energies marked by 1, while it is lowest around energies marked by 3 in Figure 4c. This trend changes when the contacting modalities to the electrode change. For example, for junctions formed by connecting the anchor to one gold atom (1Au–S), *G* follows the order 3a ≈ 1*a* > 2a around *E*_F_ marked by 3 in Figure 4c. Such variations make prediction of electrical conductance a difficult task.^11,26−28^

To minimize this ambiguity, we propose a new method for analyzing the data obtained from the quantum transport calculations in order to predict conductance trends between different molecules. Most importantly, with the proposed method we were able to accurately predict the relatively small conductance enhancement (Figure 5) caused by nitro and dimethyl amino functionalities (Table 1). First, we form a series of junctions with different contacting modalities to electrodes and calculate the electrical conductance *G* for a range of electrodes Fermi energies. Next, we create the conductance histograms using the calculated conductance for each junction and for a wide range of *E*_F_ between the HOMO–LUMO gap. The peaks in the conductance histograms are fitted with a log-normal distribution and their center is defined as the most probable computed conductance. Such data analysis is relevant because the transport usually takes place off resonance and the energetic position of the frontier orbitals relative to *E*_F_ of the electrodes is expected to vary for each trace in break junction experiments. The former is due to the different electrode surfaces formed in each break junction experiment.

**Figure 5:**
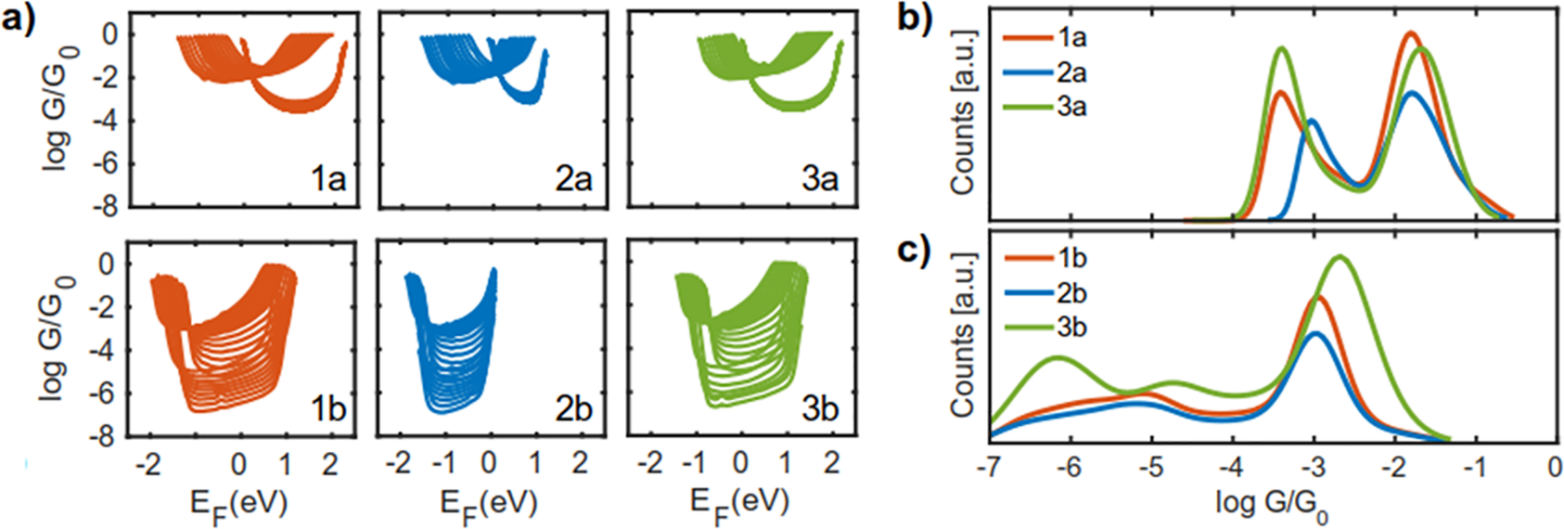
Calculated conductance histograms. (a) Conductance for molecule **1**, **2**, and **3** with different anchor groups and contacting conformation to electrodes; conductance histograms based on conductances in (a) for a wide range of Fermi energies between the HOMO–LUMO gap for **1a**, **2a**, and **3a** (b) and for **1b**, **2b**, and **3b** (c).

Figure 5a shows the conductance of molecules **1**–**3** with different anchor groups connected to electrodes through different conformations. These configurations are obtained by moving the electrodes away from the molecule and increasing the molecule–electrode distance resembling break junction experiments. This process has been repeated for all molecules and with three different Au–S configurations shown in Figure 4a. For clarity, Figure 5a shows the conductance curves between HOMO and LUMO resonances. We find that, first, the amplitude of conductance varies significantly by conformation to electrodes. Second, the position of resonances due to the frontier orbitals changes with respect to the electrodes’ Fermi energy *E*_F_. This is because of the changes in charge transfer between the molecule and electrode by contacting modalities to electrodes (e.g., Au–S contact through one, two, or three gold atoms as shown in Figure 4a) and the distance between the molecule and electrode. These effects lead to a large variation on the electrical conductance for any given *E*_F_. This ambiguity is lower for junctions with large differences in *G* for a wide range of *E*_F_, but otherwise higher.

Figures 5b,c shows the constructed conductance histograms for molecules **1**–**3** with −SAc and −SMe anchor groups, respectively, using the calculated conductance for a wide range of *E*_F_ within the HOMO–LUMO gap of molecules and for the different contacting conformations to electrodes. In agreement with the measured conductance values, the electrical conductance of the molecules with −SAc anchors is generally higher than that of molecules with −SMe anchors. Furthermore, there are at least two peaks in the calculated conductance histograms, similar to that obtained in the experimental results in Figure 3. We attribute the higher peak to those junctions formed by the anchor connected to the electrodes through more than one gold atom. The low conductance group is attributed to molecules contacting the electrodes via a single gold atom. We note that the computed conductance histograms show higher conductance values compared to the measured values. This is because DFT usually understates the HOMO–LUMO gap leading to higher electrical conductance in calculations.

Larger variations in the conductance values due to the side groups are predicted for the low conductance group (Figure 5b,c), which is in agreement with the experimental results (Figure 3). It is worth mentioning that conductances resulting from the interaction of two molecules in the junction, e.g., π–π, stacking is not included in the histograms of Figure 5. As shown in Figure S4.3 of the Supporting Information, π–π stacking could lead to a third conductance group with lower conductance. Furthermore, we examined the effect of asymmetric binding configurations to electrodes. While the computed conductance histogram of **1a** changes with additional asymmetric configurations, it retains the main features (see Figure S4.5 in the Supporting Information). Our result shows that the conductance trend for both high and low conductance groups obtained from the calculated histograms is in good agreement with the experiments for all molecules. This is important because *E*_F_ is no longer a free parameter in the statistical method proposed here to construct theoretical conductance histograms and cannot be chosen arbitrarily. The prediction is also conclusive for a series of molecules with even small differences in their *G* values. This is a general method and can be used for analysis of the conductance of any junction, theoretically.

It is worth mentioning that by introducing NO_2_ side groups, a new energy level (Figures 2b and S4.4 of the Supporting Information) and transport channel (see Figure S4.4 of the Supporting Information) is formed between the HOMO–LUMO gap of the parent tolane molecule. This new resonance leads to a higher *G* in **2a** and **2b** compared to **1a** and **1b**, respectively, which is more pronounced in the low conductance group (Figures 3 and 5). The NO_2_ substituents are also expected to change the sign of the Seebeck coefficient. To test this hypothesis, Seebeck coefficient measurements were carried out using the STMBJ technique (see Experimental Methods). All of the Seebeck coefficient vs conductance histograms (Supporting Information Figure S3.9) show a distribution similar to the corresponding conductance histograms with all of the classes in Figure S3.1, suggesting the presence of the same conductance clusters in all measurements. Therefore, the traces are classified the same way, obtaining a different Seebeck coefficient for each class, depicted in Table 2. For the LC2 classes, it is not possible to determine the Seebeck coefficient since the conductance values are outside of the measured conductance range.

**Table 2:**
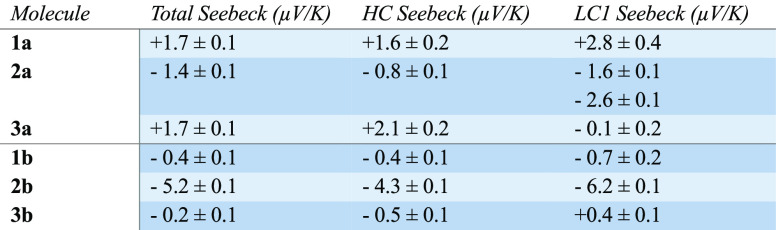
Average Seebeck Value ± the Standard Deviation in All of the Thermopower Measurements for Each Molecule^a^

a The columns correspond to the Seebeck values obtained by fitting the temperature difference dependence of the thermovoltage measurements with a linear regression (see Experimental Methods), for all of the measurements and the high-conductance HC and the low conductance LC1 clusters of each compound. The Seebeck coefficient vs conductance histograms can be found in Supporting Information section 3.2.

The results show in general more positive values for −SAc terminated compounds than for −SMe terminated compounds. −SMe terminated compounds are found to be mainly negative. In detail, compounds **1a** and **3a** show a positive Seebeck coefficient (1.7 μV/K for both), while **1b** and **3b** are negative and closer to zero (−0.4 μV/K and −0.2 μV/K respectively). Interestingly, compound **2** shows a more negative Seebeck coefficient than **1** and **3**. The measurements reveal a negative Seebeck coefficient instead of a positive one for **2a** (−1.4 μV/K) and an even more negative one for **2b** (−5.2 μV/K). Our Seebeck coefficient calculations (Figure S4.6 in the Supporting Information) are in agreement with these results. To show this effect of shifting to negative values of the Seebeck coefficient by NO_2_ side groups, Figure S3.10 depicts the linear regressions of the thermovoltage measurements performed at several temperature differences for all the compounds, where the slope of the regression is the total Seebeck value in Table 2. This supports the predictions that the NO_2_ side groups create additional charge transport channels and can therefore be used to tune charge transport properties of molecular junctions and devices.

Our calculations and experiments demonstrate that the electron-donating and -withdrawing side groups can be used to tune the conductance of tolane derivatives. These side groups modify the position of frontier orbitals by either changing electron charges on the molecular backbone or introducing additional resonance within the HOMO–LUMO gap of the backbone molecule. The changes in conductance obtained by FOE are larger for the low conductance groups. Using computational simulation of junctions with several electrode–molecule conformations and a new data analysis method, we attribute the low conductance groups to fully extended junctions where anchors are only connected to a single gold atom. While the changes in the energy levels due to FOE are large for molecules in the gas phase (shown by our CV measurement), the improvement in conductance is relatively small. This is due to the strong dependence of conductance on the Fermi energy, binding modalities to electrodes, and charge transfer between molecules and electrodes. The latter modulates the effect of electron-donating or -withdrawing groups and might be prevented by weakening the coupling between the molecular core and the electrodes.

In summary, we studied the effect of electron-donating and -withdrawing side groups on the conductance and Seebeck coefficient of molecular junctions formed by tolane derivatives. We found that (1) the measured conductance values and different conductance groups are in good agreement between two different break junction measurement methods (STMBJ and MCBJ) used; (2) the theoretical data analysis model proposed shows good agreement with the experiment. This suggests that the new method can be used to more accurately predict the electrical conductance of molecular junctions and minimize ambiguity due to uncertainty of the position of Fermi energy of electrodes relative to the energy levels of molecule; (3) the electron-donating and -withdrawing side groups can be used to tune the conductance of fully extended molecular junctions; (4) Most interestingly, the addition of nitro groups introduces additional resonances that lead to a negative shift of the Seebeck coefficient, while dimethylamino groups seem to have no substantial effect on it. While our result provides proof of the concept of FOE in OPE2 molecular junctions, further studies are needed to increase the changes in electrical conductance using other combinations of molecular backbones and electron-donating and -withdrawing side groups.

## Methods

### Computational Methods

. The optimized geometries with ground-state Hamiltonian and overlap matrix elements for gas phase molecules and molecules between electrodes were obtained using DFT. These results were then combined with the Green’s function method to calculate the phase-coherent, elastic-scattering properties of the system, consisting of two gold electrodes and the molecule as the scattering region. The details of the computational methods are provided in the Supporting Information.

### Molecular Synthesis

**Scheme 1:**
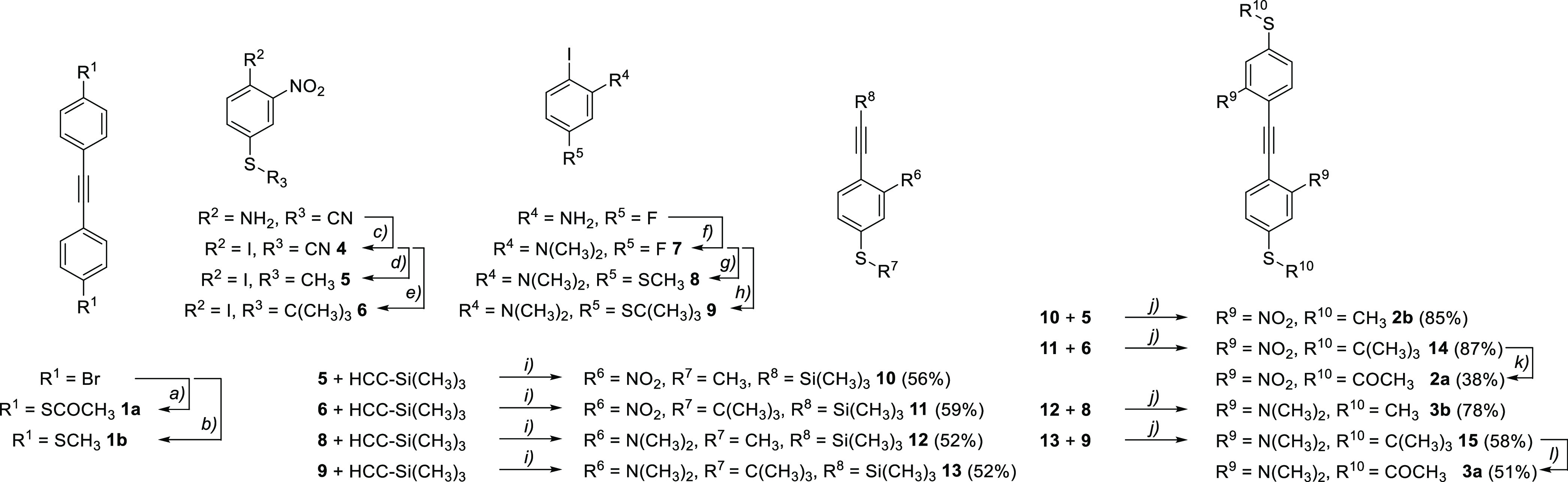
Synthesis of the Model Compounds **1**–**3 a-b** Reagents and Conditions: *a)* Xantphos, Pd_2_(dba)_3_, NEt_3_, KSCOCH_3_, toluene, 100 °C, 15 h, 57%; *b)* Xantphos, Pd_2_(dba)_3_, NEt_3_, NaSCH_3_, toluene, 100 °C, 18 h, 62%; *c)* NaNO_2_, KI, *p*-TsOH, CH_3_CN, 0 °C – rt., 65%; *d)* 1.) KOH, CH_3_OH, rt., 2 h, 2.) CH_3_I, 2 h, rt., quant.; *e)* 1.) KOH, CH_3_OH, rt., 2 h, 2.) AlCl_3_, ClC(CH_3_)_3_, 4 h, rt., 86%; *f)* K_2_CO_3_, CH_3_I, DMF, 45 °C, 24 h, 86%; *g)* Cs_2_CO_3_, NaSCH_3_, DMAc, 110 °C, 16 h, 73%; *h)* Cs_2_CO_3_, KSC(CH_3_)_3_, DMAc, 110 °C, 32 h, 44%; *i)* CuI, Pd(PPh_3_)_2_Cl_2_, THF, NEt_3_, 14-17 h; *j)* K_2_CO_3_, CuI, Pd(PPh_3_)_4_, THF, CH_3_OH, NEt_3_, 16 h; *k)* Bi(OTf)_3_, ClCOCH_3_, CH_2_Cl_2_, 38%; l) BBr_3_, ClCOCH_3_, CH_2_Cl_2_, 51%.

. The assembly of the model compounds is based on different synthetic strategies and displayed in Scheme 1 (synthetic protocols are provided in the Supporting Information). The parent tolanes **1a** and **1b** were both obtained in a single step by an already reported Pd-catalyzed substitutions^29^ of both terminal bromine substituents of 1,2-bis(4-bromophenyl)ethyne using either potassium thioacetate to obtain **1a**, or sodium methanthiolate for **1b**, respectively. Both tolanes, **1a** and **1b** were already described in the literature although obtained by different approaches.^30,31^

In the case of the nitro- and dimethylamine decorated tolanes **2a**, **2b**, **3a**, and **3b**, the corresponding 4-iodophenyl-alkylsulfanes were synthesized first. The nitro iodophenyles **5** and **6** were prepared from commercially available 2-nitro-4-thiocyanoaniline by a Sandmeyer reaction protocol previously described in the literature^32^ followed by transprotection of the thiol toward either tertbutyl thiol or methyl thiol with AlCl_3_ and ClC(CH_3_)_3_ or MeI respectively, while the dimethylamino iodophenylenes **8** and **9** were prepared from the commercially available 5-fluoro-2-iodoaniline by methylation with methyl iodide and subsequent nucleophilic aromatic substitution on the fluorine with the desired thiol moiety. With all four 4-iodophenyl-alkylsulfanes **5**, **6**, **8**, and **9** in hand, the corresponding tolanes were assembled by two subsequent Sonogashira–Hagihara coupling reactions introducing the interconnecting acetylenes. To enable the conditions of transformations and coupling reactions, the terminal thiol anchor groups were masked as *tert*-butyl sulfide and were converted in the last step to the acetyl sulfide of **2a** and **3a**. For the nitro derivative, this was performed using bismuth(III) triflate, while for the dimethyl amino derivative with BBr_3_/AcCl in DCM was used as bismuth triflate could coordinate to the two amines resulting in a lower yield or no conversion at all as described by Nielsen.^33^

### Experimental Methods

. MCBJ experiments consist of stretching a lithographically defined gold nanowire until atomic sized electrodes are formed. Then, these electrodes are repeatedly opened and closed, while the current flowing through them is recorded using a constant bias of 0.1 V. The measured current versus the applied electrode displacement comprises the so-called conductance-displacement trace. More information about this technique can be found in previous reports.^34^ When a measurement is performed, first the device is characterized without any molecules. Afterward, around 5 μL of a 0.1 mM solution in dichloromethane of the desired molecule are dropcast on the junction, and the measurement is started. The set of traces collected in a measurement is used to build two-dimensional (2D-) conductance/displacement and one-dimensional (1D-) conductance histograms. Peaks in the latter are fitted with a log-normal distribution, whose center defines the most probable conductance (or in short, the “conductance”) of the molecular junction. To better highlight the presence of different types of features in each measurement, the data were classified using a two-step approach. First, a supervised learning algorithm was used to split traces that contain molecular features from the ones that only show clean exponential decay. Subsequently, an unsupervised machine learning algorithm^35^ has been used to separate the molecular traces in different classes. The raw-data 2D- and 1D-histograms, as well as all those obtained through the described clustering analysis can be found in Supporting Information section 2.1.

STMBJ measurements were taken by recording the current while consecutively approaching and pulling the tip, a mechanically cut 0.25 mm diameter gold wire (Goodfellow), to the sample, a preannealed gold surface (Arrandee) on which the molecules were deposited from a 1 mM solution in DCM, at a constant bias of 0.1 V. The resultant conductance-displacement traces are used to build two-dimensional (2D-) conductance/displacement and one-dimensional (1D-) conductance histograms. Traces with molecular feature are selected and classified into the different classes by the use of the k-means algorithm. For the thermopower measurements, a 1 kΩ resistance on the tip is used to heat it, creating the temperature difference between the electrodes, keeping the sample at room temperature. Several temperature differences are applied, while 10 mV bias voltage ramps are performed when a molecular junction is detected (the conductance points measured are within a defined range). The conductance and the thermovoltage are determined simultaneously as the slope and the voltage offset of the linearly fitted IV-curves. Due to the temperature difference, there is an additional term in the current, that is observed as an offset in voltage of the IV curves (Supporting Information Figure S3.11). The conductance and the thermovoltage are determined simultaneously as the slope and the voltage offset for every linearly fitted IV curve. These values give rise to the points in the histograms in Figure S3.9. The temperature dependence of the thermovoltage values is linearly fitted, so the slope is the mean Seebeck value.

## Notes

The input files to reproduce simulation data and underlying experimental data can be accessed by contacting the authors.

## Supporting Information Available

Synthesis details, a complete set of measurement data obtained using the MCBJ and STMBJ methods, as well as details on the data analysis method, theory and modeling methods, and supporting figures (PDF)
