# Supplementary material for: Engineering Transport Orbitals in Single-Molecule Junctions
Source: J Phys Chem Lett. 2022 Sep 27;13(39):9156–64. doi: 10.1021/acs.jpclett.2c01851 (PMC9549519; doi:10.1021/acs.jpclett.2c01851)
Supplement: Supplementary file 1 — jz2c01851_si_001.pdf [file jz2c01851_si_001.pdf]

Supporting information

Engineering Transport Orbitals in Single Molecule Junctions

Abdalghani Daaoub[1,+], Luca Ornago[2,+], David Vogel[3,+], Pablo Bastante[4,+], Sara Sangtarash[1],Matteo Parmeggiani[5], Jerry Kamer[2], Nicolás Agraït[4,*], Marcel Mayor[3,6,7,*], Herre van der Zant[2,*], Hatef Sadeghi[1,*]

[1] School of Engineering, University of Warwick, CV4 7AL Coventry, United Kingdom
[2] Kavli Institute of Nanoscience, Delft University of Technology, Lorentzweg 1, 2628 GJ Delft, The Netherlands
[3] Department of Chemistry, University of Basel, St. Johanns-Ring 19, 4056 Basel, Switzerland
[4] Departamento de Física de la Materia Condensada, Universidad Autónoma de Madrid, E-28049 Madrid, Spain
[5] Department of Applied Science and Technology (DISAT), Politecnico di Torino, Corso Duca degli Abruzzi 24, 10129 Torino, Italy
[6] Institute for Nanotechnology, Karlsruhe Institute of Technology (KIT), P. O. Box 3640, 76021 Karlsruhe, Germany
[7] Lehn Institute of Functional Materials (LIFM), School of Chemistry, Sun Yat-Sen University (SYSU), 510275 Guangzhou, China

+ Authors with equal contributions.
**Hatef.Sadeghi@warwick.ac.uk; h.s.j.vanderzant@tudelft.nl; marcel.mayor@unibas.ch; nicolas.agrait@uam.es*

Table of content

1 Synthesis 1

2 MCBJ measurements and data analysis 85

3 STMBJ measurements and data 101

4 Theory and modelling 107

Supporting References 112

1 Synthesis

All chemicals and anhydrous solvents were used as purchased without further purification, unless stated otherwise. Deuterated solvents were obtained from Cambridge Isotope Laboratories, Inc. (Andover, MA, USA). All other commercial available starting materials were purchased from Sigma-Aldrich, Acros or Fluorochem. NMR experiments were acquired on a 400 or 500 MHz Bruker Avance III spectrometer equipped with a QNP or BBFO probe head respectively. The chemical shifts (δ) are reported in parts per million (ppm) relative to tetramethylsilane or referenced to residual solvent peaks and the J values are given in Hz (± 0.1 Hz). For high-resolution mass spectrometry (HRMS) a HR-ESI-ToF-MS measurement on a maXisTM 4G instrument from Bruker was performed. Gas chromatography-mass spectrometry (GC-MS) were recorded using a Shimadzu GC-MS-2020- SE instrument equipped with a Zebron 5 MS Inferno column, with a temperature range of up to 350 °C. Column chromatography was performed on SiliaFlash®P60 from SILICYCLE with a particle size of 40-63 µm (230-400 mesh). Thin layer chromatography (TLC) was performed on Silica gel 60 F254 glass plates with a thickness of 0.25 mm from Merck using fluorescent quenching under UV light at 254 nm for the localization of sample spots. Automated recycling GPC was performed on a Shimadzu Prominence System equipped with two SDV preparative columns in series from Polymer Standards Service (PSS) (20 × 600 mm each, exclusion limit: 30,000 g/mol). UV–vis absorption spectra were recorded at 20 °C on a Jasco V-770 spectrophotometer. UV-Vis emission spectra were recorded at 20 °C on a JASCO Spectrofluorometer FP-8600. Cyclic voltammetry was performed in an MBraun Glovebox under argon. As working electrode, a glassy carbon disk electrode was used while as counter and (pseudo) reference electrode a silver wire was used. The voltage was applied and controlled with a Versastat 3-200 potentiostat from Princeton Applied Research. IR spectra were recorded with a Shimadzu IRTracer-100.

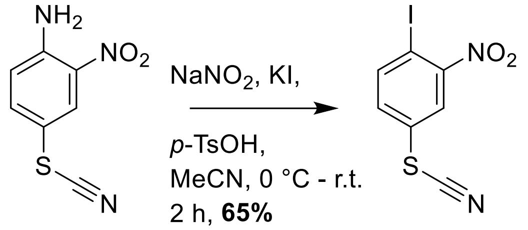

4

1-iodo-2-nitro-4-thiocyanatobenzene 4: An oven dried argon flushed two necked round bottom flask was charged with 2-nitro-4-thiocyanatoaniline (500 mg, 2.56 mmol, 1 eq.) and *p*-TsOH (1.460 g, 7.68 mmol, 3 eq.) suspended in MeCN (10 mL). The mixture was cooled to 0 °C in an ice bath. Then a solution of $NaNO_2$ (353 mg, 5.12 mmol, 2 eq.) and KI (1.06 g, 6.40 mmol, 2.5 eq.) in Milli-Q water (1.8 mL) was gradually added. The mixture was stirred at 0 °C for 10 min and left to warm up to r.t. over 2 h. The reaction was then poured into aq. sat. $NaHCO_3$, extracted with EtOAc. The combined organic phase was washed with aq. sat. $Na_2S_2O_3$, water, brine, dried over anhydrous $MgSO_4$, filtered, concentrated under reduced pressure and purified by column chromatography on silica gel (cyclohexane : EtOAc gradient from 100 : 0 to 50 : 50) yielding a yellow solid (510 mg, 1.67 mmol, 65%).

^{1}H-NMR (500 MHz, $CDCl_3$) δ 8.13 (d, J = 8.4 Hz, 1H), 7.98 (d, J = 2.3 Hz, 1H), 7.43 (dd, J = 8.4, 2.3 Hz, 1H).

^{13}C-NMR (126 MHz, $CDCl_3$) δ 153.57(extracted from HMBC), 143.77, 133.42, 127.28, 125.78, 108.10, 87.56.

IR *ν*(cm^{-1}): 3085.37, 2158.65, 1579.57, 1545.82, 1517.85, 1455.65, 1344.27, 1274.84, 1255.56, 1344.27, 1274.84, 1255.56, 1161.53, 1102.71, 1020.71, 964.33, 877.54, 823.54, 755.07, 742.53, 700.10, 655.26, 593.54, 481.68, 449.86

GC-MS (EI): *m/z* (%) = 305.6 (100), 259.7(22.6), 148.9 (17.8), 132.9 (89.8), 120.9 (21.18), 74.9 (35.4), 63.0 (36.5)

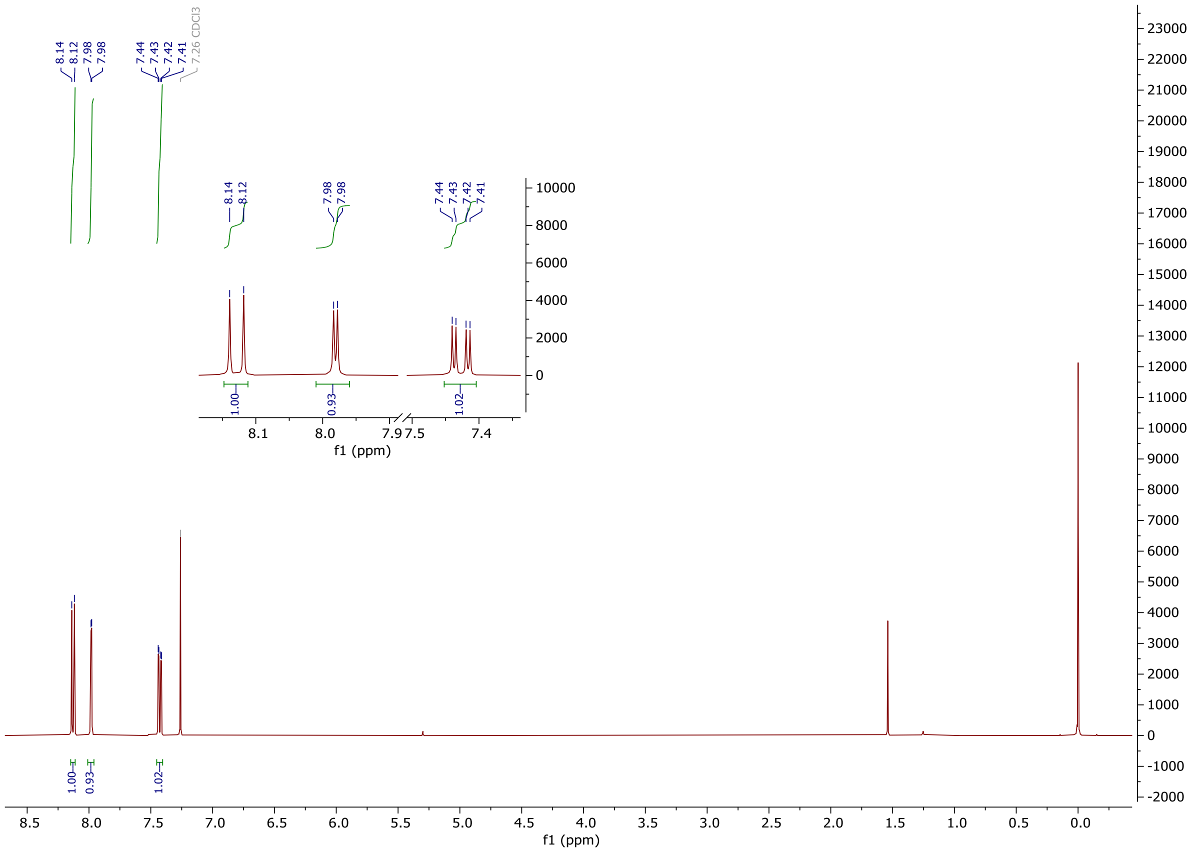

Figure S1.1: ^{1}H-NMR (500 MHz, $CDCl_3$) spectrum of 4.

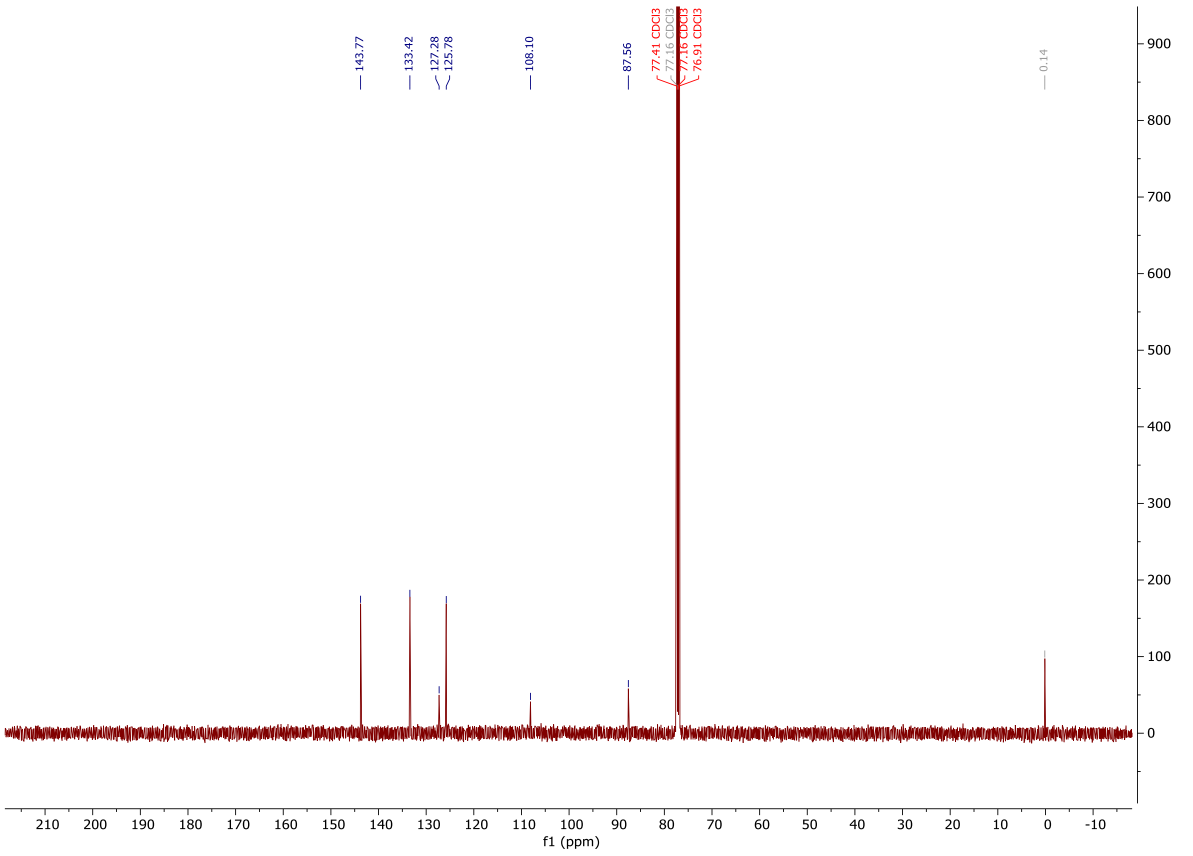

Figure S1.2: ^{13}C-NMR (126 MHz, $CDCl_3$) spectrum of 4.

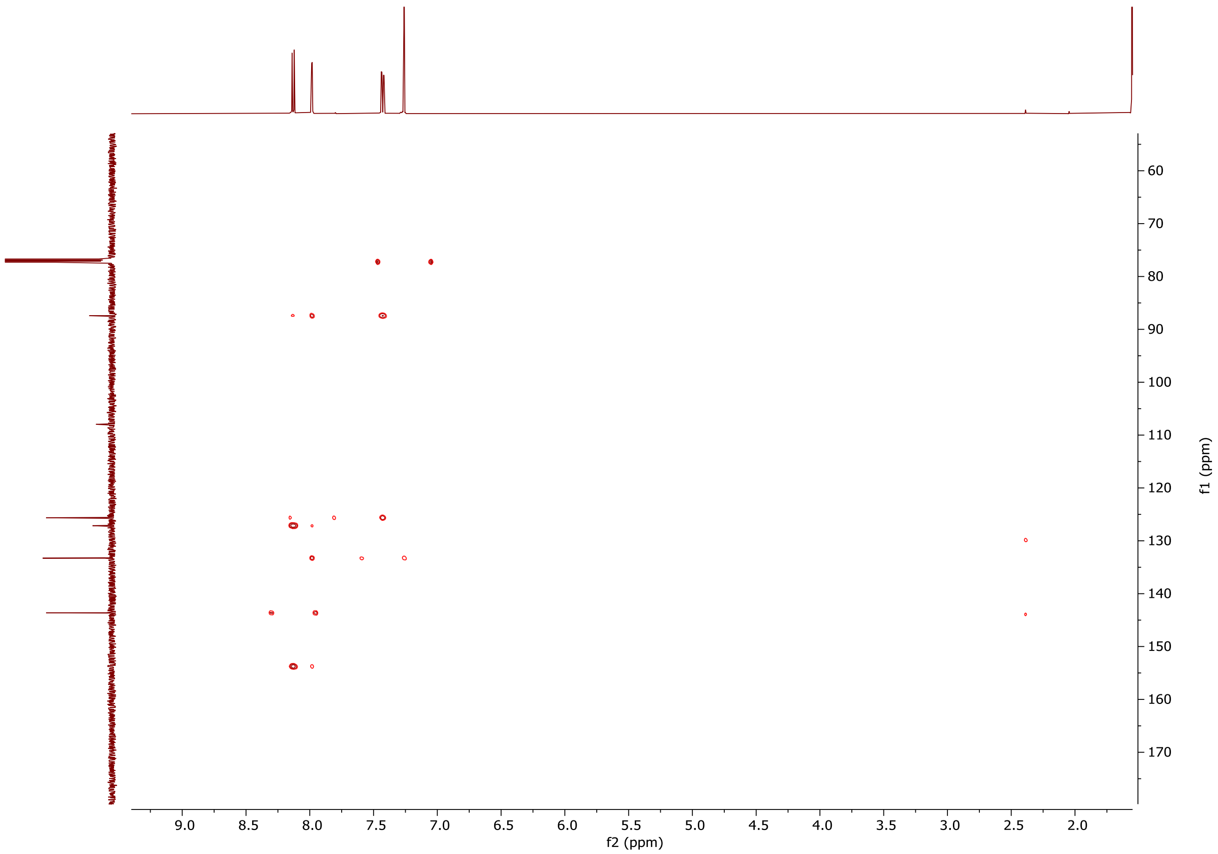

Figure S1.3: HMBC_GPSW ($CDCl_3$) spectrum of 4.

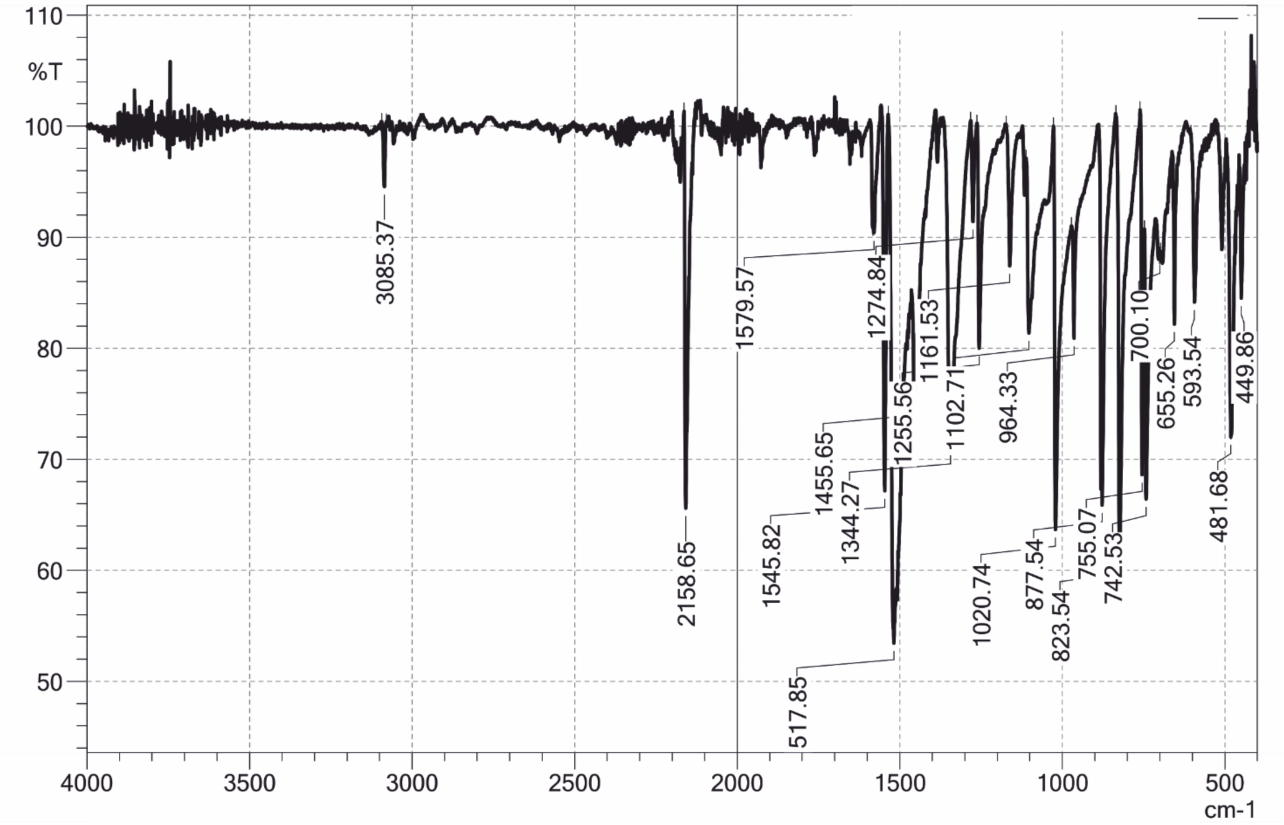

	Item	Value
2	Sample name	VOE_372
3	Sample ID	VOE_372
4	Option	
5	Intensity Mode	%Transmittance
6	Apodization	Happ-Genzel
9	No. of Scans	20
10	Resolution	1 cm-1

	Peak	Intensity	Corr. Intensity	Base (H)	Base (L)	Area	Corr. Area	Comment
1	449.86	84.50	7.64	452.27	446.00	78.386	27.212	
2	481.68	72.00	2.72	491.33	480.72	165.694	0.238	
3	593.54	84.16	13.25	604.63	581.49	187.085	125.259	
4	655.26	82.16	11.78	657.19	649.48	80.681	35.971	
5	700.10	88.25	0.19	700.58	698.17	27.846	0.252	
6	742.53	66.42	24.34	747.35	716.98	554.958	275.631	
7	755.07	68.60	28.26	760.86	747.35	196.112	143.429	
8	823.54	61.22	34.00	835.59	806.18	551.911	373.227	
9	877.54	65.86	5.37	879.47	835.59	434.019	-202.975	
10	964.33	80.92	10.64	971.56	947.93	250.523	58.863	
11	1020.74	63.65	34.24	1027.01	989.88	711.138	479.732	
12	1102.71	81.37	13.94	1111.39	1079.57	408.611	210.914	
13	1161.53	87.42	12.07	1172.62	1143.69	154.618	133.947	
14	1255.56	80.01	17.07	1262.79	1243.98	205.900	132.990	
15	1274.84	91.40	9.01	1282.07	1262.79	58.463	65.728	
16	1344.27	71.07	29.11	1363.08	1282.07	1015.603	1039.053	
17	1455.65	75.45	2.40	1456.62	1449.39	160.349	8.599	
18	1517.85	53.44	1.77	1519.78	1516.41	154.253	2.904	
19	1545.82	67.16	33.82	1554.50	1535.69	263.856	282.507	
20	1579.57	90.37	0.82	1581.98	1577.16	44.492	2.015	
21	2158.65	65.60	18.45	2163.95	2155.27	185.942	74.762	
22	3085.37	94.54	5.85	3093.57	3079.58	28.401	33.847	

Figure S1.4: FT-IR spectra and peak table of 4.

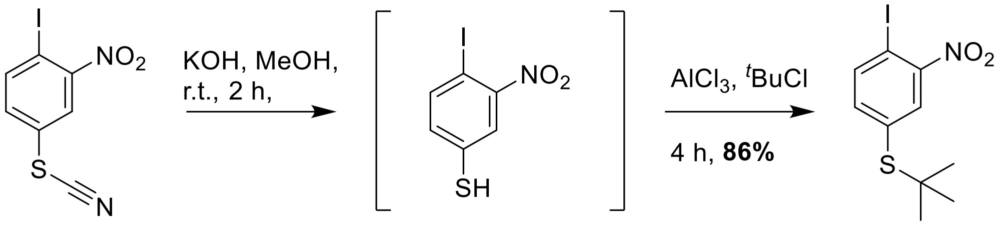

***tert*-butyl(4-iodo-3-nitrophenyl)sulfane 6**: An argon flushed oven dried two necked round bottom flask was charged with KOH (642 mg, 11.5 mmol, 5 eq.) and absolute ethanol (20 mL). The solution was sparged with argon. Thiocyanide 4 (700 mg, 2.29 mmol, 1 eq.) was added portion wise at 10 °C. The mixture was stirred for 30 min at r.t. and a degassed mixture of H_2SO_4 in EtOH (10% Vol.) was added cautiously. The mixture was then poured into water and extracted twice with EtOAc. The combined organic phase was dried over anhydrous $MgSO_4$, filtered and concentrated under reduced pressure. The crud intermediate 4a was directly used without further purification.

A round bottom flask equipped with a reflux condenser was charged with *tert*-butyl chloride (5 mL, 45.8 mmol, 20 eq.) and sparged with argon. Thiol 4a (644 mg, 2.29 mmol, 1 eq.) was added and the mixture was degassed. Then $AlCl_3$ (183 mg, 1.37 mmol, 0.6 eq.) was added portion wise (10 mg portions) and the mixture was stirred for 4 h at r.t. The reaction was quenched with water and extracted with DCM. The combined organic phase was dried over anhydrous $MgSO_4$, filtered, concentrated under reduced pressure and purified by column chromatography on silica gel (cyclohexane : EtOAc 8:1) and column chromatography on silica gel (cyclohexane : toluene 5:1) yielding 6 as a beige solid (661 mg, 1.96 mmol, 86%)

^{1}H-NMR (500 MHz, $CDCl_3$) δ 7.98 (d, J = 8.1 Hz, 1H), 7.96 (d, J = 2.1 Hz, 1H), 7.38 (dd, J = 8.1, 2.1 Hz, 1H), 1.32 (s, 9H).

^{13}C-NMR (126 MHz, $CDCl_3$) δ 152.99, 141.82, 141.66, 135.68, 133.24, 86.87, 47.53, 31.07.

HRMS (ESI) m/z: calcd. for $[C_{10}H_{12}INO_2S+Na]^+$ 359.9522 $[M+Na]^+$; found 359.9526

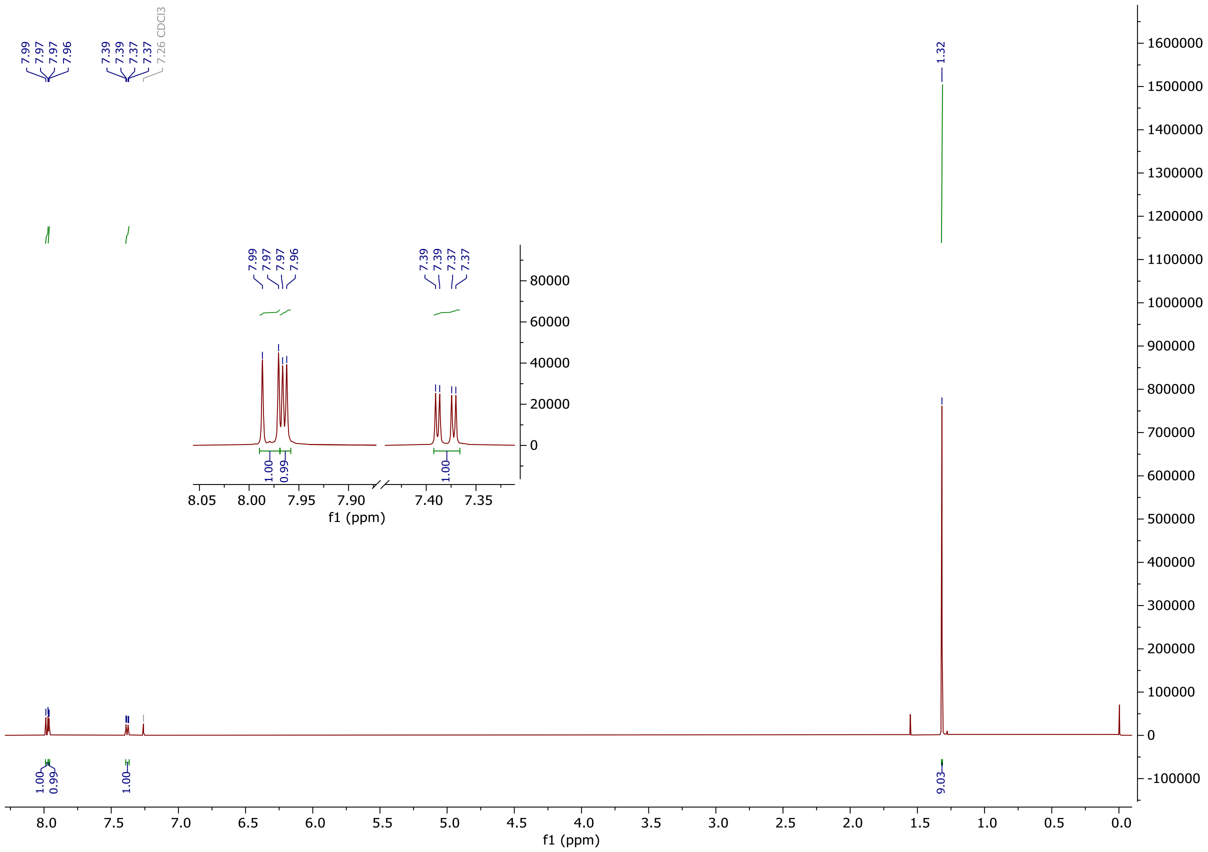

Figure S1.5: ^{1}H-NMR (500 MHz, $CDCl_3$) spectrum of 6.

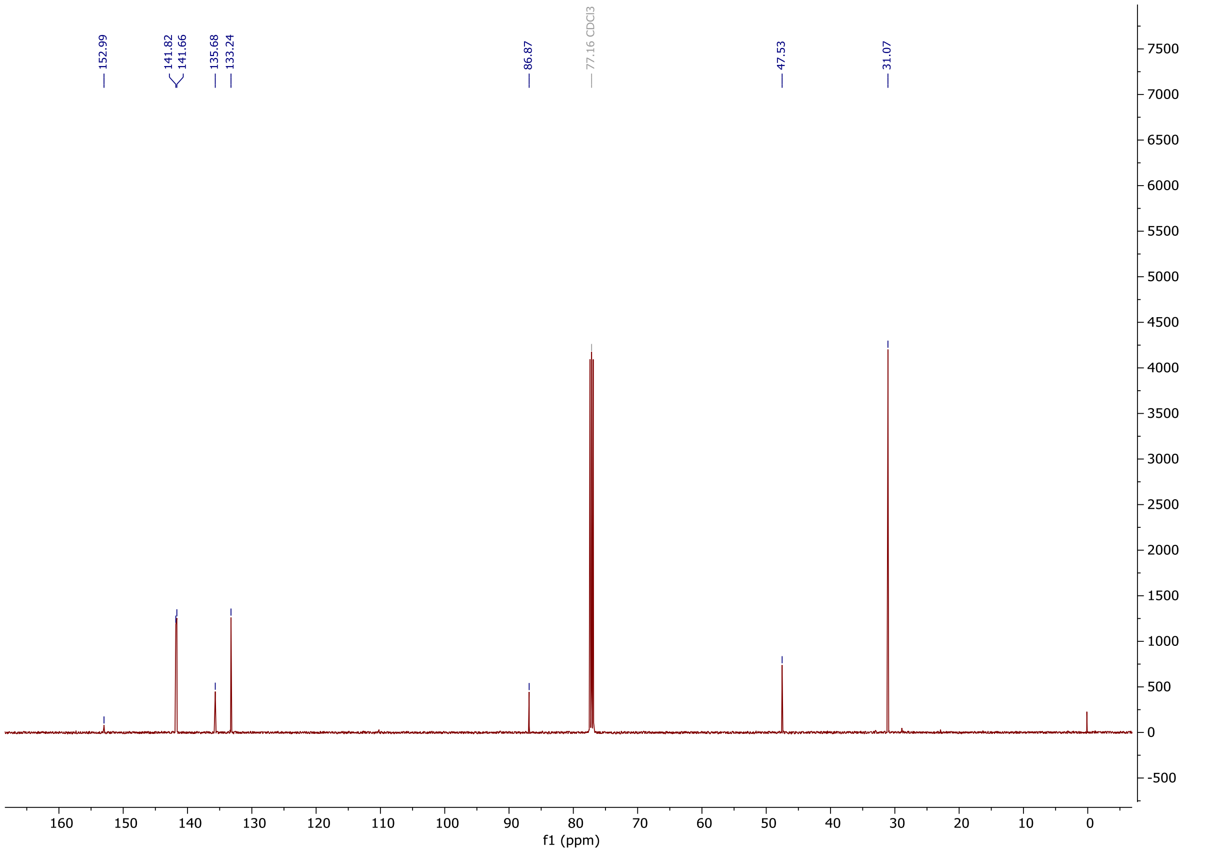

Figure S1.6: ^{13}C-NMR (126 MHz, $CDCl_3$) spectrum of 6.

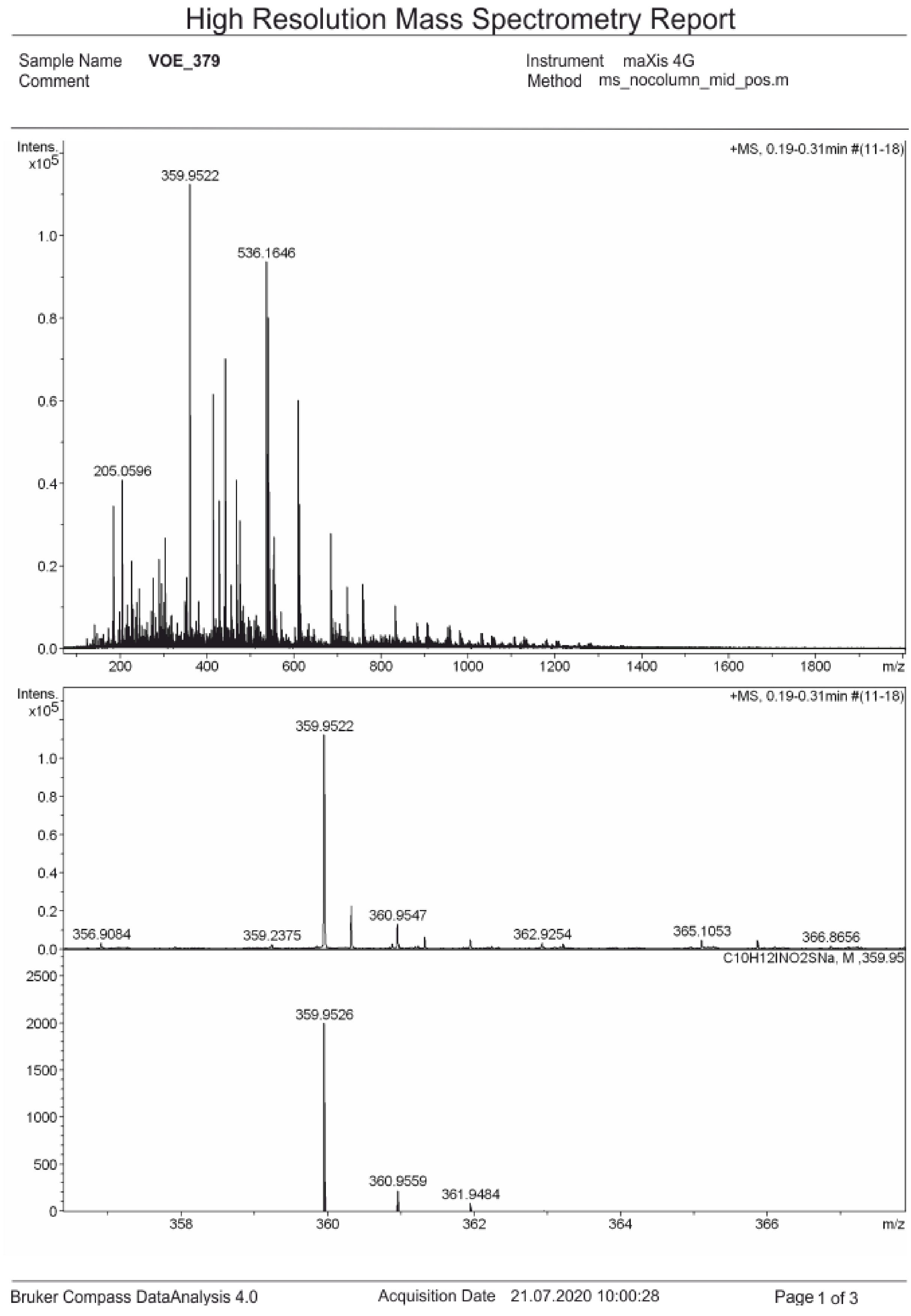

Figure S1.7: HRMS (ESI) spectrum of 6.

High Resolution Mass Spectrometry Report

Measured m/z vs. theoretical m/z

Meas. m/z	#	Formula	Score	m/z	err [mDa]	err [ppm]	mSigma	rdb	e⁻ Conf	z
359.9522	1	C 10 H 12 I N Na O 2 S	100.00	359.9526	0.4	1.1	4.3	4.5	even	1+

Mass list

#	m/z	I %	I
1	183.0775	6.1	6842
2	185.1144	30.9	34785
3	199.1300	8.1	9123
4	205.0596	36.4	41003
5	216.9789	6.5	7297
6	217.0467	8.8	9912
7	217.1042	9.5	10711
8	226.9510	19.0	21401
9	227.1250	6.2	7024
10	229.8924	8.6	9729
11	235.9096	6.7	7487
12	236.0712	7.0	7885
13	239.0883	10.2	11441
14	243.9410	6.4	7247
15	245.0778	13.2	14840
16	261.1303	5.8	6545
17	271.1872	8.2	9236
18	275.1613	15.3	17186
19	280.9401	7.0	7825
20	288.9214	19.4	21810
21	294.9193	14.2	16028
22	301.1401	10.2	11485
23	303.8968	24.1	27073
24	305.1708	5.8	6525
25	317.2443	7.2	8075
26	319.2598	7.3	8222
27	348.9893	10.3	11616
28	350.9865	9.4	10596
29	353.1446	13.2	14858
30	353.2653	15.5	17409
31	359.9522	100.0	112520
32	360.3228	20.6	23168
33	360.9547	12.0	13556
34	361.3259	6.0	6724
35	373.9675	6.1	6877
36	381.2965	10.4	11717
37	413.2653	12.2	13763
38	413.9990	54.9	61823
39	415.0016	8.8	9918
40	419.3129	6.6	7399
41	428.0143	32.0	35982
42	429.0170	6.2	7010
43	439.1240	6.8	7625
44	439.8716	9.3	10459
45	441.2966	62.4	70242
46	442.0298	14.1	15882
47	442.2997	18.2	20475
48	455.3118	13.9	15643
49	456.0454	10.8	12195
50	457.2703	6.4	7246
51	467.1009	36.5	41058
52	468.1018	15.0	16929
53	469.0993	10.6	11952
54	469.3263	7.5	8452
55	470.0608	9.5	10704
56	475.3245	27.8	31268
57	476.3273	7.2	8132
58	478.3877	6.0	6802
59	482.0610	8.3	9321
60	484.0764	9.3	10476
61	496.0767	7.0	7849
62	497.3951	6.3	7057

Bruker Compass DataAnalysis 4.0 Acquisition Date 21.07.2020 10:00:28 Page 2 of 3

Figure S1.8: HRMS (ESI) peak table of 6.

High Resolution Mass Spectrometry Report

#	m/z	I %	I
63	508.1875	6.3	7037
64	513.1426	7.3	8208
65	536.1646	83.3	93734
66	537.1650	42.1	47391
67	538.1629	29.0	32612
68	539.1629	10.6	11953
69	541.1198	71.3	80254
70	542.1203	34.1	38329
71	543.1179	24.9	28071
72	544.1182	8.3	9309
73	553.4576	24.3	27290
74	554.4613	9.5	10692
75	557.0935	14.0	15724
76	558.0944	6.6	7389
77	559.0921	6.0	6707
78	559.1297	6.3	7082
79	569.4315	8.1	9167
80	610.1832	53.6	60346
81	611.1837	31.3	35218
82	612.1817	22.7	25531
83	613.1814	9.6	10746
84	615.1381	7.8	8801
85	684.2017	24.9	27973
86	685.2021	15.9	17925
87	685.4334	9.6	10764
88	686.2000	12.7	14332
89	687.1997	6.2	6940
90	693.9059	5.8	6552
91	722.5252	13.4	15099
92	723.5284	7.6	8596
93	758.2199	14.1	15855
94	759.2205	10.8	12119
95	760.2188	9.6	10822
96	832.2383	9.4	10570
97	833.2393	7.4	8365
98	834.2374	6.1	6860
99	883.2479	5.7	6435
100	906.2569	5.8	6494

Acquisition Parameter

**General**	Fore Vacuum<br>Scan Begin	2.39e+000 mBar<br>75 m/z	High Vacuum<br>Scan End	1.21e-007 mBar<br>2000 m/z	Source Type<br>Ion Polarity	ESI<br>Positive
**Source**	Set Nebulizer<br>Set Dry Heater	2.0 Bar<br>200 °C	Set Capillary<br>Set End Plate Offset	4500 V<br>-500 V	Set Dry Gas	8.0 l/min
**Quadrupole**	Set Ion Energy (MS only)	4.0 eV				
**Coll. Cell**	Collision Energy	8.0 eV	Set Collision Cell RF	600.0 Vpp	100.0 Vpp	
**Ion Cooler**	Set Ion Cooler Transfer Time	75.0 µs	Set Ion Cooler Pre Pulse Storage Time	10.0 µs		

Bruker Compass DataAnalysis 4.0 Acquisition Date 21.07.2020 10:00:28 Page 3 of 3

Figure S1.9: HRMS (ESI) peak table of 6.

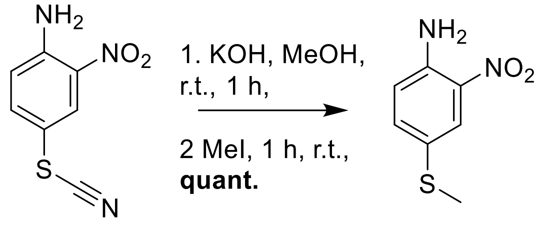

5a

4-(methylthio)-2-nitroaniline 5a: An argon flushed round bottom flask was charged with KOH (845 mg, 12.8 mmol, 5 eq.) and MeOH (15 mL). The mixture was sparged with argon for 15 min and added to a degassed mixture of 2-nitro-4-thiocyanatoaniline (500 mg, 2.56 mmol, 1 eq.) in MeOH (10 mL). The mixture was stirred under argon for 1 h. Then methyl iodide (0.32 mL, 5.12 mmol, 2 eq.) was added at once and the reaction mixture was stirred at r.t. for 1 h. The reaction was quenched with aq. NH_3 and extracted with DCM. The combined organic phase was dried over anhydrous $MgSO_4$, filtered and concentrated under reduced pressure yielding 5a as a red solid (470 mg, 2.55 mmol, quant.)

^{1}H-NMR (500 MHz, $CDCl_3$) δ 8.06 (d, J = 2.2 Hz, 1H), 7.35 (dd, J = 8.7, 2.2 Hz, 1H), 6.77 (d, J = 8.7 Hz, 1H), 6.05 (s, 2H), 2.47 (s, 3H).

^{13}C-NMR (126 MHz, $CDCl_3$) δ 143.21, 137.01, 132.56, 126.06, 125.33, 119.62, 17.90.

IR *ν*(cm^{-1}): 3473.03, 3340.92, 3161.55, 6112.85, 2920.95, 2851.52, 1631.64, 1576.68, 1554.98, 1498.08, 1448.42, 1403.10, 1361.63, 1330.29, 1237.72, 1172.62, 1106.57, 953.24, 897.31, 858.73, 819.68, 761.34, 718.91, 643.69, 530.38

GC-MS (EI): *m/z* (%) = 183.8 (100), 168.8 (16.9), 137.9 (62.3), 123.0 (21.3), 110.8 (13.8), 90.9 (26.5)

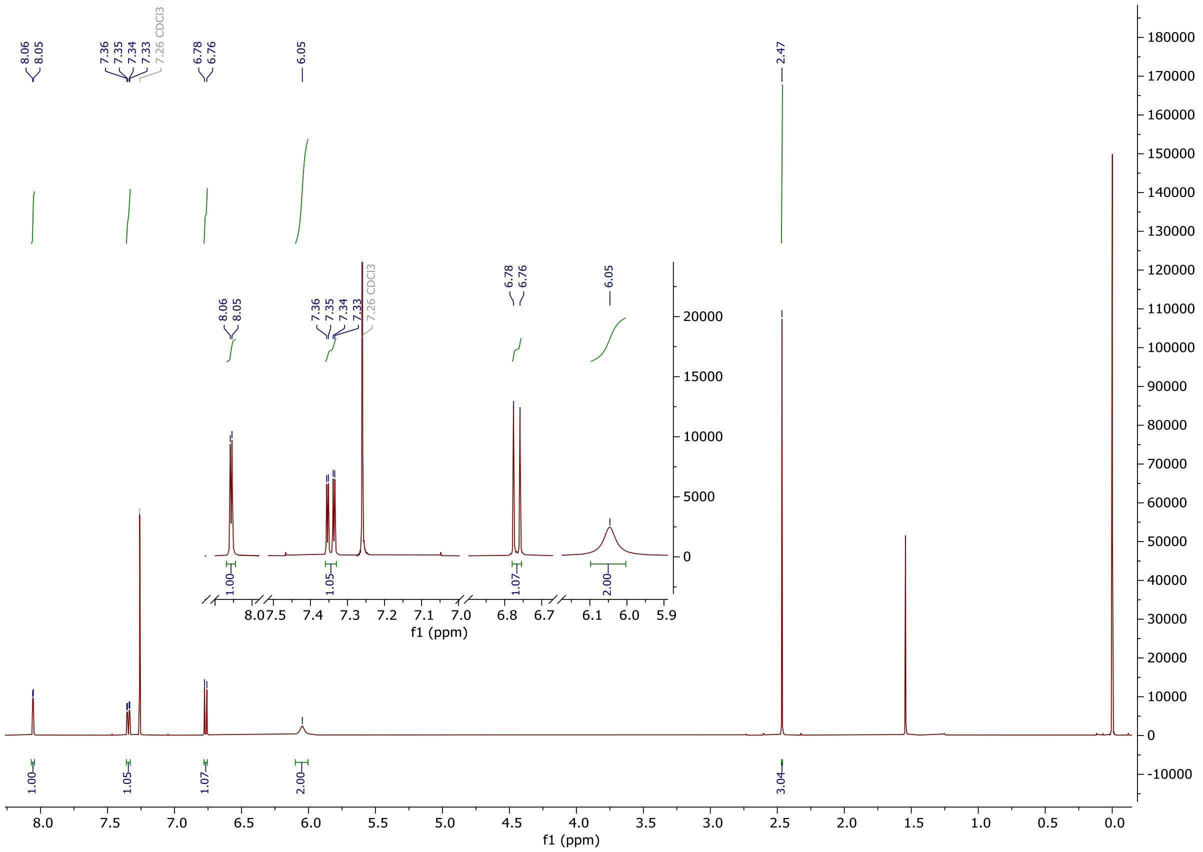

Figure S1.10: ^{1}H-NMR (500 MHz, $CDCl_3$) spectrum of 5a.

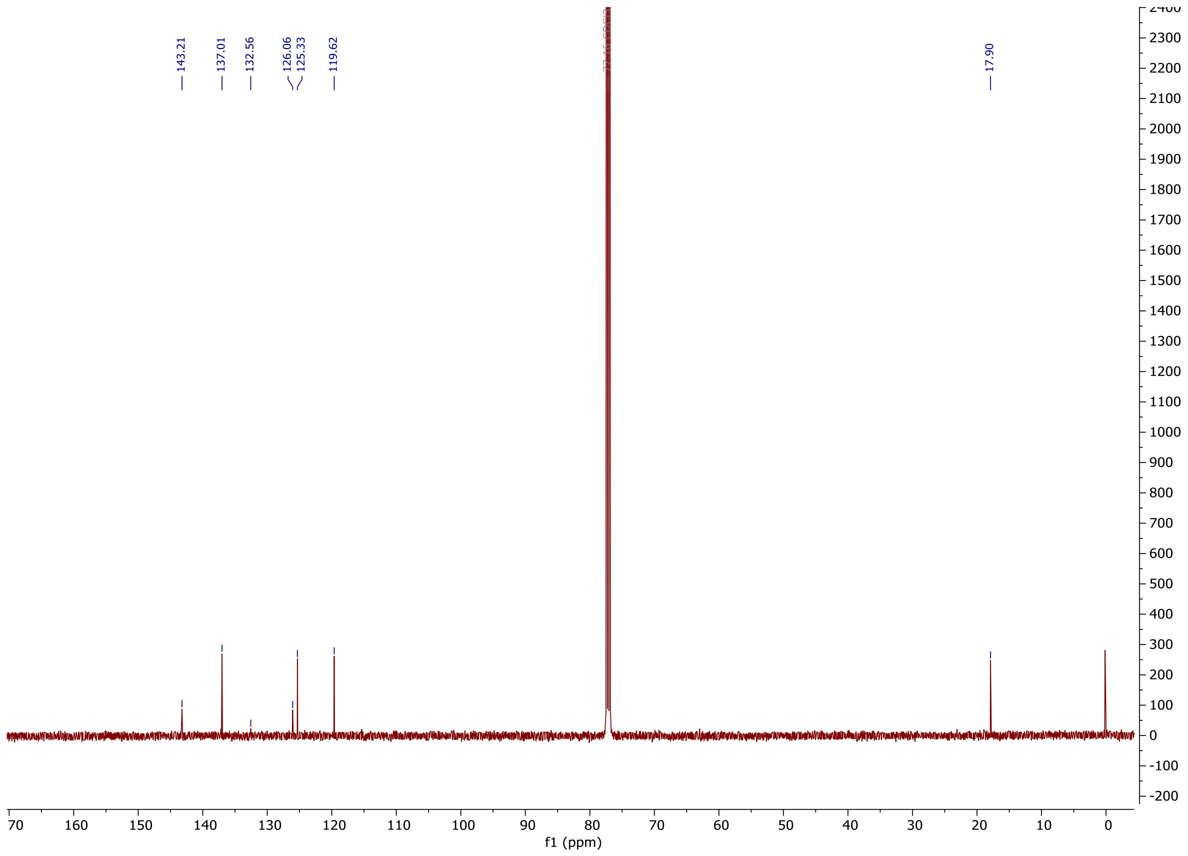

Figure S1.11: ^{13}C-NMR (126 MHz, $CDCl_3$) spectrum of 5a.

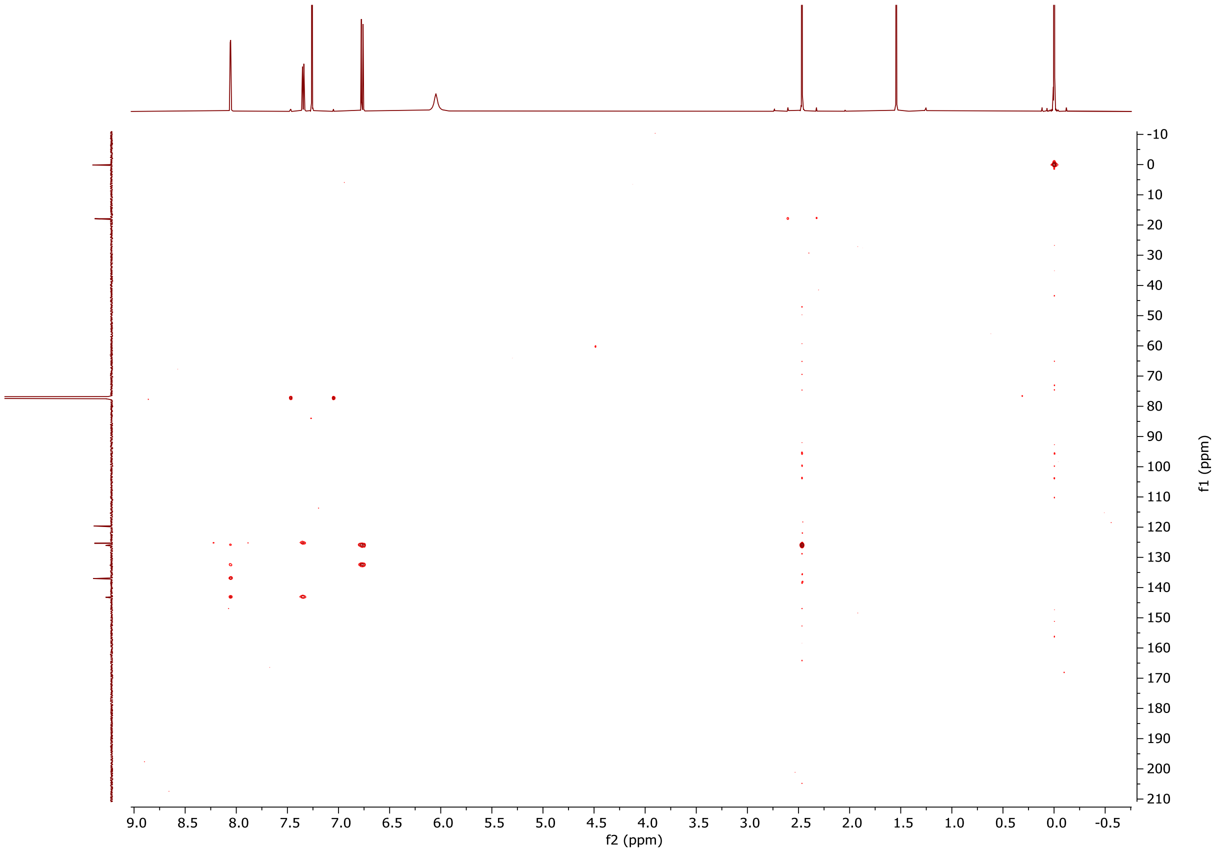

Figure S1.12: HMBC_GPSW ($CDCl_3$) spectrum of 5a.

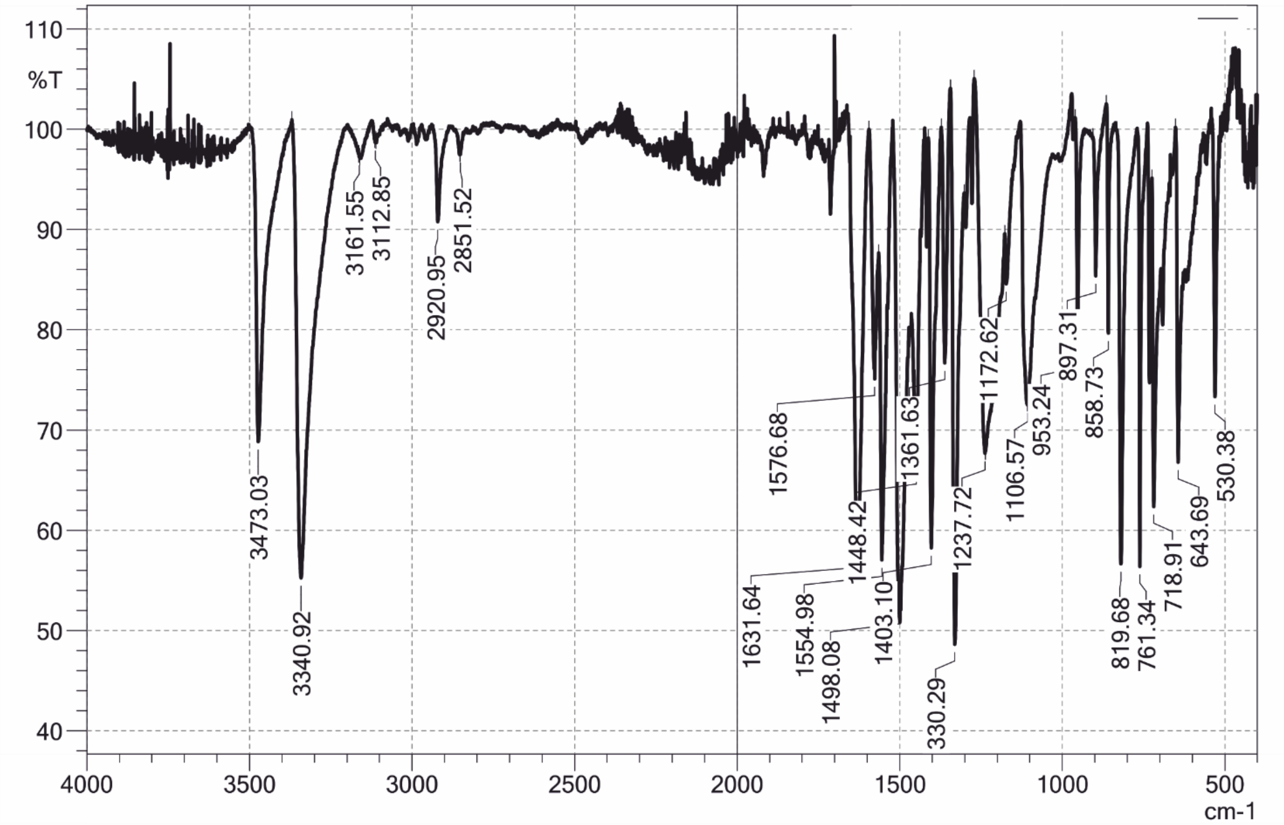

	Item	Value
2	Sample name	VOE_359
3	Sample ID	VOE_359
4	Option	
5	Intensity Mode	%Transmittance
6	Apodization	Happ-Genzel
9	No. of Scans	20
10	Resolution	1 cm-1

	Peak	Intensity	Corr. Intensity	Base (H)	Base (L)	Area	Corr. Area	Comment
1	530.38	73.34	25.21	537.13	519.77	227.282	196.608	
2	643.69	66.78	27.23	651.40	631.64	391.408	238.425	
3	718.91	62.36	29.42	724.21	703.96	493.891	266.158	
4	761.34	56.39	40.50	766.64	752.66	245.580	191.070	
5	819.68	56.66	40.97	831.73	796.54	638.263	517.294	
6	858.73	79.66	21.59	865.00	849.09	79.003	93.340	
7	897.31	85.38	13.91	905.50	878.50	131.600	113.807	
8	953.24	77.94	22.48	960.47	939.74	150.791	153.122	
9	1106.57	72.49	0.67	1107.53	1084.39	517.545	7.957	
10	1172.62	84.58	6.09	1176.96	1154.78	243.056	73.253	
11	1237.72	67.67	1.27	1270.98	1236.75	489.033	25.752	
12	1330.29	48.62	52.02	1344.27	1301.84	974.007	925.720	
13	1361.63	76.70	24.97	1372.24	1344.27	252.914	312.771	
14	1403.10	58.25	41.09	1411.78	1372.24	691.717	677.906	
15	1448.42	66.28	14.41	1456.62	1437.81	483.038	132.984	
16	1498.08	52.35	0.09	1498.57	1497.60	45.901	0.045	
17	1554.98	57.04	4.58	1564.62	1554.02	286.331	3.598	
18	1576.68	75.11	4.52	1594.52	1575.71	242.023	40.170	
19	1631.64	57.95	4.30	1646.11	1629.23	511.520	32.365	
20	2851.52	97.49	0.20	2852.00	2845.73	11.583	0.715	
21	2920.95	90.77	7.00	2932.04	2911.31	103.995	60.166	
22	3112.85	98.64	0.32	3114.30	3110.44	4.779	0.749	
23	3161.55	97.19	0.15	3163.96	3160.59	9.043	0.280	
24	3340.92	55.26	34.53	3369.36	3308.61	1691.820	1025.784	
25	3473.03	68.86	25.64	3487.98	3446.99	770.948	485.269	

Figure S1.13: FT-IR spectra and peak table of 5a.

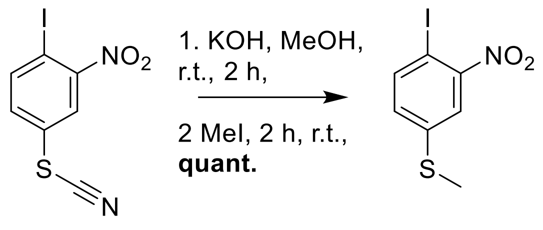

4 5

(4-iodo-3-nitrophenyl)(methyl)sulfane 5: An argon flushed round bottom flask was charged with KOH (53.8 mg, 815 µmol, 5 eq.) and MeOH (4 mL). The mixture was sparged with argon for 15 min and added to a degassed mixture of 2-nitro-4-thiocyanatoaniline (50 mg, 163 µmol, 1 eq.) in MeOH (2 mL) and the mixture was stirred under argon for 2 h. Methyl iodide (0.02 mL, 326 µmol, 2eq.) was added at once and the reaction mixture was stirred at r.t. for 2 h. The reaction was quenched with aq.NH_3 and extracted with DCM. The combined organic phase was washed with water, Brine, dried over anhydrous $MgSO_4$, filtered and concentrated under reduced pressure yielding 5 as a yellow solid (48.0 mg, 163 µmol, quant.)

Alternative way of synthesizing 5:

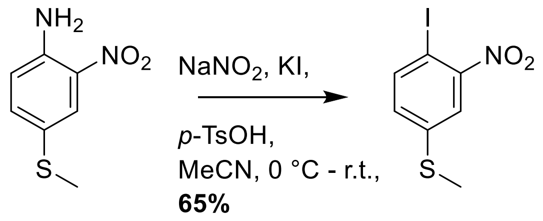

5a 5

(4-iodo-3-nitrophenyl)(methyl)sulfane 5: An oven dried argon flushed two necked round bottom flask was charged with aniline 5a (400 mg, 2.17 mmol, 1eq.) and *p*-TsOH (1.24 g, 6.51 mmol, 3 eq.) suspended in MeCN (9 mL). The mixture was cooled to 0 °C in an ice bath. A solution of $NaNO_2$ (299 mg, 4.34 mmol, 2 eq.) and KI (901 mg, 5.43 mmol, 2.5 eq.) in water (1.5 mL) was added gradually into the flask. The mixture was stirred at 0 °C for 10 min and left to warm up to r.t. over 2 h. The reaction was quenched with aq. sat. $NaHCO_3$ diluted with aq. sat. $Na_2S_2O_3$ and extracted with EtOAc. The combined organic phase was dried over anhydrous $MgSO_4$, filtered and concentrated under reduced pressure yielding 5 as a yellow solid (416 mg, 1.41 mmol, 65%).

^{1}H-NMR (500 MHz, $CDCl_3$) δ 7.86 (d, J = 8.4 Hz, 1H), 7.65 (d, J = 2.2 Hz, 1H), 7.10 (dd, J = 8.4, 2.3 Hz, 1H), 2.52 (s, 3H).

^{13}C-NMR (126 MHz, $CDCl_3$) δ 153.26, 141.96, 141.56, 130.66, 122.05, 80.25, 15.28.

HRMS (ESI) m/z: calcd. for $[C_7H_6NIO_2S+Na]^+$ 317.9051 $[M+Na]^+$; found 317.9056

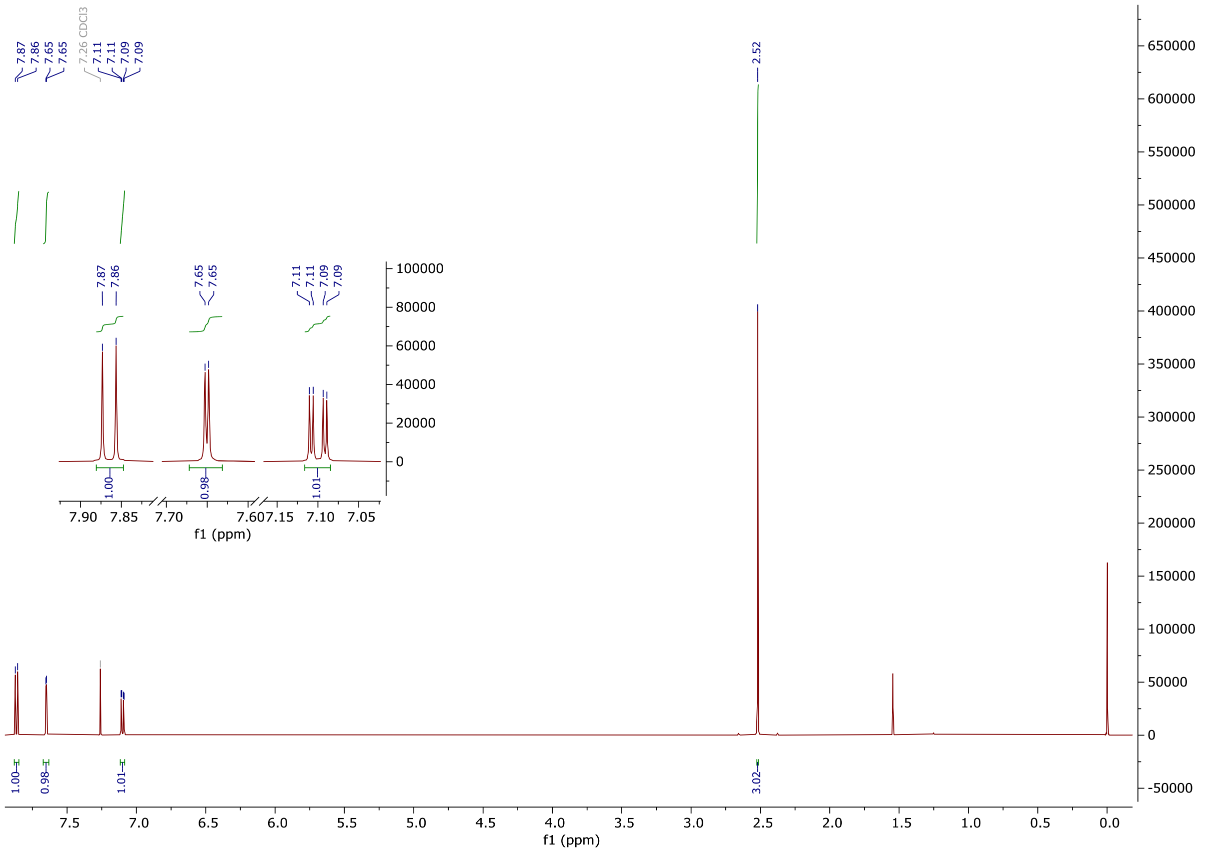

Figure S1.14: ^{1}H-NMR (500 MHz, $CDCl_3$) spectrum of 5.

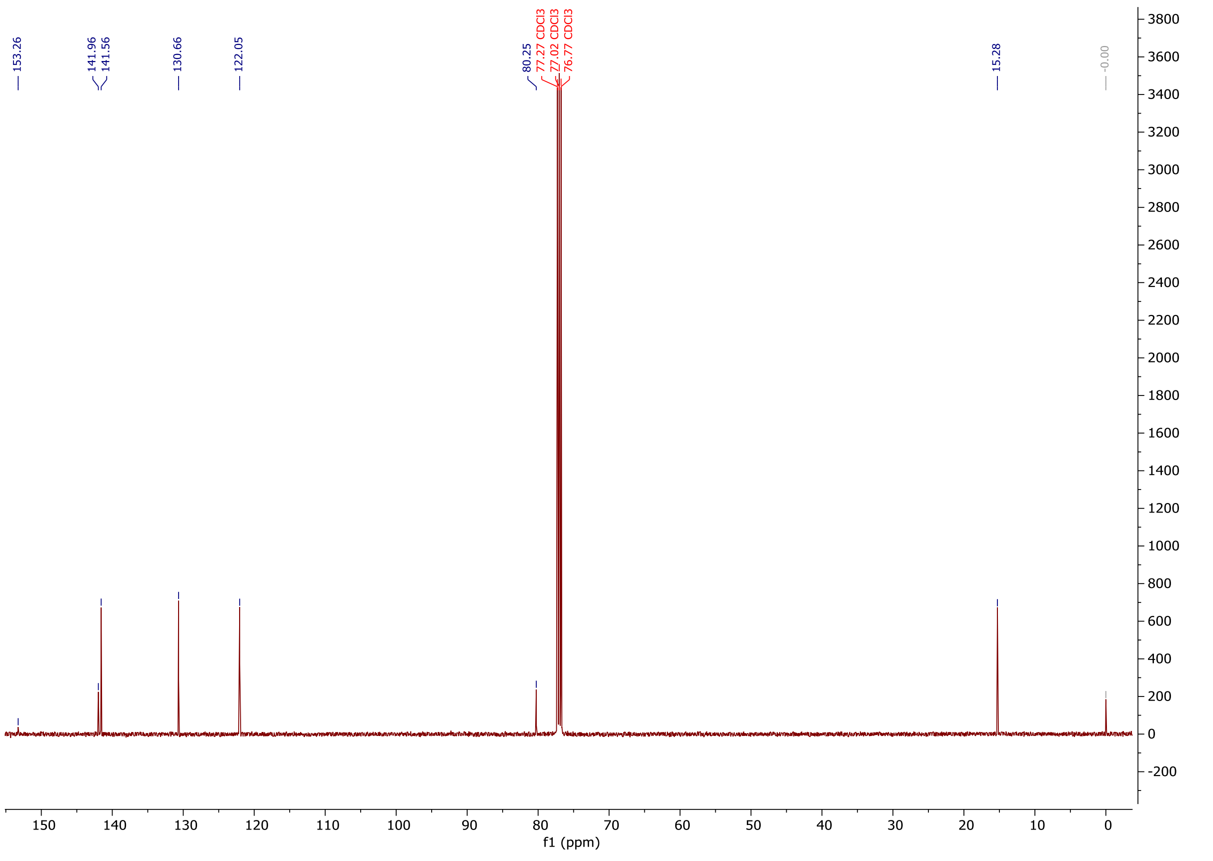

Figure S1.15: ^{13}C-NMR (126 MHz, $CDCl_3$) spectrum of 5.

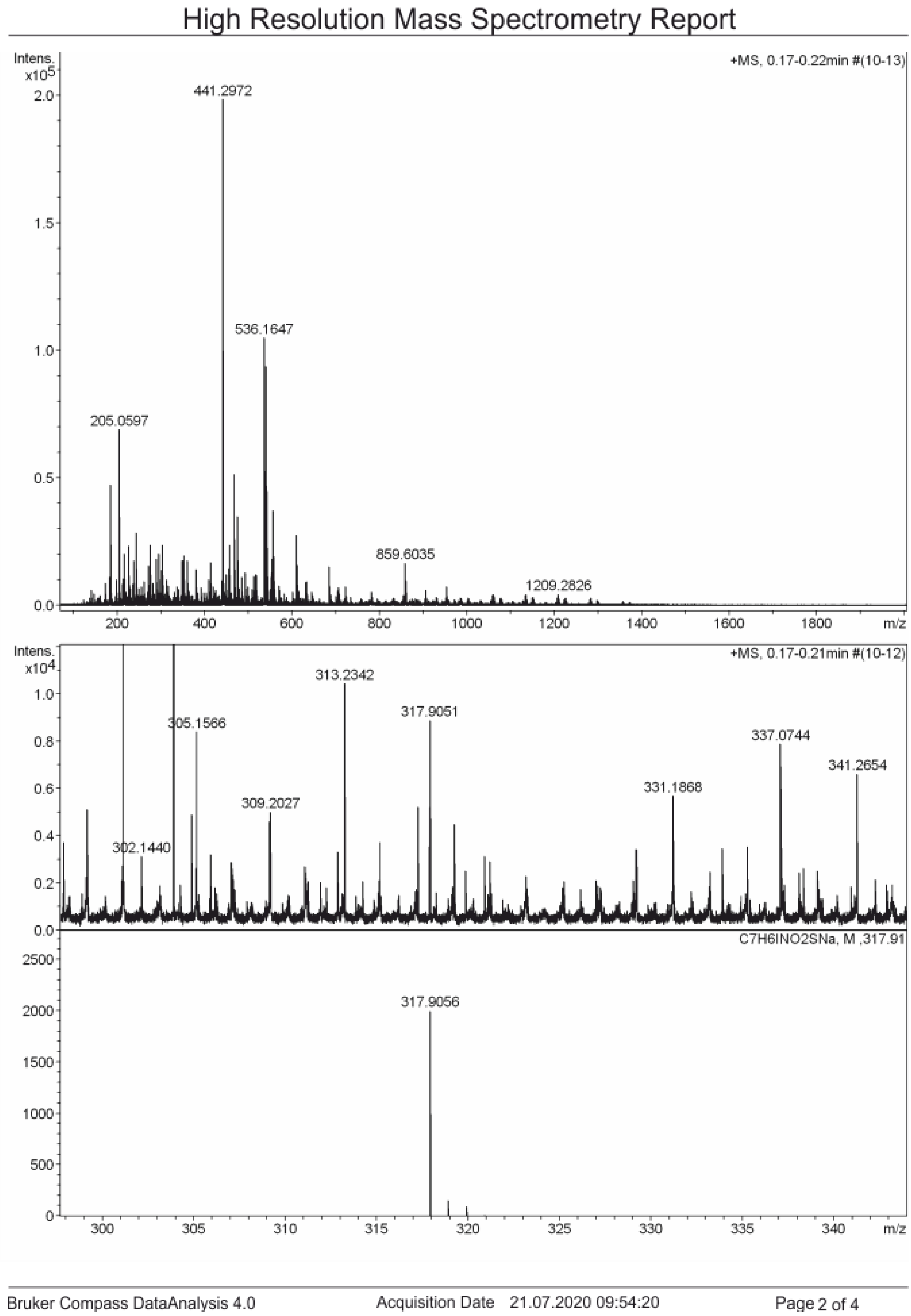

Figure S1.16: HRMS (ESI) spectrum of 5.

High Resolution Mass Spectrometry Report

Measured m/z vs. theoretical m/z

Meas. m/z	#	Formula	Score	m/z	err [mDa]	err [ppm]	mSigma	rdb	e⁻ Conf	z
317.9051	1	C 7 H 6 I N Na O 2 S	100.00	317.9056	0.5	1.6	58.2	4.5	even	1+

Meas. m/z # Formula Score m/z err [mDa] err [ppm] mSigma rdb e⁻ Conf z

Mass list

#	m/z	I %	I
1	173.0782	4.5	9496
2	183.0778	5.4	11333
3	185.1145	22.4	47358
4	199.1299	5.0	10555
5	205.0596	31.9	67469
6	213.1459	4.1	8600
7	215.1248	5.2	10964
8	216.9790	4.0	8367
9	217.0467	6.4	13602
10	217.1043	10.1	21276
11	226.9510	10.9	23077
12	227.1246	5.3	11286
13	229.1403	3.9	8223
14	229.8925	3.3	6887
15	236.0710	4.1	8648
16	239.0885	8.3	17508
17	243.9411	3.6	7632
18	245.0778	13.2	27803
19	255.1558	3.6	7626
20	261.1304	4.6	9734
21	271.1872	7.5	15814
22	273.1666	3.5	7463
23	275.1613	11.2	23728
24	280.9400	4.3	9073
25	288.9214	8.7	18276
26	294.9191	9.7	20401
27	301.1402	6.7	14196
28	303.8968	11.2	23663
29	305.1566	4.0	8393
30	305.1707	3.4	7115
31	313.2342	4.9	10441
32	317.9051	4.2	8899
33	337.0744	3.7	7884
34	341.2654	3.1	6631
35	348.9894	8.2	17259
36	350.9863	8.4	17786
37	353.1445	8.4	17729
38	353.2654	9.8	20784
39	360.3229	8.5	18039
40	381.2968	7.2	15158
41	393.2962	3.6	7565
42	411.0927	5.1	10854
43	413.2655	8.5	17865
44	419.3147	3.6	7594
45	425.1086	3.1	6578
46	439.1242	4.7	9877
47	439.8715	4.6	9664
48	441.2972	100.0	211207
49	442.3002	27.0	56946
50	443.3026	4.5	9470
51	449.2863	6.2	13051
52	455.3121	7.1	15054
53	457.2704	11.9	25215
54	458.2741	3.6	7682
55	467.1014	25.6	53980
56	468.1016	10.9	23127
57	469.0996	7.1	14984
58	469.3266	5.1	10726
59	475.3243	16.5	34758
60	476.3275	4.4	9296

Bruker Compass DataAnalysis 4.0 Acquisition Date 21.07.2020 09:54:20 Page 3 of 4

Figure S1.17: HRMS (ESI) peak table of 5.

High Resolution Mass Spectrometry Report

#	m/z	I %	I
61	478.3881	3.5	7482
62	485.1116	5.7	11995
63	493.3128	6.4	13596
64	499.1268	3.8	8015
65	508.1872	3.1	6603
66	513.1427	5.7	12125
67	517.2943	6.0	12673
68	519.2945	6.0	12720
69	536.1646	50.6	106948
70	537.1652	24.0	50720
71	537.3387	4.7	9864
72	538.1630	16.7	35280
73	539.1630	6.8	14258
74	541.1203	46.5	98268
75	542.1207	22.5	47456
76	543.1185	15.1	31791
77	544.1183	5.9	12419
78	550.1800	3.3	6945
79	553.4579	8.9	18865
80	554.4612	3.3	6923
81	557.0939	18.4	38874
82	558.0944	9.6	20203
83	559.0920	7.9	16609
84	559.1296	5.8	12235
85	560.0925	3.3	6947
86	560.1301	3.3	6909
87	569.4317	3.8	8058
88	610.1832	13.4	28215
89	611.1835	7.8	16397
90	612.1818	6.0	12625
91	631.1122	4.5	9535
92	633.1484	4.9	10353
93	684.2010	3.1	6621
94	685.4331	7.6	15956
95	686.4371	3.6	7576
96	707.1674	3.7	7716
97	722.5256	3.8	7953
98	859.6035	8.5	17874
99	860.6064	4.9	10332
100	952.7973	3.6	7664

#	m/z	I %	I
1	317.9056	100.0	2000
2	318.9026	0.4	7
3	318.9089	8.4	167
4	319.9014	4.6	92
5	319.9099	0.7	13
6	320.9048	0.4	8

Acquisition Parameter

**General**	Fore Vacuum<br>Scan Begin	2.39e+000 mBar<br>75 m/z	High Vacuum<br>Scan End	1.21e-007 mBar<br>2000 m/z	Source Type<br>Ion Polarity	ESI<br>Positive
**Source**	Set Nebulizer<br>Set Dry Heater	2.0 Bar<br>200 °C	Set Capillary<br>Set End Plate Offset	4500 V<br>-500 V	Set Dry Gas	8.0 l/min
**Quadrupole**	Set Ion Energy (MS only)	4.0 eV				
**Coll. Cell**	Collision Energy	8.0 eV	Set Collision Cell RF	600.0 Vpp	100.0 Vpp	
**Ion Cooler**	Set Ion Cooler Transfer Time	75.0 µs	Set Ion Cooler Pre Pulse Storage Time	10.0 µs		

Bruker Compass DataAnalysis 4.0 Acquisition Date 21.07.2020 09:54:20 Page 4 of 4

Figure S1.18: HRMS (ESI) peak table of 5.

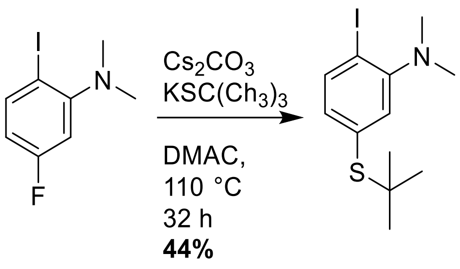

7 9

5-(*tert*-butylthio)-2-iodo-*N,N*-dimethylaniline 9: A Schlenk tube was charged with fluorine 7 (200 mg, 844 µmol, 1 eq.), Cs_2CO_3 (825 mg, 2.53 mmol, 3 eq.) and $KSC(CH_3)_3$ (169 mg, 1.51 mmol, 2 eq.). The solids were purged with argon and dissolved in degassed dry DMAc (1.2 mL). The reaction mixture was heated to 110°C and stirred for 32 h. The mixture was then cooled to r.t., diluted with water and extracted with DCM. The combined organic phase was dried over anhydrous $MgSO_4$, filtered, concentrated under reduced pressure and purified by column chromatography on silica gel (DCM) yielding 9 as a yellowish oil (110 mg, 328 µmol, 44%).

^{1}H-NMR (500 MHz, $CDCl_3$) δ 7.78 (d, J = 8.1 Hz, 1H), 7.19 (d, J = 2.1 Hz, 1H), 6.92 (dd, J = 8.1, 2.1 Hz, 1H), 2.77 (s, 6H), 1.29 (s, 9H).

^{13}C-NMR (126 MHz, $CDCl_3$) δ 155.10, 140.09, 133.94, 133.72, 129.61, 98.27, 46.25, 45.09, 31.11.

HRMS (ESI) m/z: calcd. for $[C_{12}H_{19}INS+H]^+$ 336.0277 $[M+H]^+$; found 336.0277

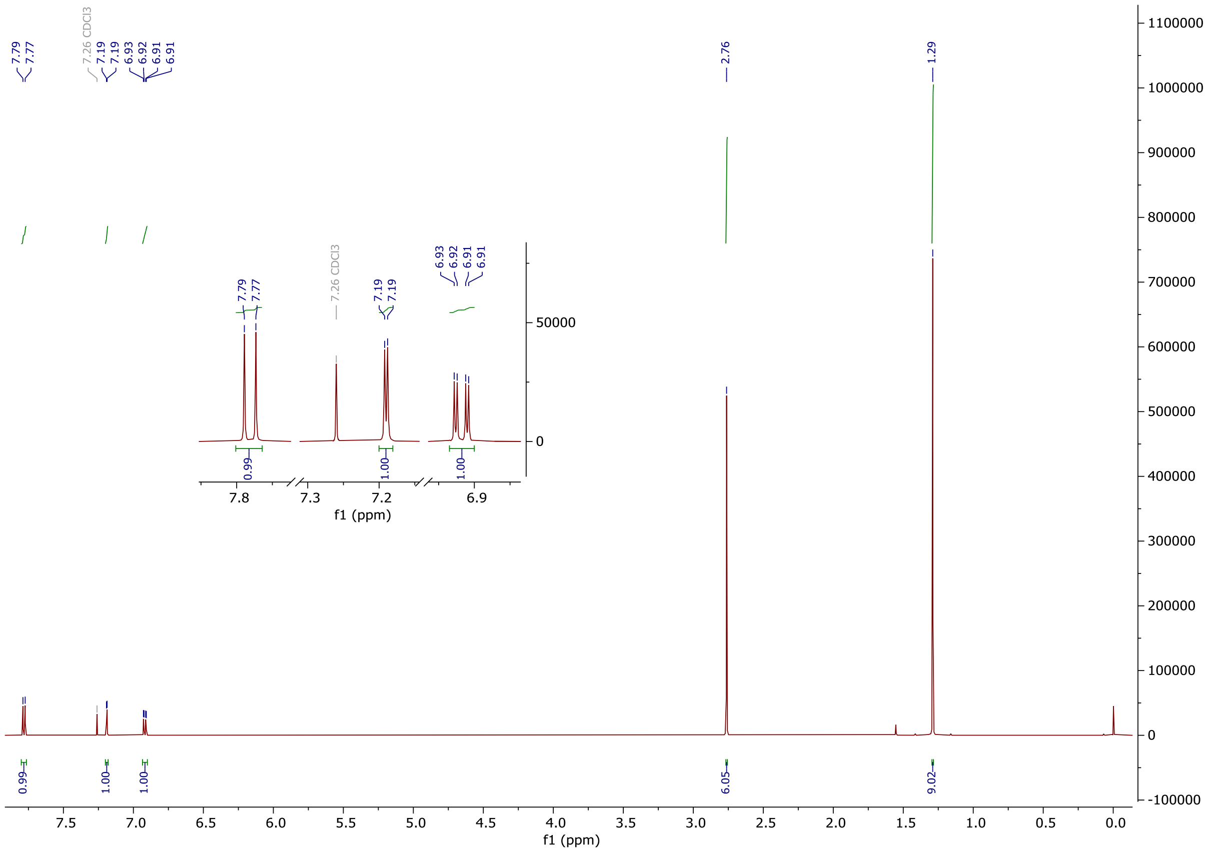

Figure S1.19: ^{1}H-NMR (500 MHz, $CDCl_3$) spectrum of 9.

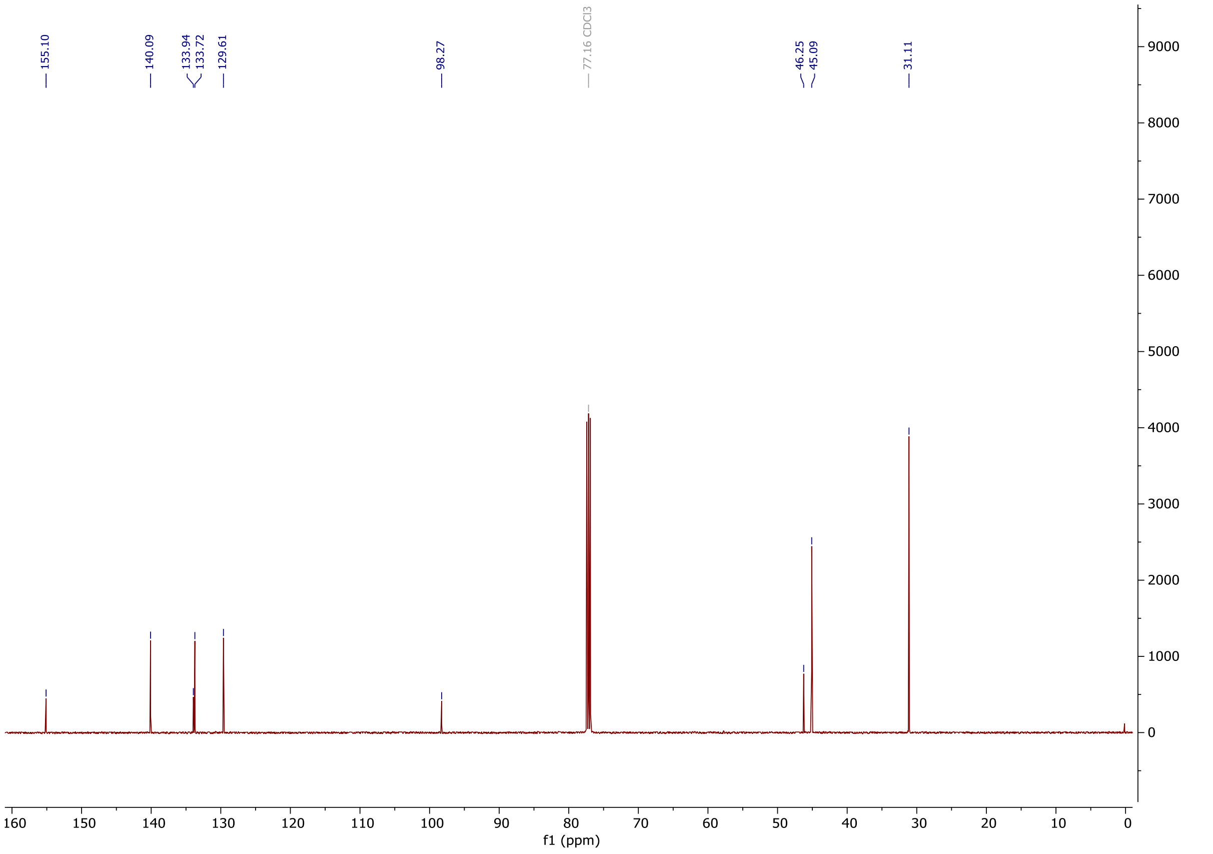

Figure S1.20: ^{13}C-NMR (126 MHz, $CDCl_3$) spectrum of 9.

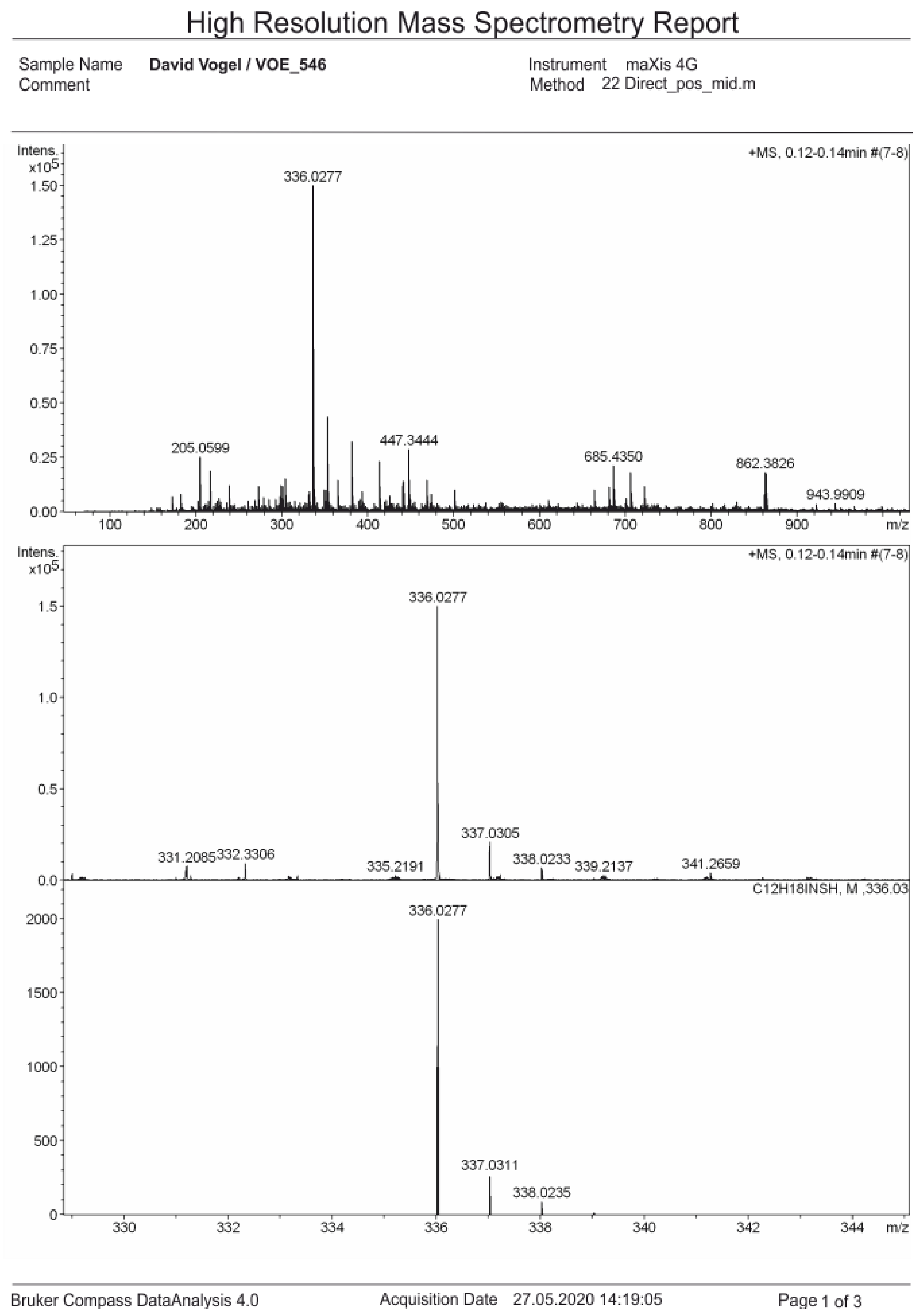

Figure S1.21: HRMS (ESI) spectrum of 9.

High Resolution Mass Spectrometry Report

Measured m/z vs. theoretical m/z

Meas. m/z	#	Formula	Score	m/z	err [mDa]	err [ppm]	mSigma	rdb	e⁻ Conf	z
336.0277	1	C 12 H 19 I N S	100.00	336.0277	0.1	0.2	4.0	3.5	even	1+

Mass list

#	m/z	I %	I
1	173.0783	4.9	7318
2	183.0781	5.7	8520
3	203.0525	3.6	5381
4	205.0599	17.0	25573
5	215.1249	3.5	5303
6	217.1047	12.8	19221
7	223.0940	2.7	4029
8	225.1091	3.5	5225
9	227.1251	4.2	6367
10	229.1407	3.1	4691
11	236.0710	3.1	4618
12	239.0884	8.1	12217
13	261.1297	3.4	5054
14	269.1355	3.7	5581
15	273.1668	8.0	11952
16	279.2288	4.4	6626
17	285.1304	4.1	6105
18	293.1731	2.9	4291
19	293.2080	3.9	5899
20	298.1658	4.8	7251
21	299.1617	8.2	12337
22	301.1404	8.1	12153
23	301.1612	3.4	5068
24	304.2991	10.3	15540
25	305.3028	2.6	3962
26	307.2602	3.0	4568
27	309.2040	3.2	4788
28	315.1563	3.4	5123
29	321.2394	2.9	4299
30	327.0079	3.0	4547
31	329.0050	2.7	4025
32	331.1872	3.6	5399
33	331.2085	5.3	7955
34	332.3306	6.5	9738
35	336.0277	100.0	150219
36	337.0305	14.1	21198
37	338.0233	4.8	7170
38	341.2659	3.0	4548
39	348.9894	6.9	10383
40	350.9866	7.0	10467
41	353.2296	2.8	4186
42	353.2658	29.2	43897
43	354.2691	6.5	9737
44	365.1050	9.7	14553
45	381.2969	21.6	32446
46	382.3005	5.2	7772
47	389.2503	3.5	5228
48	391.2831	4.1	6138
49	393.2968	6.4	9575
50	407.3123	2.8	4185
51	413.2656	15.5	23277
52	414.2689	4.3	6529
53	419.3146	3.2	4749
54	421.3284	3.7	5611
55	425.3617	5.1	7606
56	427.2663	2.7	4118
57	435.3439	2.7	4130
58	441.2969	9.7	14499
59	442.3000	3.2	4833
60	447.3444	19.3	29045
61	448.3476	5.7	8575
62	449.3600	4.0	5943

Bruker Compass DataAnalysis 4.0 Acquisition Date 27.05.2020 14:19:05 Page 2 of 3

Figure S1.22: HRMS (ESI) peak table of 9.

High Resolution Mass Spectrometry Report

#	m/z	I %	I
63	452.8700	2.7	3989
64	455.3122	3.2	4737
65	463.3742	2.6	3978
66	469.3278	9.8	14701
67	470.3309	3.4	5057
68	473.3443	2.7	4129
69	473.4688	5.5	8283
70	481.3133	2.8	4248
71	501.4997	6.9	10330
72	537.3935	3.1	4595
73	553.3884	2.8	4169
74	555.5106	3.1	4658
75	610.1835	3.6	5474
76	622.0284	2.7	4053
77	644.0102	2.9	4389
78	663.4529	7.0	10551
79	664.4559	3.4	5106
80	680.4788	7.6	11389
81	681.4827	3.9	5871
82	685.4350	14.3	21468
83	686.4380	7.2	10792
84	699.5942	2.8	4141
85	700.6253	4.3	6503
86	705.5816	12.3	18422
87	706.5846	5.7	8632
88	721.5754	8.0	12081
89	722.5789	4.2	6372
90	829.7225	3.2	4846
91	861.3820	5.4	8051
92	861.8835	5.2	7854
93	862.3826	12.2	18393
94	862.8833	11.6	17469
95	863.3832	11.9	17812
96	863.8844	7.8	11717
97	864.3836	4.3	6509
98	943.9909	2.7	4074
99	1221.9876	3.2	4808
100	1243.9726	3.1	4706

Acquisition Parameter

**General**	Fore Vacuum<br>Scan Begin	2.61e+000 mBar<br>75 m/z	High Vacuum<br>Scan End	1.14e-007 mBar<br>1700 m/z	Source Type<br>Ion Polarity	ESI<br>Positive
**Source**	Set Nebulizer<br>Set Dry Heater	0.4 Bar<br>180 °C	Set Capillary<br>Set End Plate Offset	3600 V<br>-500 V	Set Dry Gas	4.0 l/min
**Quadrupole**	Set Ion Energy (MS only)	4.0 eV				
**Coll. Cell**	Collision Energy	8.0 eV	Set Collision Cell RF	350.0 Vpp		
**Ion Cooler**	Set Ion Cooler Transfer Time	75.0 µs	Set Ion Cooler Pre Pulse Storage Time	10.0 µs		

Bruker Compass DataAnalysis 4.0 Acquisition Date 27.05.2020 14:19:05 Page 3 of 3

Figure S1.23: HRMS (ESI) peak table of 9.

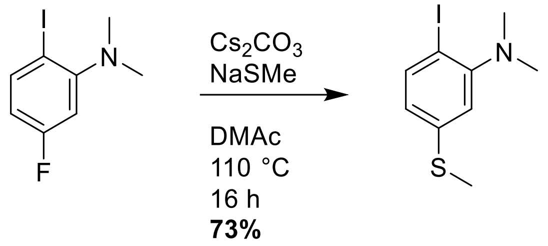

7 8

2-iodo-*N*,*N*-dimethyl-5-(methylthio)aniline 8: A Schlenk tube was charged with fluoride 7 (99.9 mg, 377 µmol, 1 eq.), Cs_2CO_3 (369 mg, 1.13 mmol, 3 eq.) and $NaSCH_3$ (52.8 mg, 754 µmol, 2 eq.). The solids were purged with argon and dry degassed DMAc (1.2 mL) was added. The mixture was stirred at 110 °C for 16 h. The mixture was then cooled to r.t., diluted with water and extracted with DCM. The combined organic phase was dried over anhydrous $MgSO_4$, filtered, concentrated under reduced pressure and purified by column chromatography on silica gel (DCM) yielding 8 as a yellowish oil (81 mg, 276 µmol, 73%).

^{1}H-NMR (500 MHz, $CDCl_3$) δ 7.71 (d, J = 8.3 Hz, 1H), 6.96 (d, J = 2.3 Hz, 1H), 6.66 (dd, J = 8.3, 2.3 Hz, 1H), 2.76 (s, 6H), 2.47 (s, 3H).

^{13}C-NMR (126 MHz, $CDCl_3$) δ 155.29, 140.40, 139.94, 122.86, 119.02, 92.22, 44.99, 16.00.

HRMS (ESI) m/z: calcd. for [$C_9H_{12}INS$+H]$^+$ 293.9812 [M+H]$^+$; found 293.9808

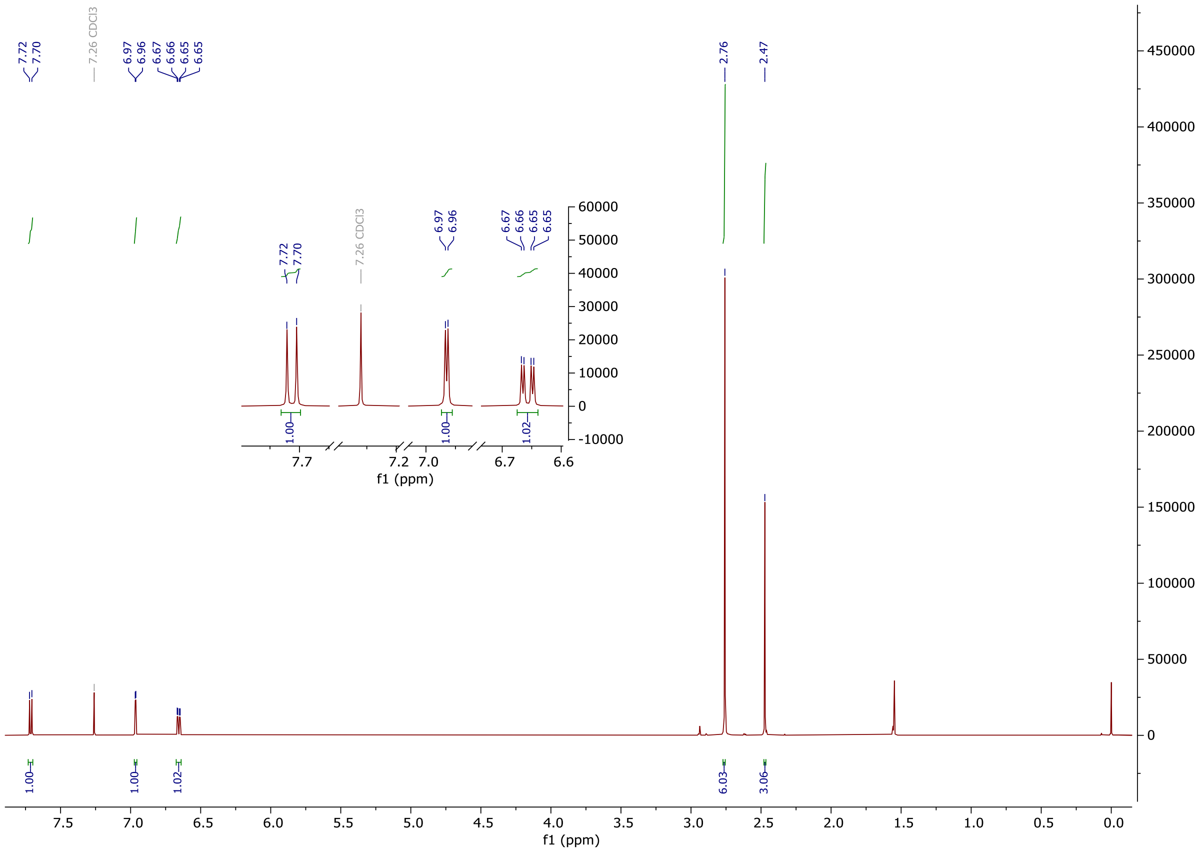

Figure S1.24: ^{1}H-NMR (500 MHz, $CDCl_3$) spectrum of 8.

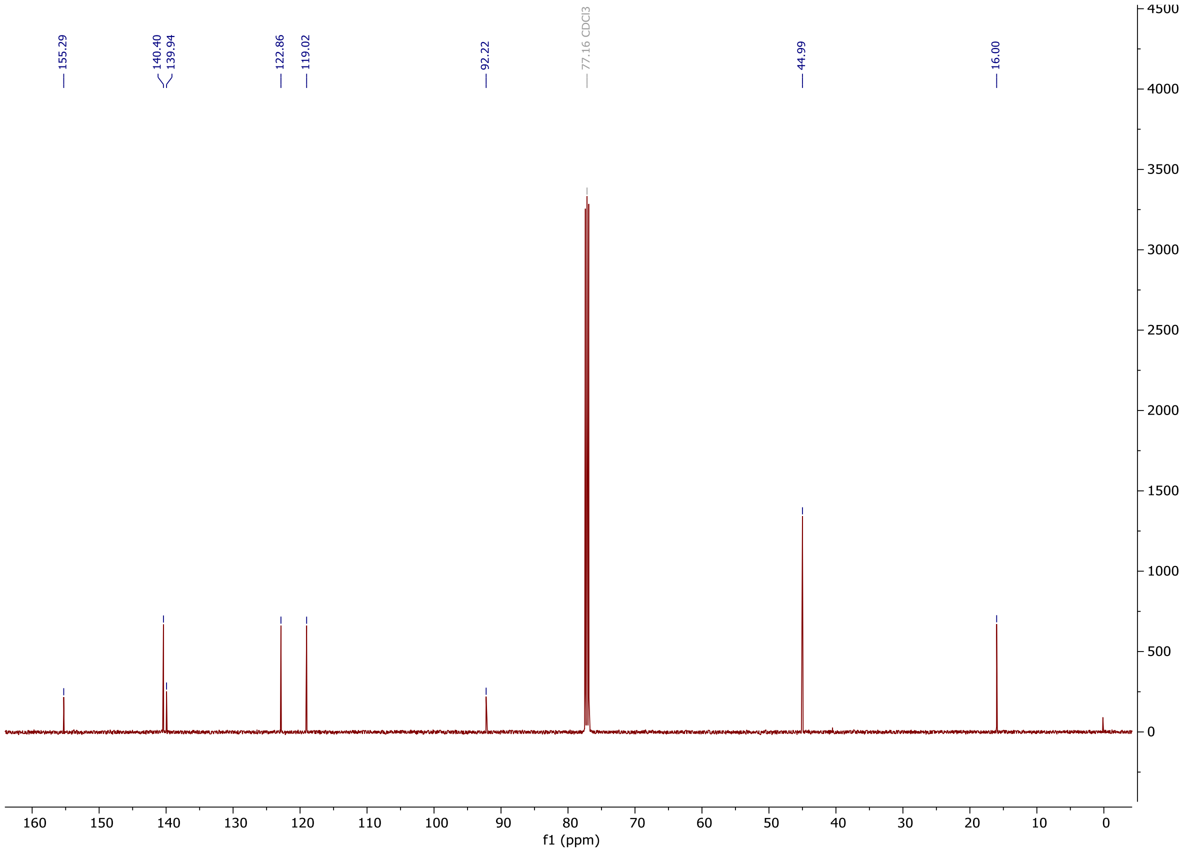

Figure S1.25: ^{13}C-NMR (126 MHz, $CDCl_3$) spectrum of 8.

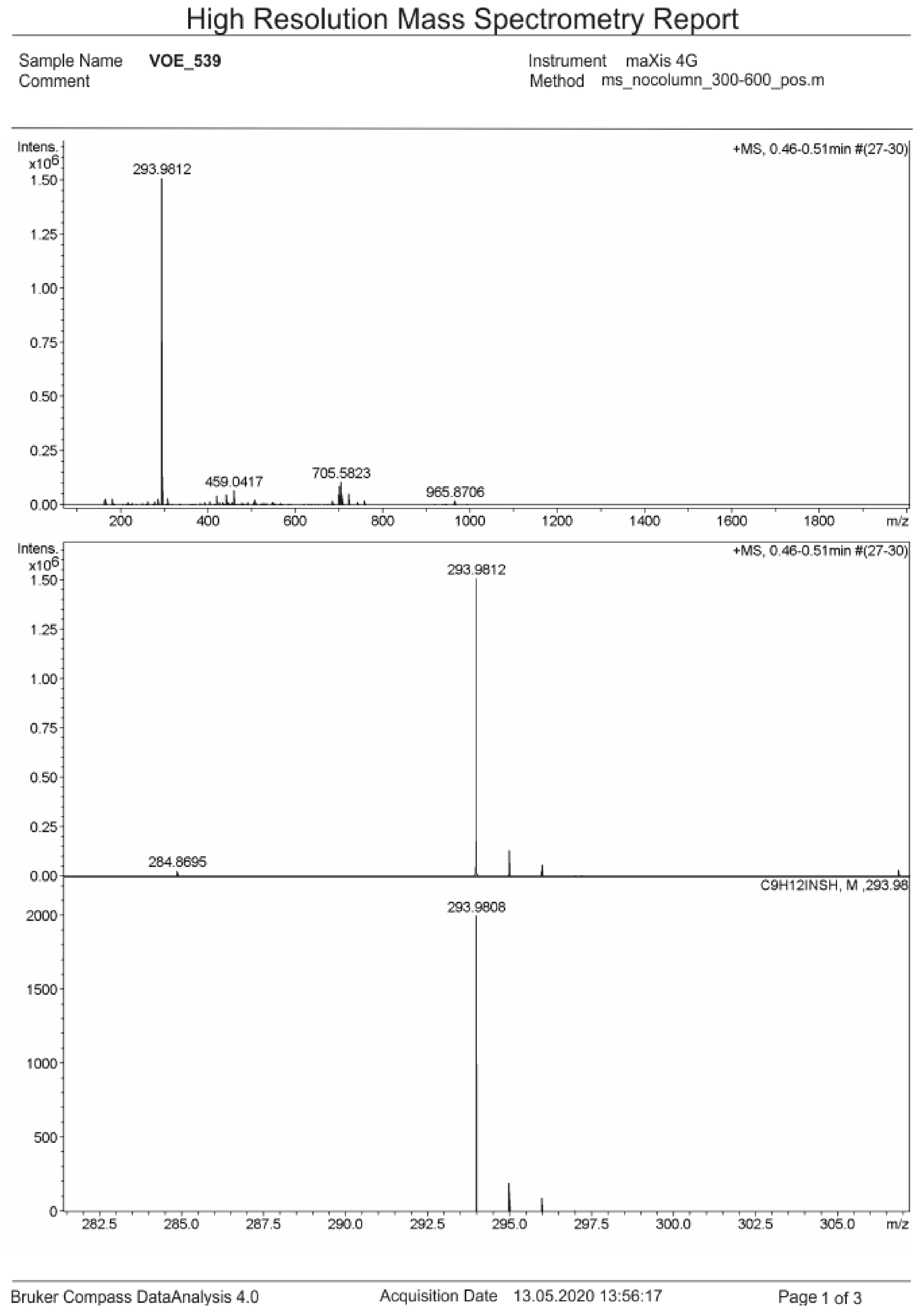

Figure S1.26: HRMS (ESI) spectrum of 8.

High Resolution Mass Spectrometry Report

Measured m/z vs. theoretical m/z

Meas. m/z	#	Formula	Score	m/z	err [mDa]	err [ppm]	mSigma	rdb	e¯ Conf	z
293.9812	1	C 9 H 13 I N S	100.00	293.9808	-0.4	-1.3	11.9	3.5	even	1+

Mass list

#	m/z	I %	I
1	163.0390	1.3	19804
2	164.9206	2.0	29802
3	168.0841	0.7	10367
4	180.0839	2.0	29909
5	183.0778	0.3	5271
6	186.0744	0.5	6975
7	214.0715	0.4	6421
8	216.9790	0.4	5543
9	217.0467	0.9	13548
10	218.9283	0.5	7370
11	226.0716	0.5	7064
12	227.0398	0.7	10626
13	249.8978	0.7	11236
14	262.8873	1.1	17199
15	275.1635	0.5	7336
16	278.0033	1.1	16415
17	278.9566	0.4	5358
18	284.8695	1.9	28460
19	293.9597	0.7	9902
20	293.9812	100.0	1510020
21	294.9836	8.9	134009
22	295.9766	3.8	57635
23	296.9797	0.4	6092
24	297.1956	0.4	5660
25	306.8513	2.2	33775
26	309.2051	0.4	5883
27	334.8749	0.3	5264
28	337.1199	0.4	6050
29	382.8367	0.7	10245
30	391.8289	1.0	14618
31	393.1482	0.5	7478
32	404.8187	1.2	18783
33	417.0285	0.4	6792
34	419.3148	3.0	45593
35	420.3182	1.0	14483
36	426.8006	1.0	15178
37	433.3303	0.8	12092
38	441.2969	3.4	50605
39	442.3001	1.0	14761
40	443.0641	0.4	5833
41	445.0255	0.9	12909
42	447.3456	0.4	5605
43	448.7826	0.6	9051
44	455.3125	0.8	11952
45	459.0417	4.5	67722
46	460.0442	1.0	14491
47	461.0382	0.4	6694
48	469.3278	0.3	5063
49	476.8067	0.5	8159
50	478.3879	0.7	10560
51	480.5132	0.6	9213
52	482.4045	0.3	5169
53	491.0133	1.0	15088
54	502.7870	0.4	5788
55	505.0292	1.0	15461
56	506.5286	1.6	23770
57	507.5317	0.6	9809
58	508.5432	0.6	9282
59	511.7783	0.7	10013
60	524.7684	0.7	11024
61	526.4304	0.3	5151
62	528.5106	0.6	9642

Bruker Compass DataAnalysis 4.0 Acquisition Date 13.05.2020 13:56:17 Page 2 of 3

Figure S1.27: HRMS (ESI) peak table of 8.

High Resolution Mass Spectrometry Report

#	m/z	I %	I
63	533.7604	0.7	10352
64	546.7502	0.7	10027
65	548.5028	0.8	12128
66	553.4580	0.6	8679
67	565.6023	0.7	10513
68	568.7322	0.4	6481
69	590.7141	0.5	6830
70	618.7382	0.4	6338
71	638.1182	0.4	5333
72	644.7183	0.3	4991
73	666.7002	0.4	5658
74	675.6920	0.4	6379
75	683.6001	0.7	10738
76	684.1059	1.4	20605
77	684.6032	0.3	5255
78	685.1089	0.5	7643
79	686.1036	0.3	5064
80	699.5951	3.3	49607
81	700.6264	6.0	90016
82	701.4923	0.4	5386
83	701.6296	2.9	43263
84	702.6306	0.8	12518
85	705.5823	7.2	108150
86	706.5853	3.4	51013
87	707.5866	1.1	16160
88	721.5768	3.5	53553
89	722.5798	1.6	24824
90	723.5799	0.5	7567
91	742.6737	0.8	12608
92	743.6761	0.5	6960
93	758.6683	1.3	19724
94	759.6714	0.6	9123
95	760.6713	0.5	6982
96	936.8527	0.4	5844
97	942.8473	0.4	6303
98	963.8752	0.6	9269
99	965.8706	1.3	19713
100	966.8726	0.5	7240

Acquisition Parameter

**General**	Fore Vacuum<br>Scan Begin	2.38e+000 mBar<br>75 m/z	High Vacuum<br>Scan End	1.14e-007 mBar<br>2000 m/z	Source Type<br>Ion Polarity	ESI<br>Positive
**Source**	Set Nebulizer<br>Set Dry Heater	2.0 Bar<br>200 °C	Set Capillary<br>Set End Plate Offset	4500 V<br>-500 V	Set Dry Gas	8.0 l/min
**Quadrupole**	Set Ion Energy (MS only)	4.0 eV				
**Coll. Cell**	Collision Energy	8.0 eV	Set Collision Cell RF	600.0 Vpp		
**Ion Cooler**	Set Ion Cooler Transfer Time	75.0 µs	Set Ion Cooler Pre Pulse Storage Time	10.0 µs		

Bruker Compass DataAnalysis 4.0 Acquisition Date 13.05.2020 13:56:17 Page 3 of 3

Figure S1.28: HRMS (ESI) peak table of 8.

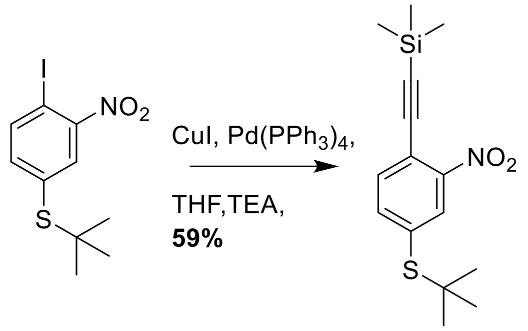

6 11

((4-(*tert*-butylthio)-2-nitrophenyl)ethynyl)trimethylsilane 11: An oven dried Schlenk tube was charged with iodine 6 (300 mg, 890 µmol, 1 eq.), CuI (17.0 mg, 89.0 µmol, 0.1 eq.) $Pd(PPh_3)_4$ (51.4 mg, 44.5 µmol, 0.05 eq.) the solids were degassed for 20 min and dissolved in a degassed mixture of dry THF(2.4 mL), TEA(1.2 mL) and TMS acetylene (0.14 mL, 0.979 mmol, 1.1 eq.). The mixture was stirred at r.t. for 15 h and poured into aq. sat. NH_4Cl and extracted with DCM. The combined organic phase was dried over anhydrous $MgSO_4$, filtered, concentrated under reduced pressure and purified by column chromatography on silica gel (cyclohexane : toluene 3:2) yielding a yellowish oil (170 mg, 553 µmol, 59%)

^{1}H-NMR (500 MHz, $CDCl_3$) δ 8.14 (d, J = 1.8 Hz, 1H), 7.67 (dd, J = 8.0, 1.8 Hz, 1H), 7.58 (d, J = 8.1 Hz, 1H), 1.31 (s, 9H), 0.28 (s, 9H).

^{13}C-NMR (126 MHz, $CDCl_3$) δ 150.18, 141.17, 135.72, 135.12, 132.55, 118.73, 105.85, 99.35, 48.01, 31.38, -0.00.

HRMS (ESI) m/z: calcd. for $[C_{15}H_{21}NO_2SSi+Na]^+$ 330.0952 $[M+Na]^+$; found 330.0954

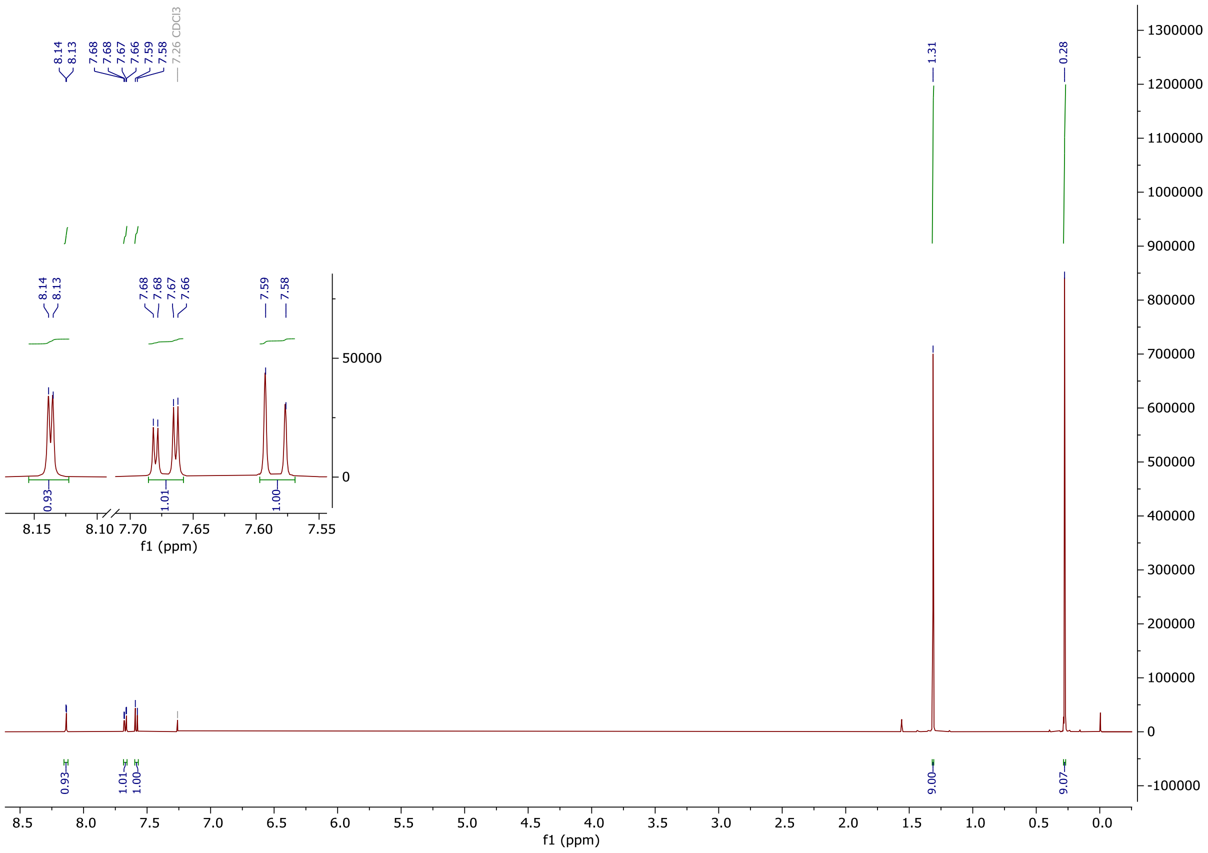

Figure S1.29: ^{1}H-NMR (500 MHz, $CDCl_3$) spectrum of 11.

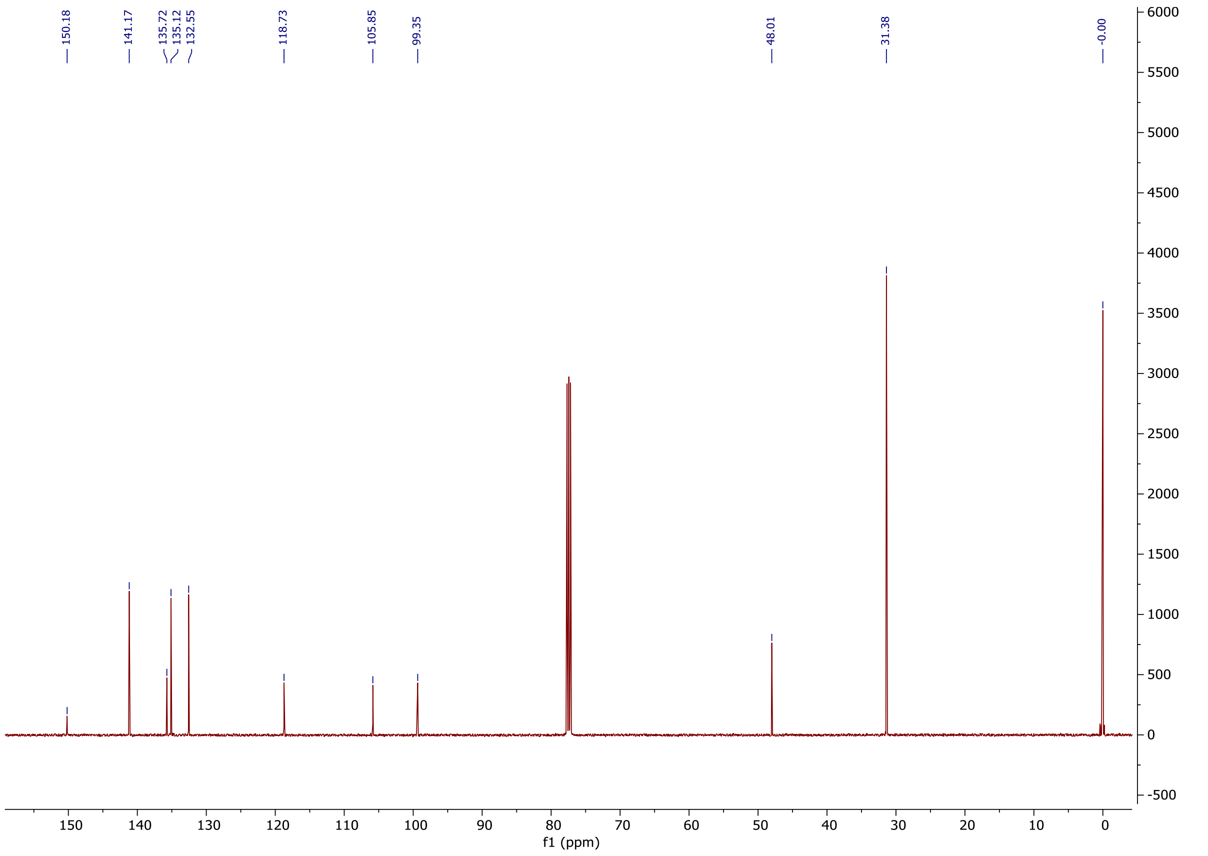

Figure S1.30: ^{13}C-NMR (126 MHz, $CDCl_3$) spectrum of 11.

High Resolution Mass Spectrometry Report

Sample Name	**David Vogel / VOE_282**	Instrument	maXis 4G
Comment	10 ug / mL in MeCN, analyzed in MeOH	Method	22 Direct_pos_mid.m

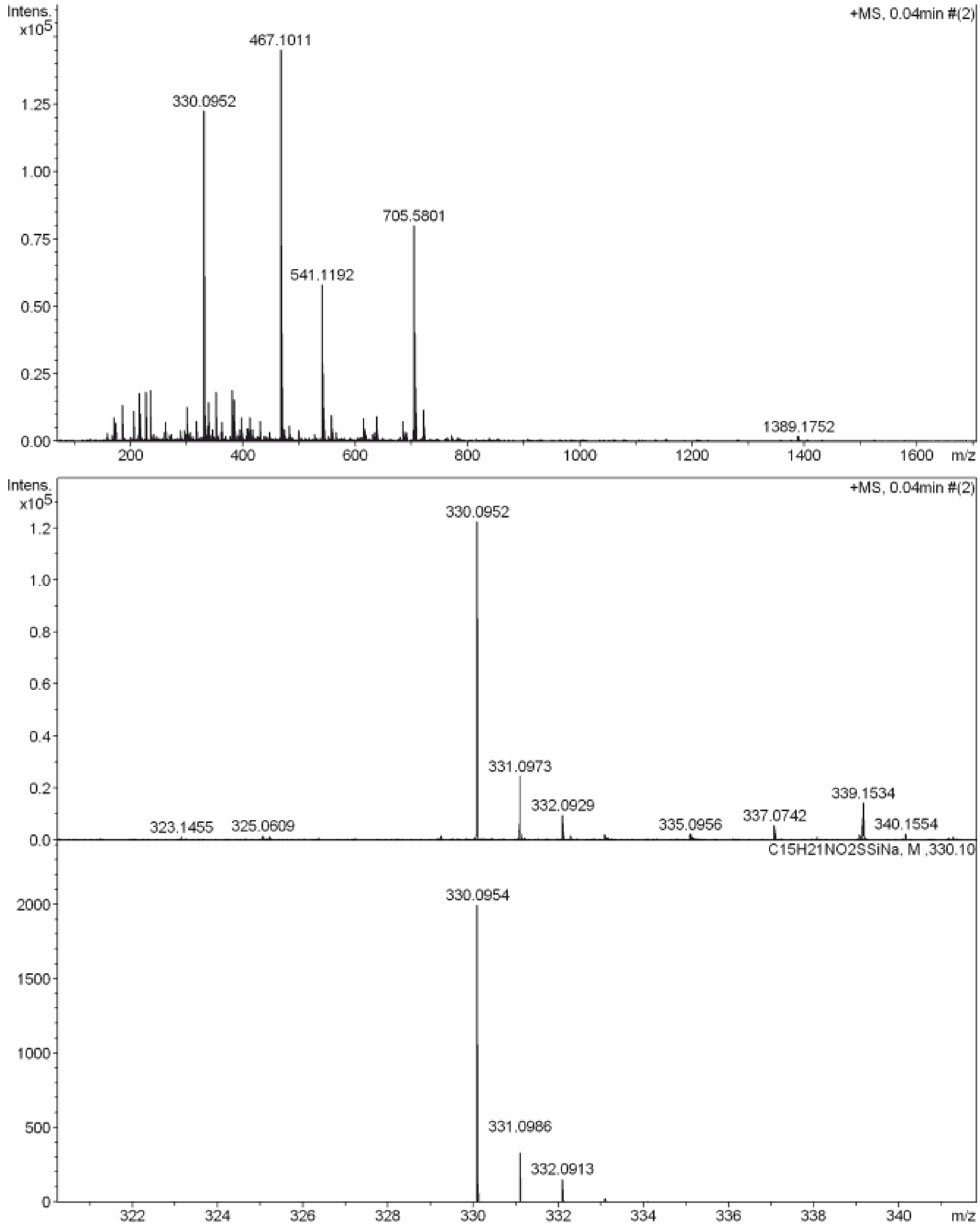

Bruker Compass DataAnalysis 4.0 | Acquisition Date 16.08.2019 09:48:05 | Page 1 of 3

Figure S1.31: HRMS (ESI) spectrum of 11.

High Resolution Mass Spectrometry Report

Measured m/z vs. theoretical m/z

Meas. m/z	#	Formula	Score	m/z	err [mDa]	err [ppm]	mSigma	rdb	e⁻ Conf	z
330.0952	1	C 15 H 21 N Na O 2 S Si	100.00	330.0954	0.3	0.8	16.7	6.5	even	1+

Mass list

#	m/z	I %	I
1	158.9647	2.2	3250
2	167.1042	1.7	2532
3	171.0996	6.3	9148
4	173.0789	4.7	6861
5	185.1151	9.3	13490
6	205.0601	8.0	11561
7	213.1464	1.8	2557
8	215.1257	12.5	18196
9	217.1044	7.1	10338
10	226.9517	12.6	18345
11	227.1256	6.7	9678
12	236.0718	13.2	19182
13	240.9674	2.0	2897
14	241.0678	1.8	2545
15	261.1309	2.5	3581
16	263.0560	4.6	6701
17	269.1357	1.7	2530
18	273.1682	2.0	2890
19	288.2892	2.9	4216
20	294.9392	3.0	4408
21	299.1608	2.0	2976
22	301.0747	8.9	12936
23	301.1410	4.9	7188
24	301.2110	3.4	4990
25	302.0641	2.0	2897
26	302.0768	1.8	2670
27	305.1566	2.4	3529
28	317.1717	5.4	7842
29	330.0952	84.4	122658
30	331.0973	17.1	24793
31	332.0929	6.9	9960
32	337.0742	4.1	5981
33	339.1534	10.0	14554
34	340.1554	1.8	2669
35	344.1110	1.8	2665
36	346.0685	3.0	4420
37	353.2656	12.7	18406
38	354.2687	2.3	3320
39	360.3229	2.4	3540
40	362.9254	5.1	7346
41	381.2966	13.2	19168
42	382.2998	3.5	5138
43	384.1416	10.9	15804
44	385.1432	2.9	4221
45	391.2089	1.8	2673
46	395.0613	3.1	4506
47	398.1571	6.2	8976
48	399.1596	2.2	3149
49	399.3074	1.8	2626
50	407.1407	3.4	4917
51	409.1127	3.5	5076
52	410.1143	1.7	2514
53	411.0929	2.4	3520
54	412.1732	3.8	5462
55	413.2650	6.2	8949
56	414.2692	1.9	2730
57	417.3438	3.1	4462
58	430.9131	5.3	7724
59	447.3438	2.5	3677
60	467.1011	100.0	145306
61	468.1018	40.0	58082
62	469.0988	27.8	40324

Figure S1.32: HRMS (ESI) peak table of 11.

High Resolution Mass Spectrometry Report

#	m/z	I %	I
63	470.0986	8.4	12242
64	471.0957	3.8	5456
65	475.1264	3.2	4600
66	483.0750	4.0	5746
67	483.1314	1.7	2520
68	484.0746	1.9	2804
69	498.8990	2.9	4245
70	528.5100	1.9	2801
71	541.1192	40.2	58397
72	542.1198	17.5	25440
73	543.1172	15.8	22886
74	544.1169	5.8	8442
75	545.1144	2.7	3918
76	557.0930	6.7	9670
77	558.0931	4.0	5806
78	559.0908	2.4	3532
79	566.8873	2.5	3636
80	615.1373	6.0	8788
81	616.1398	3.3	4822
82	617.1364	2.9	4236
83	619.5243	1.8	2632
84	631.1131	2.1	2980
85	634.8741	2.1	3029
86	637.1985	6.6	9530
87	638.2027	2.9	4177
88	639.1982	2.1	3020
89	685.4336	5.4	7864
90	686.4357	2.7	3890
91	689.1551	1.9	2709
92	691.2466	2.4	3512
93	702.8587	3.0	4304
94	705.2604	2.5	3598
95	705.5801	55.2	80229
96	706.5829	27.5	39913
97	707.5840	7.4	10762
98	708.5823	2.2	3149
99	721.5738	8.2	11976
100	722.5784	3.6	5280

Acquisition Parameter

**General**	Fore Vacuum<br>Scan Begin	2.68e+000 mBar<br>75 m/z	High Vacuum<br>Scan End	1.21e-007 mBar<br>1700 m/z	Source Type<br>Ion Polarity	ESI<br>Positive
**Source**	Set Nebulizer<br>Set Dry Heater	0.4 Bar<br>180 °C	Set Capillary<br>Set End Plate Offset	3600 V<br>-500 V	Set Dry Gas	4.0 l/min
**Quadrupole**	Set Ion Energy (MS only)	4.0 eV				
**Coll. Cell**	Collision Energy	8.0 eV	Set Collision Cell RF	350.0 Vpp		
**Ion Cooler**	Set Ion Cooler Transfer Time	75.0 µs	Set Ion Cooler Pre Pulse Storage Time	10.0 µs		

Bruker Compass DataAnalysis 4.0 Acquisition Date 16.08.2019 09:48:05 Page 3 of 3

Figure S1.33: HRMS (ESI) peak table of 11.

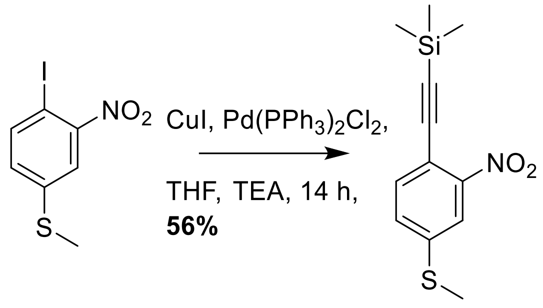

5 10

Trimethyl((4-(methylthio)-2-nitrophenyl)ethynyl)silane 10: An oven dried argon flushed Schlenk tube was charged with iodine 5 (200 mg, 0.678 mmol, 1 eq.), purged with argon and the solid was dissolved in a degassed mixture of THF (1.6 mL) and TEA (0.8 mL). Then CuI (13.0 mg, 67.8 µmol, 0.1 eq.), $Pd(PPh_3)_2Cl_2$ (39 mg, 33.9 µmol, 0.05 eq.) and TMS acetylene (0.15 mL, 1.02 mmol, 1.5 eq.) was added and the solution was stirred at r.t. for 14 h, thereafter poured into aq. sat. NH_4Cl and extracted with DCM. The combined organic phase was dried over anhydrous $MgSO_4$, filtered, concentrated under reduced pressure and purified by column chromatography on silica gel (DCM) yielding a yellowish oil (101 mg, 381 µmol, 56%).

^{1}H-NMR (500 MHz, $CDCl_3$) δ 7.79 (d, J = 2.0 Hz, 1H), 7.52 (d, J = 8.2 Hz, 1H), 7.35 (dd, J = 8.3, 2.0 Hz, 1H), 2.54 (s, 3H), 0.27 (s,9H).

^{13}C-NMR (126 MHz, $CDCl_3$) δ 150.57, 141.96, 135.05, 129.64, 120.75, 114.29, 103.38, 99.45, 15.29, -0.19.

HRMS (ESI) m/z: calcd. for $[C_{12}H_{15}NO_2SSi+Na]^+$ 288.0480 $[M+Na]^+$; found 288.0485
$[C_{12}H_{15}NO_2SSi+K]^+$ 304.0223 $[M+K]^+$; found 304.0224

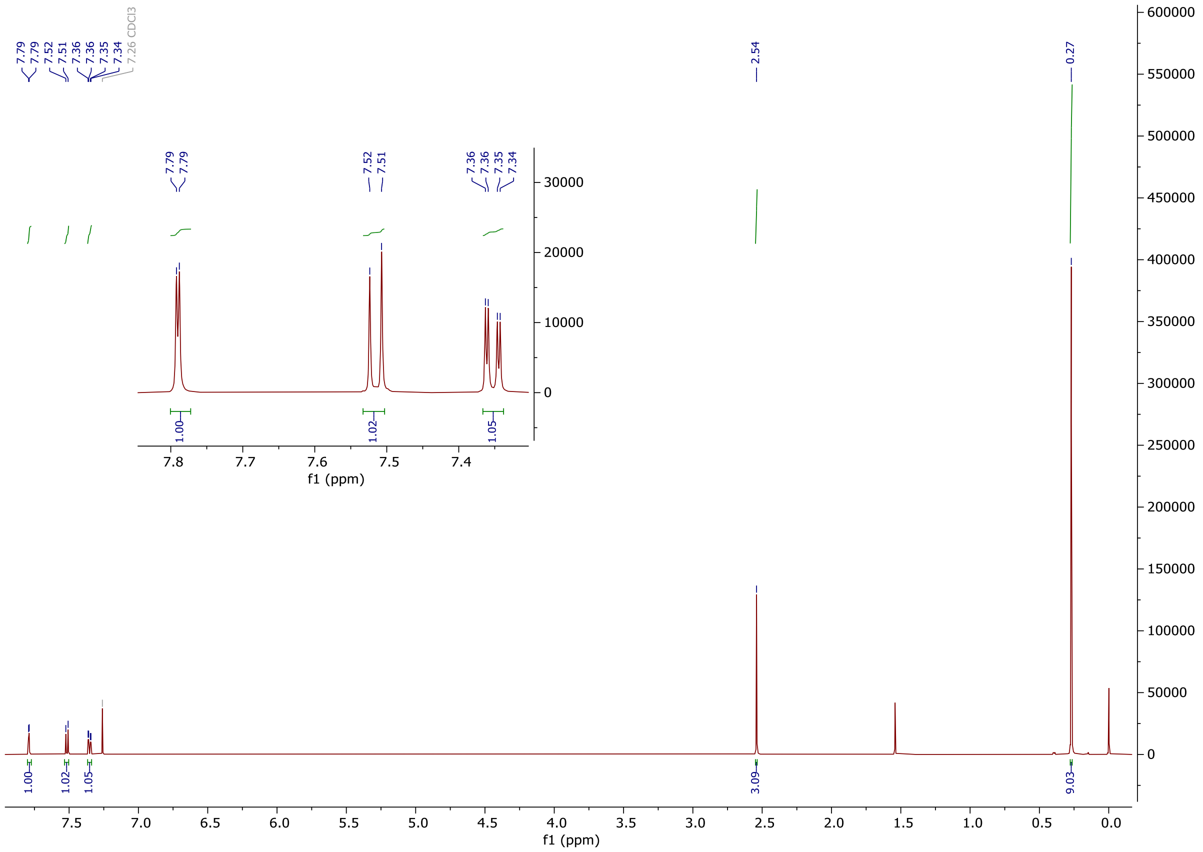

Figure S1.34: ^{1}H-NMR (500 MHz, $CDCl_3$) spectrum of 10.

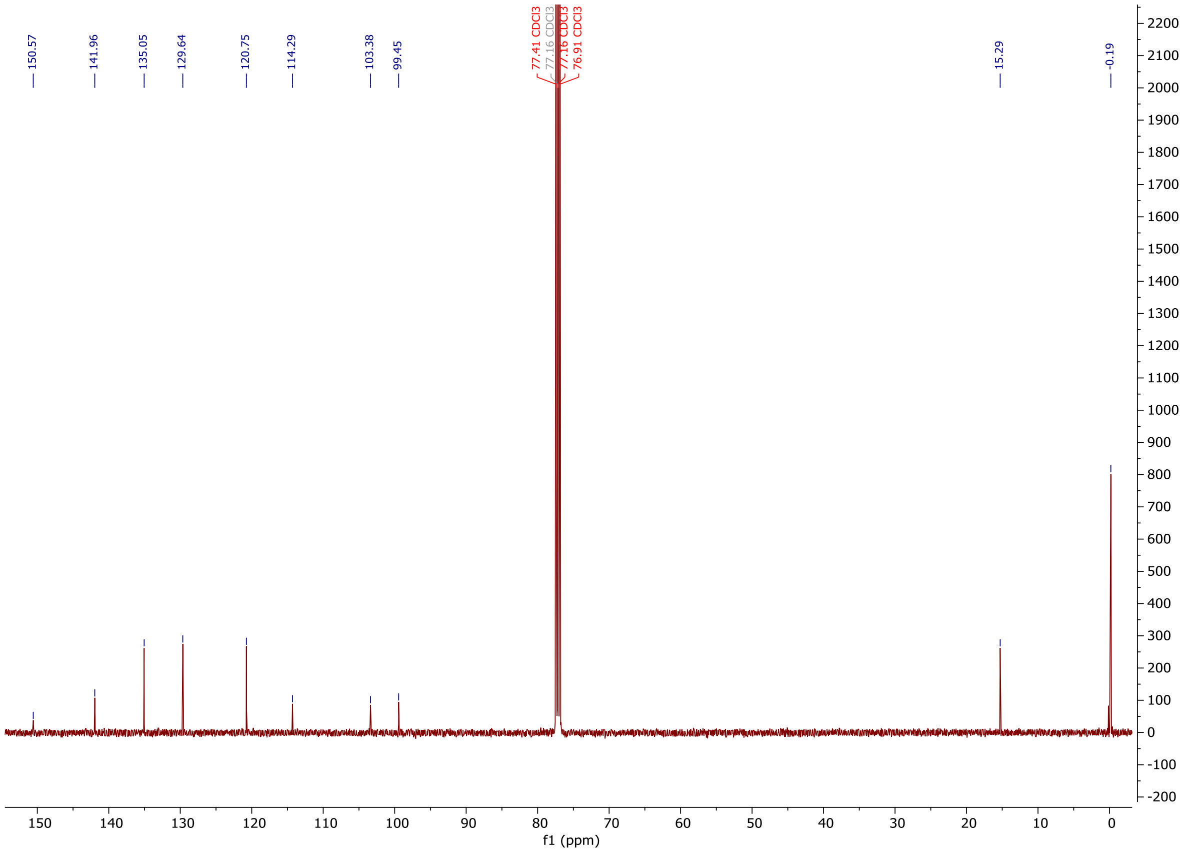

Figure S1.35: ^{13}C-NMR (126 MHz, $CDCl_3$) spectrum of 10.

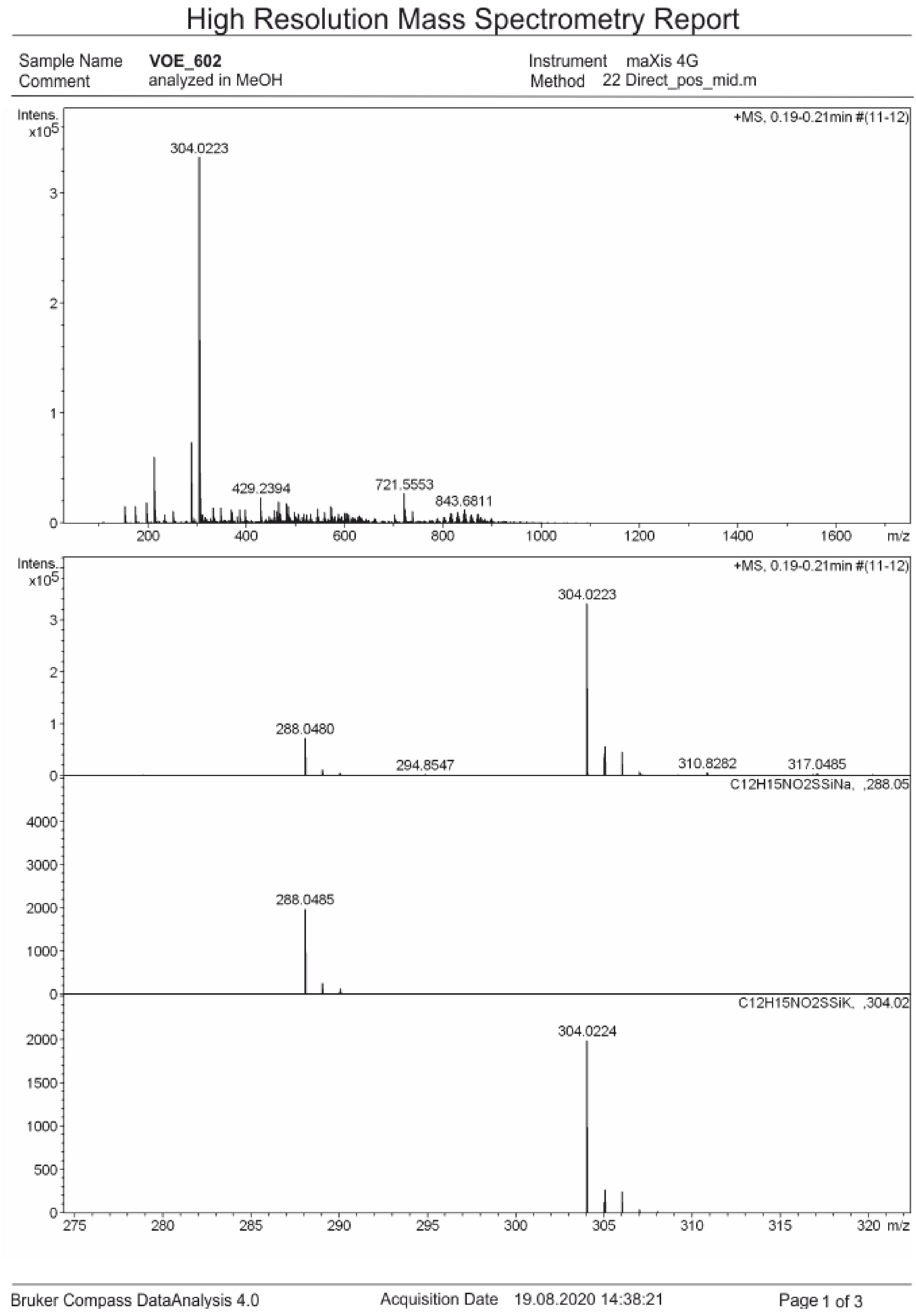

Figure S1.36: HRMS (ESI) spectrum of 10.

High Resolution Mass Spectrometry Report

Measured m/z vs. theoretical m/z

Meas. m/z	#	Formula	Score	m/z	err [mDa]	err [ppm]	mSigma	rdb	e⁻ Conf	z
288.0480	1	C 12 H 15 N Na O 2 S Si	100.00	288.0485	0.5	1.7	16.3	6.5	even	1+
304.0223	1	C 12 H 15 K N O 2 S Si	100.00	304.0224	0.1	0.4	18.9	6.5	even	

Mass list

#	m/z	I %	I
1	152.9354	4.7	15773
2	174.8960	4.7	15674
3	196.8777	5.8	19223
4	212.8515	18.2	60466
5	214.8497	3.9	12933
6	234.8333	2.5	8420
7	250.8074	3.4	11193
8	288.0480	22.2	73905
9	289.0500	3.8	12706
10	290.0446	1.7	5784
11	304.0223	100.0	332979
12	305.0242	17.3	57528
13	306.0197	13.9	46280
14	307.0213	2.6	8661
15	310.8282	2.3	7803
16	317.0485	1.7	5760
17	326.8238	1.6	5215
18	332.8104	4.2	13914
19	335.1975	1.6	5166
20	348.7844	4.3	14163
21	369.2393	3.7	12477
22	370.7658	3.1	10209
23	381.1086	1.5	4995
24	381.2502	1.9	6193
25	386.7400	3.8	12515
26	397.2707	3.7	12386
27	429.2394	7.2	23883
28	430.2425	1.8	5882
29	446.7609	1.9	6348
30	457.2706	3.5	11694
31	463.3176	3.3	11098
32	465.3477	6.0	19933
33	466.3511	2.1	6889
34	468.7430	2.7	8956
35	481.3435	5.6	18612
36	482.3471	1.8	5848
37	484.7187	2.8	9238
38	484.7339	2.3	7598
39	485.3017	4.9	16220
40	486.3052	1.6	5188
41	489.4059	1.7	5780
42	497.3381	3.0	10091
43	500.7121	1.9	6320
44	506.6992	2.6	8626
45	513.3333	1.8	5915
46	517.4372	2.7	9124
47	522.6740	2.5	8229
48	531.4527	2.8	9203
49	543.4528	1.8	5887
50	545.4680	4.1	13626
51	546.4718	1.6	5285
52	547.4818	1.7	5570
53	559.4835	3.1	10444
54	571.4842	4.7	15789
55	572.4875	1.9	6249
56	573.4990	4.2	13974
57	574.5028	1.7	5583
58	575.5130	1.7	5517
59	577.4213	1.5	5009
60	579.4379	2.0	6552
61	585.4989	1.6	5205

Bruker Compass DataAnalysis 4.0 Acquisition Date 19.08.2020 14:38:21 Page 2 of 3

Figure S1.37: HRMS (ESI) peak table of 10.

High Resolution Mass Spectrometry Report

#	m/z	I %	I
62	587.5139	2.7	8927
63	593.4530	1.9	6227
64	599.5153	2.9	9577
65	601.5295	2.9	9797
66	605.4529	3.0	9896
67	607.4679	2.5	8308
68	615.5457	1.8	6015
69	619.4683	1.6	5206
70	620.6686	1.5	5156
71	627.5460	1.7	5679
72	629.5614	2.2	7272
73	633.4849	1.8	5899
74	661.5289	1.6	5217
75	701.4079	2.4	8150
76	721.5553	8.3	27481
77	722.5580	3.9	12994
78	723.5566	2.0	6708
79	737.5491	3.4	11384
80	789.6341	1.5	5012
81	801.6348	1.7	5496
82	803.6500	1.8	5919
83	813.6350	1.6	5168
84	815.6499	2.9	9779
85	816.6530	1.5	5058
86	817.6645	2.6	8657
87	827.6508	1.7	5770
88	829.6656	3.0	10036
89	831.6796	2.3	7588
90	841.6663	2.7	9076
91	842.6690	1.5	5134
92	843.6811	3.7	12470
93	844.6846	2.2	7226
94	845.6944	2.6	8607
95	855.6809	1.9	6237
96	857.6971	2.4	8122
97	869.6959	2.5	8238
98	870.7015	1.7	5651
99	871.7118	2.7	8910
100	877.4848	1.7	5642

Acquisition Parameter

**General**	Fore Vacuum<br>Scan Begin	2.48e+000 mBar<br>75 m/z	High Vacuum<br>Scan End	1.40e-007 mBar<br>1700 m/z	Source Type<br>Ion Polarity	ESI<br>Positive
**Source**	Set Nebulizer<br>Set Dry Heater	0.4 Bar<br>180 °C	Set Capillary<br>Set End Plate Offset	3600 V<br>-500 V	Set Dry Gas	4.0 l/min
**Quadrupole**	Set Ion Energy (MS only)	4.0 eV				
**Coll. Cell**	Collision Energy	8.0 eV	Set Collision Cell RF	350.0 Vpp	100.0 Vpp	
**Ion Cooler**	Set Ion Cooler Transfer Time	75.0 µs	Set Ion Cooler Pre Pulse Storage Time	10.0 µs		

Bruker Compass DataAnalysis 4.0 Acquisition Date 19.08.2020 14:38:21 Page 3 of 3

Figure S1.38: HRMS (ESI) peak table of 10.

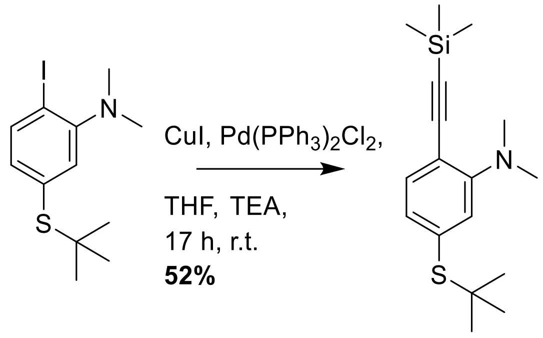

9 13

5-(*tert*-butylthio)-*N,N*-dimethyl-2-((trimethylsilyl)ethynyl)aniline 13: An oven dried argon flushed Schlenk tube was charged with iodine 9 (50 mg, 149 µmol, 1 eq.) and purged with argon. Then degassed THF (1 mL) and degassed TEA (0.5 mL), CuI (2.84 mg, 14.9 µmol, 0.1 eq.), $Pd(PPh_3)_2Cl_2$ (5.23 mg, 7.45 µmol, 0.05 eq.) and TMS acetylene (0.02 mL, 164 µmol, 1.1 eq.) were added and the solution was stirred for 17 h at r.t. The reaction mixture was poured into aq. sat. NH_4Cl and extracted with DCM. The combined organic phase was dried anhydrous Na_2SO_4, filtered, concentrated under reduced pressure and purified by column chromatography on silica gel (DCM) yielding 13 as a yellowish oil (23.5 mg, 77.0 µmol, 52%).

^{1}H-NMR (500 MHz, $CDCl_3$) δ 7.35 (d, J = 7.8 Hz, 1H), 7.01 (d, J = 1.6 Hz, 1H), 6.99 – 6.97 (m, 1H), 2.95 (s, 6H), 1.29 (s, 9H), 0.25 (s, 9H).

^{13}C-NMR (126 MHz, $CDCl_3$) 154.82, 134.57, 134.24, 128.72, 125.68, 114.94, 104.21, 101.22, 46.44, 43.34, 31.14, -0.00.

HRMS (ESI) m/z: calcd. for $[C_{17}H_{27}NSSi+H]^+$ 306.1710 $[M+H]^+$; found 306.1706

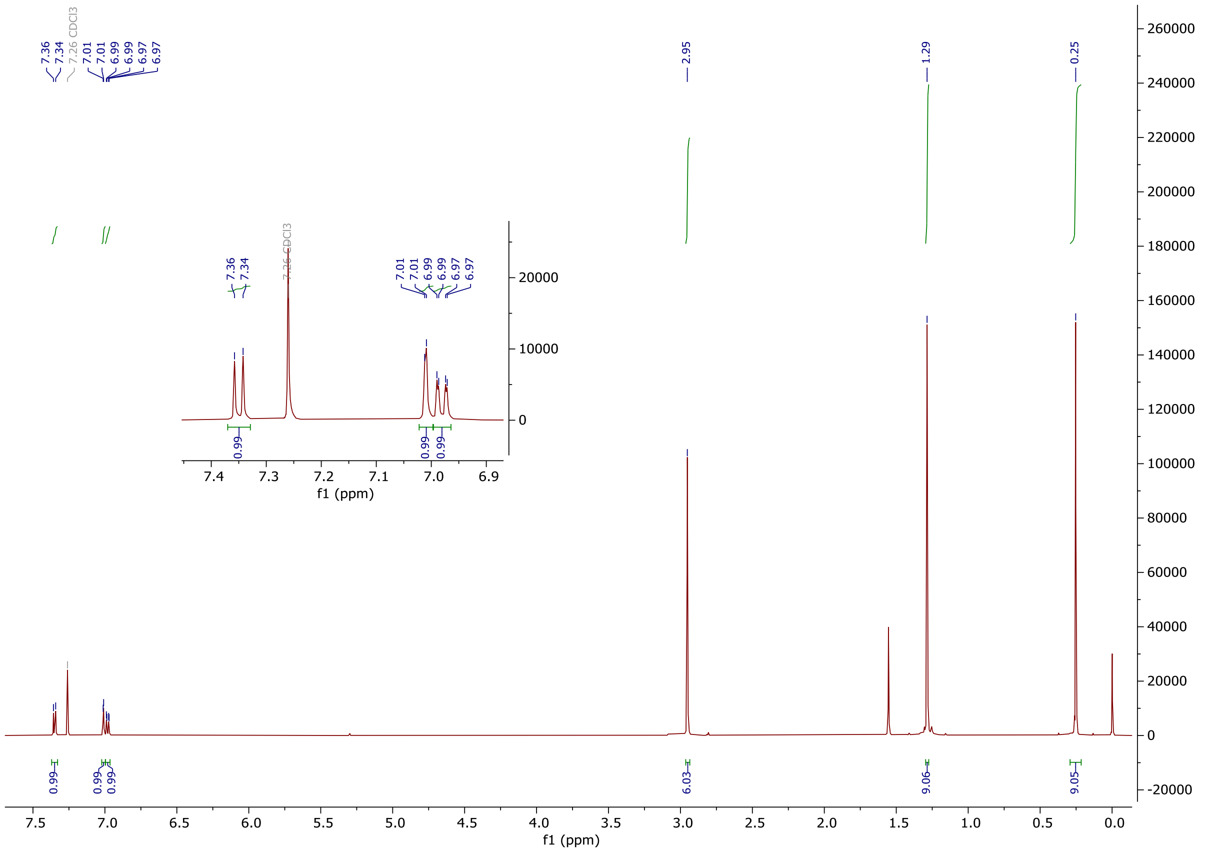

Figure S1.39: ^{1}H-NMR (500 MHz, $CDCl_3$) spectrum of 13.

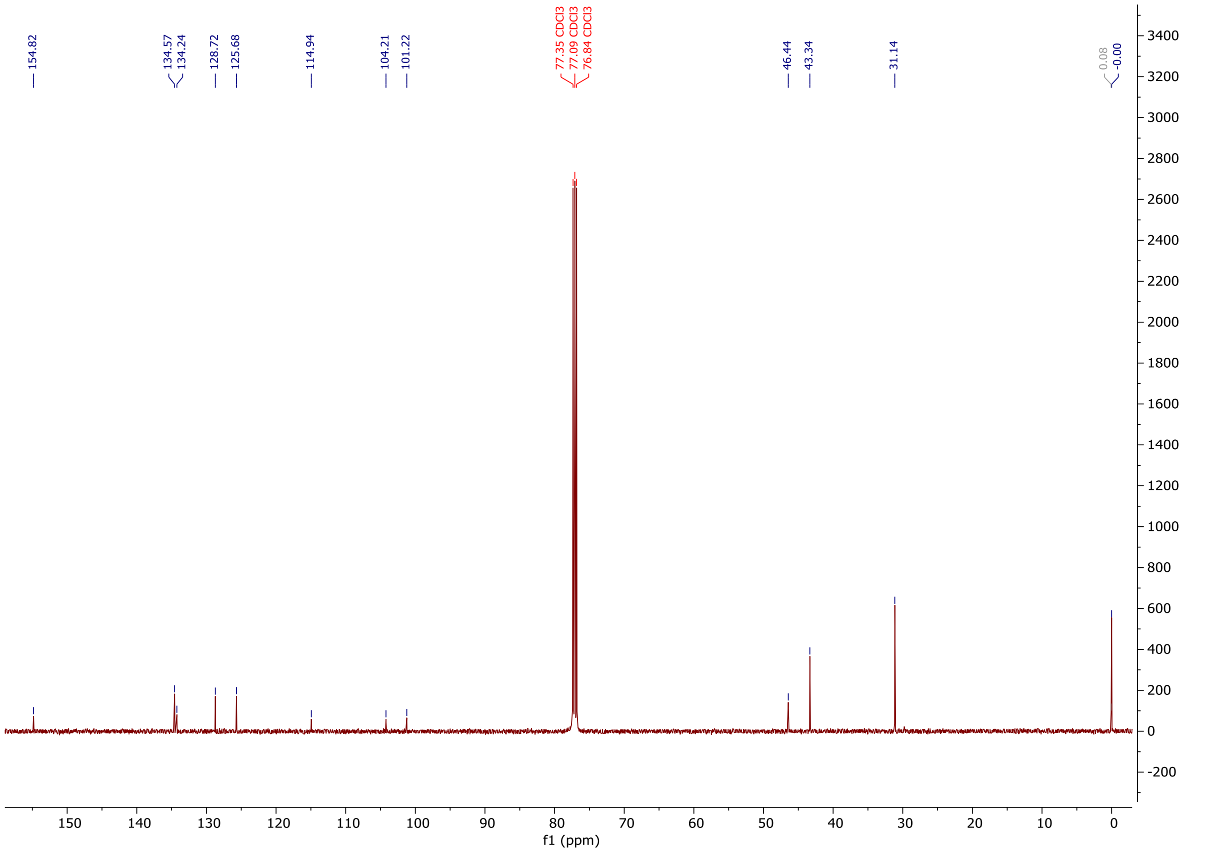

Figure S1.40: ^{13}C-NMR (126 MHz, $CDCl_3$) spectrum of 13.

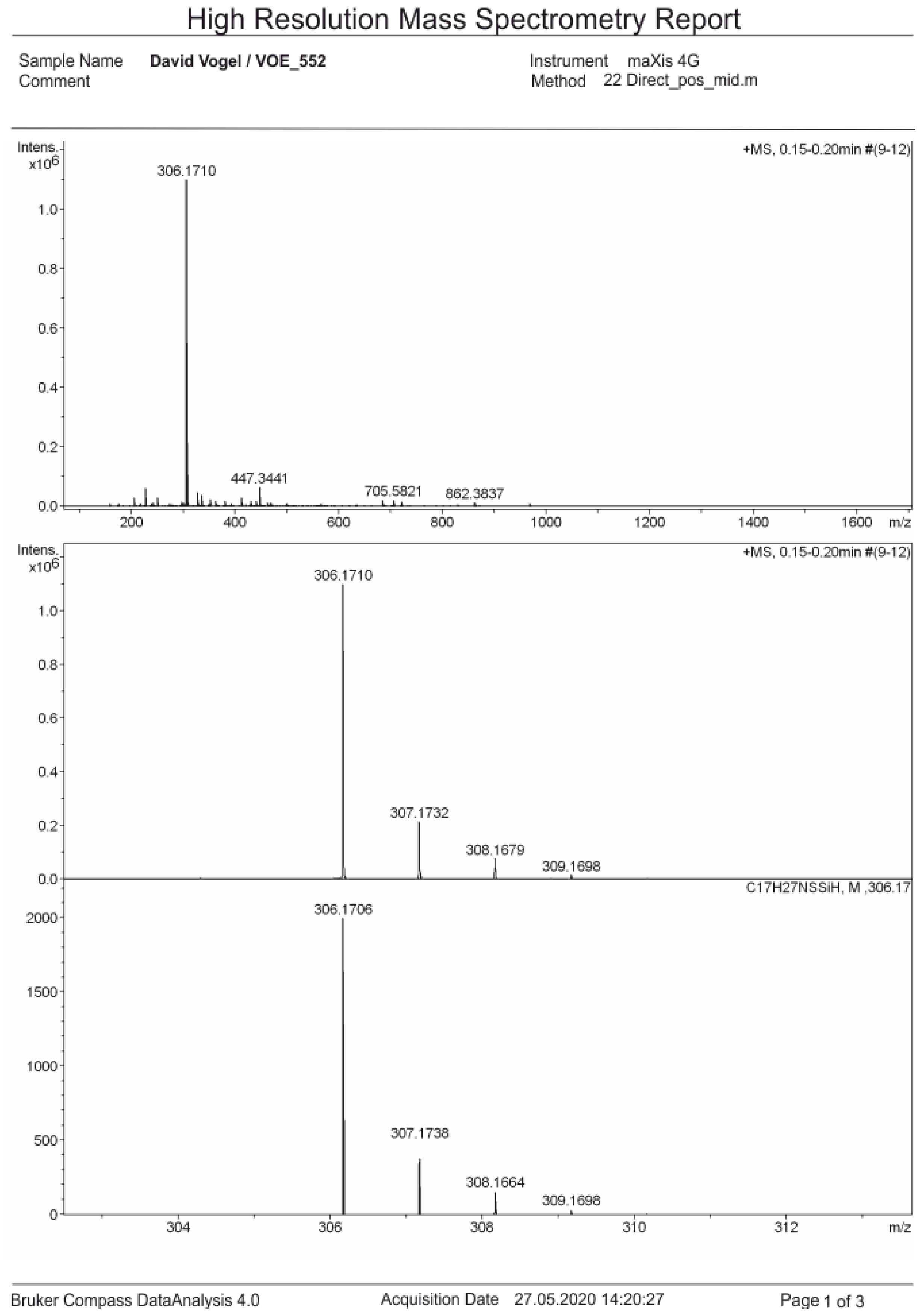

Figure S1.41: HRMS (ESI) spectrum of 13.

High Resolution Mass Spectrometry Report

Measured m/z vs. theoretical m/z

Meas. m/z	#	Formula	Score	m/z	err [mDa]	err [ppm]	mSigma	rdb	e⁻ Conf	z
306.1710	1	C 17 H 28 N S Si	100.00	306.1706	-0.4	-1.4	31.7	5.5	even	1+

Mass list

#	m/z	I %	I
1	158.9644	0.8	8831
2	176.0527	1.0	10869
3	205.0600	2.7	29312
4	211.0934	0.4	4851
5	217.1044	0.8	8751
6	218.9361	0.6	6451
7	223.0937	0.4	4179
8	226.9514	5.8	63411
9	227.1255	0.4	4609
10	229.1409	0.5	5275
11	236.0710	0.4	4218
12	239.0886	1.0	10495
13	242.9251	1.0	11537
14	245.0778	0.4	4831
15	250.1079	2.9	31536
16	251.1097	0.5	5589
17	261.1291	0.4	4629
18	273.1671	0.8	9071
19	276.9338	0.5	5093
20	279.2289	0.6	6506
21	288.9217	0.4	4850
22	293.2082	0.4	4605
23	294.9386	0.8	8313
24	298.1655	1.4	15489
25	299.1632	0.7	8099
26	301.1408	1.1	12627
27	301.2108	0.8	8469
28	304.2990	0.6	7151
29	306.1710	100.0	1101223
30	307.1732	19.5	214797
31	307.2597	0.4	4221
32	308.1679	7.0	76651
33	309.1698	1.5	16533
34	328.1522	4.4	49003
35	329.1544	1.1	12331
36	330.1496	0.5	5082
37	331.2083	0.6	6399
38	332.3309	0.5	5378
39	336.0276	3.6	40011
40	337.0301	0.6	7062
41	348.9896	0.7	7944
42	350.9872	0.8	8354
43	353.2657	2.1	23534
44	354.2692	0.5	5823
45	362.9263	1.7	18332
46	365.1047	0.5	5450
47	365.1355	0.5	6001
48	365.2759	0.6	7000
49	369.1980	0.4	4527
50	381.2971	1.7	18938
51	382.3001	0.4	4325
52	393.2988	1.0	11069
53	407.3134	0.5	5323
54	413.2657	2.7	29984
55	414.2688	0.9	9364
56	421.3302	0.6	6965
57	429.2393	0.6	6515
58	430.9136	1.7	18336
59	435.3441	0.5	5394
60	437.1856	0.5	5101
61	441.2970	1.7	18662
62	442.3002	0.5	5319

Bruker Compass DataAnalysis 4.0 Acquisition Date 27.05.2020 14:20:27 Page 2 of 3

Figure S1.42: HRMS (ESI) peak table of 13.

High Resolution Mass Spectrometry Report

#	m/z	I %	I
63	447.3441	6.0	66272
64	448.3471	1.7	18987
65	449.3594	0.8	8352
66	455.3127	0.5	5398
67	457.2713	0.5	4978
68	463.3178	1.3	13918
69	463.3744	0.5	5003
70	464.3213	0.5	5045
71	467.1015	0.8	8749
72	469.3277	1.1	12539
73	470.3317	0.4	4247
74	473.4684	0.6	7075
75	498.9004	0.9	9675
76	501.4999	0.7	7600
77	523.3241	0.5	5506
78	529.4943	0.4	4223
79	566.8890	0.9	9520
80	591.4949	0.4	4802
81	634.8753	0.6	6160
82	685.4358	1.8	20198
83	686.4387	0.9	9988
84	701.4084	0.4	4526
85	705.5821	2.0	21602
86	706.5857	1.0	10957
87	721.5751	1.4	15183
88	722.5794	0.6	7110
89	737.5517	0.4	4703
90	801.6921	0.4	4449
91	829.7239	0.5	5606
92	861.3831	0.5	5503
93	861.8853	0.5	5418
94	862.3837	1.2	13202
95	862.8839	1.0	11234
96	863.3837	1.1	11616
97	863.8856	0.8	8998
98	864.3858	0.4	4762
99	968.6167	0.9	9833
100	969.6197	0.7	7289

Acquisition Parameter

**General**	Fore Vacuum<br>Scan Begin	2.61e+000 mBar<br>75 m/z	High Vacuum<br>Scan End	1.14e-007 mBar<br>1700 m/z	Source Type<br>Ion Polarity	ESI<br>Positive
**Source**	Set Nebulizer<br>Set Dry Heater	0.4 Bar<br>180 °C	Set Capillary<br>Set End Plate Offset	3600 V<br>-500 V	Set Dry Gas	4.0 l/min
**Quadrupole**	Set Ion Energy (MS only)	4.0 eV				
**Coll. Cell**	Collision Energy	8.0 eV	Set Collision Cell RF	350.0 Vpp		
**Ion Cooler**	Set Ion Cooler Transfer Time	75.0 µs	Set Ion Cooler Pre Pulse Storage Time	10.0 µs		

Bruker Compass DataAnalysis 4.0 Acquisition Date 27.05.2020 14:20:27 Page 3 of 3

Figure S1.43: HRMS (ESI) peak table of 13.

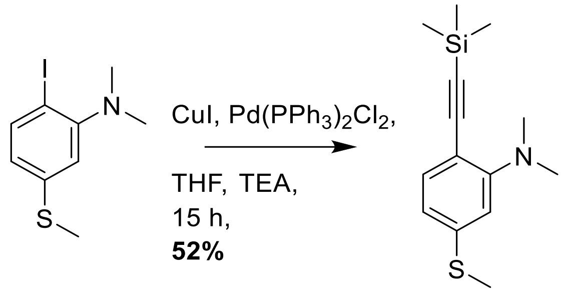

8 12

***N,N*-dimethyl-5-(methylthio)-2-((trimethylsilyl)ethynyl)aniline 12**: An oven dried argon flushed Schlenk tube was charged with iodine 8 (200 mg, 682 µmol, 1 eq.). The solids were purged with argon and dissolved in a degassed mixture of THF (2 mL) and TEA (1 mL). Then CuI (13 mg, 68.3 µmol, 0.1 eq.), $Pd(PPh_3)_2Cl_2$ (24 mg, 34.2 µmol, 0.05 eq.) and TMS acetylene (0.1 mL, 751 µmol, 1.1 eq.) was added. The solution was stirred at r.t. for 15 h, poured into aq. sat. NH_4Cl and extracted with DCM. The combined organic phase was dried over anhydrous $MgSO_4$, filtered, concentrated under reduced pressure and purified by column chromatography on silica gel (DCM : cyclohexane 1 : 2) yielding 12 as a yellowish oil (93.0 mg, 353 µmol, 52%).

^{1}H-NMR (500 MHz, $CDCl_3$) δ 7.32 (d, J = 8.1 Hz, 1H), 6.71 (d, J = 1.8 Hz, 1H), 6.68 (dd, J = 8.1, 1.8 Hz, 1H), 2.94 (s, 6H), 2.47 (s, 3H), 0.24 (s, 9H).

^{13}C-NMR (126 MHz, $CDCl_3$) δ 155.24, 140.48, 135.24, 117.46, 114.56, 111.34, 104.72, 99.59, 43.32, 15.55, 0.14.

HRMS (ESI) m/z: calcd. for $[C_{14}H_{21}NSSi+H]^+$ 264.1239 $[M+H]^+$; found 264.1237

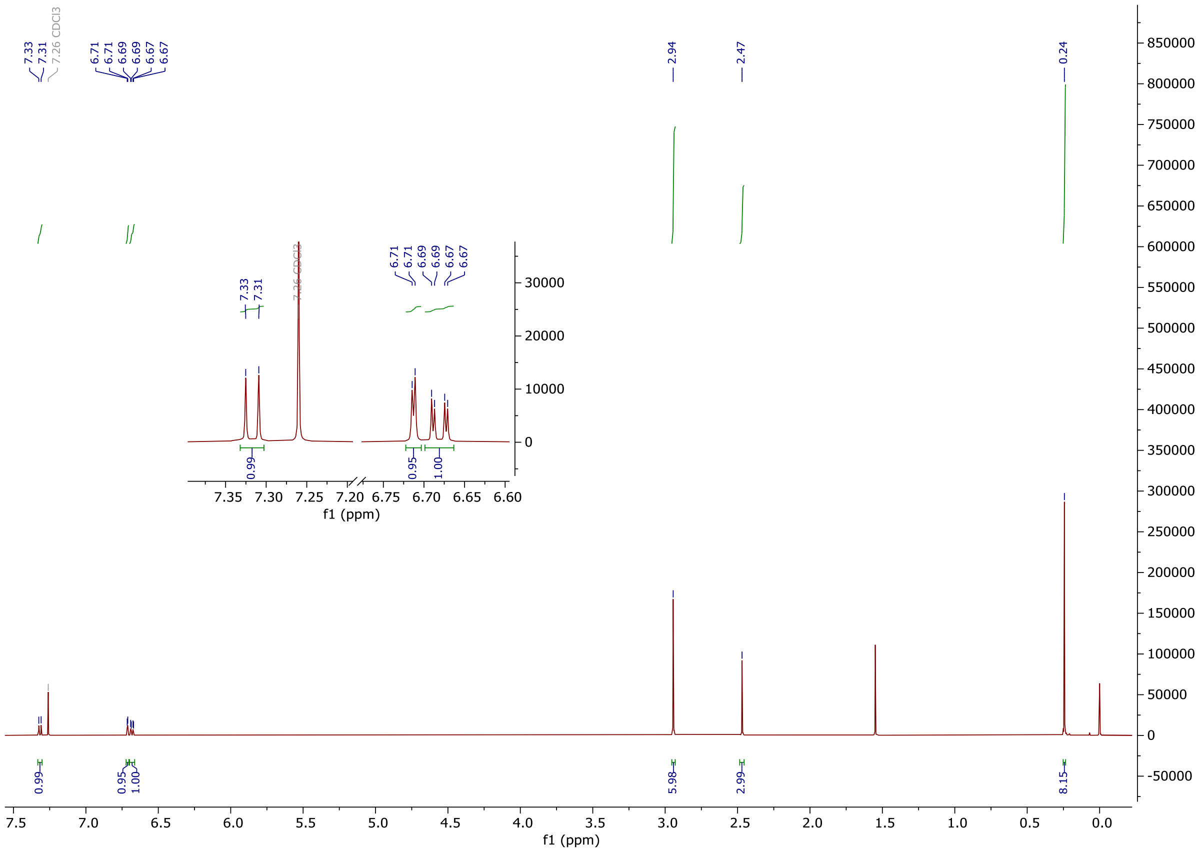

Figure S1.44: ^{1}H-NMR (500 MHz, $CDCl_3$) spectrum of 12.

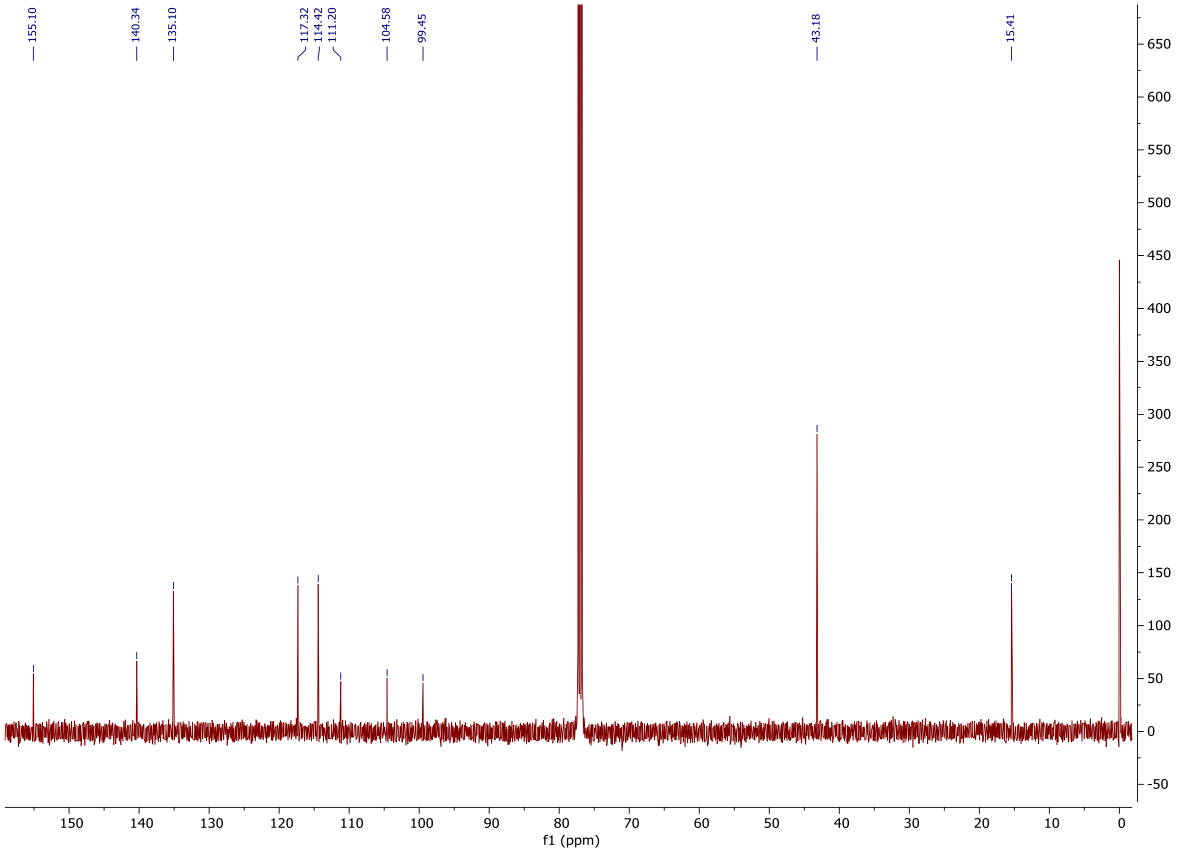

Figure S1.45: ^{13}C-NMR (126 MHz, $CDCl_3$) spectrum of 12.

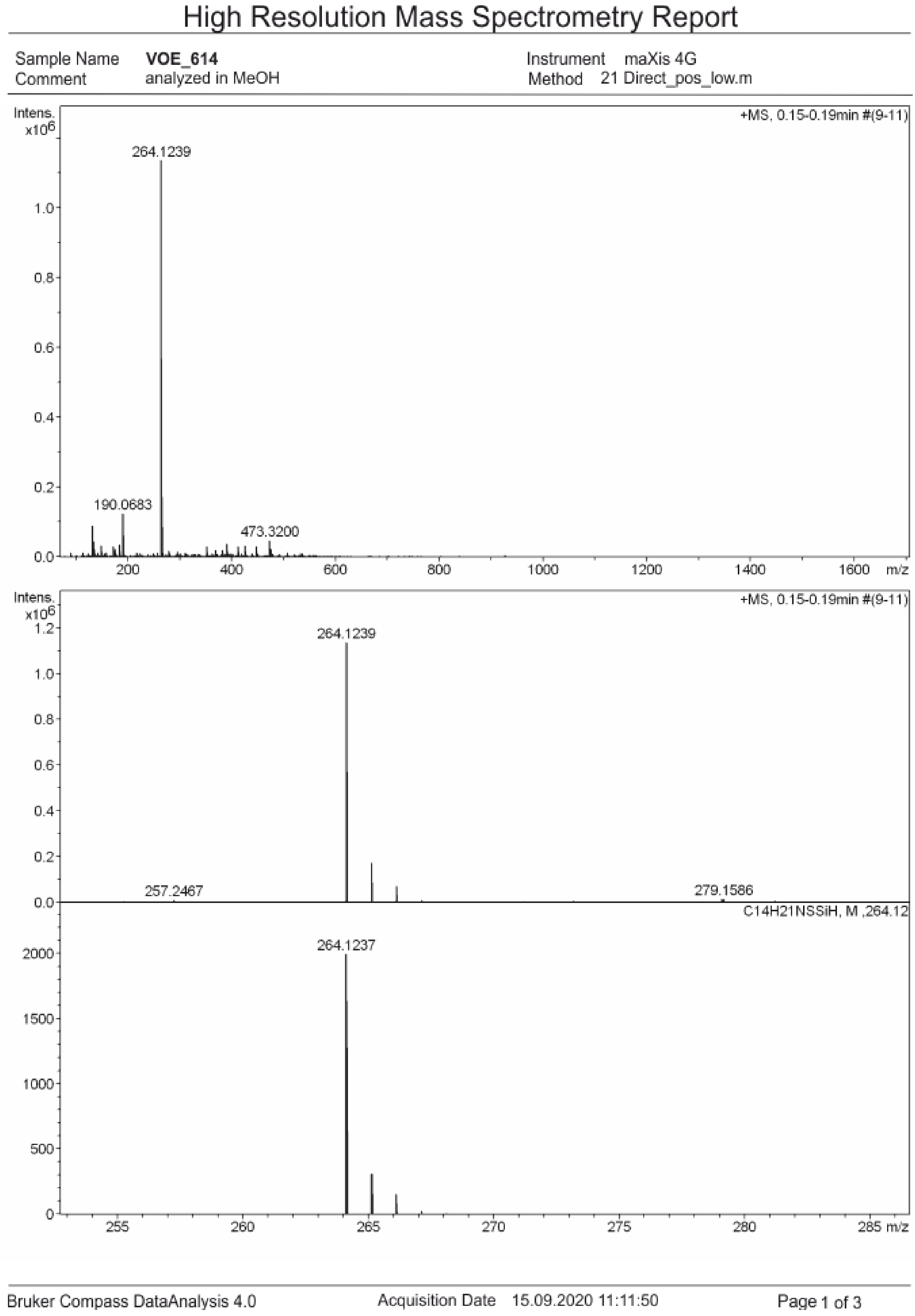

Figure S1.46: HRMS (ESI) spectrum of 12.

High Resolution Mass Spectrometry Report

Measured m/z vs. theoretical m/z

Meas. m/z	#	Formula	Score	m/z	err [mDa]	err [ppm]	mSigma	rdb	e⁻ Conf	z
264.1239	1	C 14 H 22 N S Si	100.00	264.1237	-0.2	-0.7	35.0	5.5	even	1+

Mass list

#	m/z	I %	I
1	89.5079	1.0	11835
2	90.5082	0.6	6463
3	102.1286	0.6	6530
4	113.9641	1.1	12680
5	122.0813	0.6	6700
6	122.9643	0.9	9943
7	131.9300	7.9	89654
8	133.9289	4.0	44998
9	135.9265	1.9	22091
10	141.9588	1.0	11929
11	149.0233	2.9	33339
12	149.9404	1.1	12536
13	149.9532	0.8	9256
14	155.0466	0.8	9351
15	156.0767	0.7	8008
16	158.9533	1.0	11822
17	166.8985	0.6	6348
18	172.9561	2.7	31015
19	173.0784	0.6	7167
20	174.9553	2.1	23424
21	175.0447	0.7	8070
22	175.1187	0.9	9789
23	176.1029	0.7	8517
24	176.9527	1.0	11672
25	183.0779	3.1	34773
26	186.0745	0.7	8011
27	190.0683	11.0	124708
28	191.0712	1.4	16150
29	192.0838	0.6	7035
30	214.0894	0.5	6248
31	217.1046	1.2	13659
32	217.1275	0.7	8135
33	223.0960	0.8	9297
34	239.0884	0.6	6810
35	249.0995	0.8	9500
36	249.9821	0.6	6385
37	257.2467	1.2	14143
38	264.1050	0.6	6456
39	264.1239	100.0	1136988
40	265.1259	15.4	174993
41	266.1204	6.3	72097
42	267.1225	1.2	13321
43	273.1668	0.7	7813
44	279.0930	1.4	15739
45	279.1586	1.5	17108
46	281.2103	0.9	10329
47	295.1941	1.3	15225
48	301.1409	1.0	10808
49	309.1504	0.6	6526
50	311.1661	1.2	13092
51	313.2038	0.8	8831
52	315.1769	0.6	7248
53	324.1618	0.6	7274
54	327.0775	0.8	8850
55	331.2091	0.8	8880
56	336.2206	0.6	7009
57	338.1779	0.7	8000
58	352.1928	2.7	30485
59	353.1956	0.7	8004
60	354.1940	0.6	6408
61	359.2400	0.6	6279
62	362.1788	0.8	9290

Bruker Compass DataAnalysis 4.0 Acquisition Date 15.09.2020 11:11:50 Page 2 of 3

Figure S1.47: HRMS (ESI) peak table of 12.

High Resolution Mass Spectrometry Report

#	m/z	I %	I
63	369.3508	1.8	19917
64	373.2191	0.6	6843
65	380.2236	0.7	7418
66	381.2975	0.6	6386
67	383.1402	1.7	19741
68	388.1696	0.6	6881
69	391.2839	3.3	37183
70	392.2871	0.9	10063
71	393.2194	1.2	13667
72	393.2964	0.9	9737
73	395.2200	0.7	7466
74	399.2495	1.0	11452
75	403.2324	0.7	8290
76	413.2657	2.6	29877
77	414.2688	0.7	7660
78	419.3147	0.9	10403
79	425.2141	2.1	23334
80	425.2871	0.6	6820
81	425.3622	2.9	33064
82	426.3652	0.9	9691
83	427.3915	0.6	7234
84	429.2397	0.6	6636
85	439.2026	0.9	10766
86	441.2970	0.6	6658
87	447.3445	2.6	29123
88	448.3482	0.8	9272
89	451.4503	0.6	6994
90	473.3200	4.3	48678
91	474.3233	1.5	16922
92	475.3188	2.0	22736
93	476.3217	0.7	8032
94	479.4815	0.8	9116
95	493.4969	0.7	8466
96	507.5128	1.1	12428
97	521.5278	0.6	6892
98	533.5284	0.9	10707
99	535.5436	0.8	9611
100	551.5024	0.6	6762

Acquisition Parameter

**General**	Fore Vacuum<br>Scan Begin	2.60e+000 mBar<br>75 m/z	High Vacuum<br>Scan End	1.37e-007 mBar<br>1700 m/z	Source Type<br>Ion Polarity	ESI<br>Positive
**Source**	Set Nebulizer<br>Set Dry Heater	0.4 Bar<br>180 °C	Set Capillary<br>Set End Plate Offset	3600 V<br>-500 V	Set Dry Gas	3.0 l/min
**Quadrupole**	Set Ion Energy (MS only)	4.0 eV				
**Coll. Cell**	Collision Energy	8.0 eV	Set Collision Cell RF	350.0 Vpp	55.0 Vpp	
**Ion Cooler**	Set Ion Cooler Transfer Time	55.0 µs	Set Ion Cooler Pre Pulse Storage Time	7.0 µs		

Bruker Compass DataAnalysis 4.0 Acquisition Date 15.09.2020 11:11:50 Page 3 of 3

Figure S1.48: HRMS (ESI) peak table of 12.

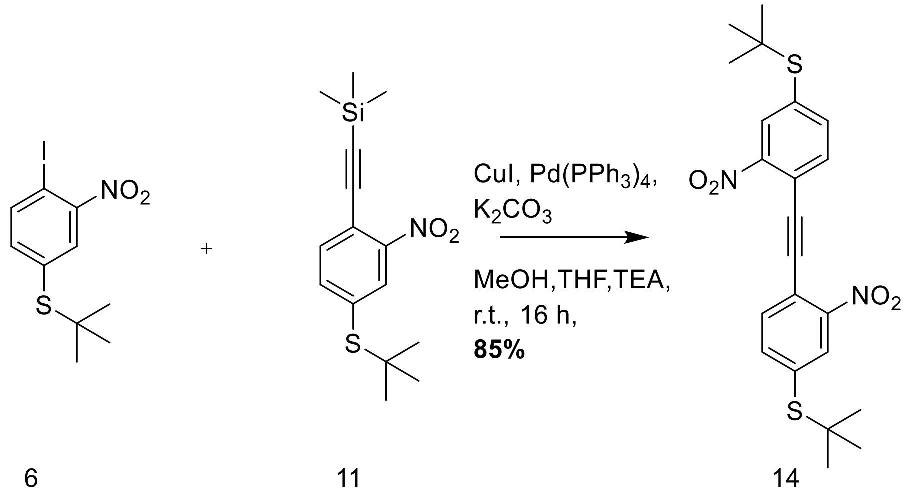

6 11 14

1,2-bis(4-(*tert*-butylthio)-2-nitrophenyl)ethyne 14: An oven dried Schlenk tube was charged with K_2CO_3 (306 mg, 2.21 mmol, 4 eq.), CuI (10.5 mg, 55.3 µmol, 0.1 eq.) and $Pd(PPh_3)_4$ (32 mg, 27.7 µmol, 0.05 eq.). The solids were purged with argon and dissolved in a degassed mixture of dry THF (2 mL), MeOH (2 mL) and TEA (1mL). To this was added the acetylene 11 (170 mg, 553 µmol, 1 eq.) and the iodine 6 (224 mg, 664 µmol, 1.2 eq.). The mixture was stirred at r.t. for 16 h and poured into aq. sat. NH_4Cl and extracted with DCM. The combined organic phase was dried over anhydrous $MgSO_4$, filtered, concentrated under reduced pressure and purified by column chromatography on silica gel (cyclohexane : toluene 1:1) followed by GPC($CHCl_3$) yielding a white solid (209 mg, 470 µmol, 85%).

^{1}H-NMR (500 MHz, $CDCl_3$) δ 8.28 (d, J = 1.6 Hz, 2H), 7.78 (dd, J = 8.0, 1.6 Hz, 2H), 7.76 (d, J = 8.0 Hz, 2H), 1.36 (s, 18H).

^{13}C-NMR (126 MHz, $CDCl_3$) δ 148.97, 141.08, 136.69, 134.82, 132.33, 117.69, 92.87, 47.92, 31.05.

HRMS (ESI) m/z: calcd. for $[C_{22}H_{24}N_2O_4S_2+Na]^+$ 467.1070 $[M+Na]^+$; found 467.1070
$[C_{44}H_{48}N_4O_8S_4+Na]^+$ 911.2255 $[2M+Na]^+$; found 911.2247

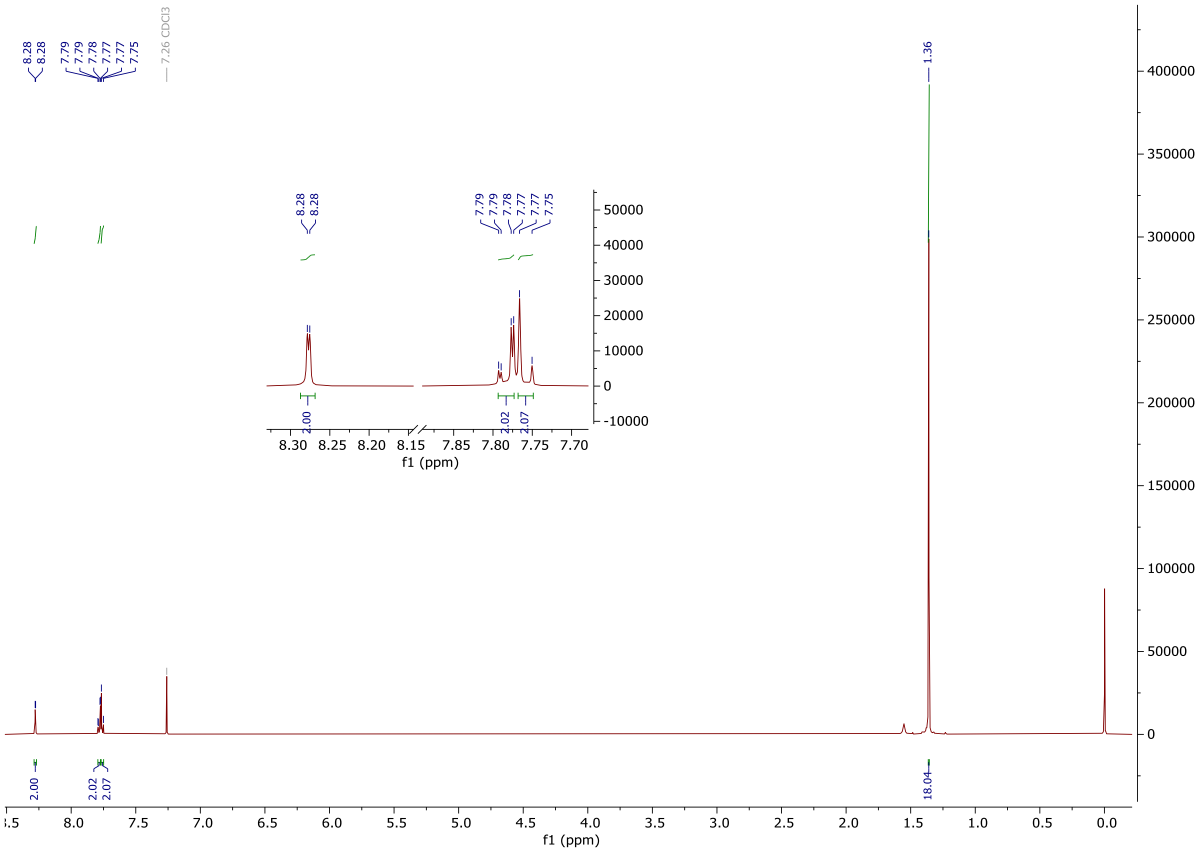

Figure S1.49: ^{1}H-NMR (500 MHz, $CDCl_3$) spectrum of 14.

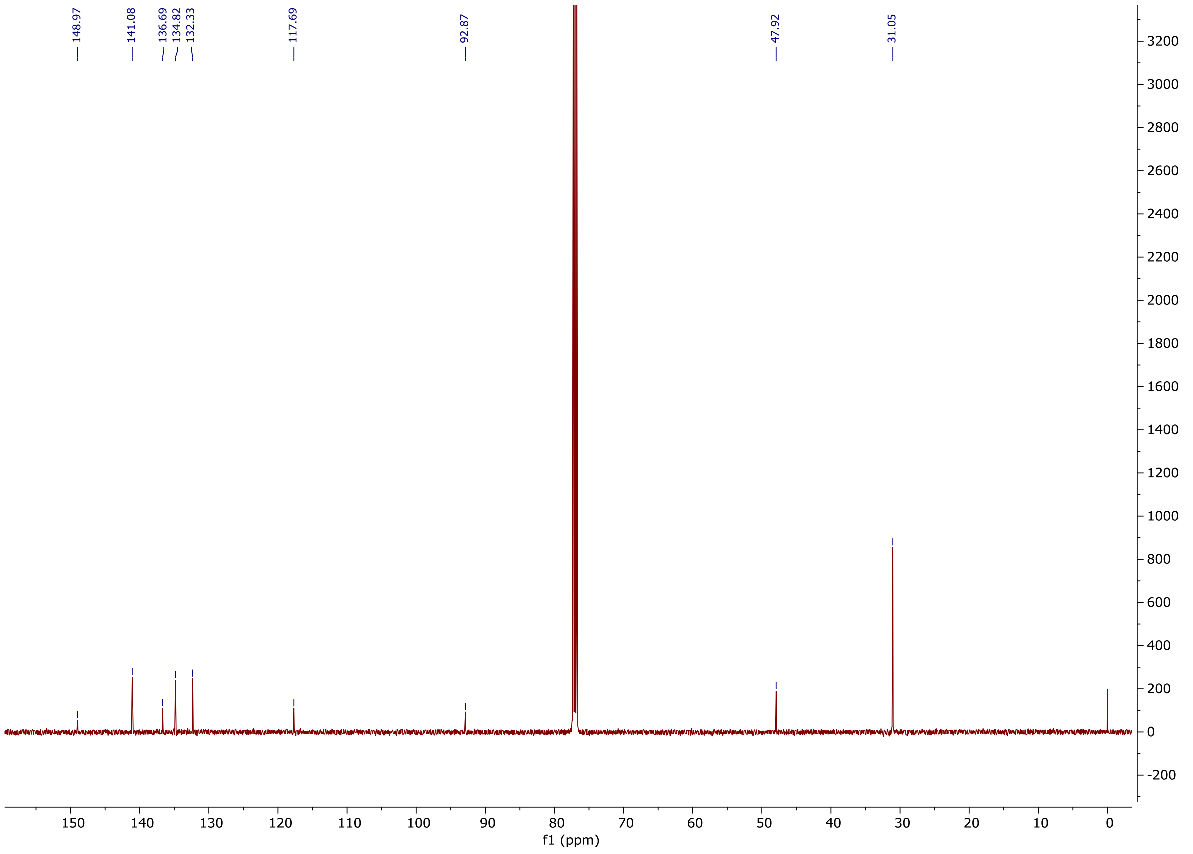

Figure S1.50: ^{13}C-NMR (126 MHz, $CDCl_3$) spectrum of 14.

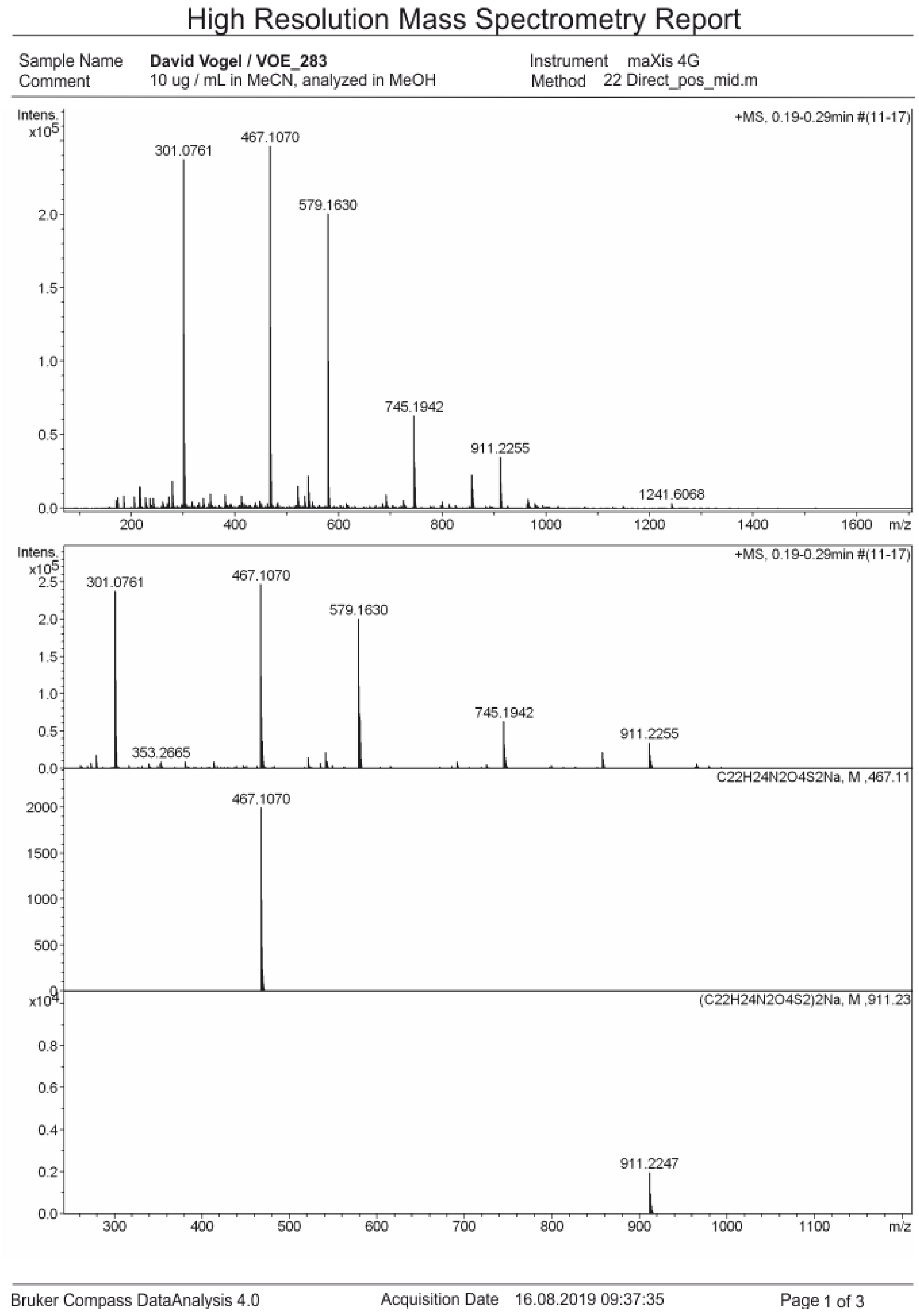

Figure S1.51: HRMS (ESI) spectrum of 14.

High Resolution Mass Spectrometry Report

Measured m/z vs. theoretical m/z

Meas. m/z	#	Formula	Score	m/z	err [mDa]	err [ppm]	mSigma	rdb	e⁻ Conf	z
467.1070	1	C 22 H 24 N 2 Na O 4 S 2	100.00	467.1070	-0.1	-0.2	16.4	11.5	even	1+
911.2255	1	C 44 H 48 N 4 Na O 8 S 4	100.00	911.2247	-0.8	-0.8	14.2	22.5	even	

Mass list

#	m/z	I %	I
1	171.0993	2.3	5779
2	173.0786	3.2	7949
3	185.1150	3.6	8867
4	205.0602	3.4	8370
5	215.1255	6.0	14842
6	217.1048	6.1	14944
7	226.9516	2.4	5903
8	227.1258	3.1	7632
9	236.0716	2.8	6949
10	242.2844	2.8	6848
11	261.1313	2.0	4834
12	263.0563	1.1	2821
13	269.1367	1.4	3402
14	273.1676	3.3	8208
15	279.0936	7.7	18895
16	280.0970	1.6	4008
17	297.2405	1.1	2718
18	301.0761	96.3	237796
19	301.1415	1.5	3803
20	301.2115	2.6	6522
21	302.0791	18.1	44649
22	303.0820	2.1	5101
23	305.1576	1.2	2965
24	317.1727	1.9	4768
25	331.2090	1.6	4008
26	339.1548	2.8	7023
27	341.2663	1.0	2511
28	349.1839	1.3	3296
29	352.1829	3.1	7599
30	353.2665	4.0	9764
31	381.2984	3.8	9447
32	382.3011	1.0	2558
33	391.2096	1.1	2692
34	393.2102	1.3	3147
35	393.2993	1.0	2575
36	413.2669	3.6	8960
37	414.2698	1.0	2483
38	417.3454	1.3	3317
39	437.2364	1.1	2643
40	439.0762	1.6	3960
41	447.3452	2.2	5388
42	449.3743	1.0	2592
43	451.1125	1.4	3387
44	463.2019	1.5	3718
45	467.1070	100.0	246890
46	468.1084	28.1	69398
47	469.1033	15.2	37575
48	470.1037	4.1	10120
49	471.1000	1.4	3482
50	481.1239	1.1	2720
51	483.0807	1.2	2898
52	483.1458	1.6	4014
53	521.1550	6.3	15443
54	522.1578	2.1	5133
55	525.0657	1.1	2623
56	535.0951	3.1	7652
57	535.1707	3.6	8915
58	536.1738	1.1	2764
59	541.1218	9.0	22182
60	542.1227	4.5	11055
61	543.1197	3.6	8974

Bruker Compass DataAnalysis 4.0 Acquisition Date 16.08.2019 09:37:35 Page 2 of 3

Figure S1.52: HRMS (ESI) peak table of 14.

High Resolution Mass Spectrometry Report

#	m/z	I %	I
62	544.1200	1.3	3311
63	549.1860	2.0	4821
64	579.1630	81.3	200736
65	579.2266	1.1	2712
66	580.1661	30.3	74912
67	581.1689	5.8	14432
68	615.1411	1.5	3758
69	616.1418	1.0	2461
70	685.4360	1.3	3303
71	691.3535	3.7	9226
72	692.3573	1.8	4451
73	705.5833	1.0	2582
74	725.4317	2.4	5863
75	726.4348	1.2	2903
76	745.1942	25.6	63290
77	746.1971	11.4	28224
78	747.1947	4.7	11490
79	748.1942	1.6	3985
80	797.3848	1.0	2562
81	799.2411	2.0	4905
82	800.2447	1.2	2865
83	813.2569	1.2	2914
84	825.4160	1.0	2550
85	857.2483	9.2	22726
86	857.3851	1.2	2862
87	858.2516	5.4	13318
88	859.2551	1.6	3884
89	911.2255	14.3	35207
90	912.2283	7.6	18762
91	913.2260	4.3	10634
92	914.2264	1.8	4347
93	963.5229	1.2	2950
94	965.2722	2.7	6693
95	966.2745	1.6	4059
96	967.2739	1.1	2795
97	979.2865	1.5	3776
98	980.2913	1.0	2564
99	1241.6068	1.5	3686
100	1242.6104	1.5	3643

Acquisition Parameter

**General**	Fore Vacuum	2.68e+000 mBar	High Vacuum	1.21e-007 mBar	Source Type	ESI
	Scan Begin	75 m/z	Scan End	1700 m/z	Ion Polarity	Positive
**Source**	Set Nebulizer	0.4 Bar	Set Capillary	3600 V	Set Dry Gas	4.0 l/min
	Set Dry Heater	180 °C	Set End Plate Offset	-500 V		
**Quadrupole**	Set Ion Energy (MS only)	4.0 eV				
**Coll. Cell**	Collision Energy	8.0 eV	Set Collision Cell RF	350.0 Vpp		
**Ion Cooler**	Set Ion Cooler Transfer Time	75.0 µs	Set Ion Cooler Pre Pulse Storage Time	10.0 µs		

Bruker Compass DataAnalysis 4.0 Acquisition Date 16.08.2019 09:37:35 Page 3 of 3

Figure S1.53: HRMS (ESI) peak table of 14.

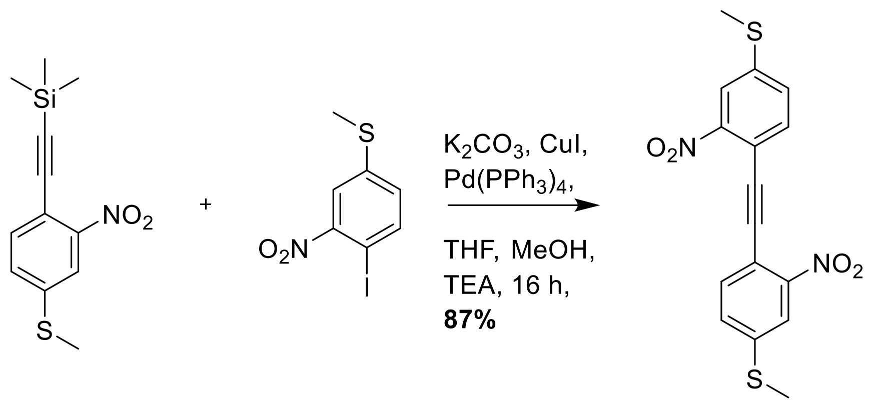

10 5 2b

1,2-bis(4-(methylthio)-2-nitrophenyl)ethyne 2b: An oven dried argon flushed Schlenk tube was charged with iodine 5 (61.0 mg, 207 µmol, 1.1 eq.), K_2CO_3 (104 mg, 752 µmol, 4 eq.), CuI (3.58 mg, 18.8 µmol, 0.1 eq.) and $Pd(PPh_3)_4$ (10.9 mg, 9.40 µmol, 0.05 eq.). The solids were purged with argon and dissolved in degassed THF (1 mL), MeOH (1 mL) and TEA (0.5 mL). To this was added a degassed solution of the TMS acetylene 10 (50.0 mg, 188 µmol, 1 eq.) in THF (1 mL). The solution was stirred at r.t. for 16 h, poured into an aq. sat. NH_4Cl solution and extracted with DCM. The combined organic phase was dried over anhydrous $MgSO_4$, filtered, concentrated under reduced pressure and purified by column chromatography on silica gel (DCM) and GPC($CHCl_3$) yielding 2b as an orange solid (59.0 mg, 164 µmol, 87%).

^{1}H-NMR (500 MHz, $CDCl_3$) δ 7.91 (d, J = 2.0 Hz, 2H), 7.68 (d, J = 8.2 Hz, 2H), 7.44 (dd, J = 8.3, 2.0 Hz, 2H), 2.58 (s, 6H).

^{13}C-NMR (126 MHz, $CDCl_3$) δ 149.37(extracted form HMBC/HMQC), 142.89, 135.11, 129.97, 121.02, 113.97, 91.99, 15.29.

HRMS (ESI) m/z: calcd. for $[C_{16}H_{12}N_2O_4S_2+H]^+$ 361.0305 $[M+H]^+$; found 361.0311
$[C_{16}H_{12}N_2O_4S_2+Na]^+$ 383.0126 $[M+Na]^+$; found 383.0131
$[C_{16}H_{12}N_2O_4S_2+K]^+$ 398.9864 $[M+K]^+$; found 398.9870

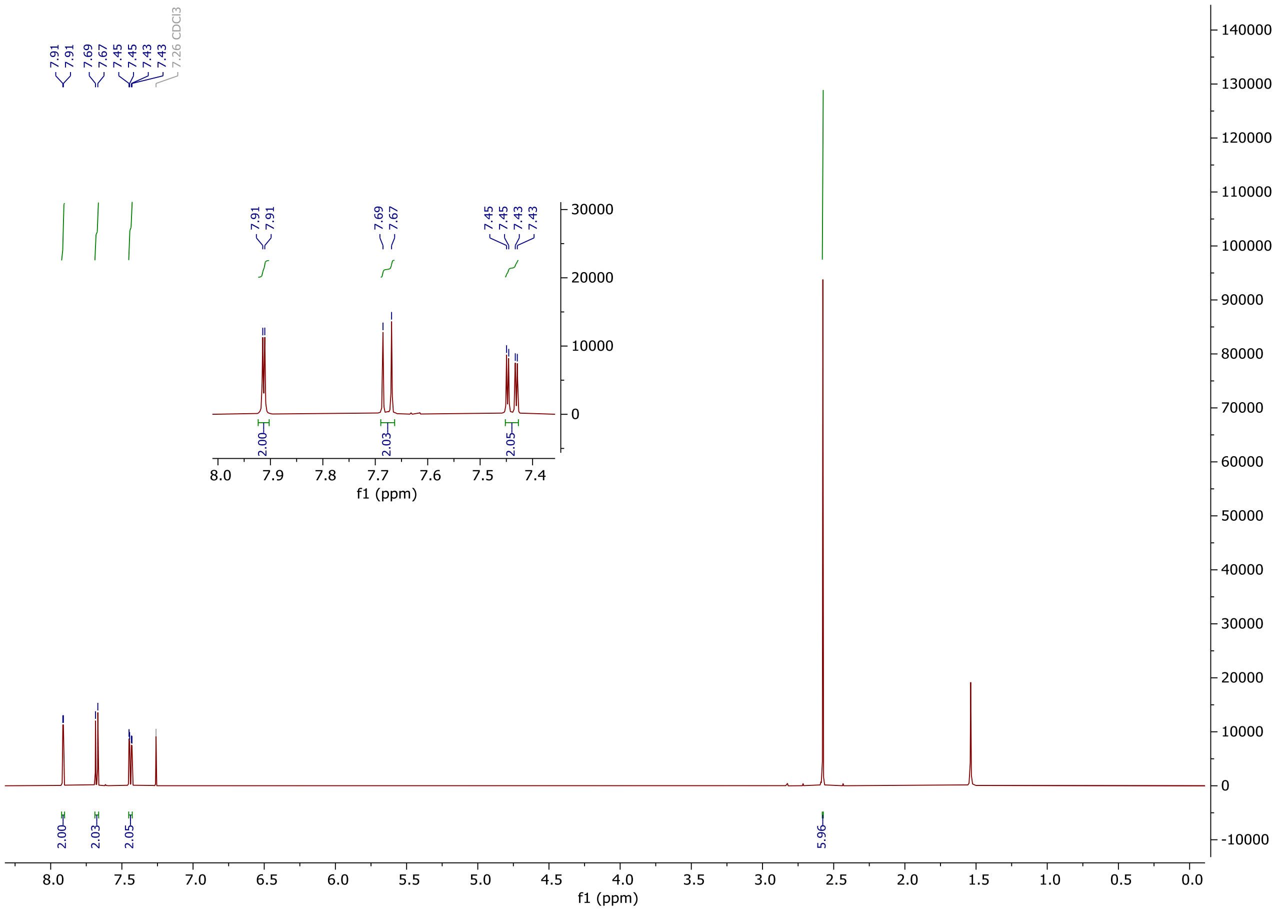

Figure S1.54: ^{1}H-NMR (500 MHz, $CDCl_3$) spectrum of 2b.

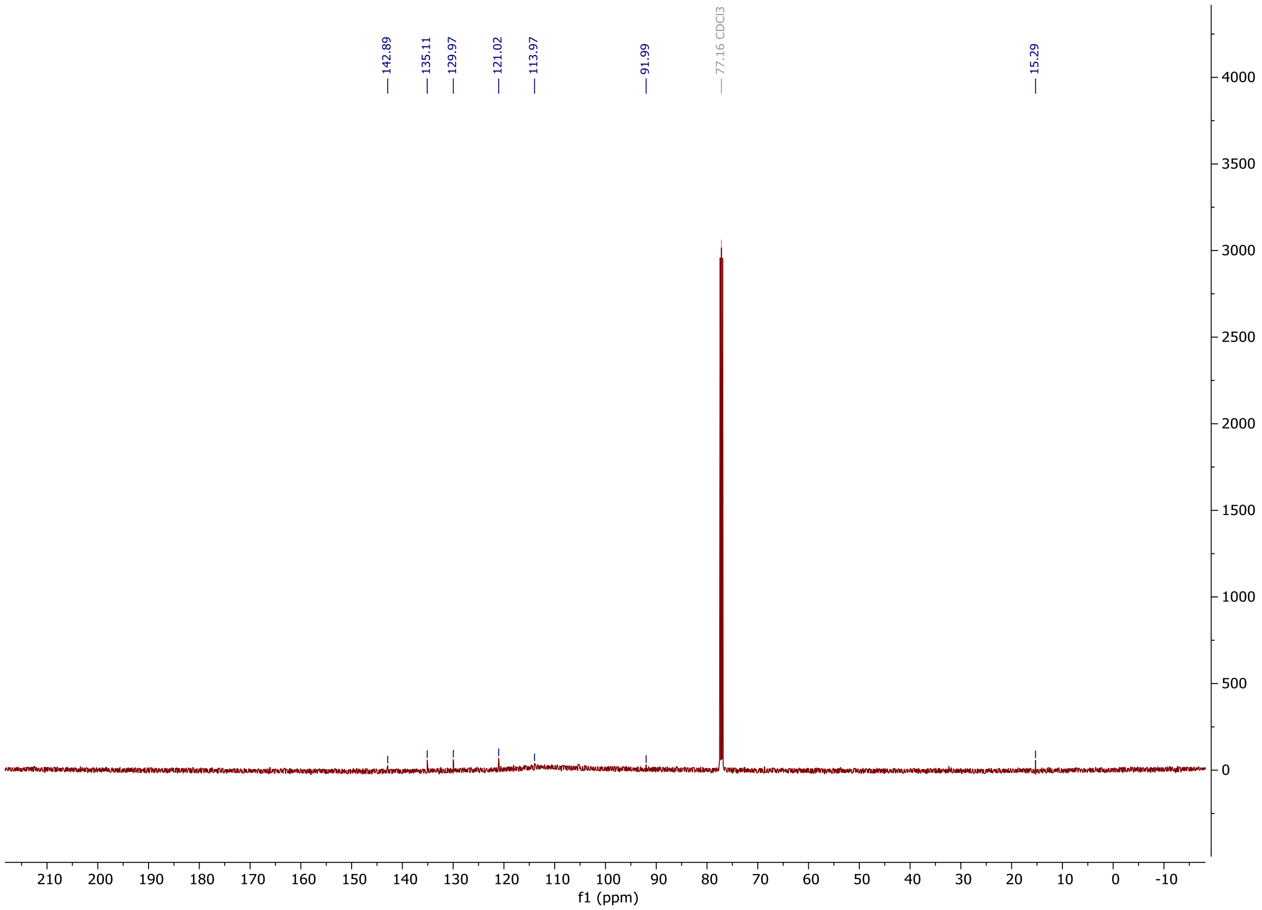

Figure S1.55: ^{13}C-NMR (126 MHz, $CDCl_3$) spectrum of 2b.

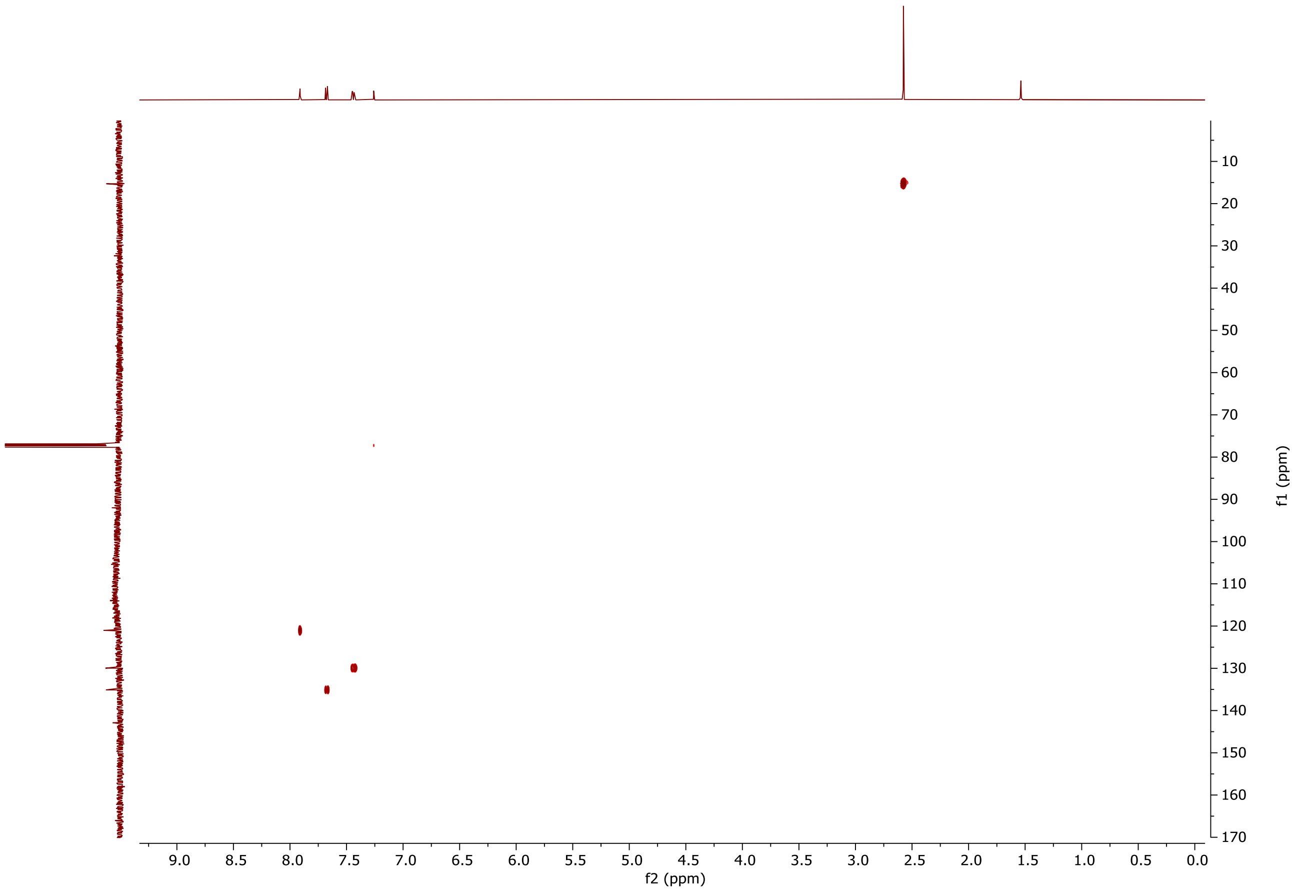

Figure S1.56: HMQC_GPSW ($CDCl_3$) spectrum of 2b.

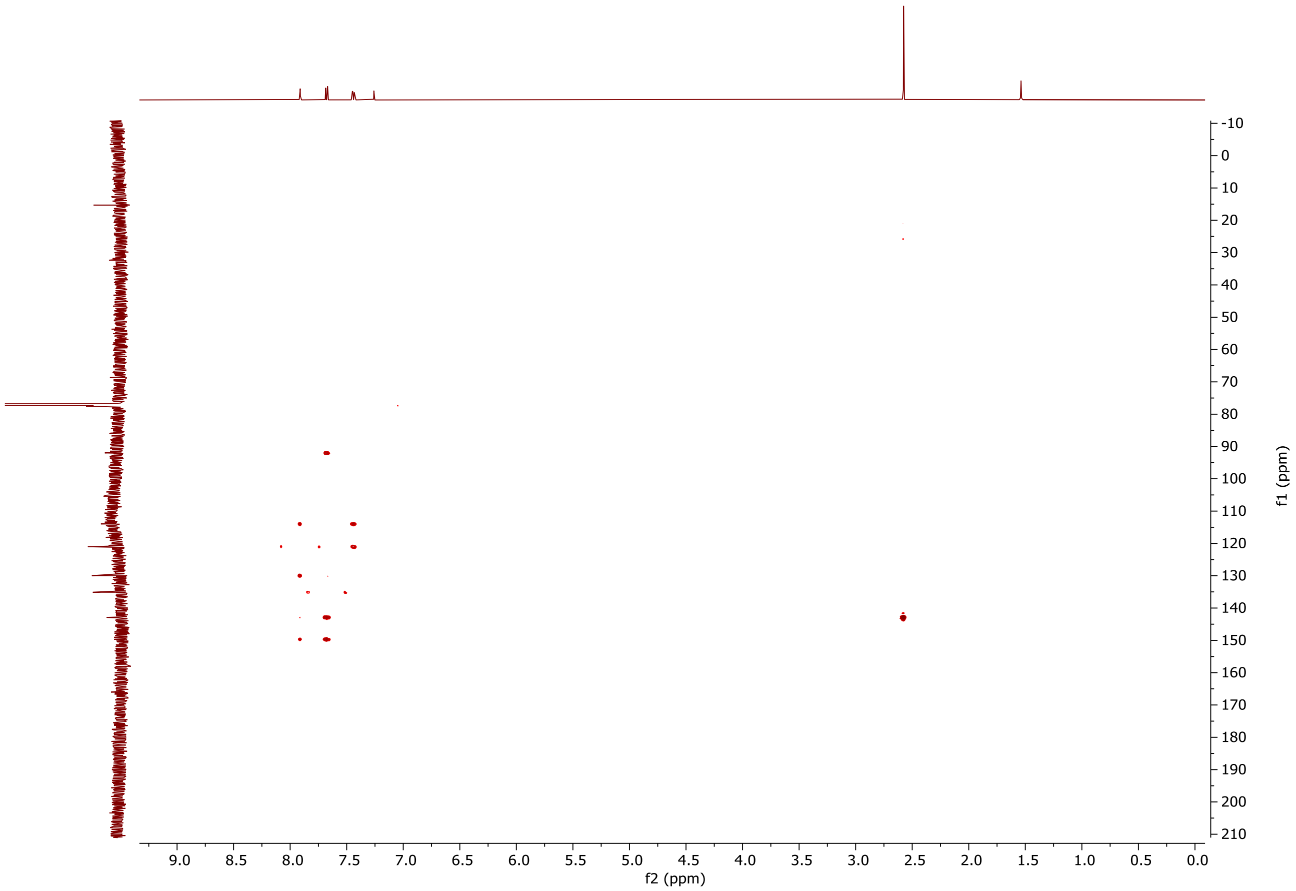

Figure S1.57: HMBC_GPSW ($CDCl_3$) spectrum of 2b.

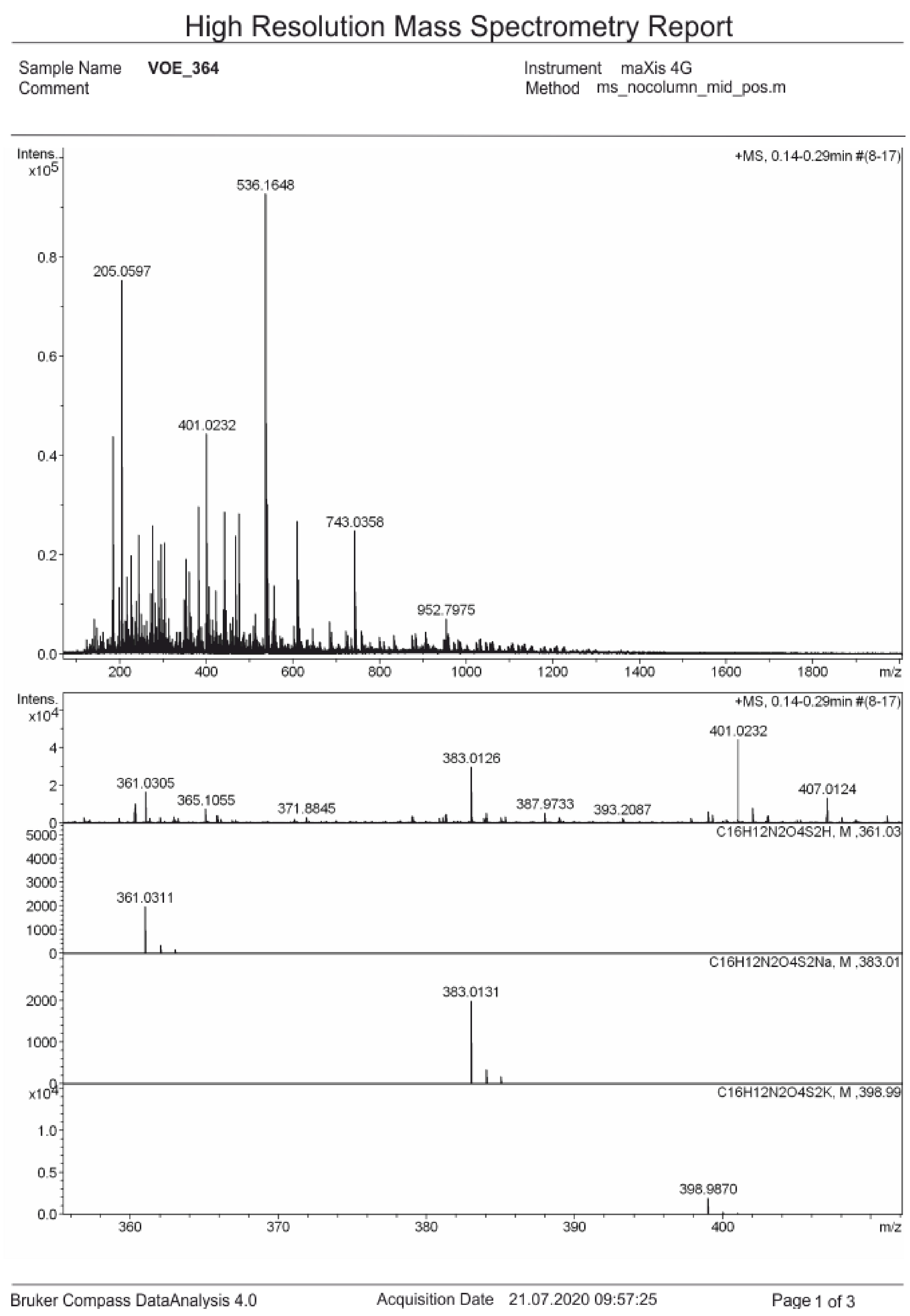

Figure S1.58: HRMS (ESI) spectrum of 2b.

High Resolution Mass Spectrometry Report

Measured m/z vs. theoretical m/z

Meas. m/z	#	Formula	Score	m/z	err [mDa]	err [ppm]	mSigma	rdb	e¯ Conf	z
361.0305	1	C 16 H 13 N 2 O 4 S 2	100.00	361.0311	0.6	1.7	11.4	11.5	even	1+
383.0126	1	C 16 H 12 N 2 Na O 4 S 2	100.00	383.0131	0.5	1.2	11.8	11.5	even	
398.9864	1	C 16 H 12 K N 2 O 4 S 2	100.00	398.9870	0.7	1.6	55.9	11.5	even	

Mass list

#	m/z	I %	I
1	140.9618	7.8	7245
2	141.9588	6.1	5617
3	147.0916	5.8	5413
4	183.0777	12.0	11090
5	185.1146	47.3	43906
6	199.1301	14.6	13570
7	205.0597	81.3	75452
8	206.0631	6.8	6314
9	213.1455	7.3	6816
10	215.1249	7.3	6774
11	216.9791	10.4	9680
12	217.0469	16.9	15664
13	217.1044	11.2	10350
14	220.9342	5.6	5159
15	226.9511	21.5	19978
16	227.1249	9.7	8990
17	229.1408	5.9	5494
18	229.8927	8.2	7649
19	235.9096	7.3	6763
20	236.0709	7.3	6764
21	237.0782	7.3	6763
22	239.0884	11.6	10788
23	243.9412	7.4	6857
24	245.0780	26.1	24194
25	251.0522	8.9	8237
26	255.1559	6.4	5959
27	261.1304	7.4	6904
28	271.1874	13.4	12472
29	273.1666	6.8	6354
30	275.1612	28.1	26030
31	280.9401	11.3	10523
32	281.0482	6.3	5879
33	284.8908	6.1	5655
34	288.9214	20.4	18934
35	291.1560	8.0	7436
36	294.9193	24.0	22228
37	301.1403	7.6	7085
38	303.8969	24.4	22667
39	305.1707	7.1	6577
40	313.2345	8.0	7404
41	348.9895	12.1	11180
42	350.9865	11.7	10883
43	353.1448	20.8	19311
44	353.2658	7.2	6716
45	354.1479	6.1	5700
46	360.3227	11.6	10768
47	361.0305	18.0	16716
48	365.1055	8.3	7657
49	381.2968	5.5	5060
50	383.0126	32.2	29868
51	384.0156	5.9	5500
52	387.9733	6.1	5638
53	398.9864	6.9	6425
54	401.0232	47.9	44479
55	402.0260	9.0	8342
56	407.0124	14.8	13706
57	413.2651	7.6	7074
58	416.9892	5.4	5016
59	423.0048	13.8	12844
60	439.1241	9.6	8883

Bruker Compass DataAnalysis 4.0 Acquisition Date 21.07.2020 09:57:25 Page 2 of 3

Figure S1.59: HRMS (ESI) peak table of 2b.

High Resolution Mass Spectrometry Report

#	m/z	I %	I
61	439.8716	9.9	9180
62	441.2967	31.1	28864
63	442.2998	9.4	8745
64	443.3332	5.7	5318
65	444.9867	9.7	8969
66	445.1191	8.0	7395
67	455.3123	6.9	6380
68	462.1457	8.1	7505
69	467.1011	25.7	23877
70	468.1021	11.3	10450
71	469.0993	7.6	7025
72	469.3264	6.0	5565
73	475.3247	30.6	28405
74	476.3278	8.7	8061
75	508.1876	6.2	5788
76	513.1430	8.9	8247
77	536.1648	100.0	92779
78	537.1654	48.6	45134
79	538.1632	33.9	31409
80	539.1630	12.8	11830
81	541.1201	32.7	30328
82	542.1206	15.7	14536
83	543.1181	11.8	10937
84	553.4581	7.4	6880
85	557.0939	15.0	13904
86	558.0947	8.0	7399
87	559.0926	7.0	6501
88	601.3912	6.2	5791
89	610.1832	29.0	26908
90	611.1838	16.3	15153
91	612.1815	12.9	11974
92	613.1815	5.5	5117
93	615.1386	5.8	5369
94	645.4169	5.7	5257
95	684.2018	7.3	6742
96	685.4343	5.4	5021
97	743.0358	26.9	24995
98	744.0380	10.7	9891
99	745.0344	6.6	6159
100	952.7975	7.9	7285

Acquisition Parameter

**General**	Fore Vacuum	2.39e+000 mBar	High Vacuum	1.21e-007 mBar	Source Type	ESI
	Scan Begin	75 m/z	Scan End	2000 m/z	Ion Polarity	Positive
**Source**	Set Nebulizer	2.0 Bar	Set Capillary	4500 V	Set Dry Gas	8.0 l/min
	Set Dry Heater	200 °C	Set End Plate Offset	-500 V		
**Quadrupole**	Set Ion Energy (MS only)	4.0 eV				
**Coll. Cell**	Collision Energy	8.0 eV	Set Collision Cell RF	600.0 Vpp	100.0 Vpp	
**Ion Cooler**	Set Ion Cooler Transfer Time	75.0 µs	Set Ion Cooler Pre Pulse Storage Time	10.0 µs		

Bruker Compass DataAnalysis 4.0 Acquisition Date 21.07.2020 09:57:25 Page 3 of 3

Figure S1.60: HRMS (ESI) peak table of 2b.

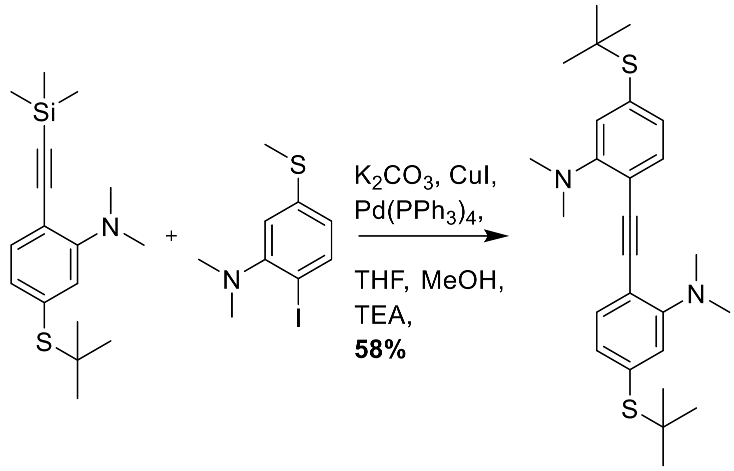

13 9 15

6,6'-(ethyne-1,2-diyl)bis(3-(*tert*-butylthio)-*N*,*N*-dimethylaniline) 15: An oven dried argon flushed Schlenk tube was charged with iodine 9 (40.9 mg, 122 µmol, 1.1 eq.), K_2CO_3 (61.4 mg, 444 µmol, 4 eq.), CuI (2.11 mg, 11.1 µmol, 0.1 eq.) and $Pd(PPH_3)_2Cl_2$ (3.4 mg, 6.00 µmol, 0.05eq.). The solids were purged with argon and dissolved in a degassed mixture of THF (1.2 mL), MeOH (1.2 mL) and TEA (0.6 mL). To this was added a degassed solution of acetylene 13 (33.9 mg, 111 µmol, 1eq.) in THF (0.6 mL). The solution was stirred at r.t. for 16 h, poured into aq. sat. NH_4Cl solution and extracted with DCM. The combined organic phase was dried over anhydrous Na_2SO_4, filtered, concentrated under reduced pressure and purified by column chromatography on silica gel (DCM) yielding 15 as a yellow solid (28.5 mg, 65.0 µmol, 58%).

^{1}H-NMR (400 MHz, $CDCl_3$) δ 7.42 (d, J = 7.8 Hz, 2H), 7.07 (d, J = 1.6 Hz, 2H), 7.05 (dd, J = 7.8, 1.7 Hz, 2H), 3.00 (s, 12H), 1.31 (s, 18H).

^{13}C-NMR (101 MHz, $CDCl_3$) δ 154.19, 133.87, 133.68, 129.17, 126.00, 116.03, 95.09, 46.54, 43.61, 31.22.

HRMS (ESI) m/z: calcd. for $[C_{26}H_{36}N_2S_2+H]^+$ 441.2390 $[M+H]^+$; found 441.2393

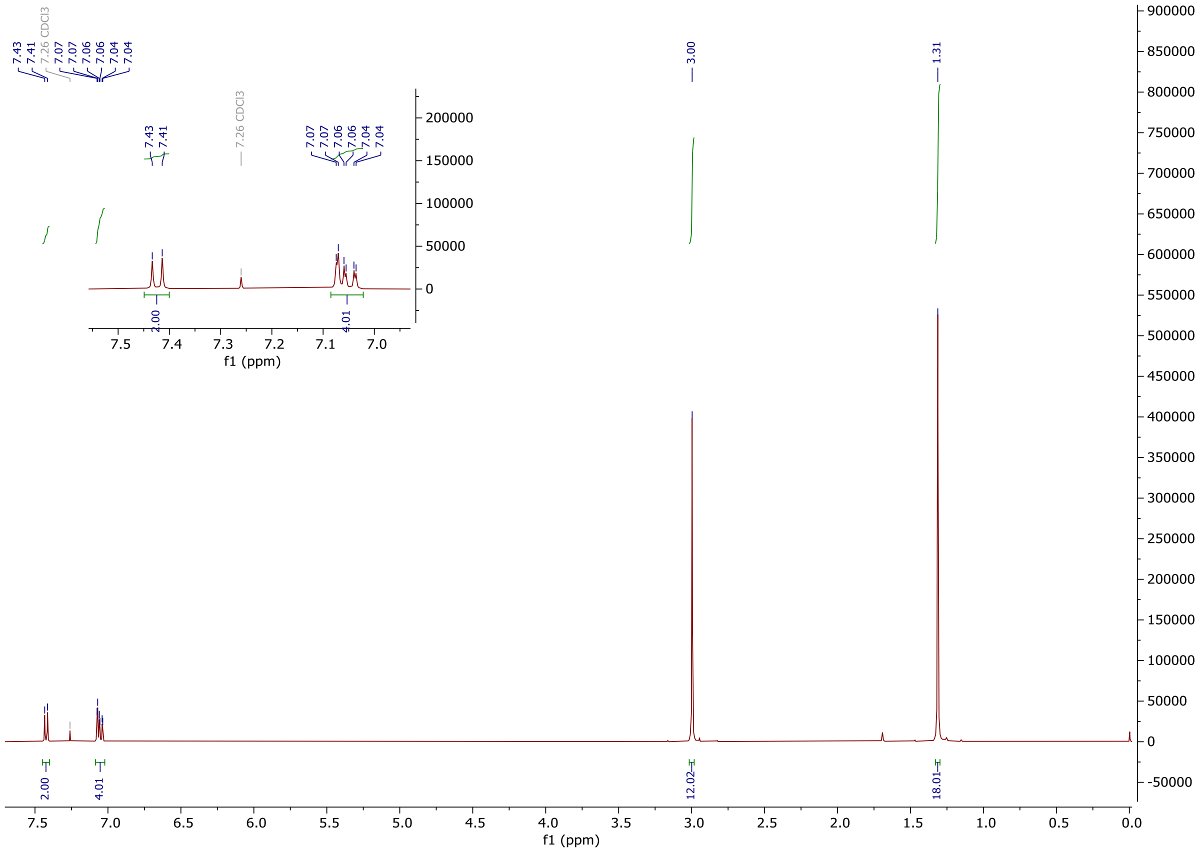

Figure S1.61: ^{1}H-NMR (500 MHz, $CDCl_3$) spectrum of 15.

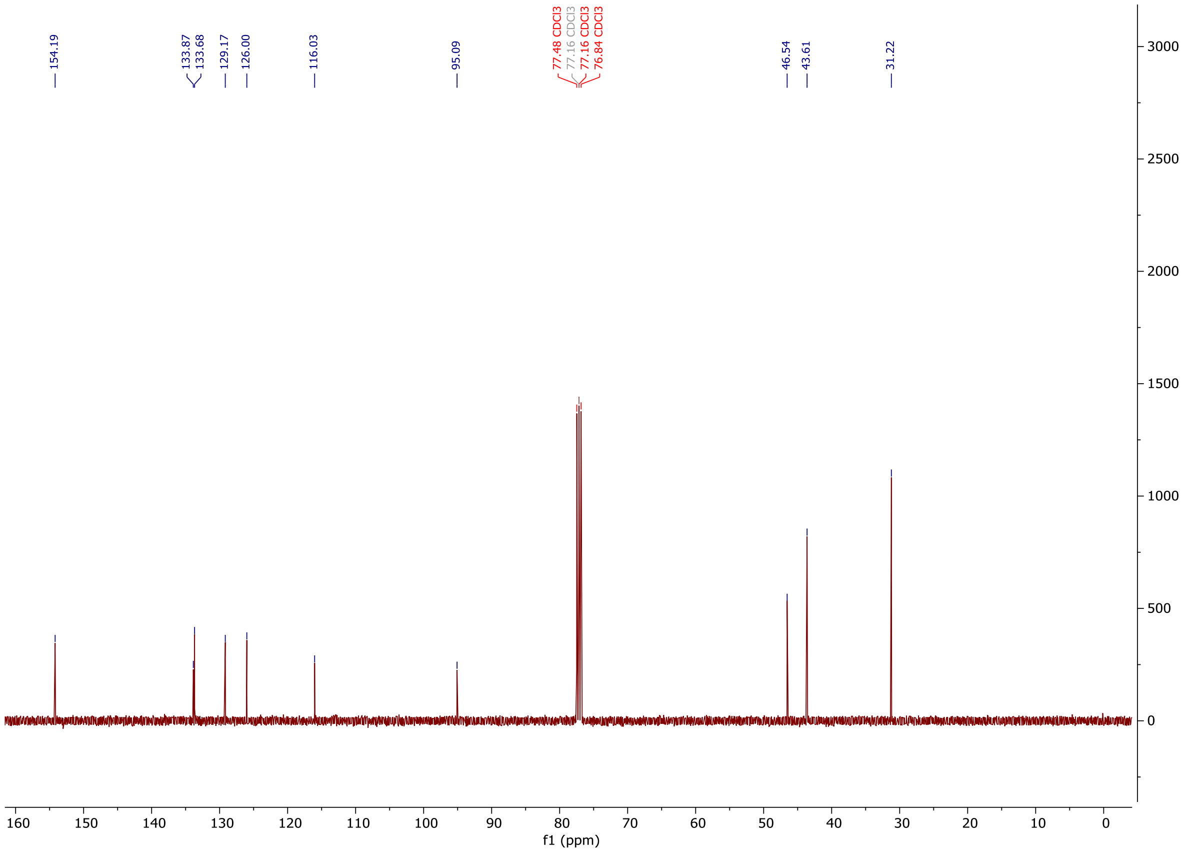

Figure S1.62: ^{13}C-NMR (126 MHz, $CDCl_3$) spectrum of 15.

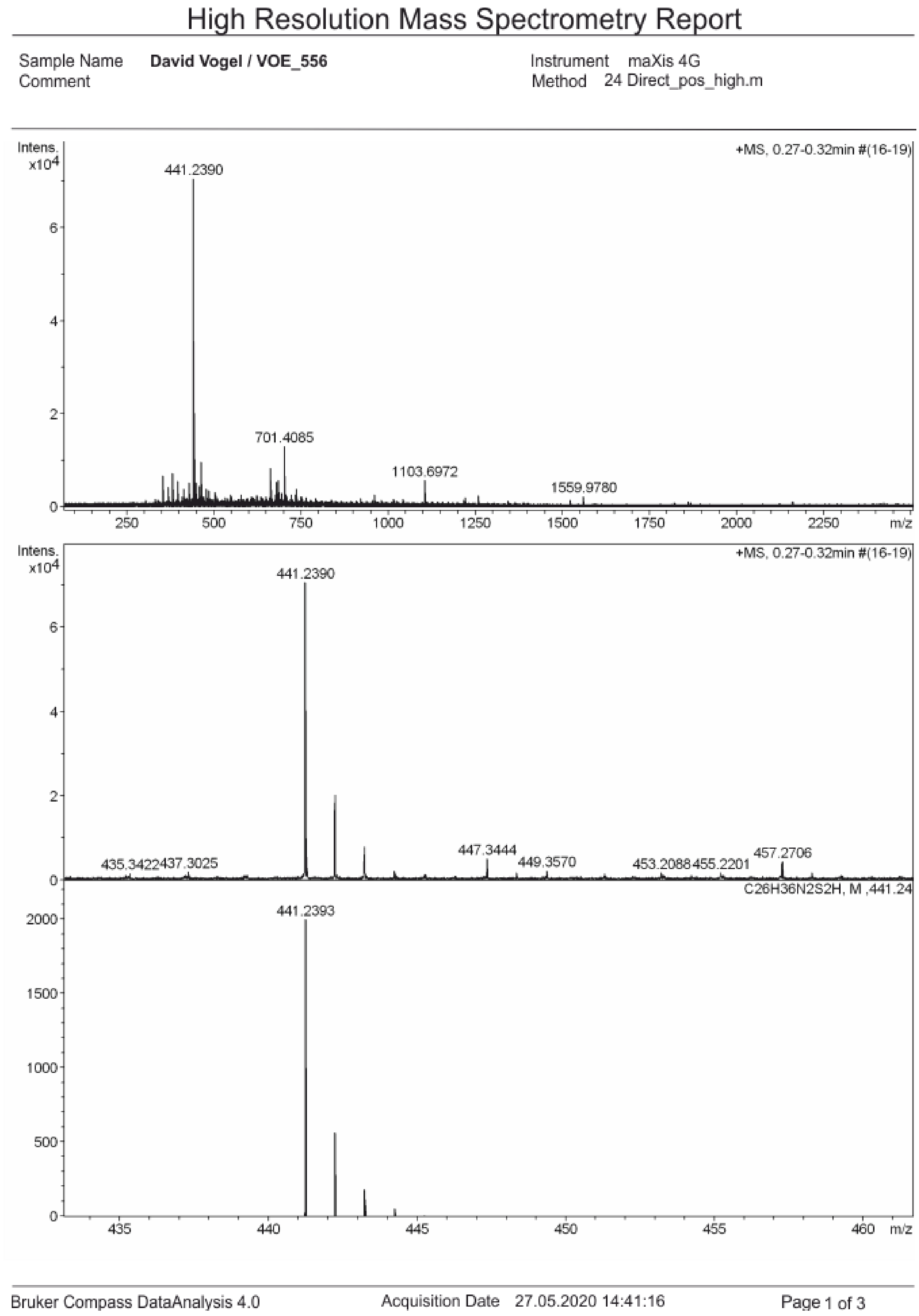

Figure S1.63: HRMS (ESI) spectrum of 15.

High Resolution Mass Spectrometry Report

Measured m/z vs. theoretical m/z

Meas. m/z	#	Formula	Score	m/z	err [mDa]	err [ppm]	mSigma	rdb	e⁻ Conf	z
441.2390	1	C 26 H 37 N 2 S 2	100.00	441.2393	0.3	0.7	15.3	9.5	even	1+

Mass list

#	m/z	I %	I
1	353.2662	9.5	6690
2	369.2401	6.1	4325
3	381.2970	10.4	7315
4	382.3008	3.3	2319
5	383.1819	2.6	1837
6	397.2707	8.0	5637
7	409.1854	2.7	1913
8	409.2694	3.4	2384
9	413.2663	5.5	3890
10	421.3279	2.9	2035
11	423.2884	2.5	1756
12	429.2396	7.5	5287
13	430.2422	3.0	2150
14	435.3422	2.5	1768
15	437.3025	2.8	2011
16	441.2390	100.0	70654
17	441.2957	4.6	3221
18	442.2418	28.8	20317
19	443.2374	11.5	8118
20	444.2391	3.2	2248
21	447.3444	7.4	5260
22	448.3471	2.7	1942
23	449.3570	3.3	2312
24	453.2088	2.6	1809
25	455.2201	2.7	1877
26	457.2706	6.3	4478
27	463.2201	6.8	4799
28	463.3180	13.8	9725
29	464.2241	2.9	2068
30	464.3203	5.2	3649
31	465.3287	3.3	2336
32	469.3276	2.9	2031
33	471.2852	3.1	2217
34	479.1945	5.5	3889
35	481.3110	2.9	2048
36	483.2731	2.5	1797
37	485.3007	5.2	3655
38	489.3169	2.7	1890
39	503.1602	4.6	3261
40	505.1589	3.6	2569
41	509.3221	2.6	1871
42	533.3437	3.0	2140
43	539.2982	2.8	1967
44	547.1366	3.8	2665
45	549.1346	3.3	2339
46	577.3702	3.7	2594
47	597.3030	2.8	2011
48	605.4010	2.5	1786
49	609.3626	3.0	2108
50	611.3151	3.0	2086
51	617.3651	2.5	1797
52	621.3969	3.4	2401
53	621.4477	2.8	1965
54	625.3334	3.4	2373
55	635.4114	3.2	2243
56	637.3914	3.0	2090
57	639.3513	2.8	1969
58	649.4283	2.8	1972
59	649.4784	3.2	2283
60	653.3630	2.5	1740
61	659.9818	3.0	2085
62	663.4525	12.0	8446

Bruker Compass DataAnalysis 4.0 Acquisition Date 27.05.2020 14:41:16 Page 2 of 3

Figure S1.64: HRMS (ESI) peak table of 15.

High Resolution Mass Spectrometry Report

#	m/z	I %	I
63	664.4555	5.4	3847
64	665.4233	3.5	2507
65	677.5062	3.5	2489
66	679.4422	3.3	2357
67	680.4784	7.6	5388
68	681.4819	3.8	2651
69	685.4355	8.2	5805
70	686.4379	4.3	3046
71	693.4631	4.3	3007
72	695.3982	2.6	1853
73	701.4085	18.5	13090
74	702.4124	9.0	6389
75	703.4124	4.0	2802
76	705.4207	2.6	1807
77	707.4836	3.9	2769
78	708.5063	2.6	1863
79	709.4446	3.3	2352
80	721.4985	3.8	2687
81	721.5734	2.9	2043
82	733.5269	2.8	1960
83	735.5171	3.7	2632
84	736.5415	5.7	3993
85	737.4873	2.5	1775
86	737.5471	5.5	3855
87	738.5514	2.5	1768
88	749.5262	2.9	2083
89	751.5109	3.2	2234
90	763.5441	3.0	2131
91	791.5703	2.6	1846
92	922.0163	2.7	1898
93	959.9704	3.8	2656
94	1015.7188	2.5	1780
95	1103.6972	8.2	5803
96	1104.6977	6.1	4293
97	1105.6999	3.6	2560
98	1222.0094	3.0	2149
99	1259.9675	3.6	2559
100	1559.9780	3.3	2331

Acquisition Parameter

**General**	Fore Vacuum<br>Scan Begin	2.60e+000 mBar<br>75 m/z	High Vacuum<br>Scan End	1.14e-007 mBar<br>2500 m/z	Source Type<br>Ion Polarity	ESI<br>Positive
**Source**	Set Nebulizer<br>Set Dry Heater	0.4 Bar<br>180 °C	Set Capillary<br>Set End Plate Offset	3600 V<br>-500 V	Set Dry Gas	4.0 l/min
**Quadrupole**	Set Ion Energy (MS only)	4.0 eV				
**Coll. Cell**	Collision Energy	10.0 eV	Set Collision Cell RF	1000.0 Vpp		
**Ion Cooler**	Set Ion Cooler Transfer Time	160.0 µs	Set Ion Cooler Pre Pulse Storage Time	18.0 µs		

Bruker Compass DataAnalysis 4.0 Acquisition Date 27.05.2020 14:41:16 Page 3 of 3

Figure S1.65: HRMS (ESI) peak table of 15.

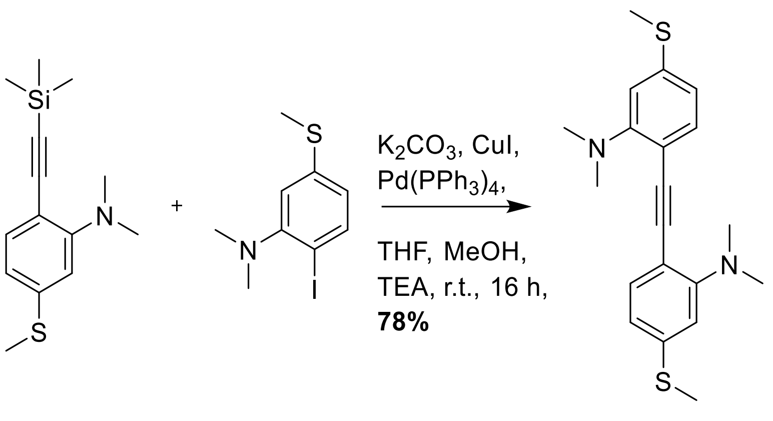

12 8 3b

6,6'-(ethyne-1,2-diyl)bis(*N*,*N*-dimethyl-3-(methylthio)aniline) 3b: An oven dried argon flushed Schlenk tube was charged with iodine 8 (178 mg, 607 µmol, 1 eq.), K_2CO_3 (336 mg, 2.43 mmol, 4 eq.), CuI (11.6 mg, 60.7 µmol, 0.1 eq.) and $Pd(PPh_3)_4$ (21.3 mg, 30.4 µmol, 0.05 eq.). The solids were purged with argon and dissolved in a mixture of degassed THF(2 mL), MeOH(5 mL) and TEA(3 mL). To this was added a degassed solution of acetylene 12 (160 mg, 607 µmol, 1eq.) in THF (3 mL). The solution was stirred at r.t. for 16 h, poured into an aq. sat. NH_4Cl solution and extracted with DCM. The combined organic phase was dried over anhydrous $MgSO_4$, filtered, concentrated under reduced pressure and purified by column chromatography on silica gel (DCM)(TLC spectroscopy reviled that the product is poorly visible at 254 nm but shows a clearly visible fluorescent spot at an irradiation of 366 nm) followed by GPC ($CHCl_3$) yielding 3b as a yellow solid (169 mg, 474 µmol, 78%).

^{1}H-NMR (500 MHz, $CDCl_3$) 7.39 (d, J = 8.0 Hz, 2H), 6.78 (d, J = 1.8 Hz, 2H), 6.75 (dd, J = 8.0, 1.8 Hz, 2H), 2.98 (s, 12H), 2.49 (s, 6H).

^{13}C-NMR (126 MHz, $CDCl_3$) δ 154.43, 139.70, 134.20, 118.00, 115.00, 112.66, 94.05, 43.51, 15.70.

HRMS (ESI) m/z: calcd. for $[C_{20}H_{24}N_2S_2+H]^+$ 357.1456 $[M+H]^+$; found 357.1454

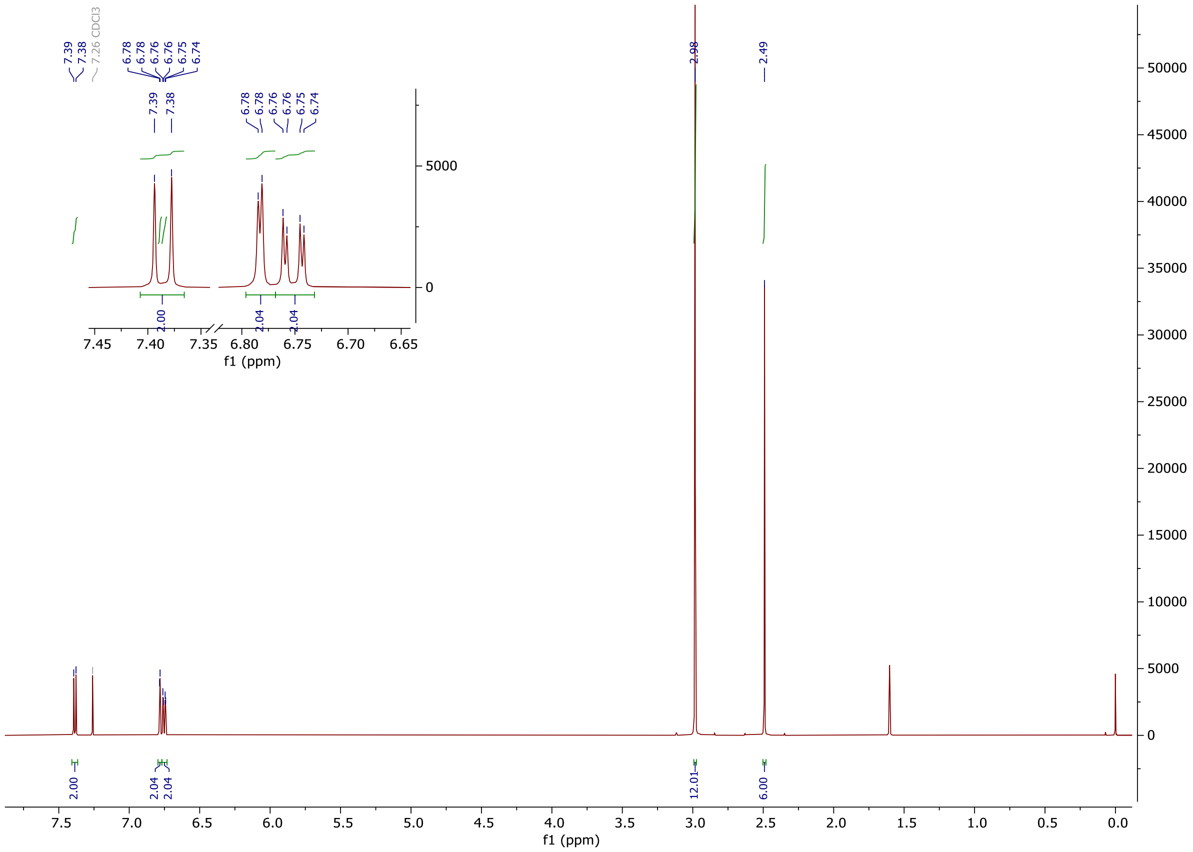

Figure S1.66: ^{1}H-NMR (500 MHz, $CDCl_3$) spectrum of 3b.

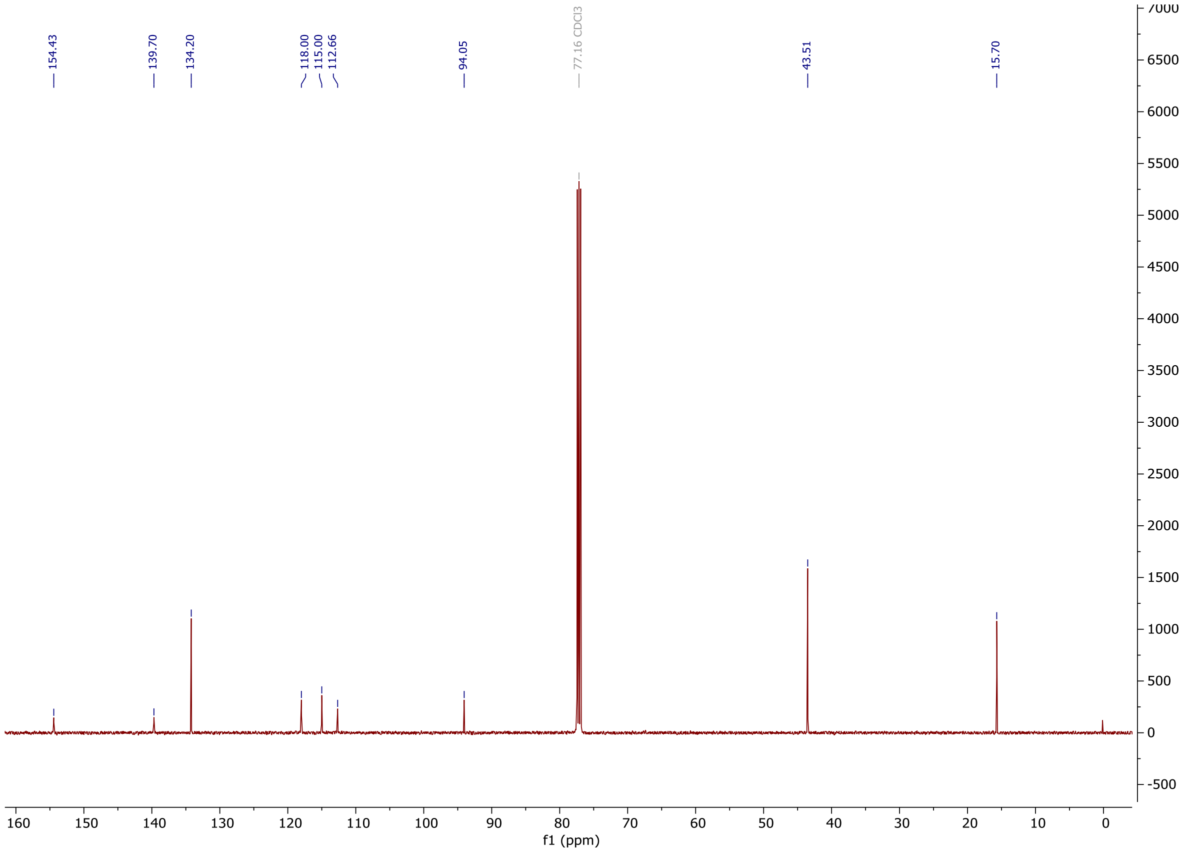

Figure S1.67: ^{13}C-NMR (126 MHz, $CDCl_3$) spectrum of 3b.

High Resolution Mass Spectrometry Report

Sample Name **VOE_544**
Comment

Instrument maXis 4G
Method ms_nocolumn_300-600_pos.m

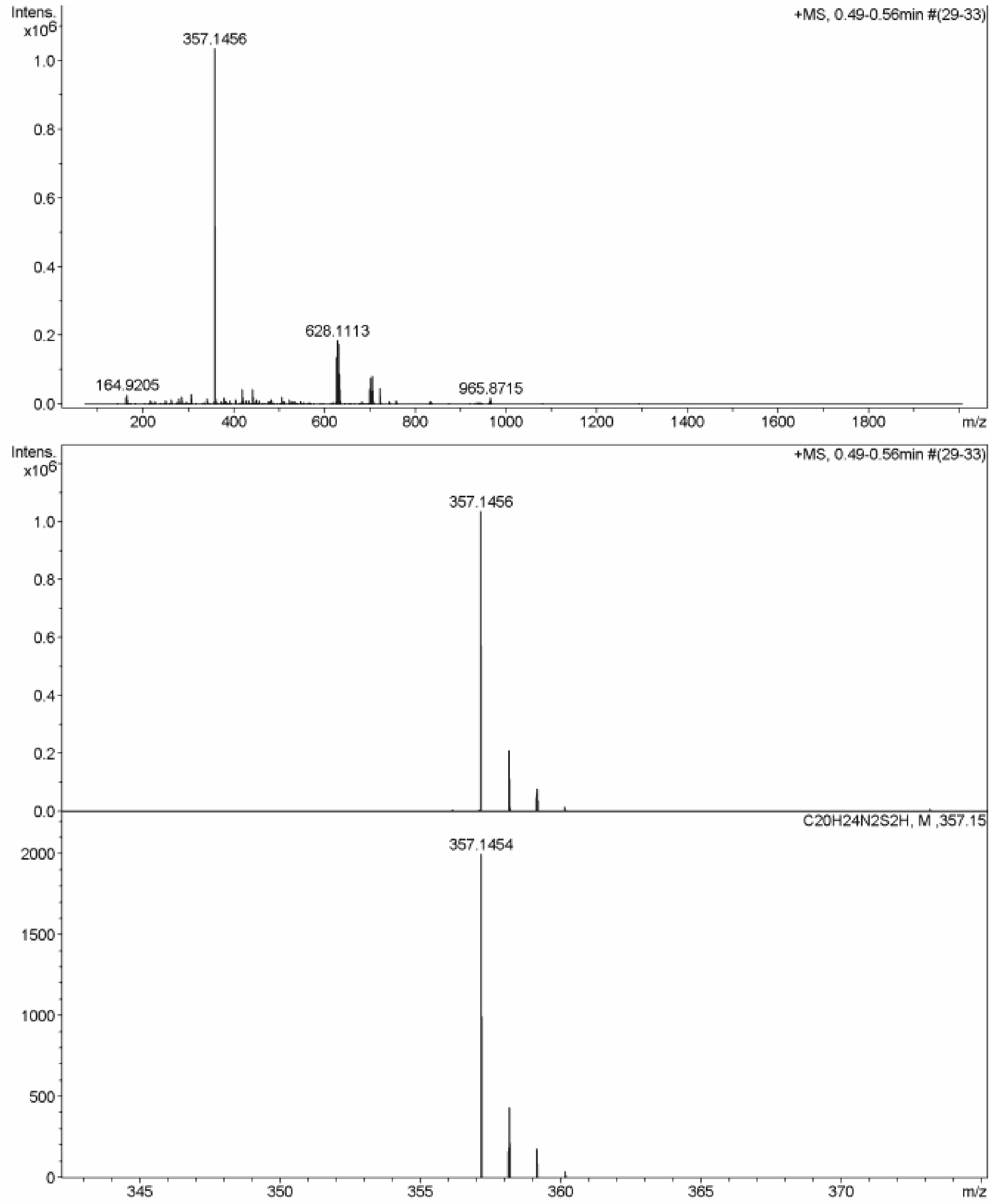

Bruker Compass DataAnalysis 4.0 Acquisition Date 13.05.2020 14:08:31 Page 1 of 3

Figure S1.68: HRMS (ESI) spectrum of 3b.

High Resolution Mass Spectrometry Report

Measured m/z vs. theoretical m/z

Meas. m/z	#	Formula	Score	m/z	err [mDa]	err [ppm]	mSigma	rdb	e⁻ Conf	z
357.1456	1	C 20 H 25 N 2 S 2	100.00	357.1454	-0.3	-0.7	27.4	9.5	even	1+

Mass list

#	m/z	I %	I
1	163.0390	1.9	20134
2	164.9205	2.9	29642
3	204.9127	0.5	4874
4	216.9794	0.6	5805
5	217.0467	1.3	13266
6	218.9285	0.8	7935
7	227.0398	1.0	10489
8	249.8977	1.1	11090
9	262.8875	1.4	13988
10	272.9367	0.5	5219
11	275.1638	0.7	7388
12	279.0927	1.6	16467
13	284.8695	2.2	22901
14	293.9801	0.7	7613
15	297.1955	0.6	6054
16	306.8513	3.0	30717
17	334.8753	0.6	5729
18	341.1134	1.7	17655
19	342.1190	0.6	6280
20	356.1368	0.8	7787
21	357.1456	100.0	1035853
22	358.1482	20.4	211042
23	359.1419	7.7	79506
24	360.1441	1.7	17637
25	373.1394	0.9	9601
26	379.1267	1.9	19350
27	382.8366	0.8	8151
28	391.8290	1.2	12901
29	404.8191	1.3	13836
30	417.0868	0.5	5516
31	419.3150	4.3	44648
32	420.3185	1.3	13265
33	426.8006	1.2	12941
34	433.3305	1.1	11633
35	441.2969	4.4	45747
36	442.3001	1.2	12819
37	447.3458	0.6	5849
38	448.7827	0.7	6739
39	449.2439	1.5	16000
40	450.2474	0.5	5218
41	455.3124	1.1	11171
42	476.8064	0.8	8472
43	478.3884	0.6	6334
44	480.5130	0.9	9234
45	482.4044	0.6	6125
46	483.0413	1.4	14417
47	506.5288	2.3	23375
48	507.5321	0.8	8736
49	508.5433	0.9	9351
50	511.7782	0.8	8419
51	522.2058	1.5	15812
52	523.2089	0.6	5981
53	524.7681	0.7	7358
54	526.4307	0.5	5245
55	528.5103	0.9	9026
56	533.0639	0.6	5891
57	533.7603	0.8	8552
58	534.0634	0.7	6967
59	536.0626	0.7	7056
60	546.7500	0.7	6881
61	548.5030	1.0	10434
62	553.4581	0.8	7825

Figure S1.69: HRMS (ESI) peak table of 3b.

High Resolution Mass Spectrometry Report

#	m/z	I %	I
63	565.6017	0.6	6226
64	618.7378	0.6	6130
65	624.1114	0.5	5396
66	626.1102	5.7	59276
67	627.1120	13.3	137936
68	628.1113	18.0	186395
69	629.1122	6.9	71411
70	630.1105	17.0	175662
71	631.1126	5.3	54648
72	632.1109	8.5	88256
73	633.1133	2.7	27617
74	634.1100	1.1	11819
75	675.6921	0.5	5060
76	683.6000	0.9	9589
77	684.6029	0.5	4962
78	699.5951	4.3	45035
79	700.6265	7.6	79197
80	701.6297	3.7	38295
81	702.6302	1.1	11250
82	705.5824	8.1	84153
83	706.5854	3.8	39469
84	707.5861	1.1	11462
85	721.5770	4.7	48812
86	722.5802	2.2	22815
87	723.5814	0.7	7119
88	742.6744	0.9	8811
89	758.6683	1.1	11004
90	759.6722	0.6	5937
91	832.1686	0.6	6162
92	833.1673	0.9	9410
93	834.1681	0.5	4976
94	835.1661	0.8	8129
95	936.8533	0.6	6037
96	942.8480	0.7	7742
97	944.8451	0.5	4902
98	963.8766	0.8	8657
99	965.8715	1.9	19360
100	966.8729	0.7	7172

Acquisition Parameter

**General**	Fore Vacuum<br>Scan Begin	2.39e+000 mBar<br>75 m/z	High Vacuum<br>Scan End	1.14e-007 mBar<br>2000 m/z	Source Type<br>Ion Polarity	ESI<br>Positive
**Source**	Set Nebulizer<br>Set Dry Heater	2.0 Bar<br>200 °C	Set Capillary<br>Set End Plate Offset	4500 V<br>-500 V	Set Dry Gas	8.0 l/min
**Quadrupole**	Set Ion Energy (MS only)	4.0 eV				
**Coll. Cell**	Collision Energy	8.0 eV	Set Collision Cell RF	600.0 Vpp		
**Ion Cooler**	Set Ion Cooler Transfer Time	75.0 µs	Set Ion Cooler Pre Pulse Storage Time	10.0 µs		

Bruker Compass DataAnalysis 4.0 Acquisition Date 13.05.2020 14:08:31 Page 3 of 3

Figure S1.70: HRMS (ESI) peak table of 3b.

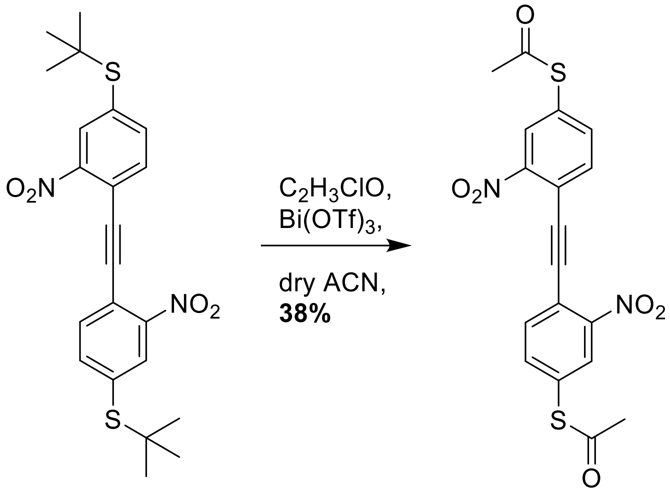

14 2a

***S,S'*-(ethyne-1,2-diylbis(3-nitro-4,1-phenylene)) diethanethioate 2a**: An oven dried argon flushed two necked round bottom flask was charged with tolane 14 (54.0 mg, 121 µmol, 1 eq.) and purged with argon. Dry CH_3CN (1 mL), acetyl chloride (0.02 mL, 303 µmol, 2.5 eq.) and bismuth(III)triflate (24.0 mg, 36.3 µmol, 0.3 eq.) were added and the reaction mixture was stirred at r.t. for 3 h. The reaction was quenched with water and extracted with DCM. The combined organic phase was dried over anhydrous $MgSO_4$, filtered, concentrated under reduced pressure and purified by column chromatography on silica gel (DCM : toluene 1:1) and GPC($CHCl_3$) yielding 2a as a yellow solid (19.0 mg, 46.0 µmol, 38%).

^{1}H-NMR (500 MHz, $CDCl_3$) δ 8.22 (d, J = 1.7 Hz, 2H), 7.85 (d, J = 8.1 Hz, 2H), 7.68 (dd, J = 8.1, 1.8 Hz, 2H), 2.50 (s, 6H).

^{13}C-NMR (126 MHz, $CDCl_3$) δ 191.34, 149.31, 138.47, 135.54, 131.06, 130.18, 118.45, 92.99, 30.50.

HRMS (ESI) m/z: calcd. for $[C_{18}H_{12}N_2O_6S_2+H]^+$ 417.0205 $[M+H]^+$; found 417.0210

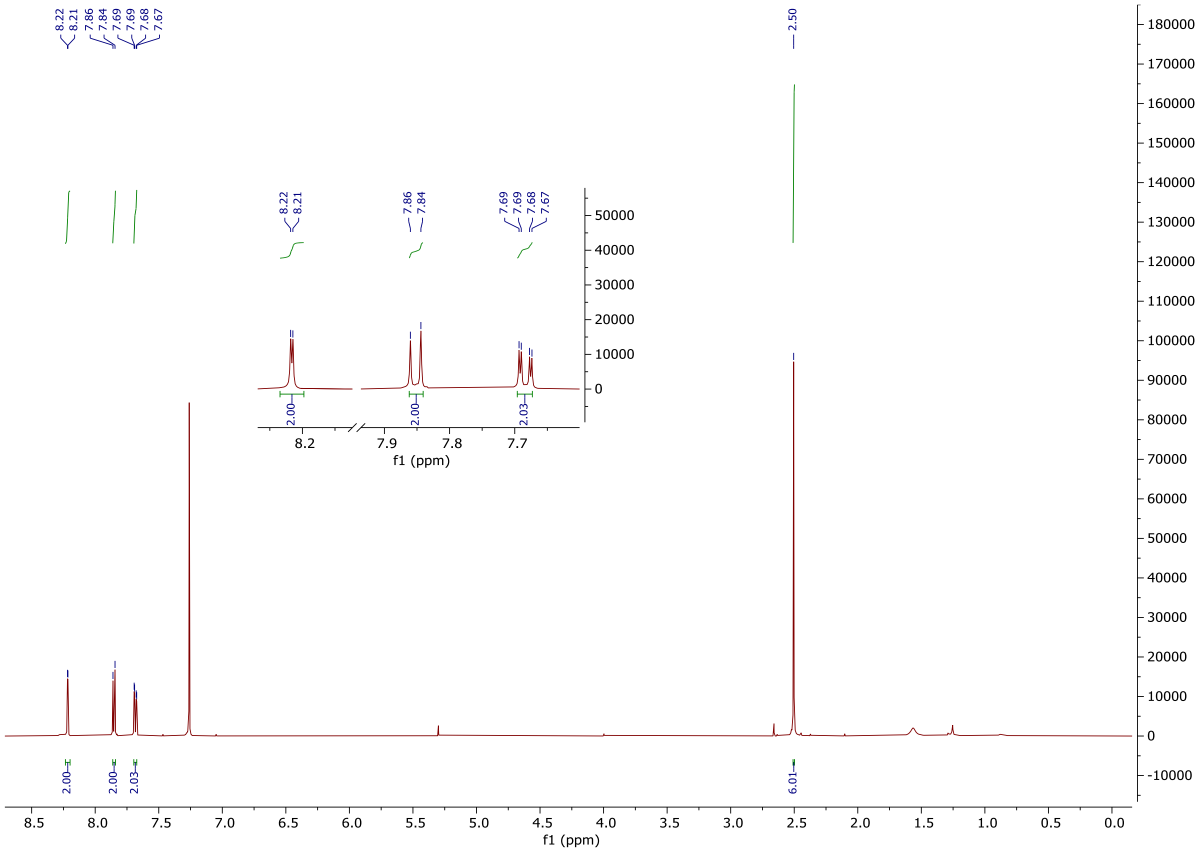

Figure S1.71: ^{1}H-NMR (500 MHz, $CDCl_3$) spectrum of 2a.

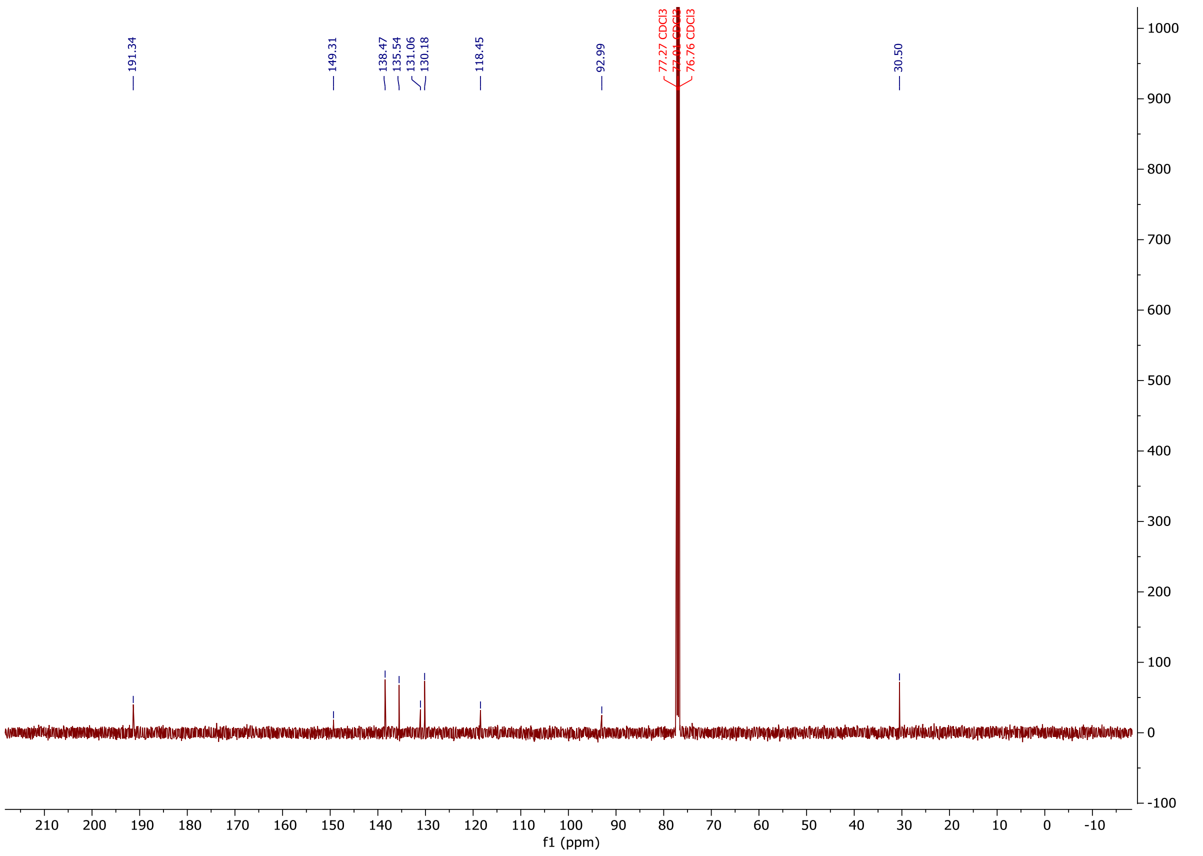

Figure S1.72: ^{13}C-NMR (126 MHz, $CDCl_3$) spectrum of 2a.

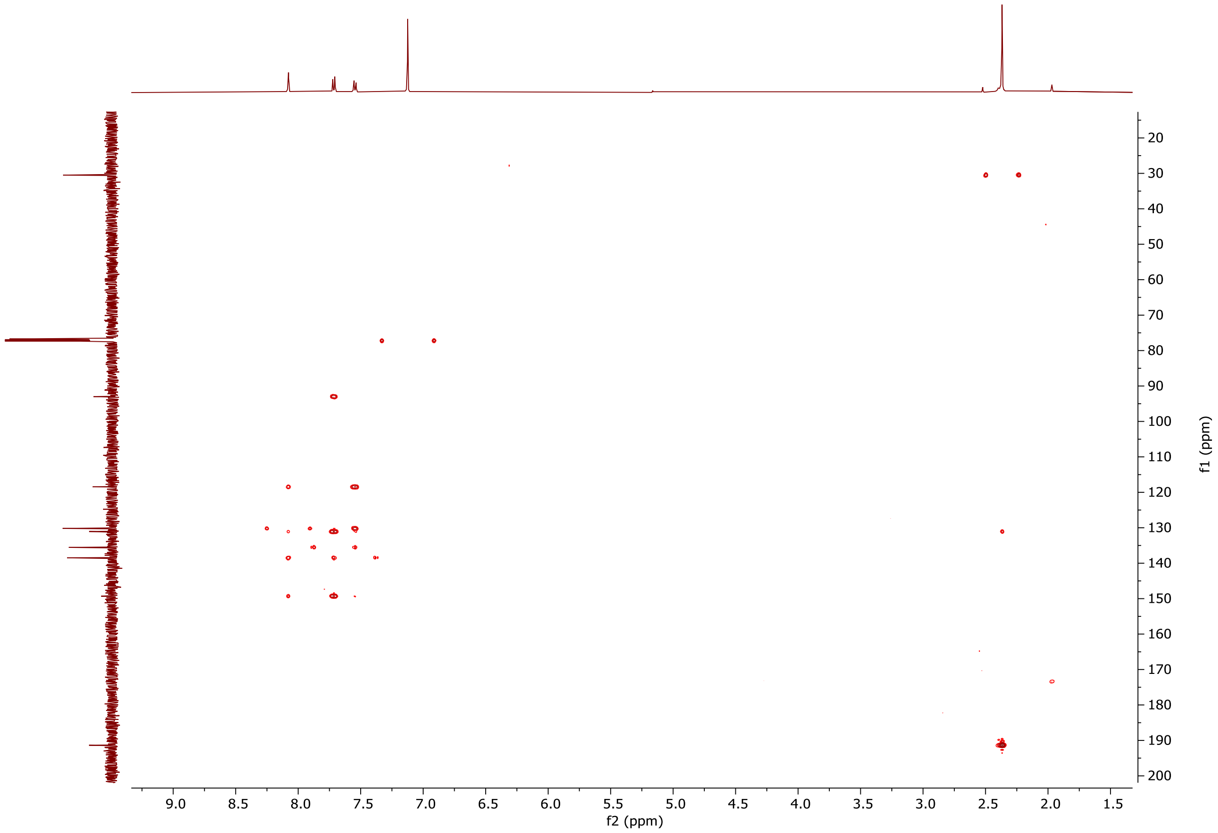

Figure S1.73: HMBC_GPSW ($CDCl_3$) spectrum of 2a.

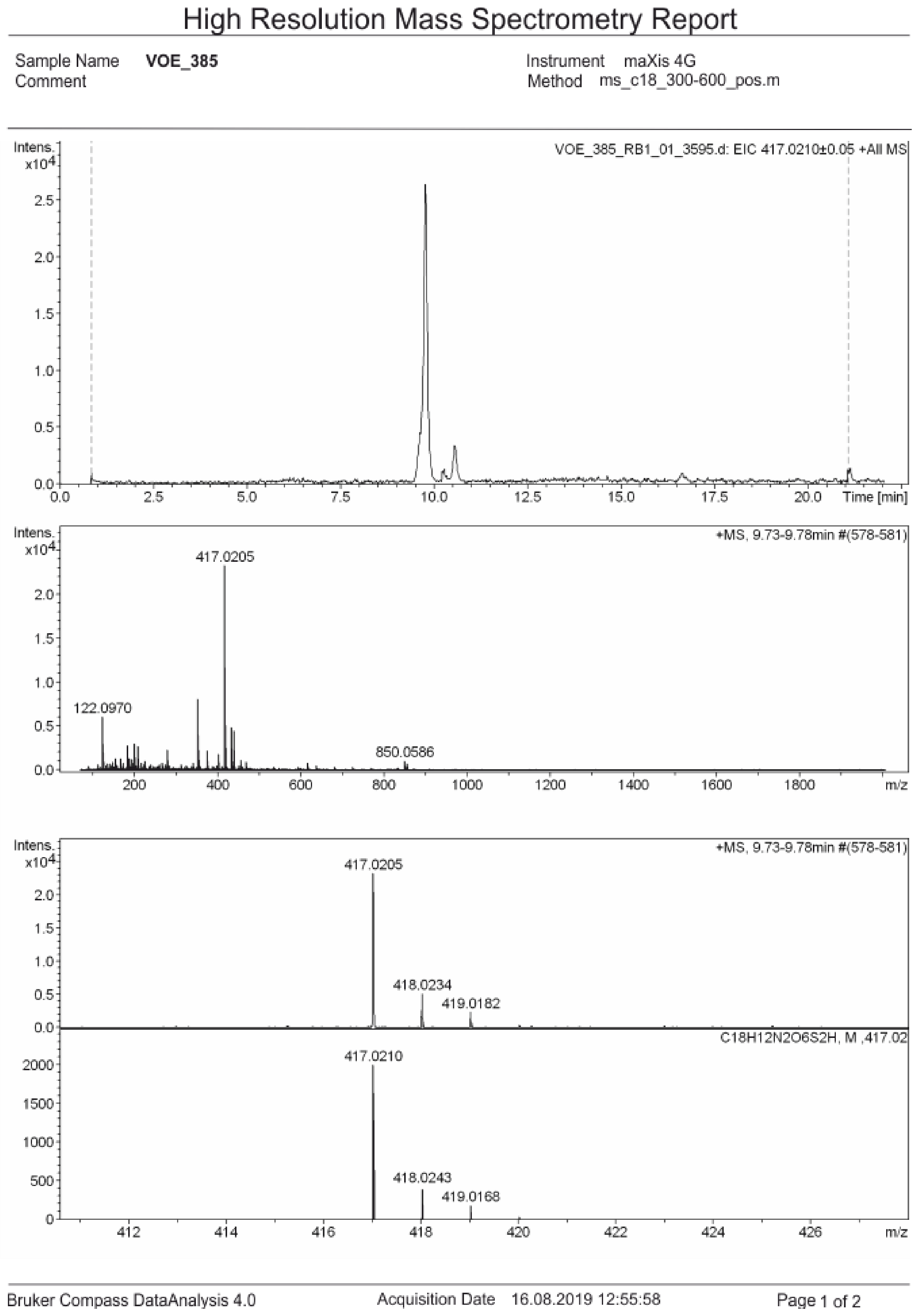

Figure S1.74: HRMS (ESI) spectrum of 2a

High Resolution Mass Spectrometry Report

Measured m/z vs. theoretical m/z

Meas. m/z	#	Formula	Score	m/z	err [mDa]	err [ppm]	mSigma	rdb	e⁻ Conf	z
417.0205	1	C 18 H 13 N 2 O 6 S 2	100.00	417.0210	0.5	1.2	15.7	13.5	even	1+

Mass list

#	m/z	I %	I
1	122.0970	26.1	6089
2	124.0875	8.7	2030
3	155.0471	5.6	1313
4	165.9961	5.7	1322
5	183.0782	12.0	2793
6	186.9560	5.6	1312
7	194.1174	5.5	1270
8	200.2006	13.0	3036
9	208.0062	11.8	2747
10	224.1276	4.7	1088
11	279.0929	9.9	2300
12	352.3392	35.0	8144
13	353.3415	10.1	2342
14	375.0097	9.7	2252
15	401.0256	8.1	1884
16	417.0205	100.0	23290
17	418.0234	22.1	5137
18	419.0182	10.3	2390
19	434.0470	20.9	4865
20	435.0499	4.6	1083
21	439.0027	19.4	4519
22	440.0052	4.6	1066
23	454.9765	5.2	1201
24	850.0586	4.8	1108

Acquisition Parameter

**General**	Fore Vacuum	2.60e+000 mBar	High Vacuum	1.22e-007 mBar	Source Type	ESI
	Scan Begin	75 m/z	Scan End	1700 m/z	Ion Polarity	Positive
**Source**	Set Nebulizer	2.0 Bar	Set Capillary	4500 V	Set Dry Gas	8.0 l/min
	Set Dry Heater	200 °C	Set End Plate Offset	-500 V		
**Quadrupole**	Set Ion Energy (MS only)	4.0 eV				
**Coll. Cell**	Collision Energy	8.0 eV	Set Collision Cell RF	350.0 Vpp		
**Ion Cooler**	Set Ion Cooler Transfer Time	75.0 µs	Set Ion Cooler Pre Pulse Storage Time	10.0 µs		

Bruker Compass DataAnalysis 4.0 Acquisition Date 16.08.2019 12:55:58 Page 2 of 2

Figure S1.75: HRMS (ESI) peak table of 2a

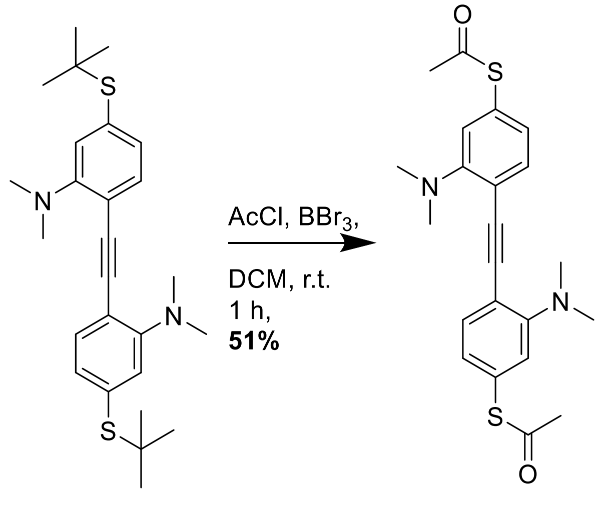

15 3a

***S,S'*-(ethyne-1,2-diylbis(3-(dimethylamino)-4,1-phenylene)) diethanethioate 3a**: An oven dried Schlenk tube was charged with tolane 15 (23.0 mg, 52.2 µmol, 1 eq.) and purged with argon. Dry DCM (1 mL) was added followed by acetyl chloride (0.11 mL, 123 mg, 1.57 mmol, 30 eq.) and BBr_3 (0.01 mL, 115 µmol, 2.2 eq.). The mixture was stirred at r.t. for 1 h, poured over ice and extracted fife times with DCM. The combined organic phase was washed with aq. 1M Na_2SO_3, Brine, dried over anhydrous Na_2SO_4, filtered, concentrated under reduced pressure and purified by GPC ($CHCL_3$) yielding 3a as an off-white solid (11.0 mg, 27.0 µmol, 51%).

^{1}H-NMR (500 MHz, $CDCl_3$) δ 7.48 (d, J = 7.9 Hz, 2H), 6.96 – 6.90 (m, 4H), 3.00 (s, 12H), 2.42 (s, 6H).

^{13}C-NMR (126 MHz, $CDCl_3$) δ 193.92, 154.63, 134.36, 128.61, 125.74, 122.64, 116.28, 94.98, 43.29, 30.28.

HRMS (ESI) m/z: calcd. for $[C_{22}H_{24}N_2O_2S_2+H]^+$ 413.1360 $[M+H]^+$; found 413.1352.

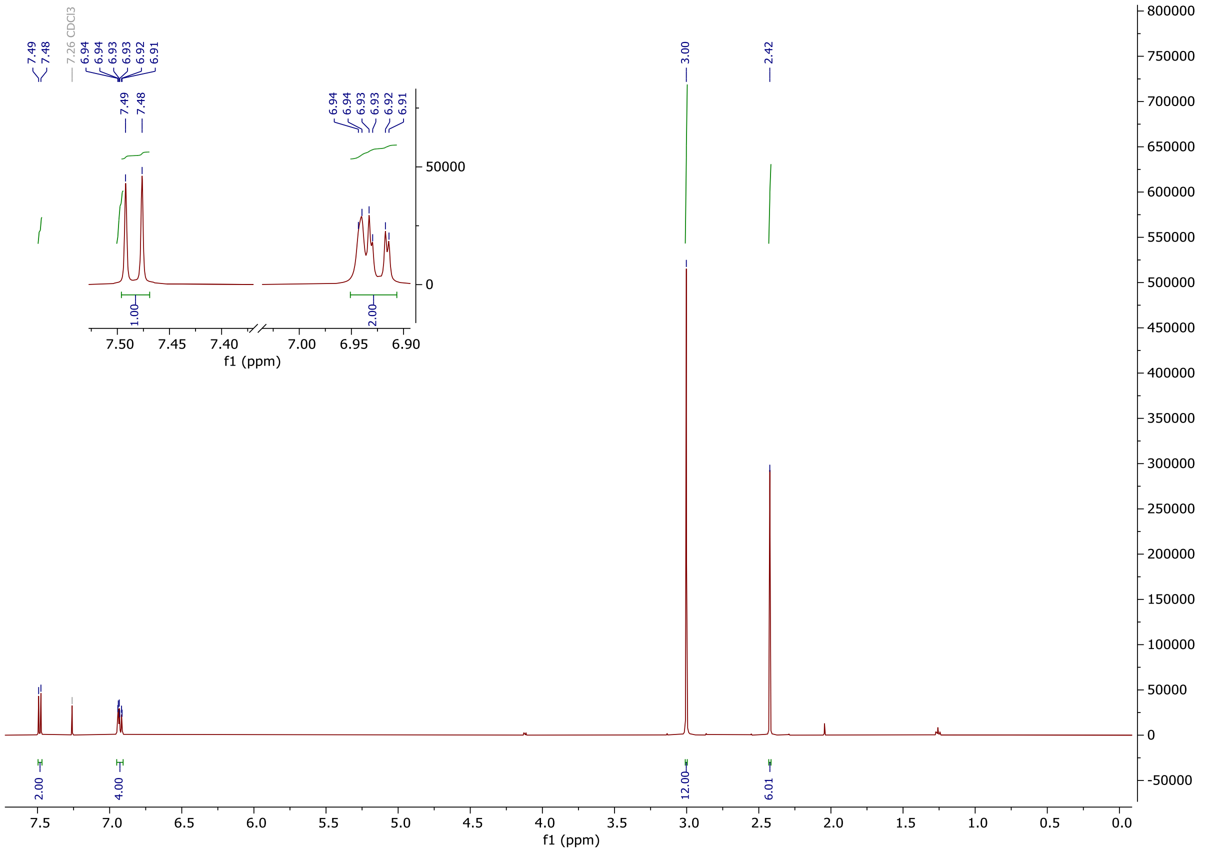

Figure S1.76: ^{1}H-NMR (500 MHz, $CDCl_3$) spectrum of 3a.

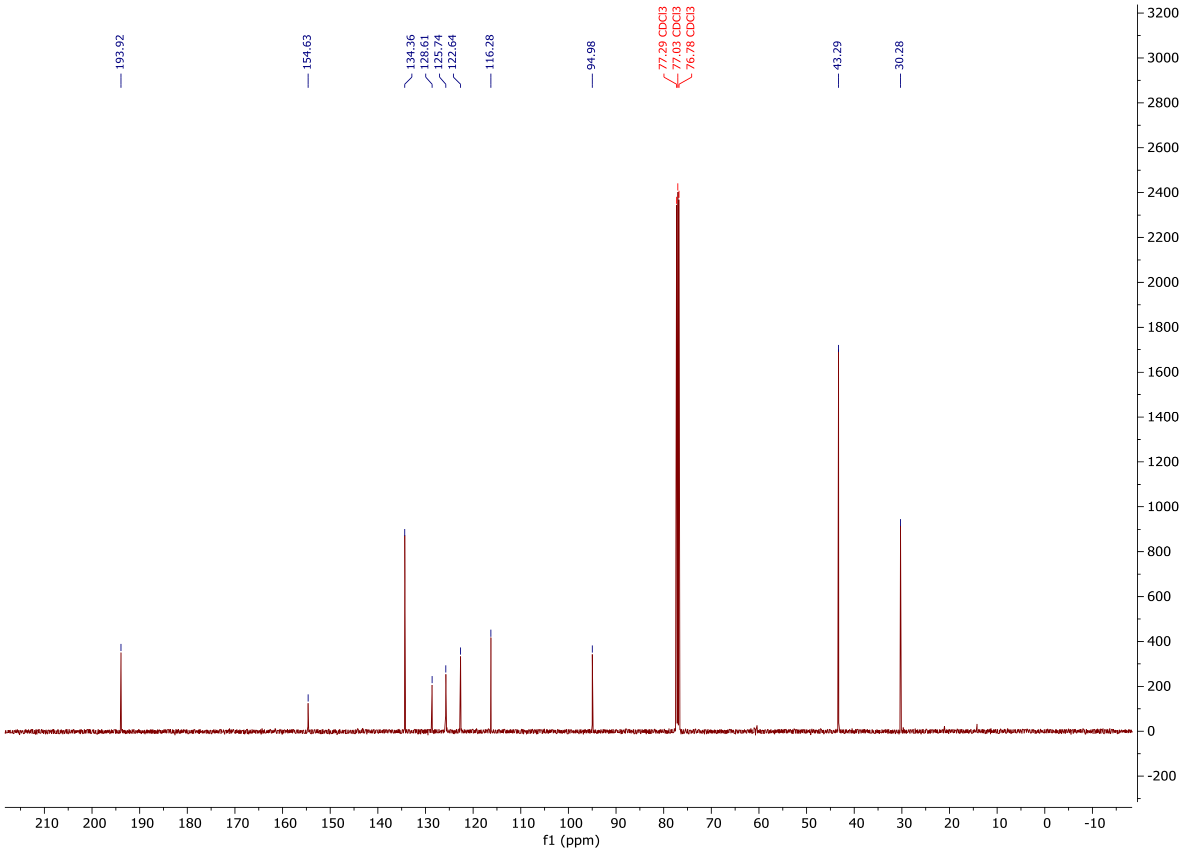

Figure S1.77: ^{13}C-NMR (126 MHz, $CDCl_3$) spectrum of 3a.

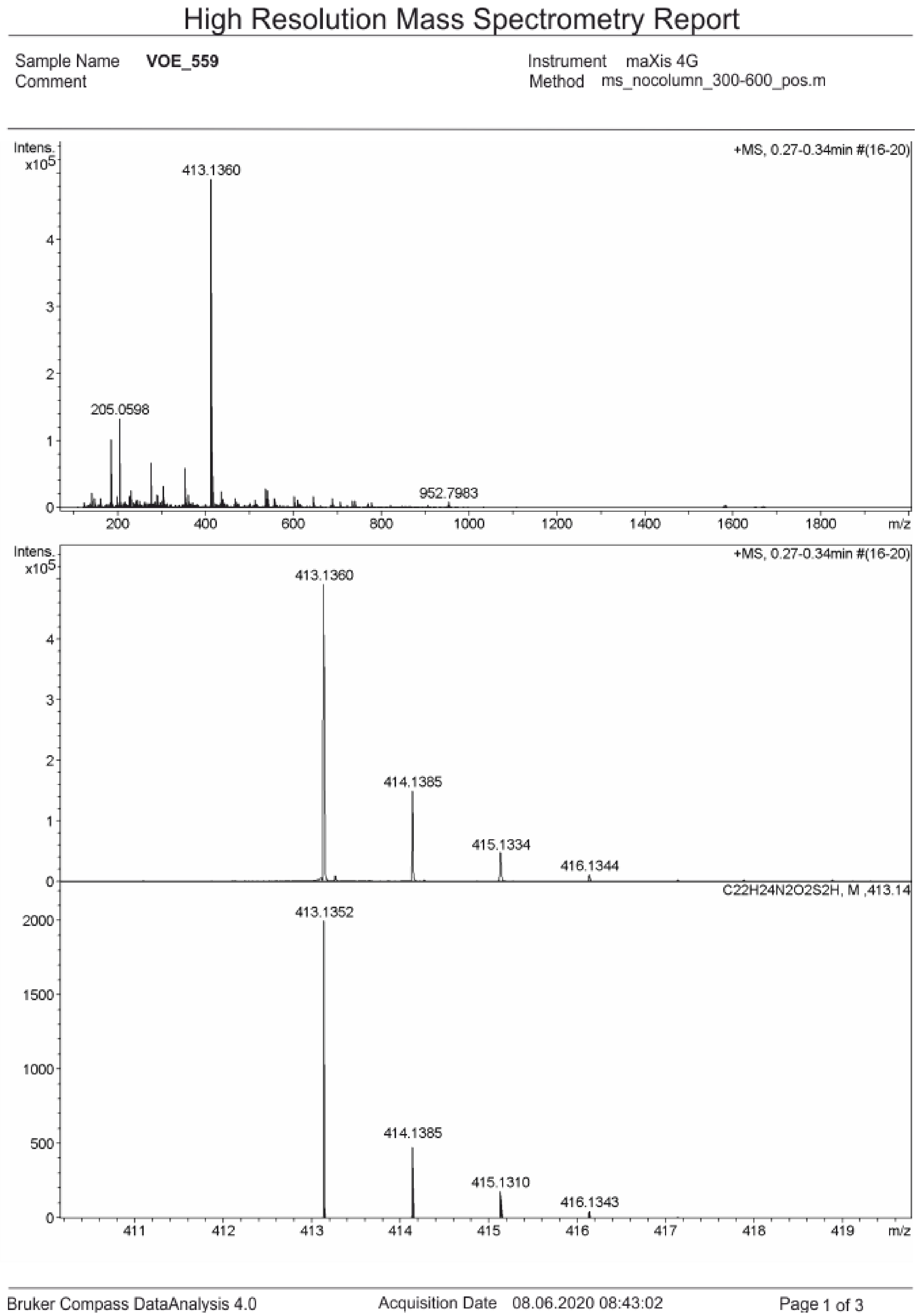

Figure S1.78: HRMS (ESI) spectrum of 3a.

High Resolution Mass Spectrometry Report

Measured m/z vs. theoretical m/z

Meas. m/z	#	Formula	Score	m/z	err [mDa]	err [ppm]	mSigma	rdb	e⁻ Conf	z
413.1360	1	C 22 H 25 N 2 O 2 S 2	100.00	413.1352	-0.8	-2.0	27.3	11.5	even	1+

Mass list

#	m/z	I %	I
1	123.0914	1.6	8060
2	137.1072	1.3	6574
3	140.9615	4.7	23107
4	147.0914	3.0	14834
5	157.0969	1.2	6080
6	161.1071	3.0	14554
7	183.0776	1.8	8725
8	185.1146	20.9	102630
9	186.1180	1.9	9433
10	187.1223	1.7	8487
11	189.0858	1.2	6084
12	199.1300	3.6	17541
13	201.1381	1.2	6134
14	205.0598	27.2	133789
15	206.0630	2.1	10244
16	213.1453	1.4	6757
17	214.9171	1.5	7612
18	215.1248	1.3	6449
19	217.1043	1.9	9565
20	226.9512	3.4	16744
21	229.0500	3.4	16802
22	229.1404	1.8	8972
23	229.8928	5.4	26400
24	235.9096	1.8	8635
25	236.0711	1.3	6420
26	239.0885	1.4	6729
27	243.1348	2.3	11258
28	244.8683	2.4	11803
29	245.0775	1.2	6105
30	251.0525	2.1	10340
31	259.1298	1.4	6862
32	261.1305	1.9	9244
33	261.1443	1.4	7028
34	262.0430	1.2	6057
35	267.0303	1.5	7478
36	271.1872	1.5	7364
37	273.1669	1.3	6248
38	275.1614	13.8	68048
39	276.1647	2.3	11339
40	277.1762	1.5	7186
41	282.0053	1.4	6785
42	285.8948	2.0	9637
43	288.9217	4.0	19890
44	291.1561	3.8	18675
45	297.8800	1.6	8025
46	301.1400	2.2	10703
47	303.8971	6.7	32697
48	305.1575	1.6	7695
49	305.1711	2.4	11704
50	312.8555	1.4	6656
51	353.1449	12.3	60362
52	354.1481	3.4	16556
53	360.3230	4.2	20573
54	365.8673	1.4	6779
55	370.1166	1.8	8625
56	400.2427	1.3	6209
57	413.1057	1.3	6463
58	413.1360	100.0	491556
59	413.2656	2.0	9789
60	414.1385	30.6	150584
61	415.1334	9.6	47313
62	416.1344	2.5	12432

Figure S1.79: HRMS (ESI) peak table of 3a.

High Resolution Mass Spectrometry Report

#	m/z	I %	I
63	422.2556	1.4	6927
64	433.8548	1.5	7190
65	435.1166	5.0	24799
66	436.1195	1.3	6316
67	439.1243	2.3	11212
68	439.8717	2.7	13244
69	444.2686	1.4	6905
70	448.8303	1.3	6325
71	466.2816	1.2	6103
72	467.1011	2.9	14387
73	468.1019	1.2	6090
74	469.3130	2.1	10080
75	475.0563	1.4	6912
76	501.8419	1.5	7400
77	513.1432	2.5	12474
78	513.3388	2.3	11102
79	536.1644	5.8	28688
80	537.1651	2.9	14127
81	538.1628	2.0	9814
82	541.1198	5.5	26862
83	542.1206	2.8	13802
84	543.1187	1.9	9465
85	557.0937	1.8	9026
86	557.3649	3.0	14740
87	601.3908	3.5	17354
88	602.3943	1.2	6121
89	610.1829	2.4	11771
90	611.1837	1.4	6761
91	615.1387	1.3	6169
92	645.4175	3.5	17006
93	646.4204	1.2	6122
94	689.4434	3.0	14599
95	705.5811	1.9	9546
96	733.4698	2.3	11248
97	739.2249	2.1	10348
98	769.8482	1.4	6773
99	777.4962	1.6	7942
100	952.7983	1.9	9135

Acquisition Parameter

**General**	Fore Vacuum<br>Scan Begin	2.39e+000 mBar<br>75 m/z	High Vacuum<br>Scan End	1.12e-007 mBar<br>2000 m/z	Source Type<br>Ion Polarity	ESI<br>Positive
**Source**	Set Nebulizer<br>Set Dry Heater	2.0 Bar<br>200 °C	Set Capillary<br>Set End Plate Offset	4500 V<br>-500 V	Set Dry Gas	8.0 l/min
**Quadrupole**	Set Ion Energy (MS only)	4.0 eV				
**Coll. Cell**	Collision Energy	8.0 eV	Set Collision Cell RF	600.0 Vpp		
**Ion Cooler**	Set Ion Cooler Transfer Time	75.0 µs	Set Ion Cooler Pre Pulse Storage Time	10.0 µs		

Figure S1.80: HRMS (ESI) peak table of 3a.

1.1 CV measurement

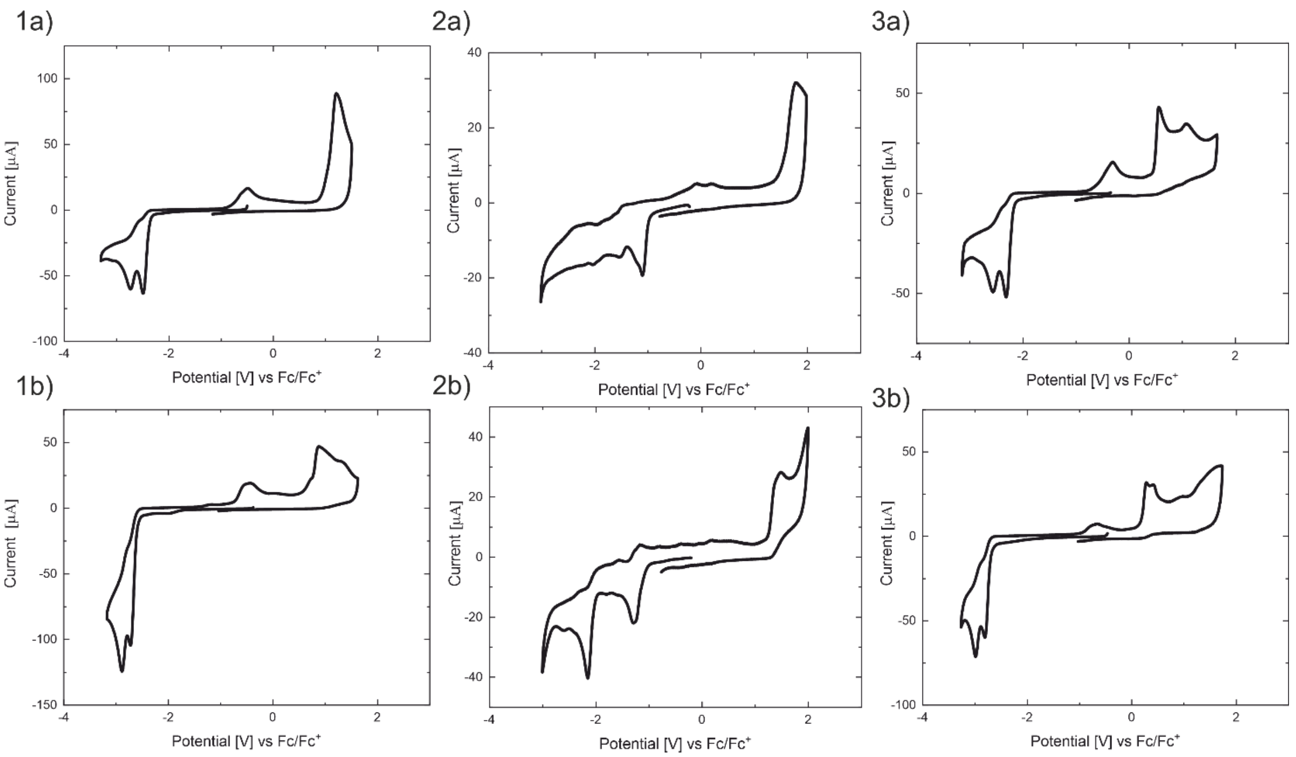

Figure S1.81. CV spectra of the 6 different tolanes (1a, 1b, 2a, 2b, 3a and 3b) recorded in dry deaerated CH_3CN with 0.1 M $TBAPF_6$ as electrolyte at a scan rate of 0.1 V/s and calibrated against the Fc/Fc^+ couple. The CV spectra of 1a, 2a, 3a and 3b were recorded at a molar concentration of 2 mM while the spectra of 1b was recorded at a concentration 2.5 mM and 2b was recorded at a concentration of 1.5 mM. 2b was recorded in a concentration of 1.5 mM due to its poor solubility. None of the recorded oxidation/reduction peaks showed reversibility at any scan rate. The irreversibility also causes artefacts to appear because of the irreversible nature of the reduction and oxidation processes. Therefore, the onset potential was used to determine the HOMO/LUMO levels of the compounds. The solvent acetonitrile was chosen such that it has a wide RED/OX window so all spectra could be recorded in the same solvent.

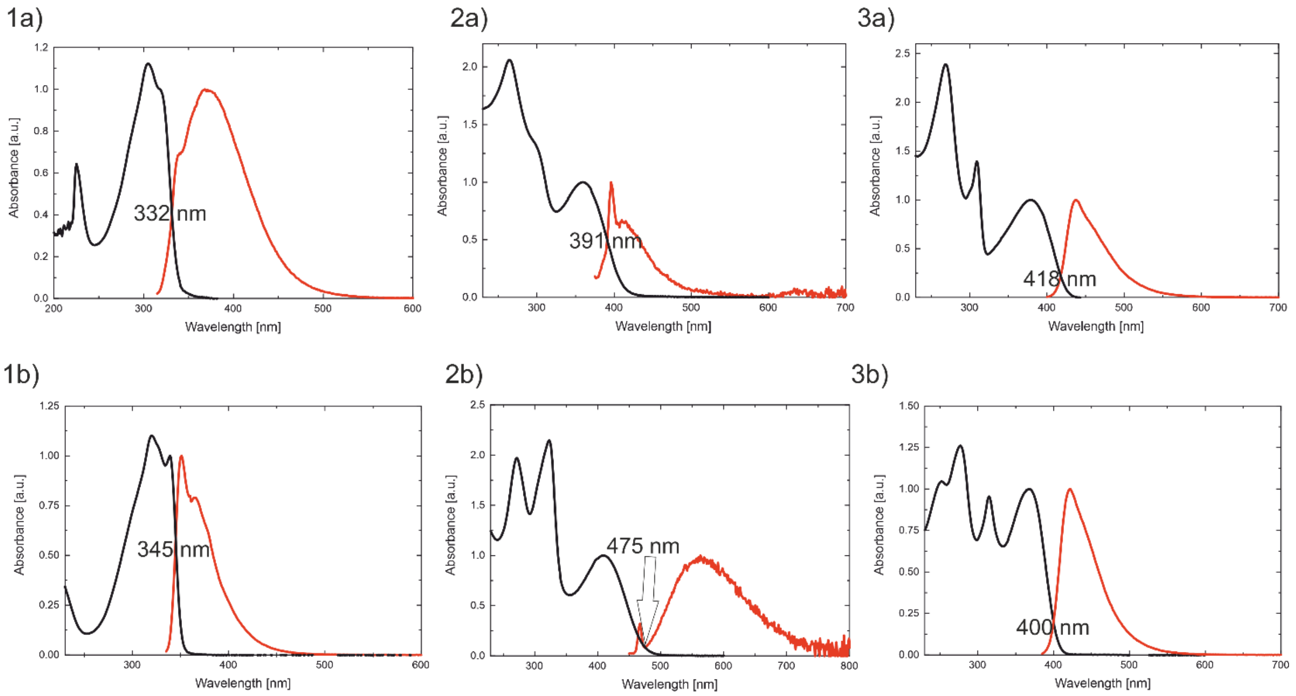

Figure S1.82. UV-Vis absorption and emission spectra of all 6 tolanes (1a, 1b, 2a, 2b, 3a, and 3b) in DCM: The black line shows the UV-Vis spectra while the red line shows the fluorescence spectra. All measurements were normalised to the longest (highest wavelength) absorption peak and the shortest emission peak respectively. The 0-0 transition of every spectra couple is given by the number displayed in each graph.

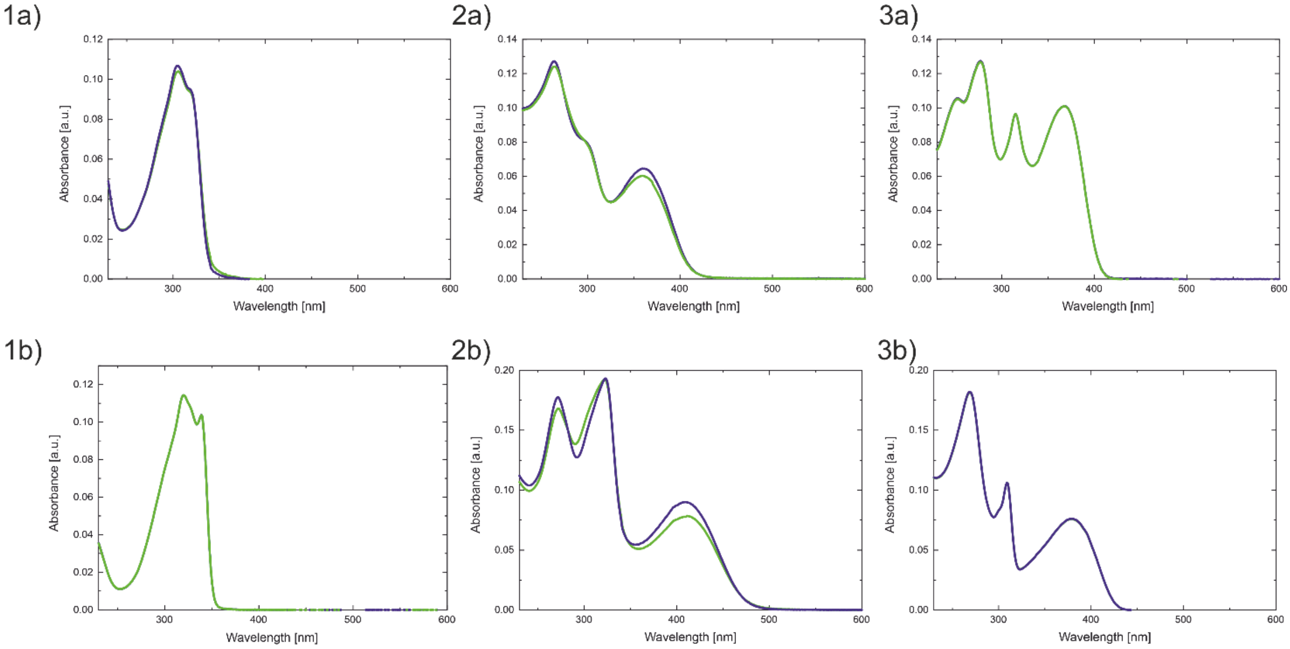

Figure S1.83. UV-Vis absorption measurement before (green) and after (blue) the florescence measurement (scan rate of 1000 nm /s excitation band with 5 nm using a xenon lamp, the excitation shutter was only opened during the measurement). The nitro derivatives 2a and 2b show clear degradation while the dimethyl amine derivatives 3a and 3b as well as the parent tolanes 1a and 1b seem to be photostable under the given conditions.

Table S1.1. Cyclic voltammetry data, their respective calculated HOMO/LUMO level and E_{gap}_CV, the 0-0 transition determined by UV-Vis, the respective E_{gap}_UV-Vis as well as the HOMO/LUMO level calculated using first principle methods and there resulting E_{gap} calc. The HOMO/LUMO level determined by CV were determined by an empirical relationship established in literature[1,2]. The 0-0 transition was used to determine the E_{gap}_UV-Vis according to Petr Klán[3].

Molecule	*E^{ox}_{onset} [V]*	*HOMO [eV]*	*E^{red}_{onset} [V]*	*LUMO [eV]*	*UV-Vis 0-0 transition [nm]*	*E_{gap}_UV-Vis [eV]*	*E_{gap}_CV [eV]*	*HOMO calc. [eV]*	*LUMO calc. [eV]*	*E_{gap} calc. [eV]*
1a	*0.94*	*-5.74*	*-2.34*	*-2.46*	*332*	*3.73*	*3.3*	*-4.49*	*-1.91*	*2.58*
1b	*0.70*	*-5.48*	*-2.57*	*-2.22*	*345*	*3.59*	*3.3*	*-4.28*	*-1.77*	*2.50*
2a	*1.51*	*-6.31*	*-0.98*	*-3.82*	*391*	*3.17*	*2.5*	*-5.00*	*-3.27*	*1.72*
2b	*1.24*	*-6.04*	*-1.04*	*-3.76*	*475*	*2.61*	*2.3*	*-4.77*	*-3.16*	*1.60*
3a	*0.41*	*-5.21*	*-2.14*	*-2.66*	*418*	*2.97*	*2.6*	*-3.79*	*-1.49*	*2.29*
3b	*0.17*	*-4.97*	*-2.65*	*-2.15*	*400*	*3.1*	*2.8*	*-3.63*	*-1.35*	*2.27*

2 MCBJ measurements and data analysis

For the classification of empty traces, the feature space was defined as follows. The 2D-histogram of the trace computed on a 25x32 grid, with limits set to -0.5nm to 3nm in displacement and -0.5 to -6 log(G/G_0) in conductance. Rows are then concatenated to each other to obtain an 800 dimensional vector. The 1D histogram of the trace with 100 bins and limits 1 to -6 log(G/G_0) was also appended to the previous vector, for a total of 900 dimensions.

The fully-connected neural network used for classification consists of an input layer with 900 nodes, 2 hidden layer having 128 and 64 nodes respectively, and an output layer of 2 dimensions (tunnelling vs. molecular). A 30% dropout rate was used between each hidden layer to prevent overfitting. Additionally, a Rectified Linear Unit (ReLU) function was applied to the output of each hidden layer, while a Softmax function was used on the output nodes. When a trace is input into the network, it will result in two scalar values, which can be interpreted as the probability of the trace being empty, and the probability of it being molecular. If the output value of the trace being molecular is >0.5, while the probability of it being empty is <0.5, the trace is classified as molecular.

The network was then trained using the adaptive moment estimation (Adam) algorithm[4] on a set of around 200.000 hand-classified traces containing a roughly equal amount of empty traces and molecular traces (where the molecular traces consisted of alkanes of different lengths: propanedithiol, hexanedithiol, and octanedithiol). A 4:1 ratio between training and validation set was used.

Each dataset measured for this study was first run through the neural network described above, which yielded a class of "empty" traces for each measurement. Subsequently, the remaining "molecular" traces were further classified using k-means++, according to a previously published method[5]. Note that the feature space used in this study consists of a 30x40 bins 2D histogram, with limits set as 0nm to 1.5nm in displacement, and -1 to -6 log(G/G_0) in conductance. The 1D histogram of the trace with 100 bins and limits -1 to -6.5 log(G/G_0) was also appended to the previous vector, for a total of 1300 dimensions.

2.1 Measurement Overview

In this section it is possible to find tables summarising the results of all measurements for each molecule, as well as the 1D-conductance and the 2D-conductance/displacement histograms obtained from the raw data and from the classification analysis explained in the method section.

Table S2.1. Summary table for the results of molecule **1a** measurements; The columns report the total number of traces in the measurement, the conductance extracted through fitting the raw data, the percentage of traces that were classified as molecular by the Neural Network (Yield); then, the conductance and yield for HC, LC1, and LC2 classes are reported. Note that the yield is reported with respect to the total number of traces.

Measurement	*N traces*	*Raw Data conductance (G_0)*	*Yield (%)*	*HC conductance (G_0)*	*HC Yield (%)*	*LC1 conductance (G_0)*	*LC1 Yield (%)*	*LC2 conductance (G_0)*	*LC2 Yield (%)*
1	1000	$1.2 \cdot 10^{-3}$	43	$1.2 \cdot 10^{-3}$	14	$1.4 \cdot 10^{-5}$	10	$2.0 \cdot 10^{-6}$	4
2	2482	$1.0 \cdot 10^{-3}$	36	$1.1 \cdot 10^{-3}$	10	$1.6 \cdot 10^{-5}$	9	$2.0 \cdot 10^{-6}$	3

Table S2.2. Summary table for the results of molecule **2a** measurements; The columns report the total number of traces in the measurement, the conductance extracted through fitting the raw data, the percentage of traces that were classified as molecular by the Neural Network (Yield); then, the conductance and yield for HC, $LC1^H$, and $LC1^L$ classes are reported.

Note that the yield is reported with respect to the total number of traces.

Measurement	*N traces*	*Raw Data conductance (G_0)*	*Yield (%)*	*HC conductance (G_0)*	*HC Yield (%)*	*$LC1^H$ conductance (G_0)*	*$LC1^H$ Yield (%)*	*$LC1^L$ conductance (G_0)*	*$LC1^L$ Yield (%)*
1	10000	$2.3 \cdot 10^{-5}$	23	$1.1 \cdot 10^{-3}$	5	$2.0 \cdot 10^{-4}$	8	$3.0 \cdot 10^{-5}$	10
2	10000	$3.8 \cdot 10^{-5}$	38	$1.5 \cdot 10^{-3}$	3	$3.4 \cdot 10^{-4}$	5	$4.3 \cdot 10^{-5}$	30
3	10000	$2.2 \cdot 10^{-5}$	30	$1.8 \cdot 10^{-3}$	2	$2.1 \cdot 10^{-4}$	4	$2.5 \cdot 10^{-5}$	28

Table S2.3. Summary table for the results of molecule **3a** measurements; The columns report the total number of traces in the measurement, the conductance extracted through fitting the raw data, the percentage of traces that were classified as molecular by the Neural Network (Yield); then, the conductance and yield for HC, LC1, and LC2 classes are reported. Note that the yield is reported with respect to the total number of traces.

Measurement	*N traces*	*Raw Data conductance (G_0)*	*Yield (%)*	*HC conductance (G_0)*	*HC Yield (%)*	*LC1 conductance (G_0)*	*LC1 Yield (%)*	*LC2 conductance (G_0)*	*LC2 Yield (%)*
1	2218	$1.7 \cdot 10^{-3}$	55	$2.0 \cdot 10^{-3}$	32	$6.7 \cdot 10^{-5}$	19	$9.7 \cdot 10^{-7}$	4
2	2230	$2.2 \cdot 10^{-3}$	64	$2.7 \cdot 10^{-3}$	28	//	//	//	//
3	10000	$1.6 \cdot 10^{-3}$	42	$2.0 \cdot 10^{-3}$	27	$5.3 \cdot 10^{-5}$	13	$3.0 \cdot 10^{-6}$	2.3

Table S2.4. Summary table for the results of molecule **1b** measurements; The columns report the total number of traces in the measurement, the conductance extracted through fitting the raw data, the percentage of traces that were classified as molecular by the Neural Network (Yield); then, the conductance and yield for HC, LC1, and LC2 classes are reported. Note that the yield is reported with respect to the total number of traces.

Measurement	*N traces*	*Raw Data conductance (G_0)*	*Yield (%)*	*HC conductance (G_0)*	*HC Yield (%)*	*LC conductance (G_0)*	*LC Yield (%)*
1	14994	$3.3 \cdot 10^{-4}$	51	$4.7 \cdot 10^{-4}$	24	$1.8 \cdot 10^{-5}$	27
2	86885	$3.6 \cdot 10^{-4}$	87	$5.4 \cdot 10^{-4}$	40	$1.9 \cdot 10^{-5}$	47
3	33368	$4.6 \cdot 10^{-4}$	77	$5.0 \cdot 10^{-4}$	37	$9.0 \cdot 10^{-6}$	40
4	10000	$6.8 \cdot 10^{-4}$	96	$6.9 \cdot 10^{-4}$	40	$1.2 \cdot 10^{-5}$	56
5	10000	$7.2 \cdot 10^{-4}$	97	$7.5 \cdot 10^{-4}$	69	$9.6 \cdot 10^{-6}$	28
6	10000	$5.2 \cdot 10^{-4}$	92	$5.7 \cdot 10^{-4}$	69	$9.1 \cdot 10^{-6}$	23

Table S2.5. Summary table for the results of molecule **2b** measurements; The columns report the total number of traces in the measurement, the conductance extracted through fitting the raw data, the percentage of traces that were classified as molecular by the Neural Network (Yield); then, the conductance and yield for HC, LC1, and LC2 classes are reported. Note that the yield is reported with respect to the total number of traces.

Measurement	*N traces*	*Raw Data conductance (G_0)*	*Yield (%)*	*HC conductance (G_0)*	*HC Yield (%)*	*LC conductance (G_0)*	*LC Yield (%)*
1	14994	$7.9 \cdot 10^{-4}$	90	$8.6 \cdot 10^{-4}$	34	$2.5 \cdot 10^{-5}$	56

2	86885	$4.9 \cdot 10^{-4}$	63	$5.1 \cdot 10^{-4}$	19	$1.9 \cdot 10^{-5}$	20
3	33368	$6.0 \cdot 10^{-4}$	65	$6.7 \cdot 10^{-4}$	23	$1.2 \cdot 10^{-5}$	26
4	10000	$6.9 \cdot 10^{-4}$	80	$7.3 \cdot 10^{-4}$	34	$1.8 \cdot 10^{-5}$	46
5	10000	$6.6 \cdot 10^{-4}$	82	$6.8 \cdot 10^{-4}$	26	$2.1 \cdot 10^{-5}$	56
6	10000	$7.2 \cdot 10^{-4}$	51	$7.3 \cdot 10^{-4}$	22	$1.4 \cdot 10^{-5}$	29

Table S2.6. Summary table for the results of molecule **3b** measurements; The columns report the total number of traces in the measurement, the conductance extracted through fitting the raw data, the percentage of traces that were classified as molecular by the Neural Network (Yield); then, the conductance and yield for HC, LC1, and LC2 classes are reported. Note that the yield is reported with respect to the total number of traces.

Measurement	*N traces*	*Raw Data conductance (G_0)*	*Yield (%)*	*HC conductance (G_0)*	*HC Yield (%)*	*LC conductance (G_0)*	*LC Yield (%)*
1	10000	$8.0 \cdot 10^{-4}$	94	$8.7 \cdot 10^{-4}$	64	$1.5 \cdot 10^{-5}$	30
2	10000	$7.4 \cdot 10^{-4}$	74	$7.6 \cdot 10^{-4}$	31	$8.2 \cdot 10^{-6}$	43
3	10000	$7.4 \cdot 10^{-4}$	85	$8.0 \cdot 10^{-4}$	27	$7.3 \cdot 10^{-6}$	58
4	10000	$6.0 \cdot 10^{-4}$	73	$6.5 \cdot 10^{-4}$	30	$1.3 \cdot 10^{-5}$	43
5	10000	$4.8 \cdot 10^{-4}$	85	$5.6 \cdot 10^{-4}$	30	$9.6 \cdot 10^{-6}$	55

2.1.1 -SAc Anchoring Group Measurements' Histograms

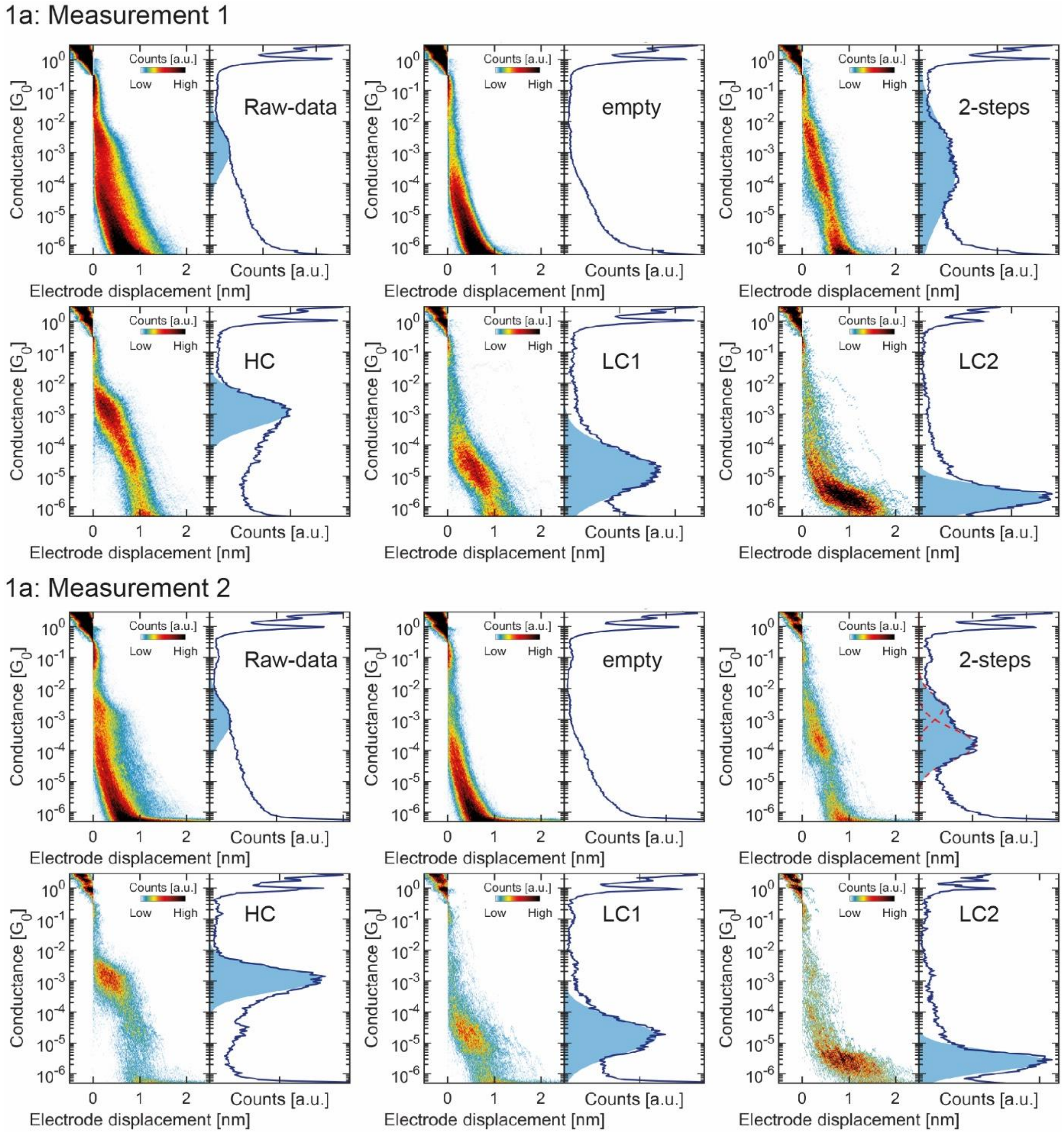

Figure S2.1. 2D and 1D histograms for measurement 1 and 2 of molecule 1a; In addition to the data before any classification, the histograms are reported for the traces excluded using the neural network ("empty") and for the classes identified with clustering analysis (HC, LC1, and LC2) . When present, the light-blue shaded area corresponds to the log-normal fit performed on the corresponding 1D-histogram.

2a: Measurement 1

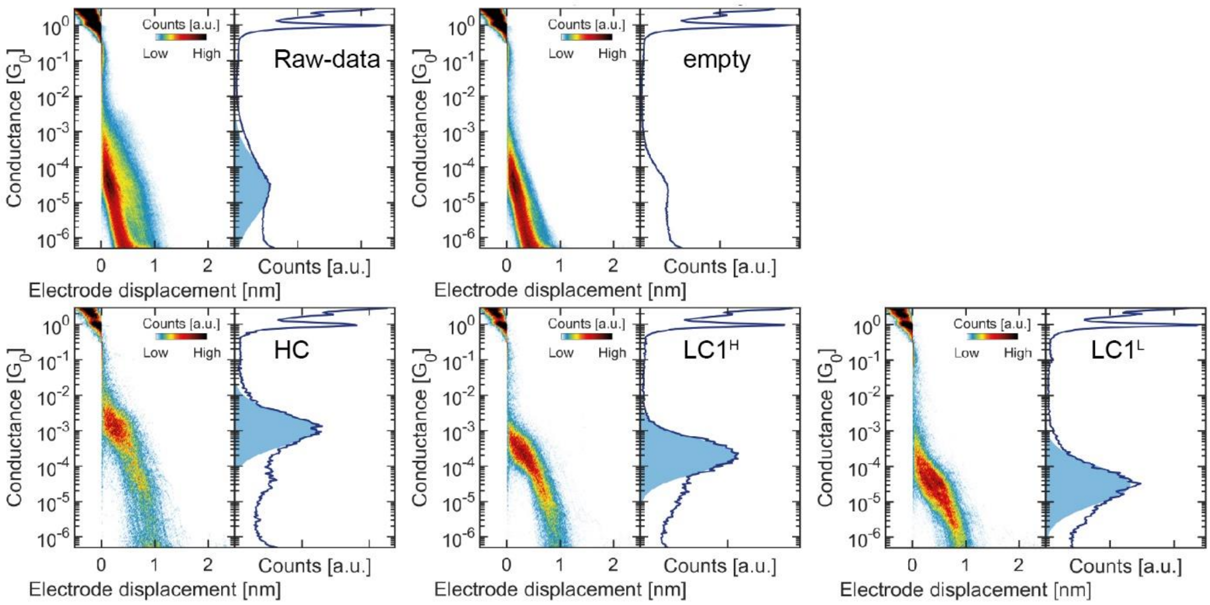

2a: Measurement 2

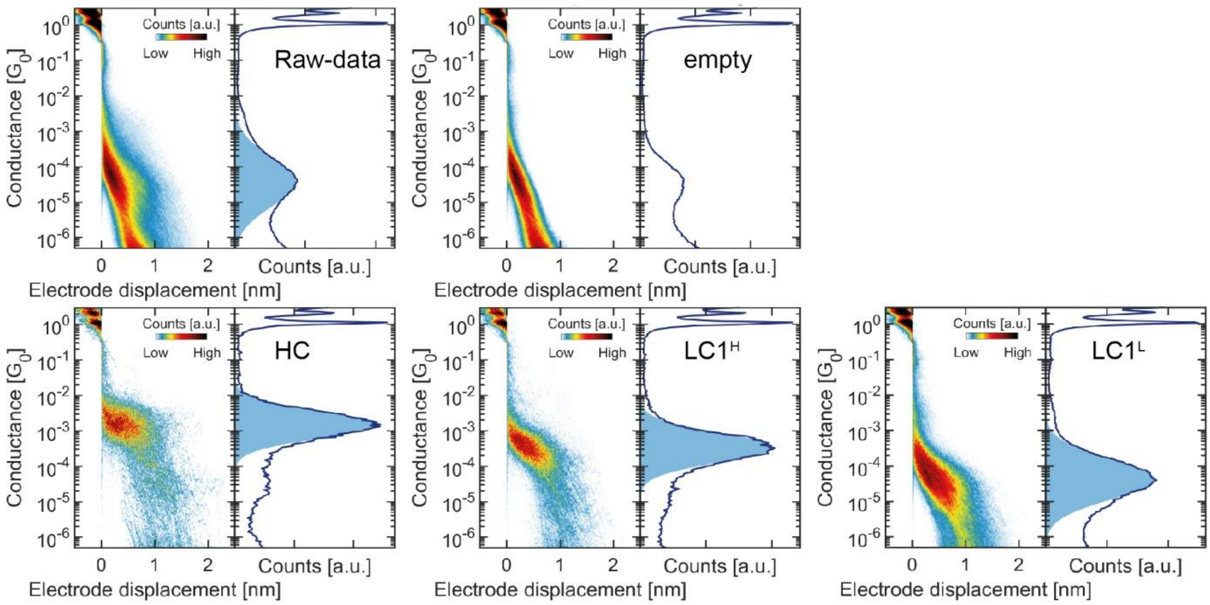

Figure S2.2. 2D and 1D histograms for measurement 1 and 2 of molecule 2a; In addition to the data before any classification, the histograms are reported for the traces excluded using the neural network ("empty") and for the classes identified with clustering analysis (HC, LC1^{H} and LC1^{L}) . When present, the light-blue shaded area corresponds to the log-normal fit performed on the corresponding 1D-histogram.

2a: Measurement 3

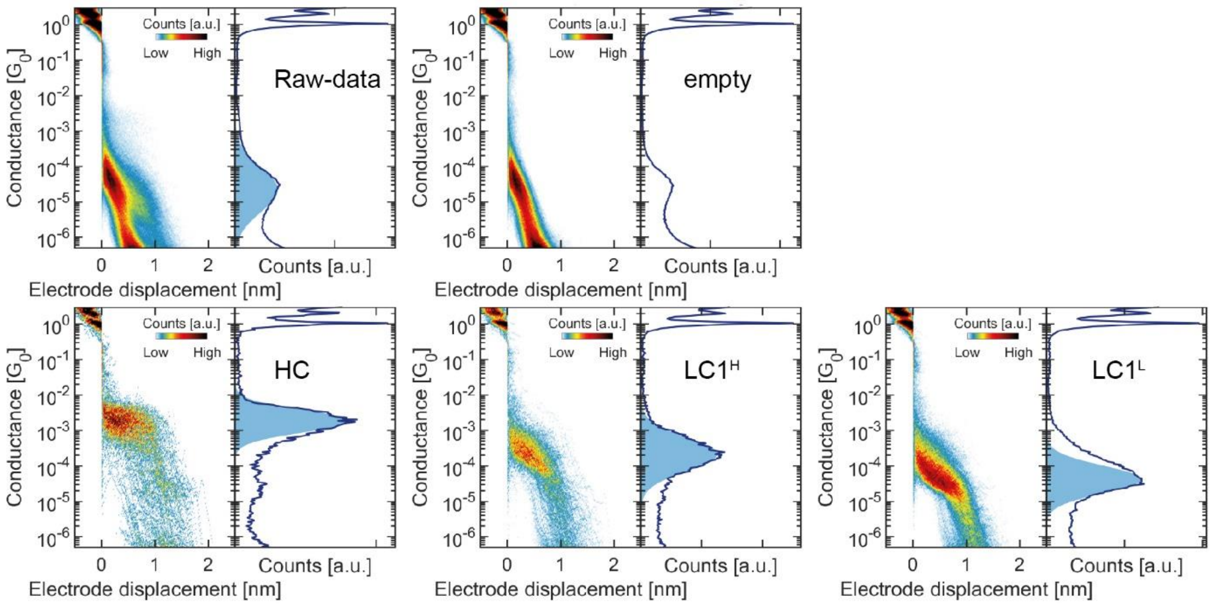

Figure S2.3. 2D and 1D histograms for measurement 3 of molecule 2a; In addition to the data before any classification, the histograms are reported for the traces excluded using the neural network ("empty") and for the classes identified with clustering analysis (HC, $LC1^H$ and $LC1^L$) . When present, the light-blue shaded area corresponds to the log-normal fit performed on the corresponding 1D-histogram.

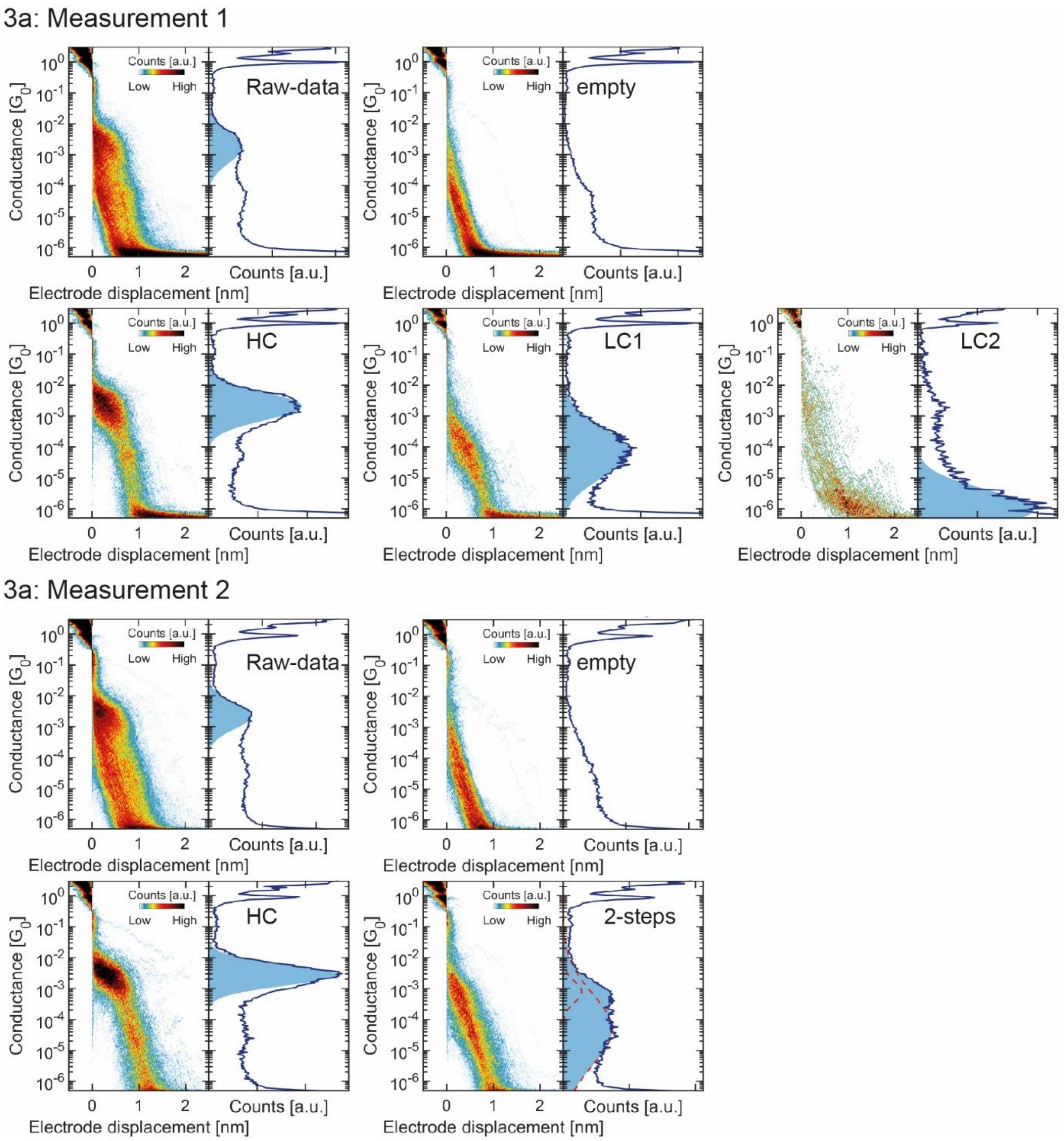

Figure S2.4. 2D and 1D histograms for measurement 1 and 2 of molecule 3a; In addition to the data before any classification, the histograms are reported for the traces excluded using the neural network ("empty") and for the classes identified with clustering analysis (HC, LC1, and LC2). When present, the light-blue shaded area corresponds to the log-normal fit performed on the corresponding 1D-histogram. Note that in measurement 2 it was not possible to identify clearly the low-conductance classes. Instead, a class containing traces with two steps was found.

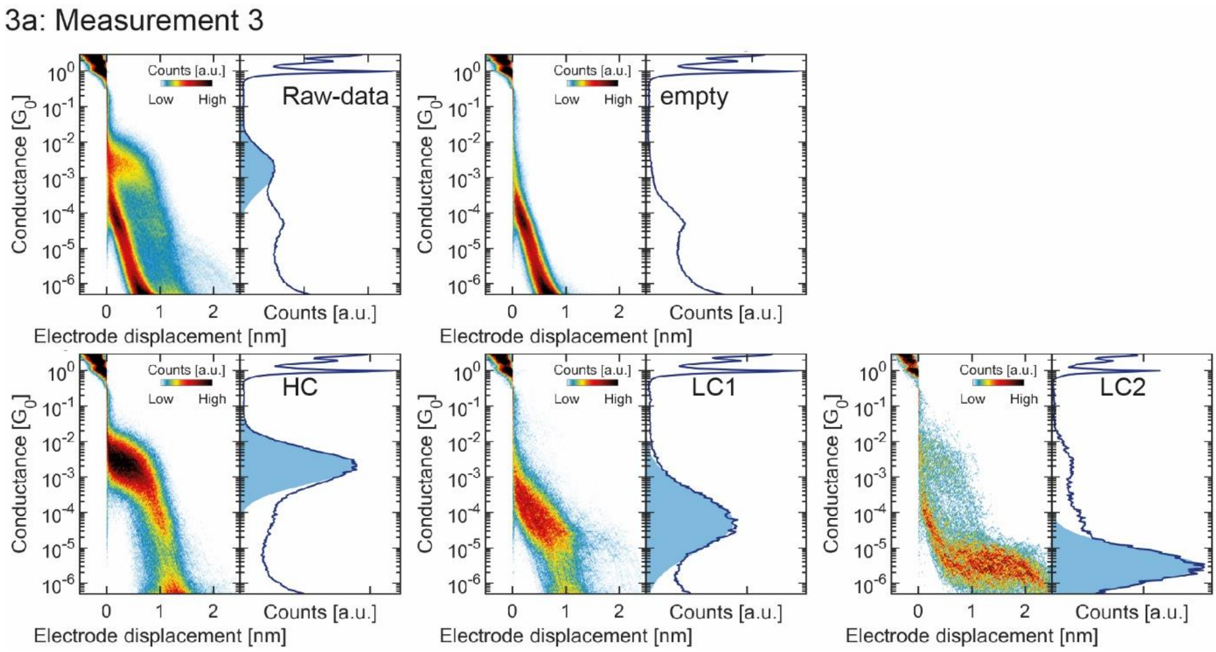

Figure S2.5. 2D and 1D histograms for measurement 3 of molecule 3a; In addition to the data before any classification, the histograms are reported for the traces excluded using the neural network ("empty") and for the classes identified with clustering analysis (HC, LC1, and LC2). When present, the light-blue shaded area corresponds to the log-normal fit performed on the corresponding 1D-histogram.

2.1.2 -SMe Anchoring Group Measurements' Histograms

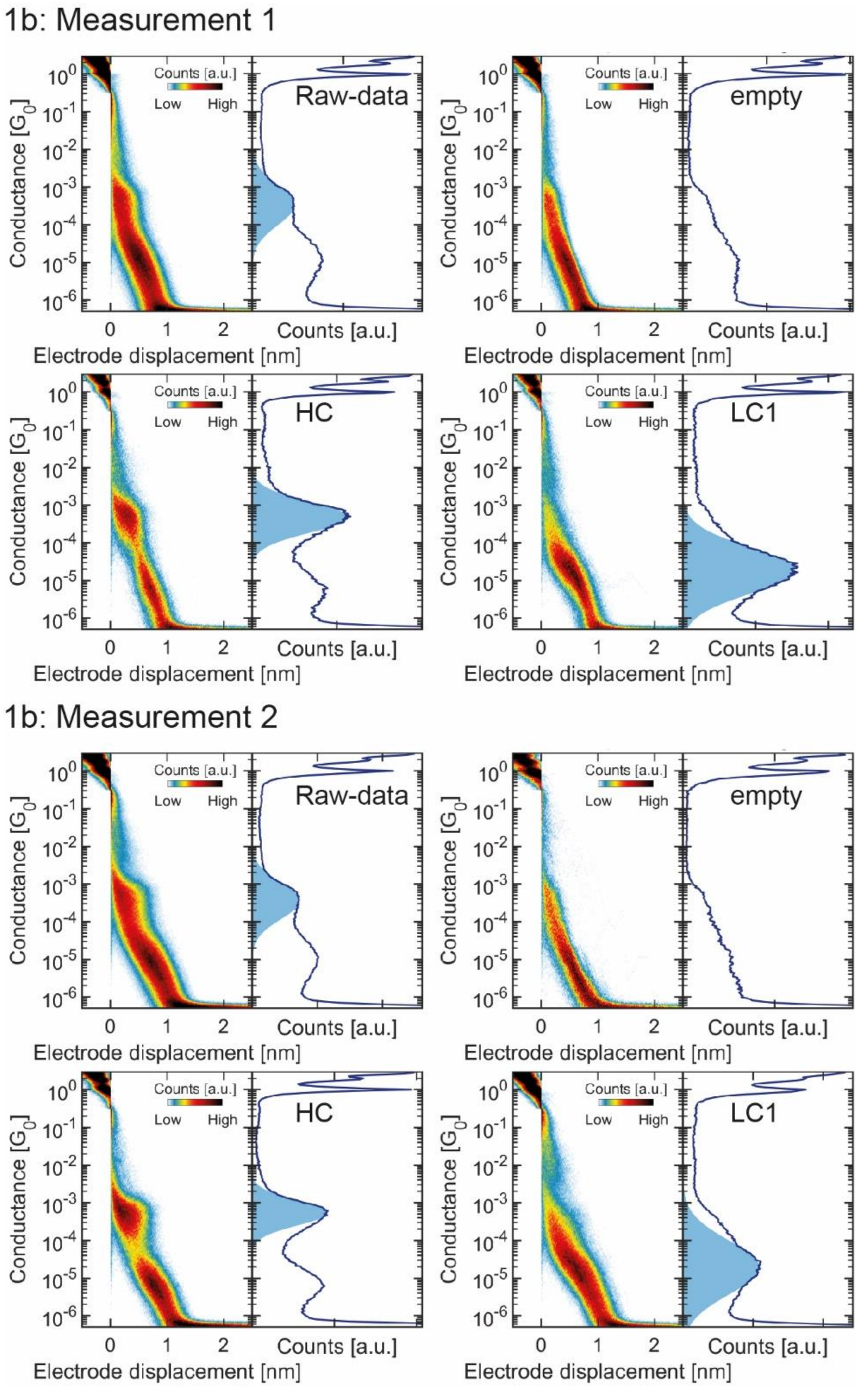

Figure S2.6. 2D and 1D histograms for measurement 1 and 2 of molecule 1b; In addition to the data before any classification, the histograms are reported for the traces excluded using the neural network ("empty") and for the classes identified with clustering analysis (HC, and LC1). When present, the light-blue shaded area corresponds to the log-normal fit performed on the corresponding 1D-histogram.

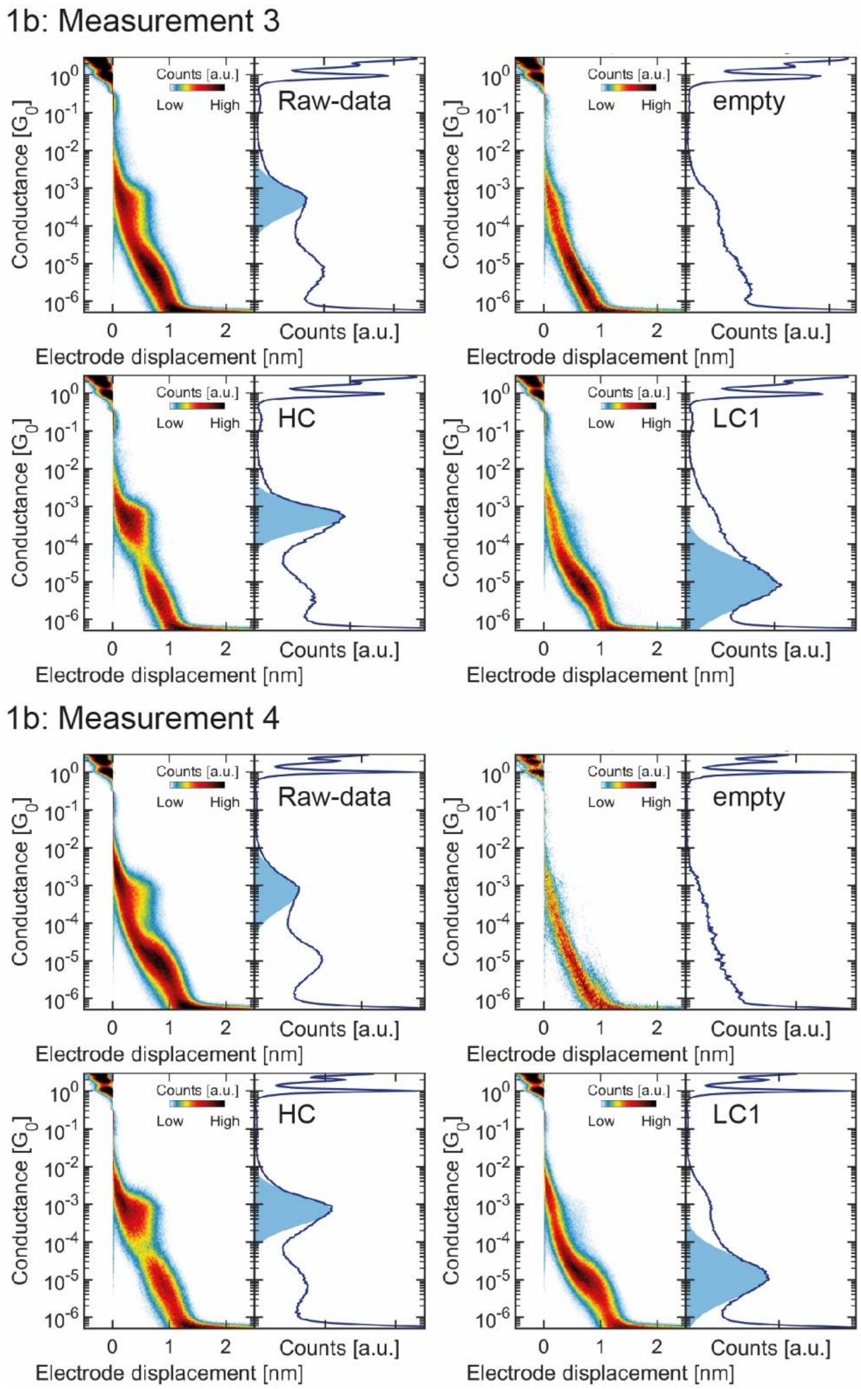

Figure S2.7. 2D and 1D histograms for measurement 3 and 4 of molecule 1b; In addition to the data before any classification, the histograms are reported for the traces excluded using the neural network ("empty") and for the classes identified with clustering analysis (HC, and LC1). When present, the light-blue shaded area corresponds to the log-normal fit performed on the corresponding 1D-histogram.

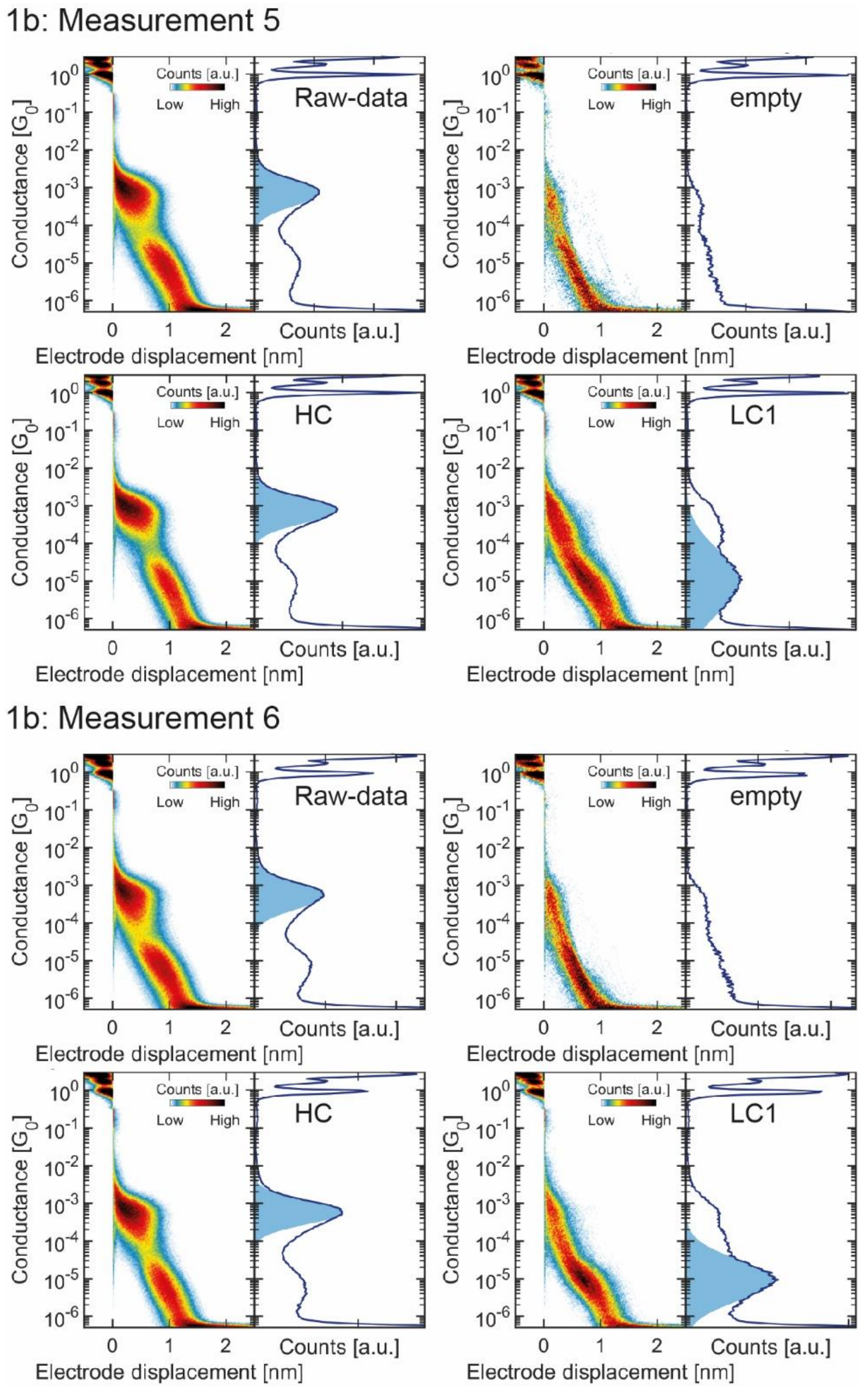

Figure S2.8. 2D and 1D histograms for measurement 5 and 6 of molecule 1b; In addition to the data before any classification, the histograms are reported for the traces excluded using the neural network ("empty") and for the classes identified with clustering analysis (HC, and LC1). When present, the light-blue shaded area corresponds to the log-normal fit performed on the corresponding 1D-histogram.

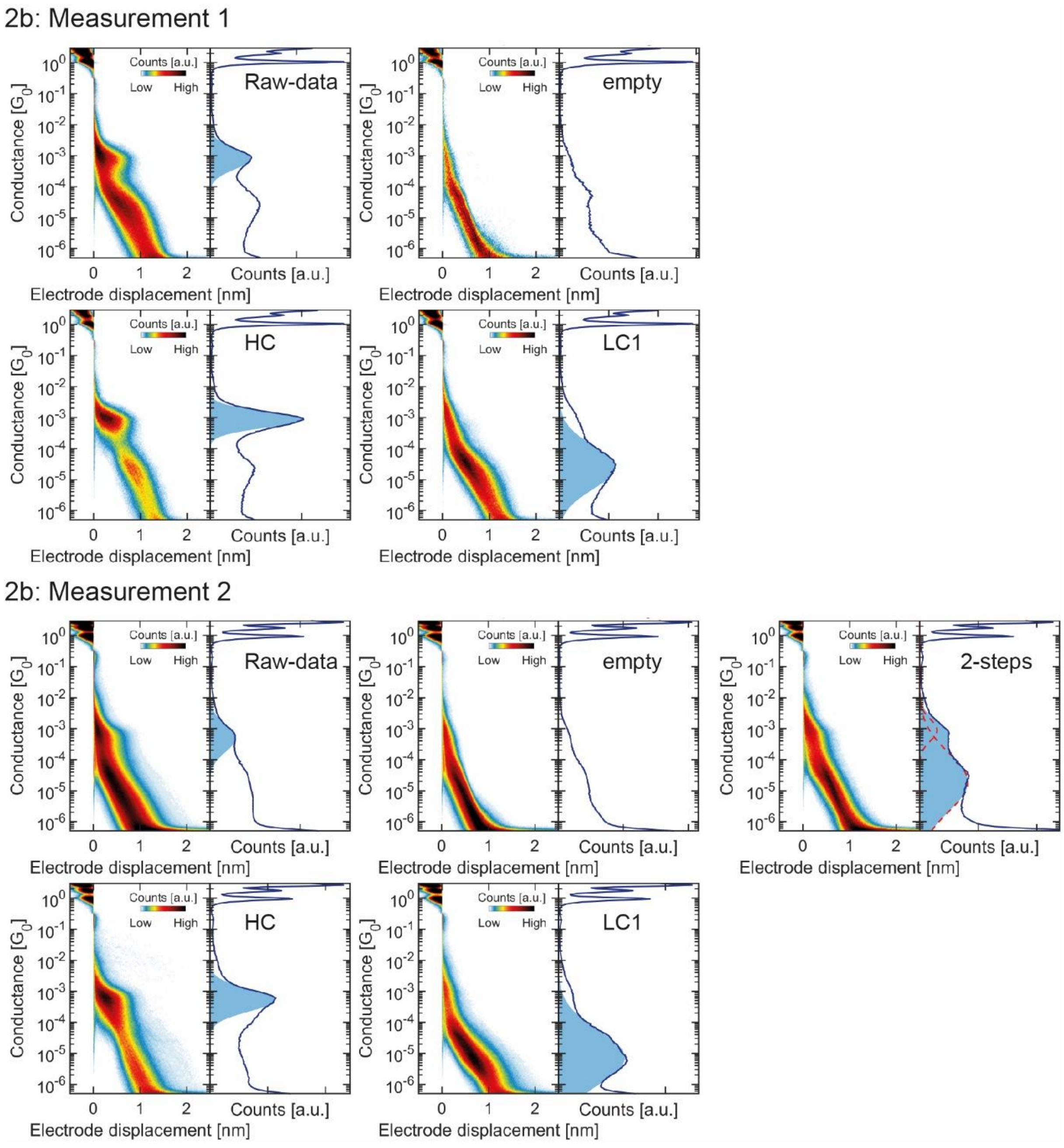

Figure S2.9. 2D and 1D histograms for measurement 1 and 2 of molecule 2b; In addition to the data before any classification, the histograms are reported for the traces excluded using the neural network ("empty") and for the classes identified with clustering analysis (HC, and LC1). When present, the light-blue shaded area corresponds to the log-normal fit performed on the corresponding 1D-histogram. Note that measurement 2 also shows a class containing traces with multiple steps. This class was not consistently identified across measurements.

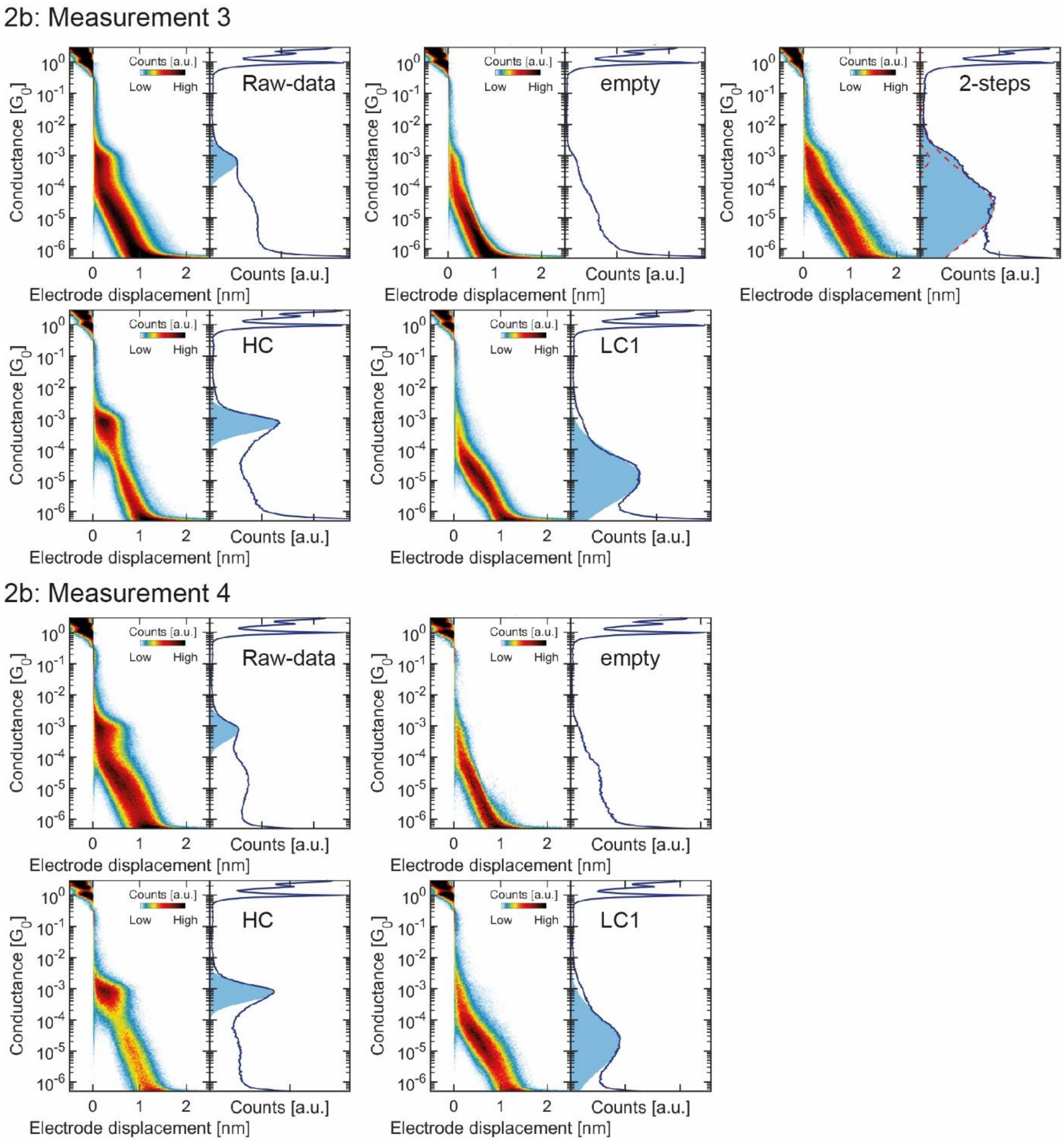

Figure S2.10. 2D and 1D histograms for measurement 3 and 4 of molecule 2b; In addition to the data before any classification, the histograms are reported for the traces excluded using the neural network ("empty") and for the classes identified with clustering analysis (HC, and LC1). When present, the light-blue shaded area corresponds to the log-normal fit performed on the corresponding 1D-histogram. Note that measurement 3 also shows a class containing traces with multiple steps. This class was not consistently identified across measurements.

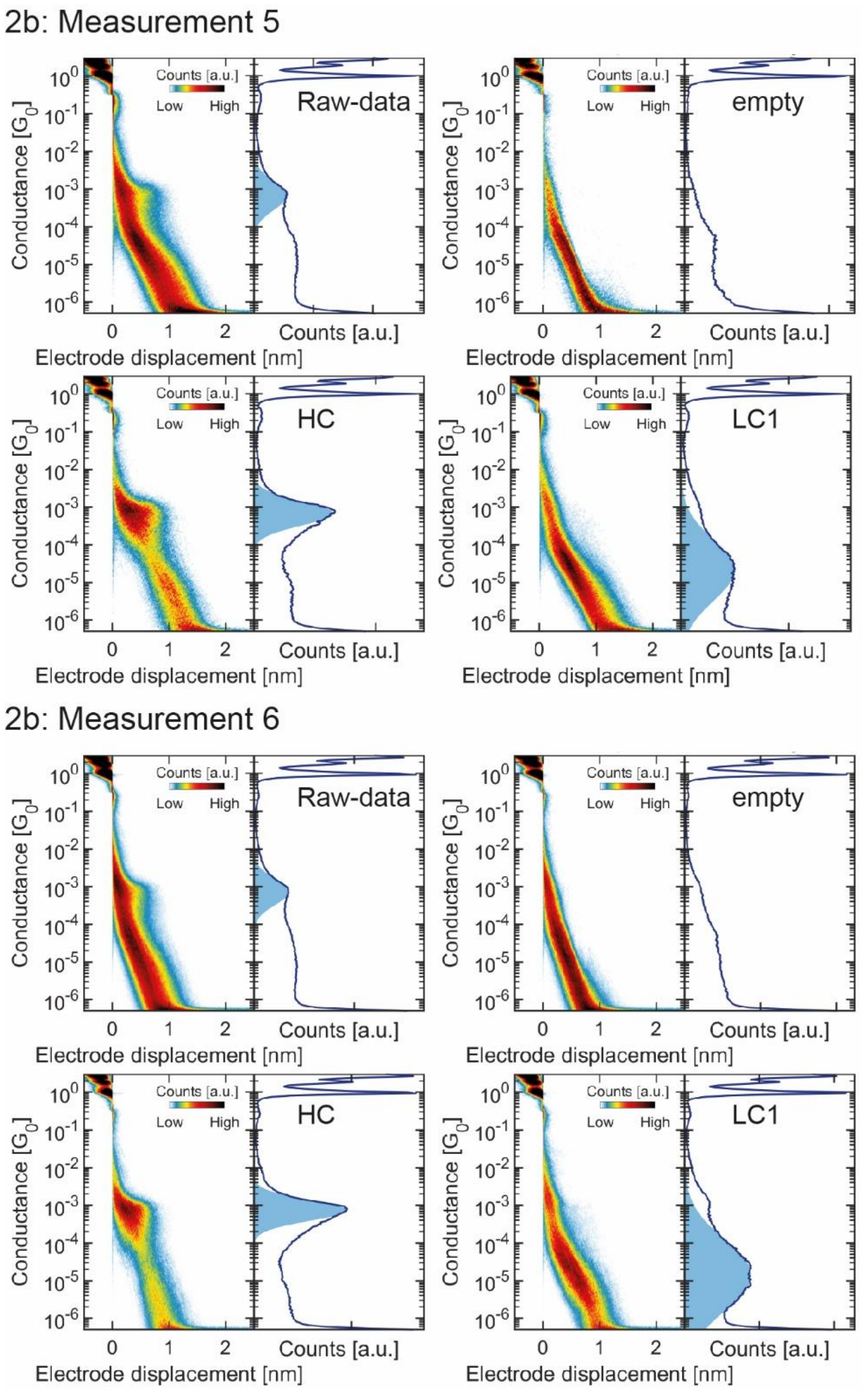

Figure S2.11. 2D and 1D histograms for measurement 5 and 6 of molecule 2b; In addition to the data before any classification, the histograms are reported for the traces excluded using the neural network ("empty") and for the classes identified with clustering analysis (HC, and LC1). When present, the light-blue shaded area corresponds to the log-normal fit performed on the corresponding 1D-histogram.

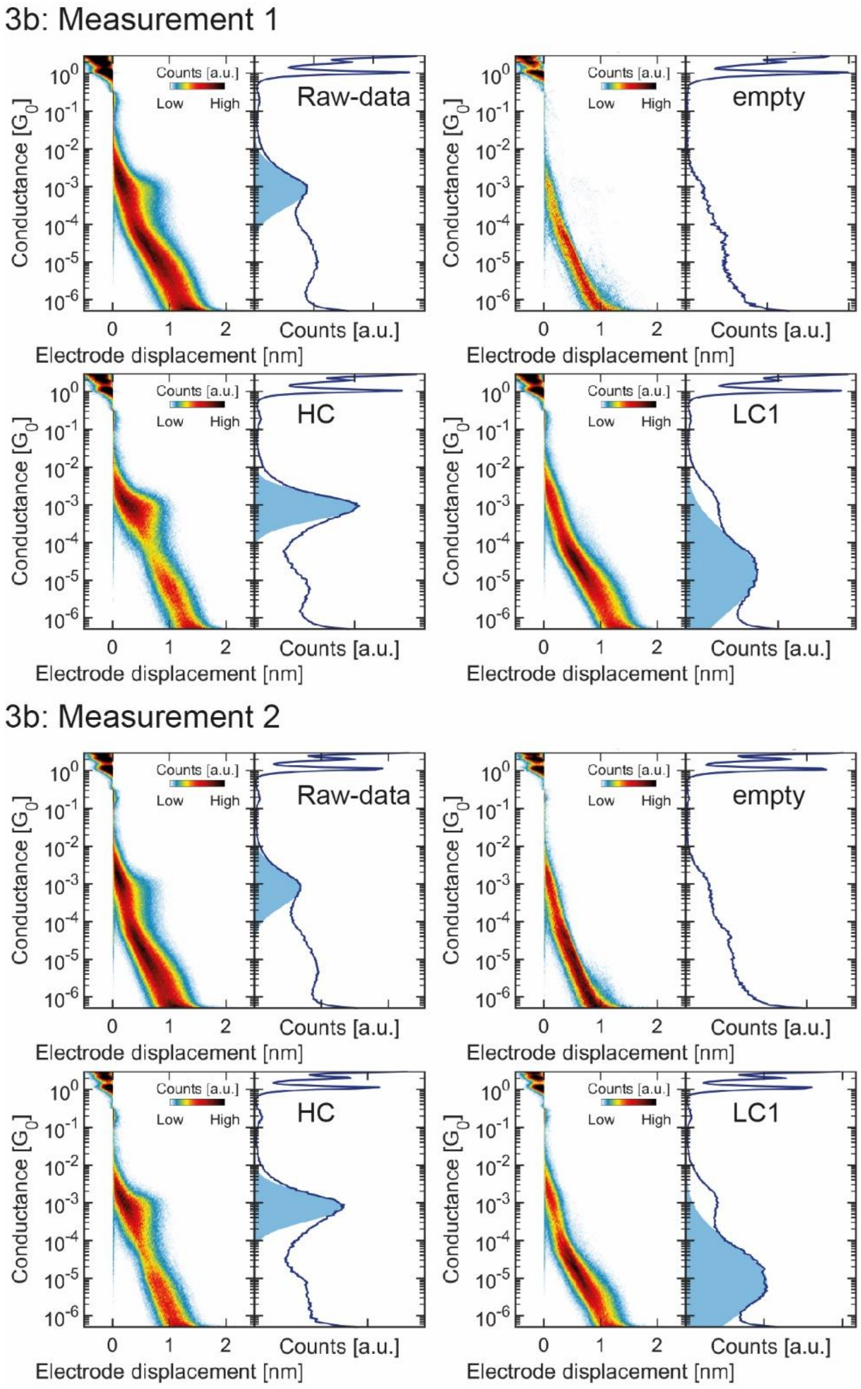

Figure S2.12. 2D and 1D histograms for measurement 1 and 2 of molecule 3b; In addition to the data before any classification, the histograms are reported for the traces excluded using the neural network ("empty") and for the classes identified with clustering analysis (HC, and LC1). When present, the light-blue shaded area corresponds to the log-normal fit performed on the corresponding 1D-histogram.

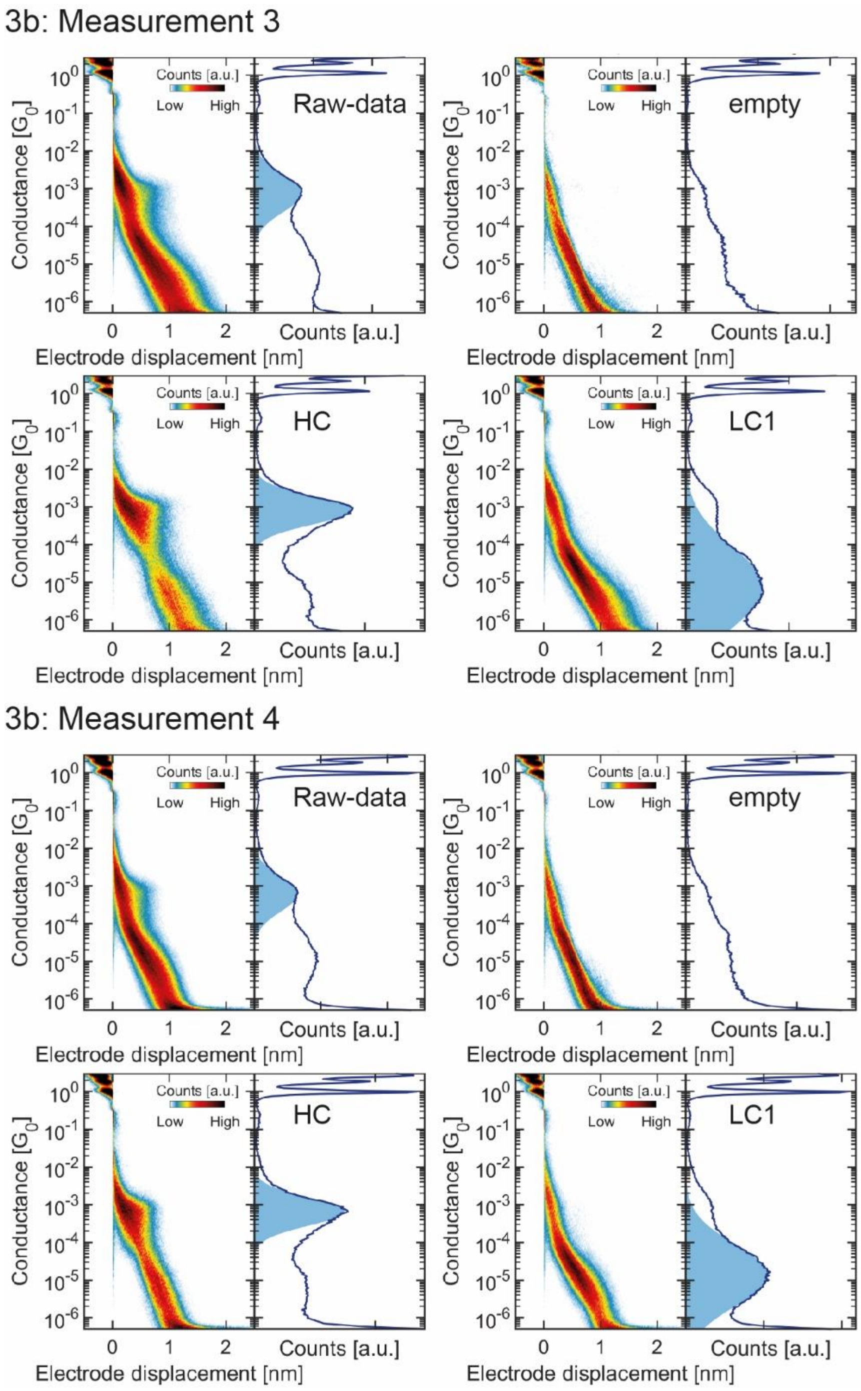

Figure S2.13. 2D and 1D histograms for measurement 3 and 4 of molecule 3b; In addition to the data before any classification, the histograms are reported for the traces excluded using the neural network (“empty”) and for the classes identified with clustering analysis (HC, and LC1). When present, the light-blue shaded area corresponds to the log-normal fit performed on the corresponding 1D-histogram.

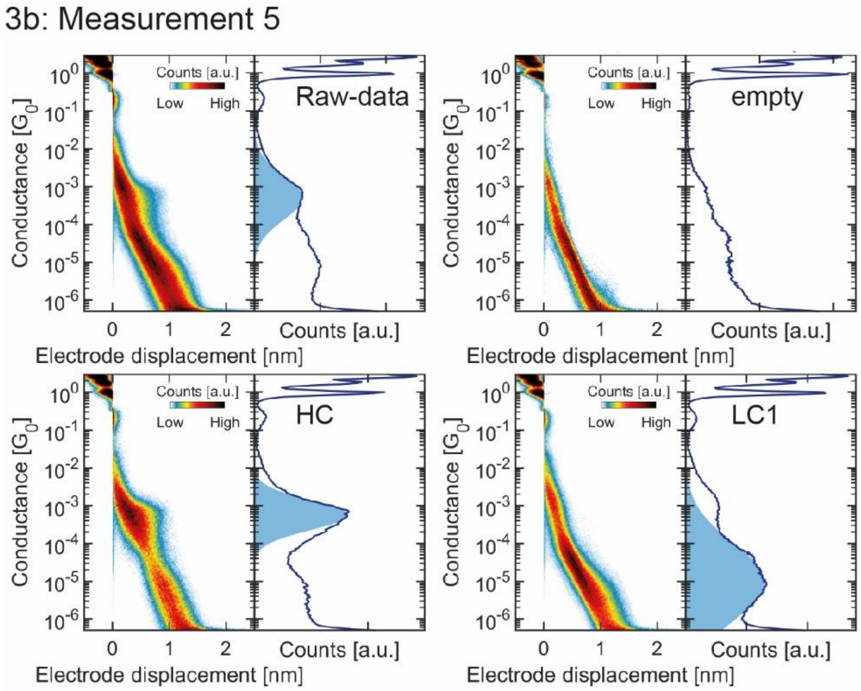

Figure S2.14. 2D and 1D histograms for measurement 5 of molecule 3b; In addition to the data before any classification, the histograms are reported for the traces excluded using the neural network (“empty”) and for the classes identified with clustering analysis (HC, and LC1). When present, the light-blue shaded area corresponds to the log-normal fit performed on the corresponding 1D-histogram.

3 STMBJ measurements and data

From the raw data, it is performed a selection of traces with the expected shape for a molecular junction, removing “empty” and “bad” traces that come as a result of several events during the experiment like the saturation of the electrical signal, mechanical perturbations, or junctions with a non-well-defined breaking. To this “cleaned” dataset, an unsupervised algorithm for clustering, in this case, the k-means, is applied. The parameters used to introduce the data in the algorithm are the same as the ones used for the MCBJ data classification: For each trace, a linear vector is created appending consecutively the raws of a 2D-histogram with 40 bins between -2 and -6 log(G/G_0) values and 30 bins between 0 and 1.5 nm of displacement. Besides, another vector from a 1D-histogram with 100 bins between -1 and -6.5 log(G/G_0) values is appended.

Up to 6 clusters may be separated to isolate both different conductance behaviors and tunnel traces. For some clusters with a molecular feature, the algorithm may be applied again to remove the remaining tunnel traces. Then all the clusters with molecular features are put together and the algorithm is applied one more time considering the number of clusters accordingly to the selection observed in the previous process, since clusters belonging to the same behavior may have been separated, or traces belonging to a different cluster may have been misclassified.

3.1 Conductance values and histograms

Table S3.1. Summary table for the results of all the molecules. The columns report the conductance values as log(G/G_0) for HC, LC1, and LC2 classes and the percentage of traces with a molecular feature with respect to the total number of

traces for HC, LC1, LC2, and Double classes, including the sum of all the classes.

Molecule	*All*	*HC*		*LC1*		*LC2*		*Double*
	% Raw	log(G/G$_0$)	% Raw	log(G/G$_0$)	% Raw	log(G/G$_0$)	% Raw	% Raw
**1a**	28.0	-2.6	15.8	-4.5	1.3	-5.5	1.7	9.2
**2a**	25.0	-2.7	8.8	-3.8<br>-4.7	9.4<br>6.8	//	//	//
**3a**	24.9	-2.6	13.8	-3.8	21.3	-5.6	4.6	1.2
**1b**	22.3	-3.2	12.8	-5.0	2.3	//	//	6.8
**2b**	14.6	-3.1	8.5	-4.5	6.1	//	//	//
**3b**	6.1	-3.1	3.3	-5.0	2.8	//	//	//

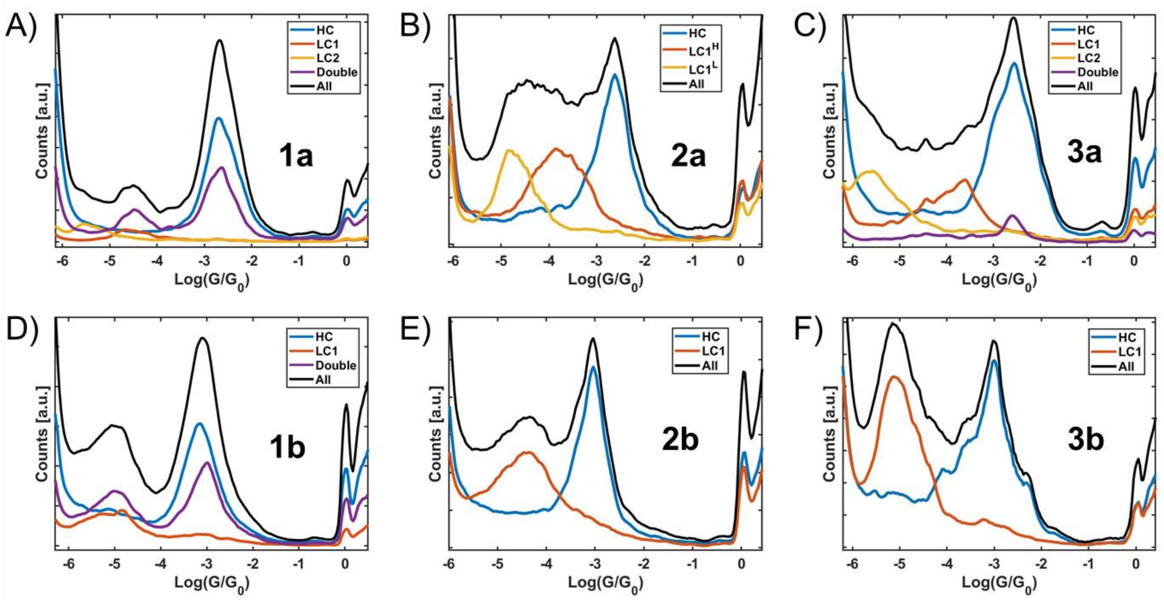

Figure S3.1. Conductance (Log(G/G_0)) 1D-histograms for all the compounds, -SAc compounds **1a**, **2a** and **3a** in the upper part (A-C) and -SMe compounds **1b**, **2b** and **3b** in the lower part (D-F). The peaks correspond to the different classes HC, LC1, LC2 and Double identified by colors in the legend, including the histogram with all the classes together in black.

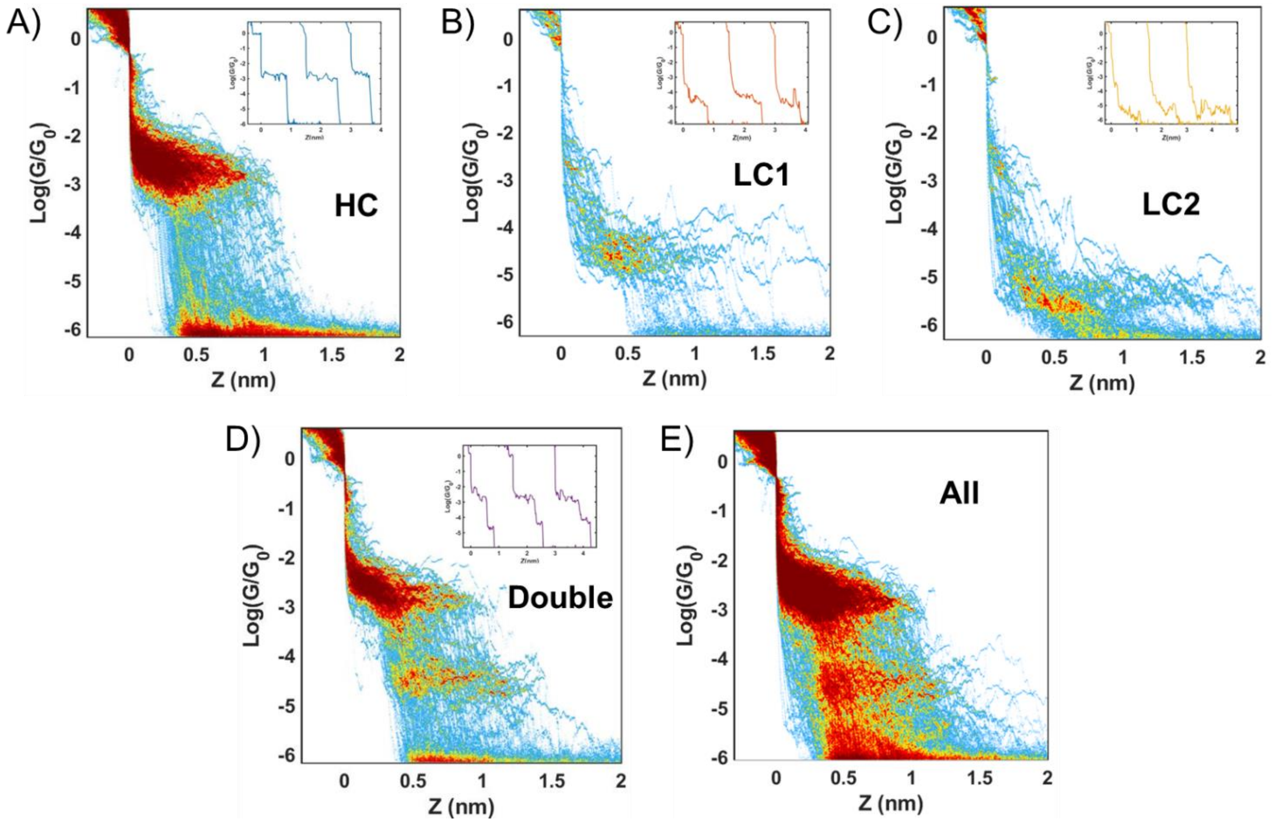

Figure S3.2. Conductance (Log(G/G_0)) / displacement (Z) 2D-histograms of molecule **1a** for classes HC, LC1, LC2 and Double, plus all combined (A-E) (inset) Examples of individual traces for each class.

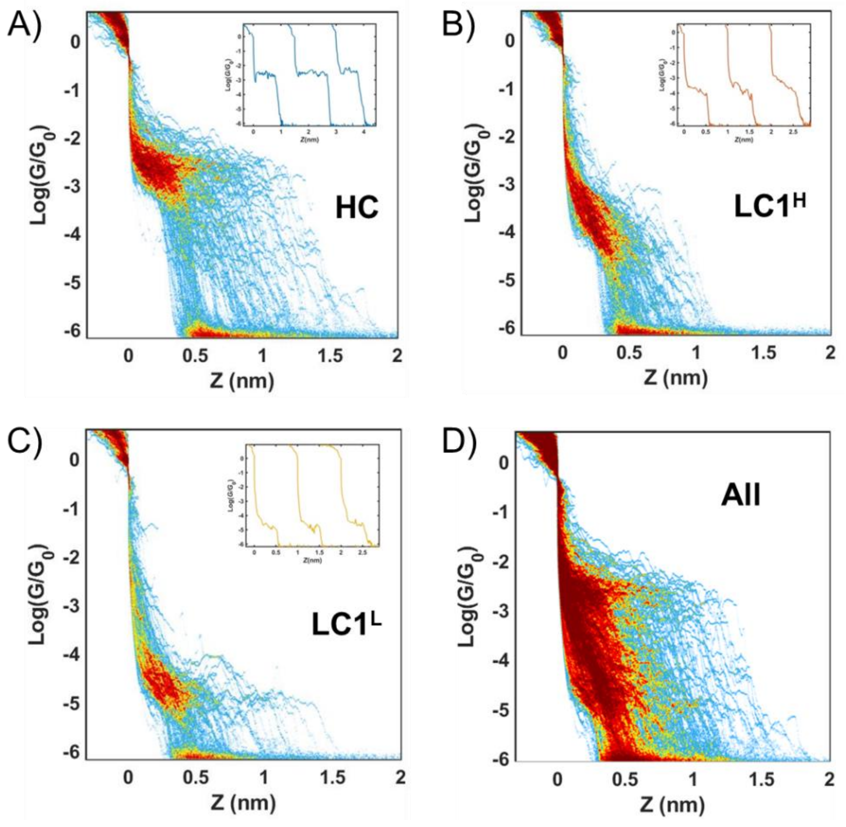

Figure S3.3. Conductance (Log(G/G_0)) / displacement (Z) 2D-histograms of molecule **2a** for classes HC, LC1^{H}, and LC1^{L}, plus all combined (A-D) (inset) Examples of individual traces for each class.

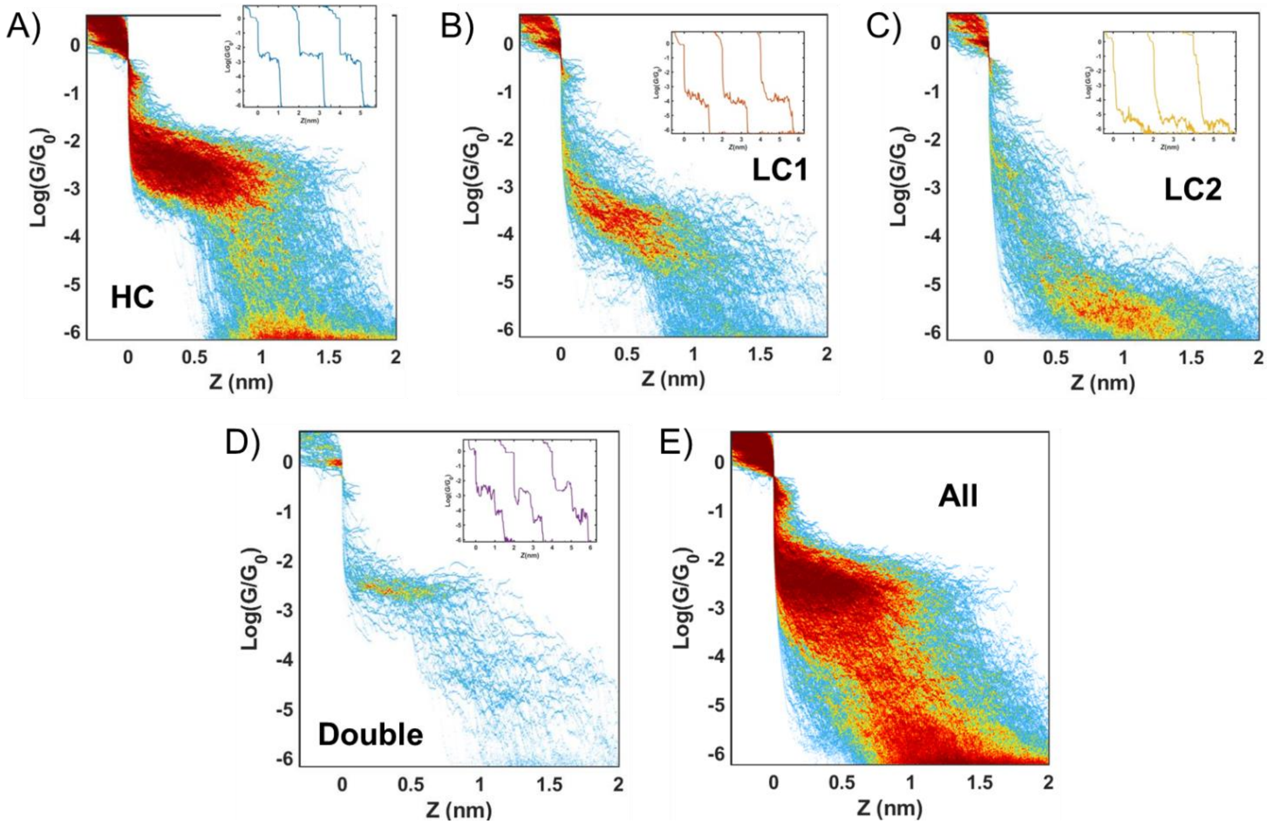

Figure S3.4. Conductance (Log(G/G_0)) / displacement (Z) 2D-histograms of molecule **3a** for classes HC, LC1, LC2 and Double, plus all combined (A-E) (inset) Examples of individual traces for each class.

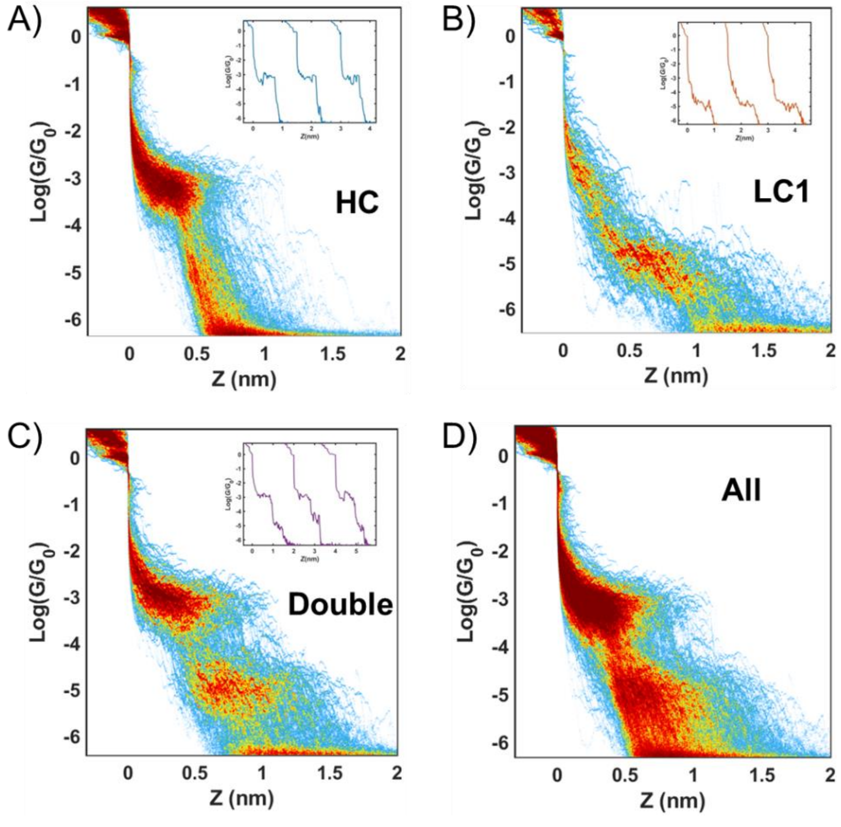

Figure S3.5. Conductance (Log(G/G_0)) / displacement (Z) 2D-histograms of molecule **1b** for classes HC, LC1, and Double, plus all combined (A-D) (inset) Examples of individual traces for each class.

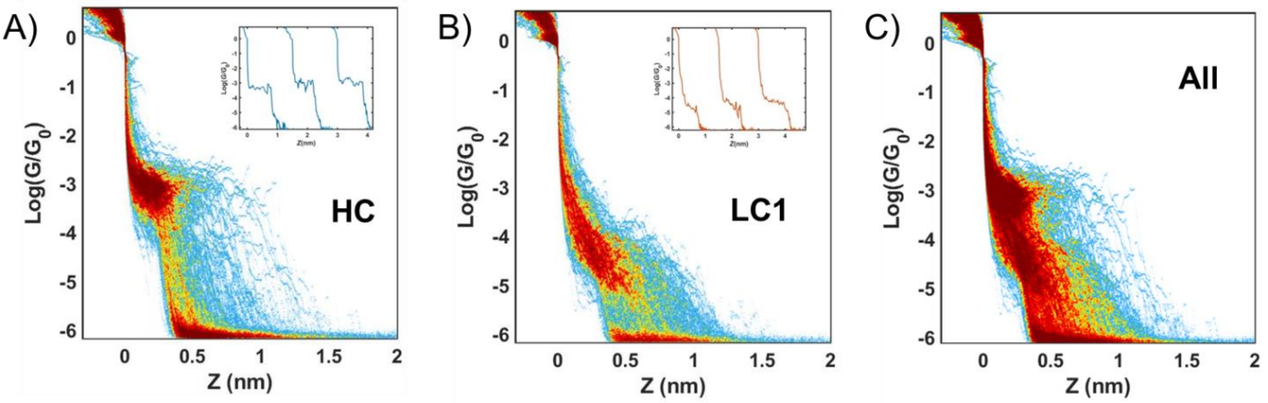

Figure S3.6. Conductance (Log(G/G_0)) / displacement (Z) 2D-histograms of molecule **2b** for classes HC and LC1, plus all combined (A-C) (inset) Examples of individual traces for each class.

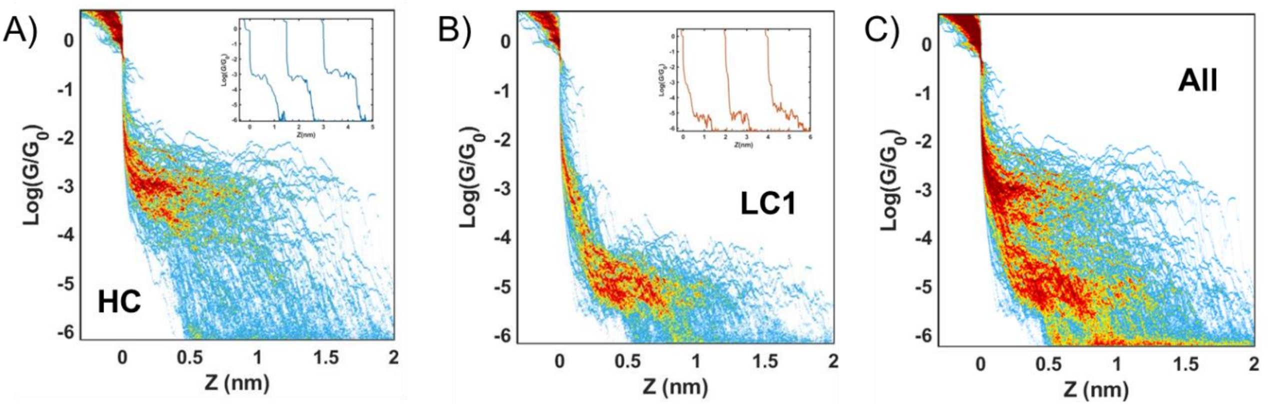

Figure S3.7. Conductance (Log(G/G_0)) / displacement (Z) 2D-histograms of molecule **3b** for classes HC and LC1, plus all combined (A-C) (inset) Examples of individual traces for each class.

3.1.1 Comparison of MCBJ and STMBJ measurements

Overall, MCBJ and STMBJ measurements show very similar classes across the series molecules, with similar conductance values and trends. However, there are some noticeable differences between MCBJ and STMBJ measurements. In general, STMBJ traces are statistically shorter than MCBJ traces, which is related to the stability of the junctions. This is not the case for compounds **3a** and **3b** traces, which are remarkably large for STMBJ. In addition to this, for compound **1a** it seems to be difficult to form a junction in MCBJ experiments, while in STMBJ experiments the percentage of LC traces is low compared to the HC. Finally, a remarkable fact is the appearance of clusters formed by double plateau traces in compounds **1a** and **1b**. It was possible to isolate a cluster of these traces also for compound **3a**, but not many. In fact, this behavior is observable for all the compounds in STMBJ experiments. Nevertheless, for measurements of compounds **1a** and **1b**, the amount of traces in the Double class is even higher than for LC. On the other hand, in MCBJ measurements, this kind of behaviour is not found in clustering. Some measurements show classes with two steps (see Figure S2.4, S2.9, or S2.10), but such classes do not show two clear plateaus as it is found in STMBJ. Additionally, the presence of two-peak clusters in MCBJ measurements is not consistent across all molecules, and not even across different measurements on the same molecule. This Double-class might be related to π-stacking, since it is present in the measurements at 1 mM concentration, while when measuring at 0.1 mM concentration, the same as for the MCBJ experiments, the class is no longer found when clustering (Figure S3.8).

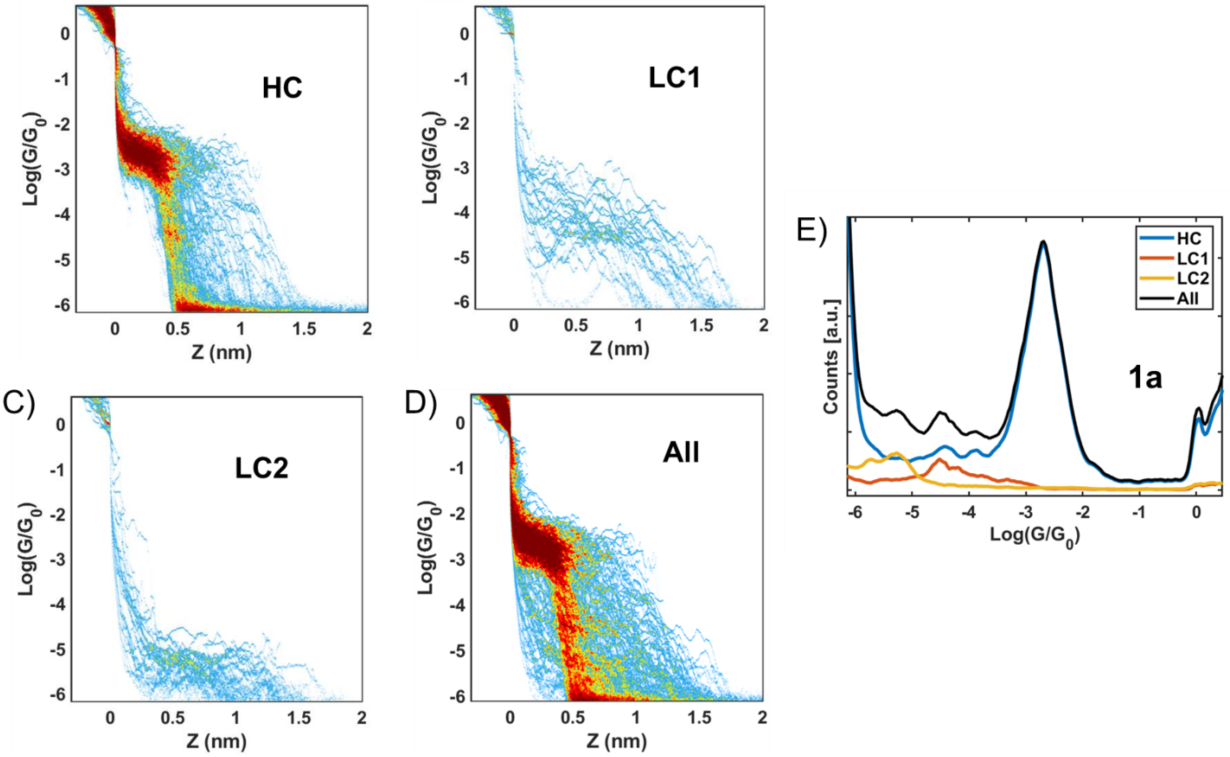

Figure S3.8. Conductance (Log(G/G_0)) / displacement (Z) 2D-histograms of molecule **1a** at a concentration 0.1 mM, and order of magnitude lower than the previous measurements, for classes HC, LC1, and LC2, plus all combined (A-D). (E) Conductance (Log(G/G_0)) 1D-histogram. The peaks correspond to the different classes HC, LC1 and LC2 identified by colors in the legend, including the histogram with all the classes together in black.

3.2 Thermopower

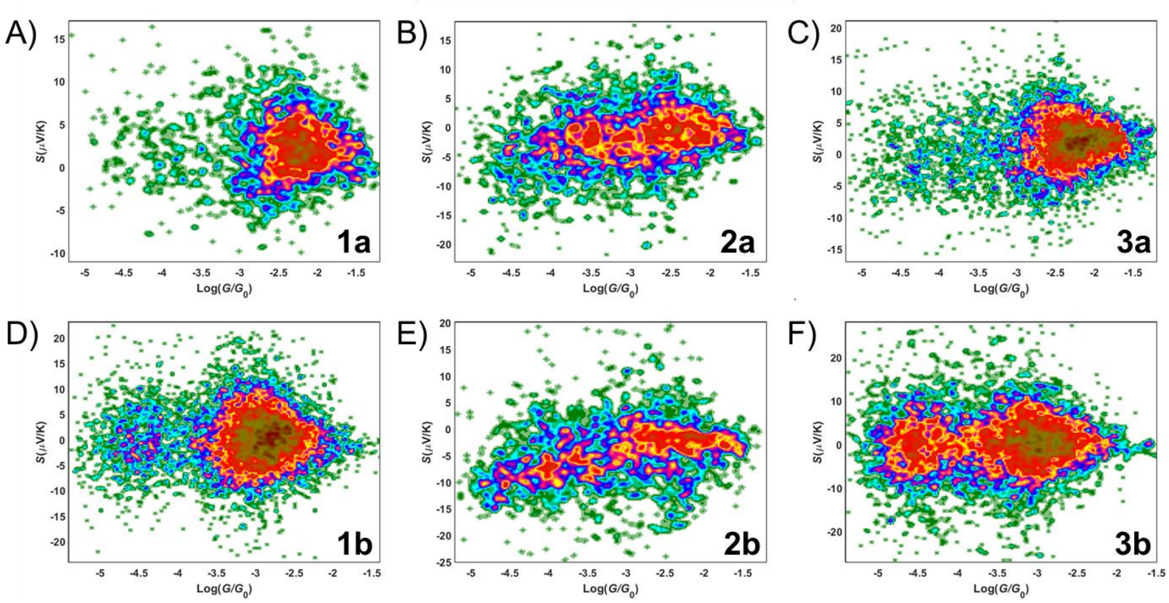

Figure S3.9. Seebeck Coefficient (S) / Conductance (Log(G/G_0)) histograms for all the compounds, -SAc compounds **1a**, **2a** and **3a** in the upper part (A-C) and -SMe compounds **1b**, **2b** and **3b** in the lower part (D-F). The distribution of the points follows the tendency of the 1D-histograms in Figure S.3.1.

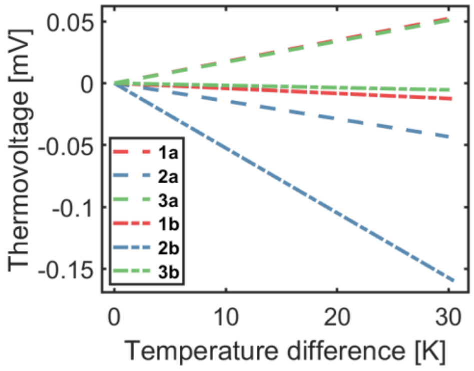

Figure S3.10. Linear regressions of the temperature difference dependence of the thermovoltage measurements for all the compounds, centered to the origin for comparison. The slope of each line is the total Seebeck coefficient corresponding to the values in Table 2.

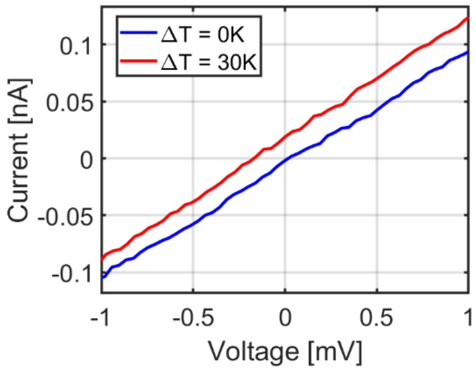

Figure S3.11. IV-curves measured for compound **2b** corresponding to class HC, under tip-sample temperature difference of 0K and 30 K. The slope of the curves is the conductance and the offset in voltage at 30K is the thermovoltage. Therefore, conductance and thermopower are simultaneously determined for every measurement in every single-molecule junction.

Table S3.2. Length of molecular junctions obtained from MCBJ and STMBJ experiments.

Molecule	*HC length (nm)*		*LC1 length (nm)*		*LC2 length (nm)*	
	MCBJ	STMBJ	MCBJ	STMBJ	MCBJ	STMBJ
**1a**	0.70 ± 0.06	0.72 ± 0.17	0.90 ± 0.04	1.09 ± 0.32	1.41 ± 0.09	1.29 ± 0.15
**2a**	0.79 ± 0.19	0.78 ± 0.22	0.70 ± 0.12<br>0.92 ± 0.09	0.61 ± 0.54<br>0.92 ± 0.58	//	//
**3a**	0.80 ± 0.11	1.37 ± 0.16	0.94 ± 0.10	1.58 ± 0.19	2.00 ± 0.38	2.08 ± 0.14
**1b**	0.59 ± 0.08	0.62 ± 0.12	1.07 ± 0.11	1.44 ± 0.26	//	//
**2b**	0.60 ± 0.08	0.56 ± 0.17	1.08 ± 0.08	0.91 ± 0.14	//	//
**3b**	0.64 ± 0.08	1.29 ± 0.24	1.14 ± 0.08	1.48 ± 0.30	//	//

4 Theory and modelling

4.1 Computational methods

Geometry optimization: The geometry of each structure studied in this paper was relaxed to the force tolerance of 10 meV/Å using the SIESTA[6] implementation of density functional theory (DFT), with a double-ζ polarized basis set (DZP) and the Local Density Approximation (LDA) functional with CA parameterization. A

real-space grid was defined with an equivalent energy cut-off of 250 Ry. To calculate molecular orbitals and spin density of gas phase molecules, an experimentally parameterised B3LYP functional was employed using Gaussian g09v2 with 6-311++g basis set and tight convergence criteria.

Electron transport: To calculate the electronic properties of the device, from the converged DFT calculation, the underlying mean-field Hamiltonian H was combined with our quantum transport code, GOLLUM[7,8]. This yields the transmission coefficient $T_e(E)$ for electrons of energy E (passing from the source to the drain) via the relation $T_e(E) = Tr(\Gamma_L^e(E) G_e^R(E) \Gamma_R^e(E) G_e^{R\dagger}(E))$ where $\Gamma_{L,R}^e(E) = i(\sum_{L,R}^e(E) - \sum_{L,R}^e{}^\dagger(E))$ describes the level broadening due to the coupling between left L and right R electrodes and the central scattering region, $\Sigma_{L,R}^e(E)$ are the retarded self-energies associated with this coupling, and $G_e^R = (ES - H - \sum_L^e - \sum_R^e)^{-1}$ is the retarded Green's function, where H is the Hamiltonian and S is the overlap matrix obtained from SIESTA implementation of DFT. DFT+$\sum$ approach has been employed for spectral adjustment[8].

Electrical conductance: Using the approach explained in[8,9], the electrical conductance $G = G_0 \int_{-\infty}^{+\infty} dE\, T_e(E)(-\partial f_{FD}(E,T,E_F)/\partial E)$ is calculated from the electron transmission coefficient $T_e(E)$ where $f_{FD} = (e^{(E-E_F)/k_B T} + 1)^{-1}$ is the Fermi-Dirac probability distribution function, T is the temperature, E_F is the Fermi energy, $G_0 = 2e^2/h$ is the conductance quantum, e is electron charge and h is the Planck's constant.

Data analysis and theoretical conductance histograms: First, we form a series of junctions with different contacting modalities to electrodes and calculate the electrical conductance G for a range of electrodes Fermi energies. Next, we create the conductance histograms using the calculated conductance for each junction and for a wide range of E_F between the HOMO-LUMO gap. To ensure including conductance values in co-tunnelling regime, the range of E_F is chosen such that they do not include HOMO and LUMO resonances. For this, $E_H+\Delta/5 < E_F < E_L-\Delta/5$ where $\Delta = E_L-E_H$ is the energy gap and E_H and E_L are energy of HOMO and LUMO, respectively. The peaks in conductance histograms are fitted with a log-normal distribution and their centre is defined as the most probable conductance.

4.2 Molecular orbitals

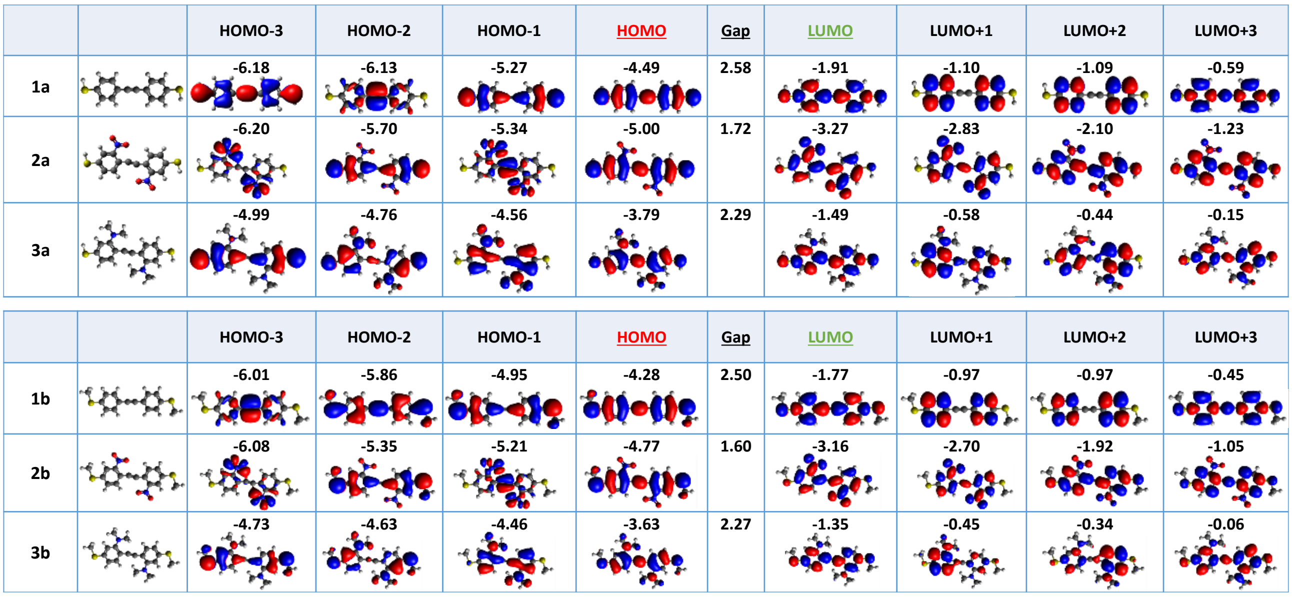

	HOMO-3	HOMO-2	HOMO-1	HOMO	Gap	LUMO	LUMO+1	LUMO+2	LUMO+3
1a	-6.18	-6.13	-5.27	-4.49	2.58	-1.91	-1.10	-1.09	-0.59
2a	-6.20	-5.70	-5.34	-5.00	1.72	-3.27	-2.83	-2.10	-1.23
3a	-4.99	-4.76	-4.56	-3.79	2.29	-1.49	-0.58	-0.44	-0.15

	HOMO-3	HOMO-2	HOMO-1	HOMO	Gap	LUMO	LUMO+1	LUMO+2	LUMO+3
1b	-6.01	-5.86	-4.95	-4.28	2.50	-1.77	-0.97	-0.97	-0.45
2b	-6.08	-5.35	-5.21	-4.77	1.60	-3.16	-2.70	-1.92	-1.05
3b	-4.73	-4.63	-4.46	-3.63	2.27	-1.35	-0.45	-0.34	-0.06

Figure S4.1. Molecular orbitals of molecule **1-3** with thiol (**a**) and SME (**b**) anchor groups. All energy units are eV.

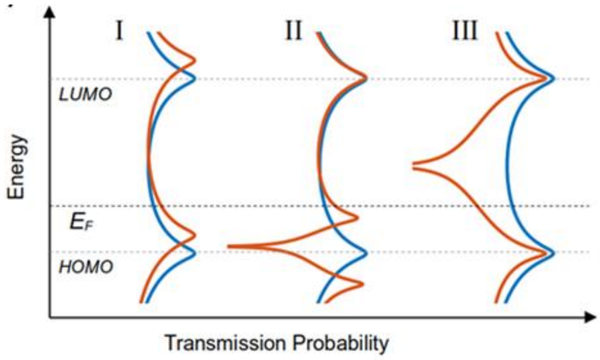

Figure S4.2. The effect of substituents on the electron transmission probability through junctions.

The substituents (e.g. side groups) are expected to affect electrical conductance in three ways. They might tune energy levels of the molecular backbone (e.g. shift the position of HOMO and LUMO) just like doping in the semiconductors (Figure S4.2)[10,11] leading to a shift in the transport resonances (Figure S4.2I). If the energy level of a side group happens to be in the HOMO-LUMO gap of the molecule, it can also open a new transport channel (e.g. due to quantum interference) and affect the electrical conductance (Figure S4.2II)[12–16]. Side groups can also affect electrical conductance if they alter conjugation of the system[17–21] which is not our focus in the present study (Figure S4.2III).

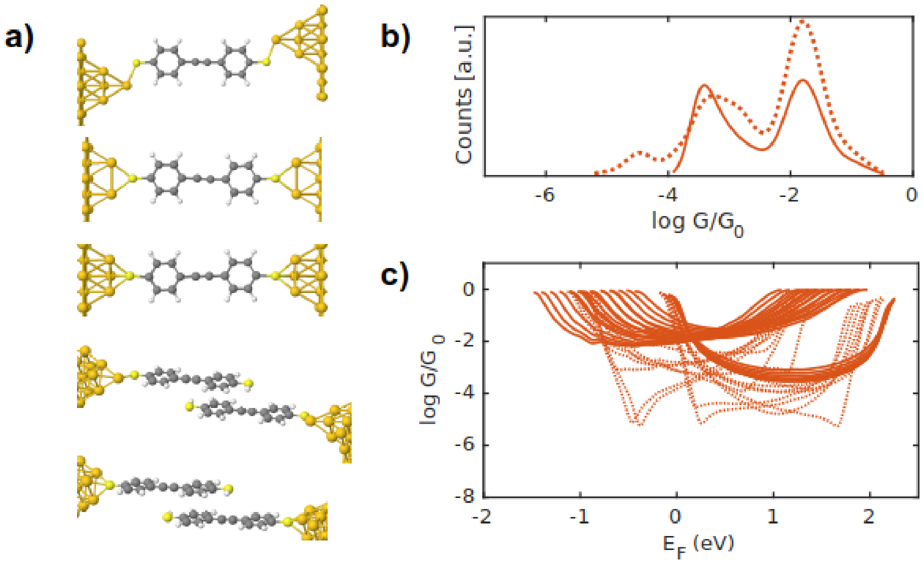

Figure S4.3. Conductance of **1a** with additional conformations including pi-pi stacking. (a) examples of different molecule electrodes conformations. (b) associated conductance histogram with (dashed line) and without (solid line) pi-pi stacking conformations. (c) correspondence G vs E_F for full range of energies between HOMO and LUMO resonances.

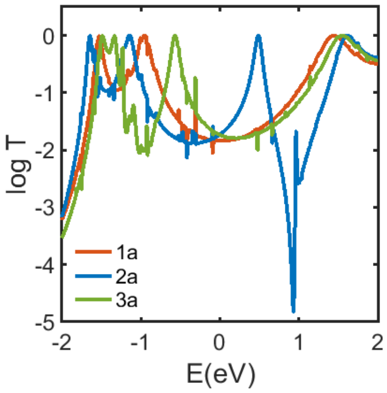

Figure S4.4. Example of $T(E)$ for **1a**, **2a** and **3a** with similar anchor-electrode conformations. The HOMO-LUMO gap of **2a** is smaller compared to **1a** and **3a** and a new resonance close to E=0eV is formed in **2a** (blue line) between the HOMO-LUMO of tolane **1a** (red line). This additional resonance is due to the nitro groups. Around DFT Fermi energy (E=0eV), the transport is dominated by HOMO for **1a** and **3a** while transport is through LUMO for **2b**. Around this Fermi energy, a positive Seebeck coefficient (S) is expected for **1a** and **3a** whereas a negative S is predicted for **2a**.

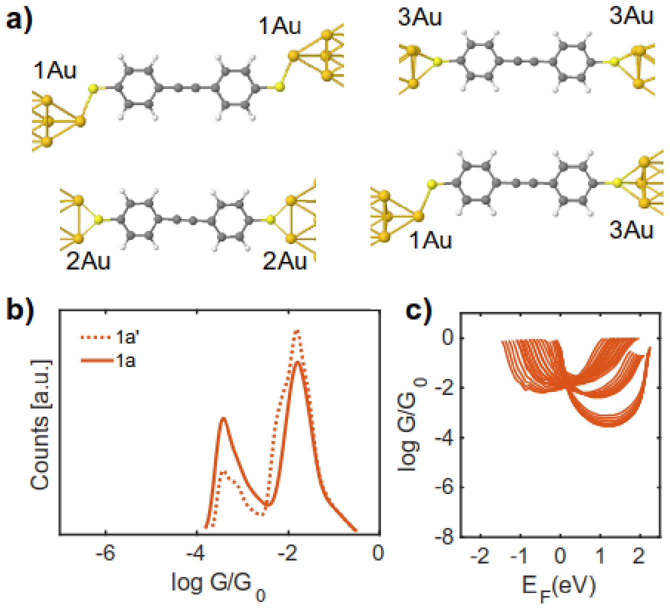

Figure S4.5. The effect of asymmetric contacting to electrode. (a) examples of binding configurations between OPE2 and electrode through 1Au-1Au, 2Au-2Au, 3Au-3Au and 1Au-3Au atoms, (b) conductance histograms with (1a) and without (1a') asymmetric configurations, (c) the conductance of 1a versus E_F with different configurations to electrode. The conductance histogram changes while retaining the main feature without additional asymmetric configurations. There are still two peaks in the histogram, and the mean of each peak is almost the same with and without additional configurations.

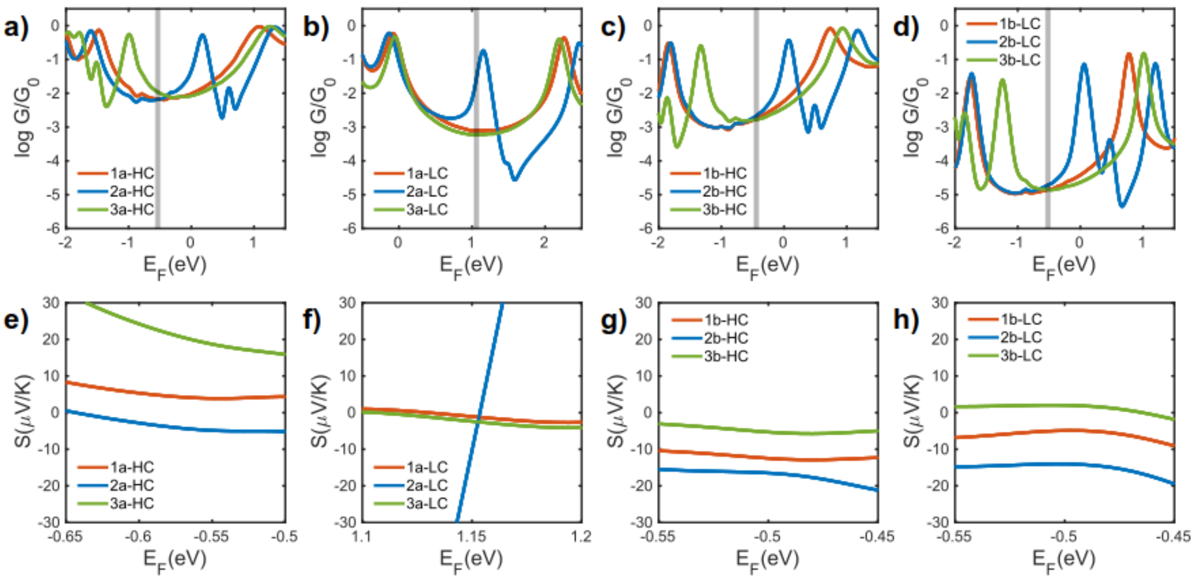

Figure S4.6. The Seebeck coefficient calculations. (a-d) room temperature electrical conductance and (e-h) corresponding Seebeck coefficient of 1a, 2a, 3a, 1b, 2b, 3b with 1Au-1Au (b, d, f, h) and 3Au-3Au (a, c, e, g) electrode structures.

To ensure the impartiality of our calculations, we have kept the procedure the same for forming different configurations with different anchors and molecules. For example, we begin from the ground-state Au-S binding configuration for all molecules and then move the electrodes away from them by the same amount. However, the probability of forming junctions with a certain Au-S distance experimentally depends on the binding energy between Au-S in SAc and SMe, which is different. For longer junctions, Au-S with a SMe anchor is likely to break earlier than the corresponding junction with a SAc anchor. This is supported by our length analysis of the junctions using the experimental conductance traces (see Table S3.2 in the SI), which shows that the junctions formed by SMe anchors are generally shorter (by ~1-2Å) than those of SAc Anchors.

The probability of electron transmission through molecules with SMe anchors is more sensitive to the Au-S distance and decreases rapidly as the distance increases (see Figure 5a). For example, if the Au-S distance is increased by 1-2 Å, the conductance decreases by up to an order of magnitude in junctions with SMe anchors. These low conductance configurations contribute to the low conductance peak in the computed conductance histograms. If we avoid these low conductance junctions, the conductance histograms are less broadened (see Figure S4.7 in the SI). But this is not desirable. The key point is that even without data selection and using a similar and equivalent number of configurations, our proposed method provides qualitative agreement between features observed experimentally and theoretically. This predictive modelling ability of the proposed theoretical method makes it a valuable tool for predicting the conductance of various molecular junctions.

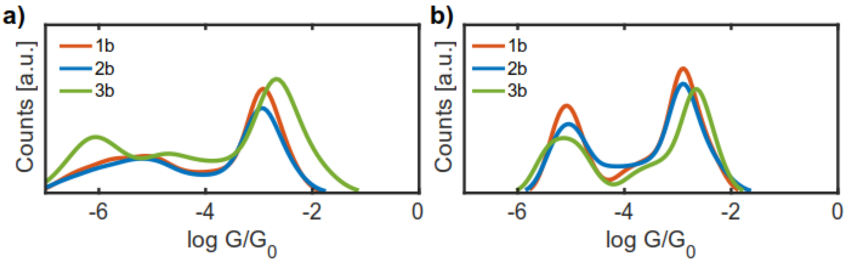

Figure S4.7. Conductance histograms for molecules with SMe anchor. (a) with the same and equivalent number of configurations as junctions with SAc anchor, (b) without junctions with large Au-S distance.

Supporting References

(1) Adeniyi, A. A.; Ngake, T. L.; Conradie, J. Cyclic Voltammetric Study of 2-Hydroxybenzophenone (HBP) Derivatives and the Correspondent Change in the Orbital Energy Levels in Different Solvents. *Electroanalysis* **2020**, *32* (12), 2659–2668. https://doi.org/https://doi.org/10.1002/elan.202060163.

(2) de Leeuw, D. M.; Simenon, M. M. J.; Brown, A. R.; Einerhand, R. E. F. Stability of N-Type Doped Conducting Polymers and Consequences for Polymeric Microelectronic Devices. *Synth. Met.* **1997**, *87* (1), 53–59. https://doi.org/https://doi.org/10.1016/S0379-6779(97)80097-5.

(3) A Crash Course in Photophysics and a Classification of Primary Photoreactions. In *Photochemistry of Organic Compounds*; John Wiley & Sons, Ltd, 2009; pp 25–72. https://doi.org/https://doi.org/10.1002/9781444300017.ch2.

(4) Kingma, D. P.; Ba, J. L. Adam: A Method for Stochastic Optimization. *3rd Int. Conf. Learn. Represent. ICLR 2015 - Conf. Track Proc.* **2015**.

(5) Phys, A.; Cabosart, D.; Abbassi, M. El; Stefani, D.; Frisenda, R.; Calame, M.; Herre, S. J.; Cabosart, D.; Abbassi, E. A Reference-Free Clustering Method for the Analysis of Molecular Break-Junction Measurements A Reference-Free Clustering Method for the Analysis of Molecular Break-Junction Measurements. **2019**, *143102* (March). https://doi.org/10.1063/1.5089198.

(6) Soler, J. M.; Artacho, E.; Gale, J. D.; García, A.; Junquera, J.; Ordejón, P.; Sánchez-Portal, D. The SIESTA Method for Ab Initio Order- N Materials Simulation. *J. Phys. Condens. Matter* **2002**, *14* (11), 2745–2779. https://doi.org/10.1088/0953-8984/14/11/302.

(7) Ferrer, J.; Lambert, C. J.; García-Suárez, V. M.; Manrique, D. Z.; Visontai, D.; Oroszlany, L.; Rodríguez-Ferradás, R.; Grace, I.; Bailey, S. W. D.; Gillemot, K.; Sadeghi, H.; Algharagholy, L. A. GOLLUM: A next-Generation Simulation Tool for Electron, Thermal and Spin Transport. *New J. Phys.* **2014**, *16*, 093029. https://doi.org/10.1088/1367-2630/16/9/093029.

(8) Sadeghi, H. Theory of Electron, Phonon and Spin Transport in Nanoscale Quantum Devices. *Nanotechnology* **2018**, *29* (37), 373001. https://doi.org/10.1088/1361-6528/aace21.

(9) Sadeghi, H.; Sangtarash, S.; Lambert, C. J. Oligoyne Molecular Junctions for Efficient Room Temperature Thermoelectric Power Generation. *Nano Lett.* **2015**, *15* (11), 7467–7472. https://doi.org/10.1021/acs.nanolett.5b03033.

(10) Low, J. Z.; Capozzi, B.; Cui, J.; Wei, S.; Venkataraman, L.; Campos, L. M. Tuning the Polarity of Charge Carriers Using Electron Deficient Thiophenes. *Chem. Sci.* **2017**, *8* (4), 3254–3259. https://doi.org/10.1039/c6sc05283e.

(11) Venkataraman, L.; Park, Y. S.; Whalley, A. C.; Nuckolls, C.; Hybertsen, M. S.; Steigerwald, M. L. Electronics and Chemistry: Varying Single-Molecule Junction Conductance Using Chemical Substituents. *Nano Lett.* **2007**, *7* (2), 502–506. https://doi.org/10.1021/nl062923j.

(12) Sadeghi, H. Quantum and Phonon Interference-Enhanced Molecular-Scale Thermoelectricity. *J. Phys. Chem. C* **2019**, *123* (20), 12556–12562. https://doi.org/10.1021/acs.jpcc.8b12538.

(13) Jiang, F.; Trupp, D. I.; Algethami, N.; Zheng, H.; He, W.; Alqorashi, A.; Zhu, C.; Tang, C.; Li, R.; Liu, J.; Sadeghi, H.; Shi, J.; Davidson, R.; Korb, M.; Sobolev, A. N.; Naher, M.; Sangtarash, S.; Low, P. J.; Hong, W.; Lambert, C. J. Turning the Tap: Conformational Control of Quantum Interference to Modulate Single-Molecule Conductance. *Angew. Chemie - Int. Ed.* **2019**, *58* (52), 18987–18993. https://doi.org/10.1002/anie.201909461.

(14) Mowbray, D. J.; Jones, G.; Thygesen, K. S. Influence of Functional Groups on Charge Transport in Molecular Junctions. *J. Chem. Phys.* **2008**, *128* (11). https://doi.org/10.1063/1.2894544.

(15) Nozaki, D.; Sevinçli, H.; Avdoshenko, S. M.; Gutierrez, R.; Cuniberti, G. A Parabolic Model to Control Quantum Interference in T-Shaped Molecular Junctions. *Phys. Chem. Chem. Phys.* **2013**, *15* (33), 13951–13958. https://doi.org/10.1039/C3CP44578J.

(16) Stadler, R. Conformation Dependence of Charge Transfer and Level Alignment in Nitrobenzene Junctions with Pyridyl Anchor Groups. *Phys. Rev. B - Condens. Matter Mater. Phys.* **2010**, *81* (16), 1–9. https://doi.org/10.1103/PhysRevB.81.165429.

(17) Mishchenko, A.; Vonlanthen, D.; Meded, V.; Bürkle, M.; Li, C.; Pobelov, I. V.; Bagrets, A.; Viljas, J. K.; Pauly, F.; Evers, F.; Mayor, M.; Wandlowski, T. Influence of Conformation on Conductance of Biphenyl-Dithiol Single-Molecule Contacts. *Nano Lett.* **2010**, *10* (1), 156–163. https://doi.org/10.1021/nl903084b.

(18) Alqahtani, J.; Sadeghi, H.; Sangtarash, S.; Lambert, C. J. Breakdown of Curly Arrow Rules in Anthraquinone. *Angew. Chemie* **2018**, *130* (46), 15285–15289. https://doi.org/10.1002/ange.201807257.

(19) Strange, M.; Seldenthuis, J. S.; Verzijl, C. J. O.; Thijssen, J. M.; Solomon, G. C. Interference Enhanced Thermoelectricity in Quinoid Type Structures Interference Enhanced Thermoelectricity in Quinoid Type Structures. **2015**, *084703* (May). https://doi.org/10.1063/1.4913290.

(20) Valkenier, H.; Guédon, C. M.; Markussen, T.; Thygesen, K. S.; van der Molen, S. J.; Hummelen, J. C. Cross-Conjugation and Quantum Interference: A General Correlation? *Phys. Chem. Chem. Phys.* **2014**, *16* (2), 653–662. https://doi.org/10.1039/C3CP53866D.

(21) Valkenier, H.; Markussen, T.; Thygesen, K. S.; Hummelen, J. C.; Molen, S. J. Van Der. Observation of Quantum Interference in Molecular Charge Transport. **2012**, *7* (May), 305–309. https://doi.org/10.1038/nnano.2012.37.
